# Skeletal rearrangement of [4]helicenes under acidic conditions: dynamic chirality and improved properties by subsequent peripheral editing

**DOI:** 10.1039/d6sc03190k

**Published:** 2026-05-13

**Authors:** Arthur Gaucherand, Romain Duwald, Christelle Herse, Céline Besnard, Gennaro Pescitelli, Jérôme Lacour

**Affiliations:** a Department of Organic Chemistry, University of Geneva Quai Ernest Ansermet 30 CH-1211 Geneva 4 Switzerland Jerome.Lacour@unige.ch; b Laboratory of crystallography, University of Geneva Quai Ernest Ansermet 24 CH-1211 Geneva 4 Switzerland; c Department of Chemistry and Industrial Chemistry, University of Pisa Via Moruzzi 13 56124 Pisa Italy

## Abstract

Cationic diaza[4]helicenes are attractive fluorescent scaffolds, yet access to unsymmetrical substitution patterns remains limited. Here, we report that chiral 1,13-dimethoxyquinacridinium (DMQA) derivatives undergo an acid-mediated skeletal rearrangement after hydride or methyl addition, enabling the sequential migration of methoxy groups and providing unprecedented 1,11- and 3,11-substituted cationic [4]helicene regioisomers. Following aerobic photooxidation, the rearranged helicenes were isolated and characterized by spectroscopy, electrochemistry, crystallography, and computation. Relative to the parent 1,13-DMQA scaffold, these core-edited systems display progressive planarization, altered redox behavior, and marked hypsochromic shifts in both absorption and emission, together with improved fluorescence efficiencies. The mono-rearranged platform further enables selective peripheral editing through S_N_Ar substitution and regioselective demethylation/refunctionalization, giving access to a broad family of *O*- and *N*-substituted dyes with finely tunable optical properties. Importantly, relocation of one methoxy group away from the helical groove strongly lowers the configurational barrier, affording configurationally labile helicenes with enantiomerization barriers of about 19 kcal mol^−1^. This dynamic chirality allows asymmetric induction studies through covalently bound chiral appendages and ion pairing with an enantiopure TRISPHAT anion, leading to moderate diastereomeric enrichment and measurable ECD responses.

## Abstract 2

Skeletal rearrangement converts 1,13-DMQA into new 1,11- and 3,11-regioisomeric cationic [4]helicenes. Selective peripheral editing tunes optical properties while loss of steric congestion unlocks dynamic chirality and asymmetric induction.

## Graphical abstract

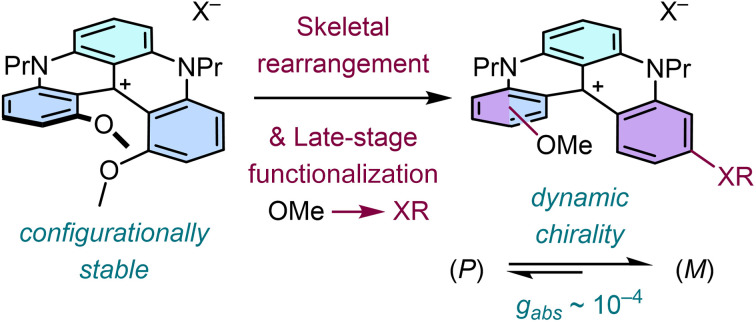

## Introduction

Helicenes, *ortho*-fused polyaromatics, are chiral by virtue of the repulsion between the terminal rings or substituents. Their delocalized π systems generate strong interest from chemistry to biology, physics and materials science alike.^1^ Helicenes – and heterohelicenes – are readily prepared by a wide variety of synthetic methods, providing large arrays of core structures.^2^ Introduction of peripheral substituents, of importance to fine-tune electronic and (chiro)optical properties, is not always trivial. Often, desired functional groups must be introduced early in synthetic approaches requiring, as a consequence, the repetition of multistep sequences for each targeted product.^2*i*–*p*^ In this regard, late-stage functionalization (LSF) is an appealing strategy in the context of helicenes. It remains unfortunately challenging in many instances. The presence of numerous C(sp^2^)–H bonds disfavors simple chemo- and regioselectivity.^3^ Sustained efforts toward novel methods of peripheral editing are investigated.^3*k*–*m*^

**Fig. 1:**
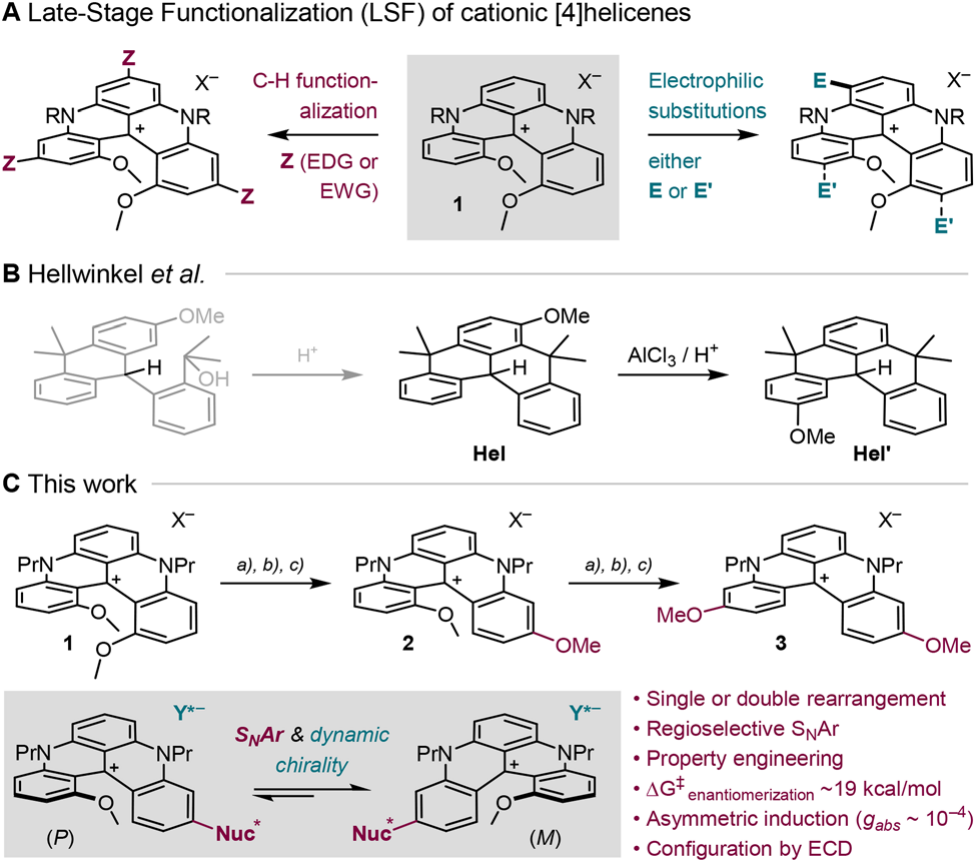
(A) Functionalization of cationic [4]helicene 1 (1,13-DMQA). (B) Acid-mediated rearrangement of Hel into Hel′. (C) Access to novel 1,11-DMQA 2 and 3,11-DMQA 3. (a) NaBH_4_ or MeLi; (b) MeSO_3_H; (c) photooxidation in air. Nuc* = achiral or stereodefinite OR, NHR, NR_2_. Y*^−^ = BF_4_^−^ or TRISPHAT.

One class of derivatives presenting a remarkable LSF propensity is that of cationic diaza[4]helicenes 1 (Fig. 1A). These configurationally stable 1,13-dimethoxyquinacridinium derivatives (Δ*G*^‡^_racem_> 40 kcal mol^−1^) are readily prepared on a multigram scale (>40 g), in two steps from dimethylresorcinol.^4^ These compounds 1 present a remarkable cationic stability (p*K*_R+_ 19)^4*b*,*c*^ and, somewhat surprisingly, a strong nucleophilic character that can be harnessed for regioselective electrophilic substitutions of diverse auxochromic functional groups, at positions *ortho*/*para* to the bridging *N*-atoms in particular (Fig. 1A).^5^ Furthermore, LSF is amenable *via* metal catalysis by either metal carbene insertions or direct triple C–H borylations.^6^ In this latter case, least hindered positions are favored under Ir-catalysis.^7^ After C–Bpin cleavage and exchange, three electron-donating groups (EDGs) or electron-withdrawing groups (EWGs) can be introduced *para* to the formal positive charge, providing a maximal influence of these substituents. At this stage, it was clear that the introduction of a single *para*-substituent would be highly desirable, to even more precisely tune electronic and optical properties of such dyes and fluorophores. It was then debatable whether it could be achieved more efficiently through a skeletal rearrangement rather than a *de novo* substitution.

In fact, helicenes can rearrange under rather forceful conditions (thermal, photochemical, Brønsted/Lewis acids, oxidative, *etc.*) to lead to products of intramolecular cyclization,^8^ ring contraction or ring expansion,^8*a*,*b*,9^ but also to the repositioning of substituents on the helical framework forming original regioisomers of the starting materials. Of particular interest to this study is the observation by Hellwinkel *et al.* that reduced all-carbon [4]helicene Hel transforms into its regioisomer Hel′ upon acid treatment (Fig. 1B).^10^ Nucleophilic addition to the carbenium center of 1 was therefore expected to yield an electron-rich intermediate prone to acid-promoted Friedel–Crafts fragmentation, allowing the sequential migration of the internal methoxy groups toward the peripheral *para* positions. Such a skeletal rearrangement would give access to regioisomeric DMQA scaffolds not available by direct functionalization, with direct consequences on photophysical properties and configurational stability.

Herein, in this context, we demonstrate that a combination of (i) nucleophilic additions (NaBH_4_ or MeLi) to the cationic center of [4]helicene 1 and (ii) Brønsted acid activation affords the successive “migration” of the internal methoxy groups toward peripheral *para* positions (Fig. 1C). Aerobic photooxidation then affords rearranged cations 2 and 3, presenting modified structural, electronic and optical properties. For instance, hyperchromism and hypsochromism demonstrate a stronger electron-donating efficiency of the *para* MeO substituents. Product 2 is also a substrate for further LSF. Aromatic nucleophilic substitutions (S_N_Ar) with diverse alcohols and amines afford products 4 and 5 while regioselective demethylation/refunctionalization of the more accessible *p*-OMe substituent generates phenol 6 and ester 7. All subsequent optical properties are in line with the expected electron-donating (or withdrawing) influence of the modified groups. Finally, the configurational stability of derivatives 2 (*p*-OMe), 4 (*p*-OR) and 5 (*p*-NR_2_) is drastically reduced with a single MeO substituent inside the helical groove (Δ*G*^‡^ ∼19 ± 1 kcal mol^−1^), opening the door to asymmetric induction (dynamic chirality) studies monitored by ^1^H-NMR and electronic circular dichroism (ECD) spectroscopy. The presence of intra- and/or intermolecular chiral auxiliaries leads to the preferred formation of diastereomeric species with moderate levels of selectivity (diastereomeric ratio, *d*. *r*. up to 2 : 1); the favored sense of induction being assigned by ECD, with experimental |*g*_abs_| values up to 9‧10^−5^, allied with TD-DFT investigations.

## Results and discussion

### Skeletal rearrangement procedures

**Scheme 1:**
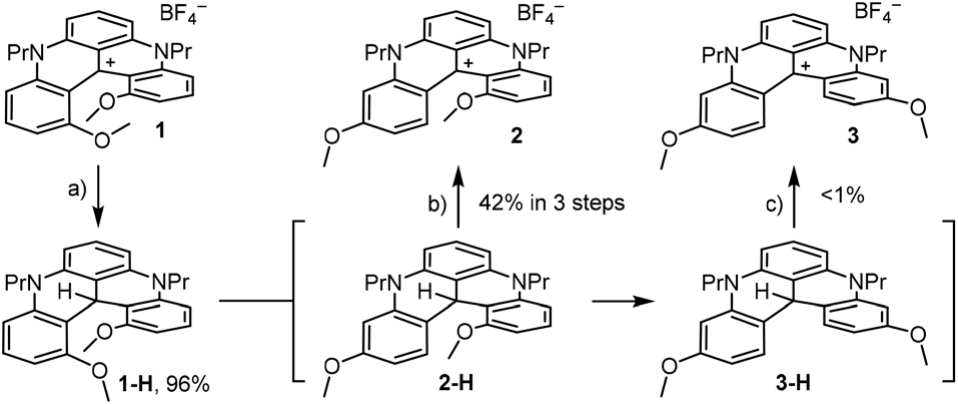
(a) NaBH_4_ (4.0 equiv.), MeOH, rt, 5 min. (b) MeSO_3_H (2.0 equiv.), DCE, 80 °C, dark, 16 h; air, white light, NaBF_4_ (aq.):DCM, 25 °C, 2 h. (c) TfOH (2.0 equiv.), DCE, 80 °C, dark, 20 h; air, white light, NaBF_4_ (aq.):DCM, 25 °C, 2 h.

1,13-Dimethoxyquinacridinium (1,13-DMQA) 1 was prepared as its tetrafluoroborate salt (BF_4_^−^) in two steps from 1,3-dimethoxybenzene (40 g scale).^4*b*,*d*,*e*^ As mentioned, this carbenium ion is particularly stable,^4*b*,*c*^ yet it is amenable to hydride addition with reagents like NaBH_3_CN or NaBH_4_.^4*b*,11^ With 4 equiv. of sodium borohydride, neutral 1-H was obtained in high isolated yield (96%, Scheme 1). Importantly, treatment of *leuco*1-H under strongly acidic conditions (MeSO_3_H, 2 equiv., p*K*_a_ −1.9)^12^ led after 22 h at 80 °C to a complex crude mixture from which several compounds could be identified, including, as a major component, product 2-H. High-resolution mass spectrometry indicated a derivative of the same molecular mass as 1-H but the lack of *C*_2_-symmetry in the ^1^H NMR spectrum pattern was clearly indicative of a skeletal rearrangement. For 2-H, only a structure with one MeO group inside the groove and one external MeO *para* to the formal center of the helical derivative was possible.^13^ Minor fractions of carbocations 1 and 2 were also observed despite the controlled anaerobic atmosphere (Ar). Stronger acidic conditions (TfOH, 2 equiv., p*K*_a_ −14)^14^ gave evidence for a rearrangement proceeding even further toward the migration of both MeO groups, but isolation of corresponding 3-H was ultimately challenging. To simplify work-up and purification steps, eventually realizing that most interesting compounds are (reoxidized) carbenium ions 2 and 3 and not their hydride precursors, care was taken to treat crude reaction mixtures directly under air and white-light irradiation (2 h) in the presence of aqueous NaBF_4_ (excess). While carbocation 2 could be isolated in 42% yield over the three combined steps, doubly rearranged 3 was isolated with ∼1% yield only.

**Scheme 2:**
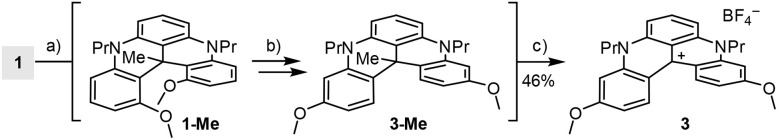
(a) MeLi (5.0 equiv.), THF, −78 °C, 16 h. (b) MeSO_3_H (2.0 equiv.), DCE, 80 °C, dark, 18 h. (c) Air, white light, NaBF_4_ (aq.):DCM, 25 °C, 2 h.

To improve the synthesis of 3, and in light of a mechanism involving most probably reversible Friedel–Crafts reactions (*vide infra*), the rearrangement was attempted with 1-Me (Scheme 2), derived from cation 1 by treatment with methyllithium (excess). With this activated adduct,^15^ the double skeletal rearrangement proceeded toward 3-Me with “milder” methanesulfonic acid. Of interest, subsequent oxidation (air, white light) afforded 3 with a better isolated yield (46%) after a C–Me bond cleavage.^16^

### Skeletal rearrangement – mechanistic rationale

**Scheme 3:**
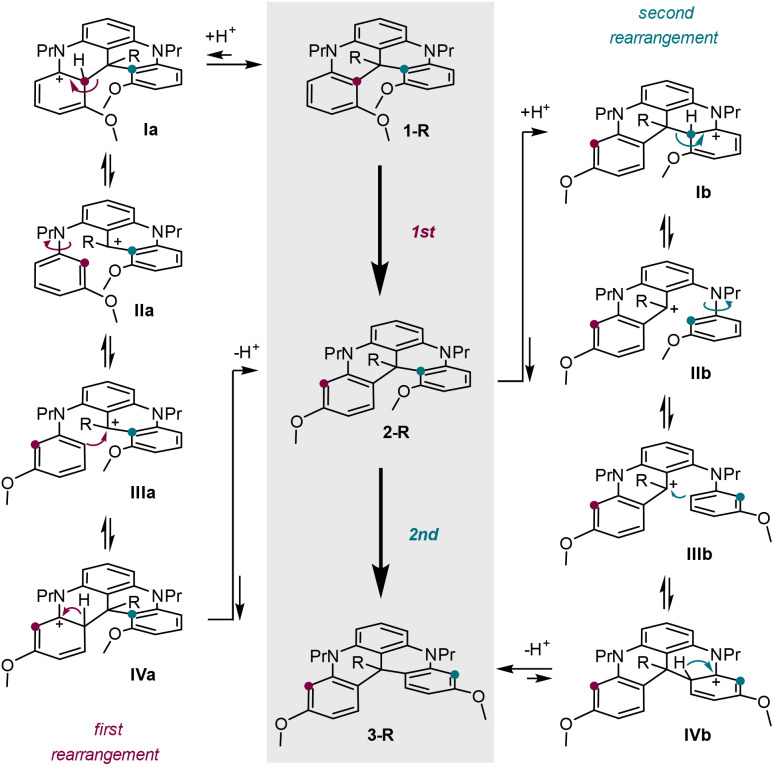
Acid-mediated transformation of 1-R into 2-R and 3-R (central column, R = H or Me). Mechanistic rationale for first (left) and second (right) skeletal rearrangements.

To explain the rearrangement of 1-R (R=H, Me) into corresponding 2-R and then 3-R, a mechanism building on reversible Friedel–Crafts reactions can be considered (Scheme 3).^17^ In fact, thanks to the electron-rich nature of each aromatic group after hydride addition (R=H) or alkylation (R = Me) of the central position, protonation of derivatives 1-R occurs readily under strongly acidic conditions to lead to stabilized Wheland intermediates of type Ia (Scheme 3, left column). Then, helped by the release of strain generated by the proximity of the MeO groups, *retro*-Friedel–Crafts reactions occur,^18^ yielding ring-opened intermediates IIa by fragmentation (C–C bond cleavage). Free rotation around the C–N bond of the detached aromatic ring (IIa → IIIa) then positions the less hindered and more nucleophilic side of the moiety, favoring the reformation of a sterically more favored arenium intermediate (IIIa → IVa). Final proton loss yields 2-R that can be observed spectroscopically.

The next rearrangement, from 2-R to 3-R, follows homologous steps onto the remaining *ortho*-OMe substituted terminal ring (Scheme 3, right column). The presence of the central methyl group in Ib, IIb, IIIb and IVb transforms these cations from secondary to tertiary intermediates, rendering the species more electrofugal and hence better intramolecular leaving groups. This is reflected in the better yield of 3 starting from 1-Me over 1-H under the same conditions (MeSO_3_H, 80 °C).

### Structural crystallographic analysis

**Fig. 2:**
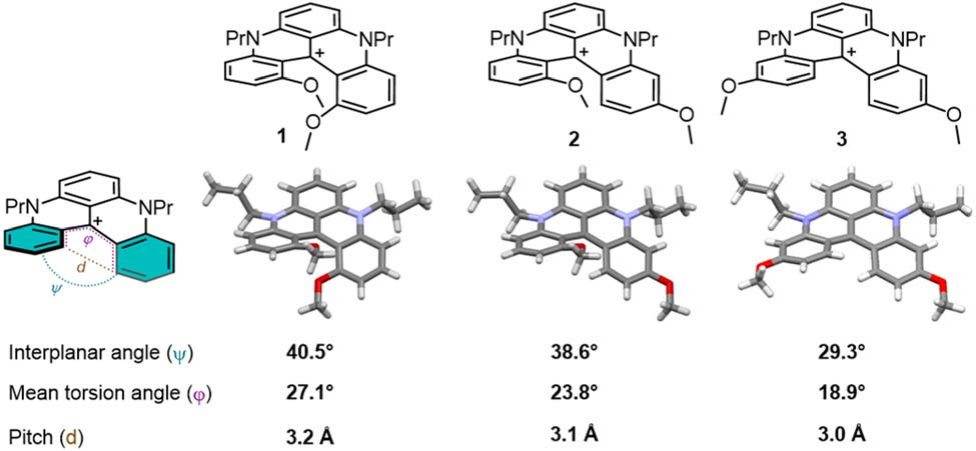
Selected structural features of DMQA 1, 2 and 3.

With rearranged dimethoxyquinacridinium 2 and 3 in hand, attention was immediately given to their geometry. Under slow-evaporation conditions, single crystals were isolated, and X-ray structural analyses were performed (Fig. 2, CCDC 2542615 and 2542616) and compared with those of classical 1 (CCDC 2319499 and 2319500).^19^ To quantify the helicity, three parameters were selected (Fig. 2 and Table S3): (i) the interplanar angle *ψ* between the planes of the terminal rings, which reflects the compactness of the structure, (ii) the mean torsion angle between four consecutive atoms inside the groove *φ*, and (iii) the pitch *d* that measures the distance between the carbons at the cove positions. As it could be expected, the helical deformation decreases from 1 to 2 and then to 3, with *φ* values lessening from 27.1° (1) to 23.8° (2) and 18.9° (3). The skeletal helicity remains nevertheless noticeable despite a gradual loss of steric hindrance inside the cove brought by the lack of MeO group(s). A certain planarization occurs with *ψ* changing from 40.5° → 38.6° → 29.3° for 1 → 2 → 3; the effect of this angle reduction will later be monitored in terms of electronic and optical properties. Consequences of the structural changes will also be investigated in terms of configurational stability (*vide infra*).

### Electrochemical and photophysical properties

**Fig. 3:**
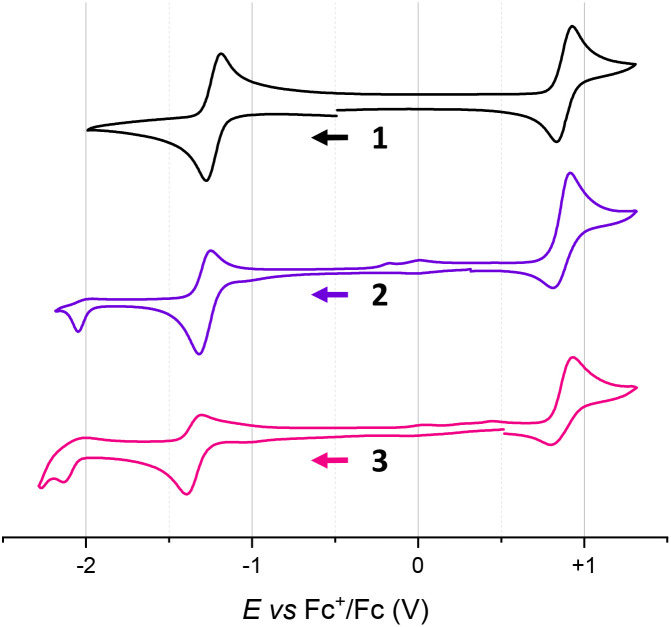
Cyclic voltammetry curves of 1 (black), 2 (purple), and 3 (pink) recorded at the Pt electrode (*Ø* = 3 mm and *ν* = 0.1 V s^−1^ for 1 and *ν* = 0.02 V s^−1^ for 2 and 3) with a concentration of 5 × 10^−4^ M in dry acetonitrile under an inert atmosphere using [^*n*^Bu_4_N][PF_6_] 10^−1^ M as supporting electrolyte. The arrow indicates the direction of the scan (negative potential first).

Compounds 2 and 3 were studied by cyclic voltammetry (CV) in acetonitrile under an inert atmosphere (Ar) and the results were compared with those of 1 (Table 1 and Fig. 3). In effect, precursor 1 presents reversible first reduction at −1.23 V (*vs.* Fc^+^/Fc, Δ*E*_p_ = 75 mV and *i*_b_/*i*_f_ ∼ 1), a second irreversible reduction at −2.11 V (not shown), and a quasi-reversible oxidation at +0.88 V.^4*b*,20^ Peripheral editing into 2 and 3 gradually influenced the first reductions that became more difficult (*E*_1/2_^red^ −1.28 V and −1.35 V for 2 and 3, respectively), while oxidation potentials remained constant (*E*_1/2_^ox^ +0.87 V).

**Table 1:** Electrochemical data of DMQA regioisomers in acetonitrile^a^

Molecule	*E*_1/2_^red,1^ (Δ*E*_p_)	*i*_b_/*i*_f_	*E*_1/2_^ox^ (Δ*E*_p_)	*i*_b_/*i*_f_	Electrochemical energy gap (eV)
1^b^	−1.23 (75)	0.96	+0.88 (90)	0.83	1.98
2^c^	−1.28 (68)	0.67	+0.87 (99)	0.59	1.99
3^c^	−1.35 (90)	0.51	+0.87 (126)	0.50	2.08

a *E*_1/2_ (V, *vs.* Fc^+^/Fc) and peak-to-peak separations (Δ*E*_p_ in mV), and the ratio between current intensity back (*i*_b_) and forward (*i*_f_) for the redox processes exhibited at the Pt electrode by compounds 1, 2, and 3 (5 × 10^−4^ M) in dry acetonitrile with [^*n*^Bu_4_N][PF_6_] 10^−1^ M as supporting electrolyte.

b Measured with *ν* = 0.1 V s^−1^.

c Measured with *ν* = 0.02 V s^−1^.

The presence of an irreversible second reduction is still observed for 2 and 3. Overall, compared to 1, oxidation and reduction processes are poorly reversible with low ratios between current intensity backward and forward (0.5 < *i*_b_/*i*_f_ < 0.7).

**Fig. 4:**
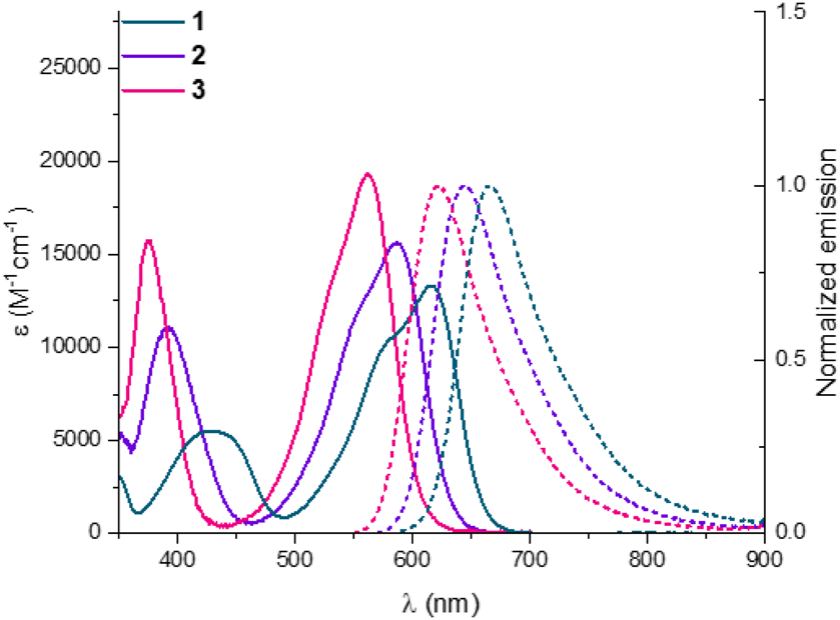
Absorption and normalized emission spectra of 1 (green), 2 (purple), and 3 (pink) in air-equilibrated MeCN at 20 °C with concentrations of 1 × 10^−5^ to 3 × 10^−5^ M.

Then, absorption and emission spectra of 2 and 3 were recorded in acetonitrile, and fluorescence quantum yields/lifetimes were determined (Fig. 4 and Table 2). 1,13-DMQA 1 displays a relatively broad absorption band with a maximum at 616 nm and an emission maximum at 666 nm, with a fluorescence quantum yield (*Φ*_f_) of 14% and a lifetime (*τ*) of 5.6 ns.^6*b*,21^ Upon rearrangement, tetrafluoroborate salts 2 and 3 present hypsochromic shifts of both absorption and emission (*λ*_max_ 616 → 587 → 562 nm and *λ*_em_ 666 → 642 → 622 nm for 1 → 2 → 3), which indicates a good correlation between optical and electrochemical energy gaps (Tables 1 and 2). Consistent with the energy gap law, the general blue-shift is also followed by an increase in *Φ*_f_ and *τ*. The experimental optical energy gap, absorption maximum and reduction potential of compounds 1–3 correlate well with the HOMO–LUMO gap, S_0_–S_1_ transition energy and LUMO energy, respectively, calculated with (TD-)DFT (Fig. S100). Overall, the hypsochromic shift is explained by a stronger electron-donating character of methoxy groups in *para* to the formal positive charge, allied with the gradual planarization of the helical skeleton, assuring a stronger influence of the *p*-OMe substituents and a progressive rise in LUMO energy (Fig. S101).

**Table 2:** Photophysical data of rearranged DMQA derivatives in acetonitrile

Molecules^a^	*λ*_max_ (nm)	*ε* (M^−1^ cm^−1^)	*λ*_em_ (nm)	Stokes shift (cm^−1^)	*Φ*_f_ b (%)	*τ* e (ns)	*k*_r_ f (10^6^ s^−1^)	*k*_nr_ g (10^6^ s^−1^)	Optical energy gap *E*_00_ (eV)
1	616	13 321	666	1219	14^c^	5.6	25	154	1.94
2	587	15 634	642	1460	24^d^	7.8	31	97	2.02
3	562	19 360	622	1717	43^d^	10.7	40	53	2.10

a Concentrations are 1 × 10^−5^ to 3 × 10^−5^ M.

b *Φ*_f_ estimated error = ±10%.

c Oxazine 170 perchlorate (*Φ* = 58% in EtOH).

d Cresyl violet perchlorate (*Φ* = 58% in EtOH).

e *λ*_exc_ = 400 nm.

f *k*_r_ = *Φ*_f_/*τ*.

g *k*_nr_ = (1 – *Φ*_f_)/*τ*.

### Subsequent reactivity and induced properties

**Fig. 5:**
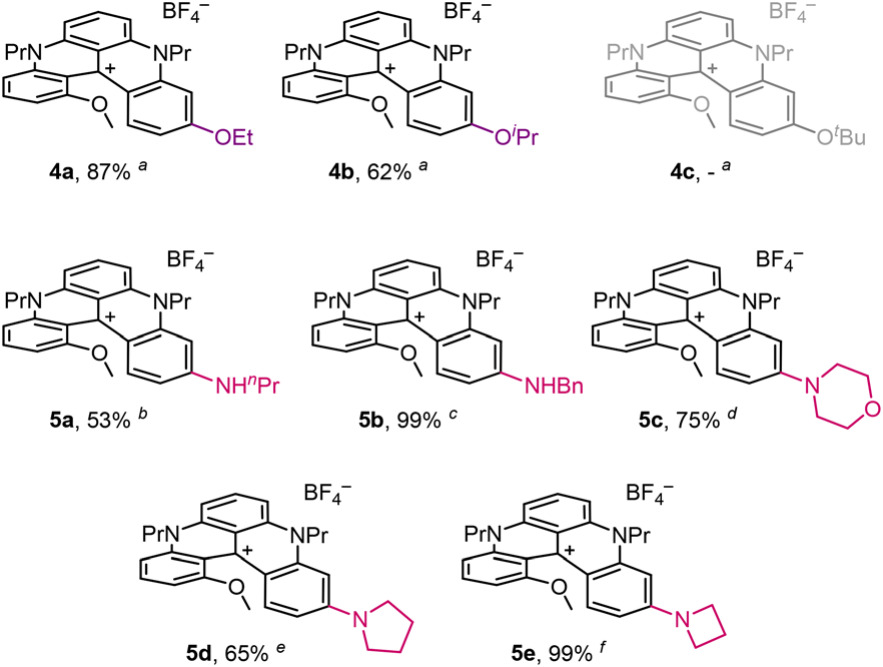
Synthesis of ethers 4 and anilines 5 starting from 2 as a substrate. ^*a*^(i) K_2_CO_3_, (2.0 equiv.) ROH, reflux, 24 h; (ii) air, white light, NaBF_4_ (aq.):DCM, 25 °C, 2 h. ^*b n*^PrNH_2_, 20 °C, 96 h. ^*c*^BnNH_2_, 40 °C, 48 h. ^*d*^Morpholine, 80 °C, 5 h. ^*e*^Pyrrolidine, 80 °C, 19 h. ^*f*^Azetidine, 20 °C, 72 h.

Then, with compound 2 readily accessible, late-stage functionalizations were tackled to expand possible derivatives of study. As expected from previous investigations, and that of Laursen *et al.* in particular,^4*a*,22^ regioselective substitutions of *p*-OMe substituents occurred in the presence of various alcohols and amines (Fig. 5). Nucleophilic aromatic substitutions (S_N_Ar) were prone to occur on the MeO group at position 11, by virtue of its less-hindered position, *para* to the formal positive charge. The other MeO group, inside the helical cove, was clearly less reactive. Faster kinetics and improved yields were obtained when nucleophiles were used directly as solvents. With ethanol and isopropanol as media, ethers 4a and 4b were generated in the presence of K_2_CO_3_ and at relatively high temperatures (reflux, 78 or 82 °C). In both instances, partial reduction of the carbocations occurred during reactions due to the hydride-donating ability of these solvents, with a predilection for isopropanol.^23,24^

An extra step of photooxidation (air, white-light irradiation, 25 °C) was then required to convert reduced neutral adducts into desired cations 4a (87%) and 4b (62%).^25^ With *tert*-butanol, formation of 4c could not be achieved due to the steric hindrance of the tertiary alcohol precluding the S_N_Ar reactivity.

With more nucleophilic primary amines as solvent, products 5a (53%) and 5b (99%) containing *n*-propyl and benzylamino residues were prepared at 20 or 40 °C, respectively. Higher temperatures (80 °C) were nevertheless necessary for the syntheses of morpholinyl 5c and pyrrolidinyl 5d derivatives in 75% and 65% yields, respectively. For the introduction of an azetidine moiety, lower temperature (20 °C) and longer reaction times were selected to yield corresponding derivative 5e in virtually quantitative yield (99%).

**Scheme 4:**
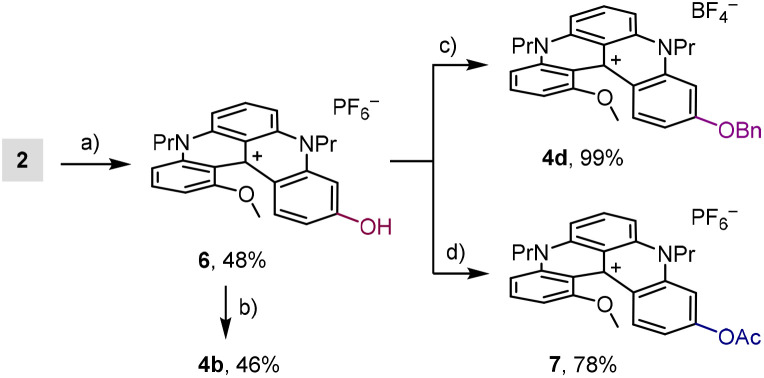
(a) (i). BBr_3_ (5.0 equiv.), CH_2_Cl_2_, 0 to 20 °C, 72 h; (ii). anion metathesis with KPF_6_ (aq.). (b) Cs_2_CO_3_ (2.5 equiv.), ^*i*^PrI (20 equiv.), DMF, 20 °C, 40 h. (c) (i). Cs_2_CO_3_ (2.5 equiv.), BnBr (20 equiv.), DMF, 20 °C, 2.5 h; (ii). anion metathesis with NaBF_4_ (aq.). (d) Et_3_N (2.0 equiv.), AcCl (3.0 equiv.), CH_2_Cl_2_, 20 °C, 15 min.

Alternatively, by treatment of 2 with BBr_3_, regioselective demethylation of the more accessible *p*-OMe group gave access to phenol 6 (Scheme 4). Counterion exchange between BF_4_^−^ and PF_6_^−^ led to a more soluble salt in dichloromethane and an easier overall purification. With 6 in hand, alkylation reactions with isopropyl iodide and benzyl bromide afforded 4b (46%) and 4d (99%) in moderate and excellent yields, respectively. Finally, acetylation of 6 gave rapid access to ester 7.

### Photophysical properties

**Fig. 6:**
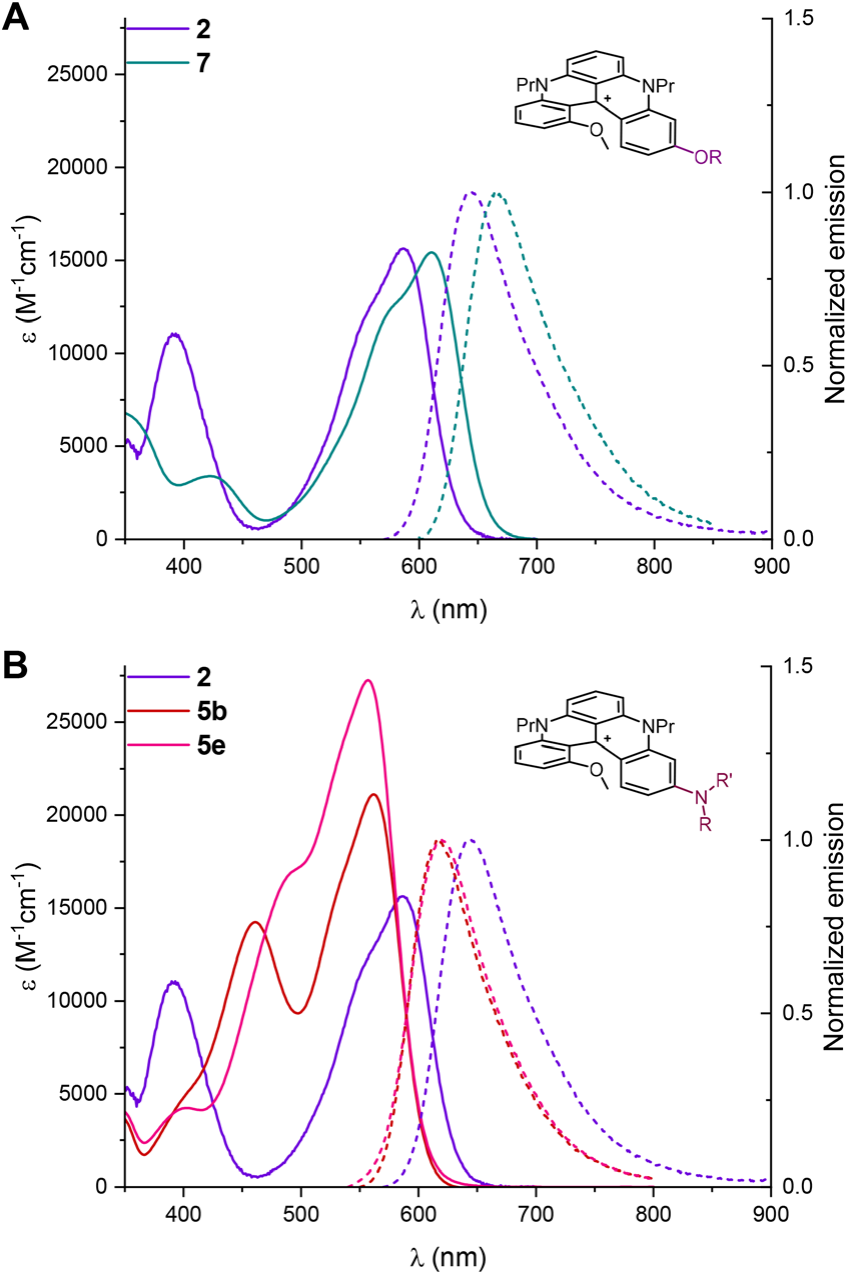
Absorption and normalized emission spectra in air-equilibrated MeCN at 20 °C with concentrations of 1 × 10^−5^ to 3 × 10^−5^ M. (A) 2 (purple) and 7 (green); (B) 2 (purple), 5b (red), and 5e (pink).

**Fig. 7:**
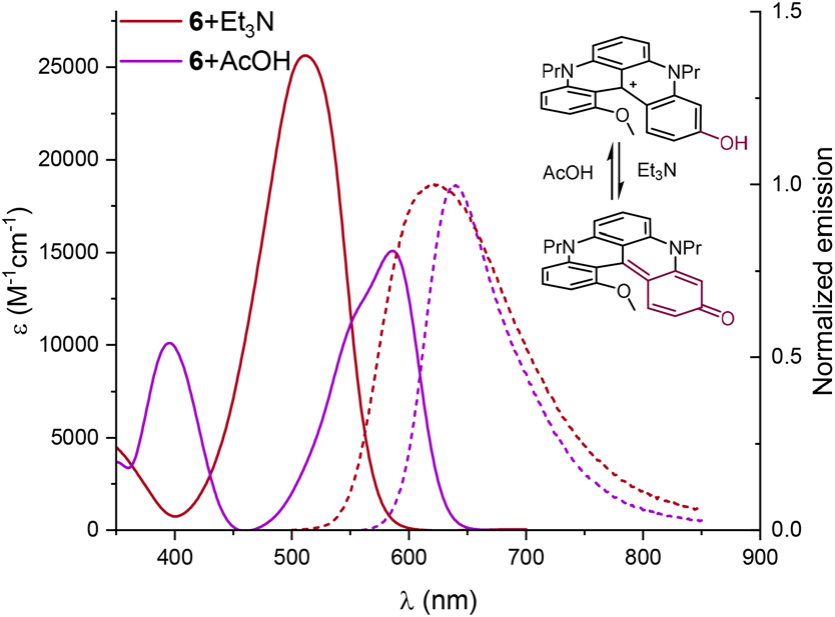
Absorption and normalized emission spectra of 6 in presence of an excess of either Et_3_N (red) or AcOH (purple) in air-equilibrated MeCN at 20 °C with concentrations of 1 × 10^−5^ to 3 × 10^−5^ M.

With compounds 4 to 7 in hand, absorption and emission spectra were recorded in acetonitrile (Table 3 and Fig. S9–S14) with selected examples presented in Fig. 6 and 7. As expected, few differences were observed between 2, 4a and 4d upon the change of the ether terminal group (Me → Et → Bn, Fig. S9). With sterically more encumbered 4b (isopropyl), a hypochromic effect (*ε* 11 746 M^−1^ cm^−1^) was noticed that cannot be readily explained at this stage. For ester 7, allied with the electron-deficient nature of the acetyl group, a bathochromic shift was noticed in both absorption and emission (*λ*_max_ 611 nm and *λ*_em_ 664 nm).^6*b*^ As a consequence, a decrease of *Φ*_f_ (8%) and *τ* (1 ns) was also observed in comparison with 2 (Fig. 6A).

**Table 3:** Photophysical data of rearranged DMQA derivatives in acetonitrile

Molecules^a^	*λ*_max_ (nm)	*ε* (M^−1^ cm^−1^)	*λ*_em_ (nm)	Stokes shift (cm^−1^)	*Φ*_f_ b (%)	*τ* (ns)	*k*_r_ h (10^6^ s^−1^)	*k*_nr_ i (10^6^ s^−1^)	Optical energy gap *E*_00_ (eV)
OEt (4a)	586	17 389	642	1489	25^c^	8.5^e^	29	88	2.02
OiPr (4b)	585	11 746	643	1542	27^c^	8.4^f^	32	87	2.02
OBn (4d)	588	17 192	643	1455	24^c^	7.6^g^	32	100	2.02
OAc (7)	610	15 420	664	1306	8^c^	1.0^e^	80	920	1.95
NHnPr (5a)	557	20 431	614	1667	57^c^	12.7^f^	45	34	2.12
NHBn (5b)	562	21 128	617	1586	54^c^	13.0^e^	42	35	2.11
Morpholinyl (5c)	569	18 482	633	1777	38^c^	10.1^e^	38	61	2.07
Pyrrolidinyl (5d)	559	13 963	613	1576	46^c^	12.0^e^	38	45	2.12
Azetidinyl (5e)	557	27 253	620	1824	50^c^	12.1 g	41	41	2.11
OH (6) + Et_3_N j	511	25 618	625	3569	4^d^	1.0^e^	40	960	2.21
OH (6) + AcOH k	586	15 098	638	1391	29^c^	8.0^e^	36	89	2.03

a Concentrations are 1 × 10^−5^ to 3 × 10^−5^ M.

b *Φ*_f_ estimated error = ±10%.

c Cresyl violet perchlorate (*Φ* = 58% in EtOH).

d Rhodamine B (*Φ* = 70% in MeOH).

e *λ*_exc_ = 470 nm.

f *λ*_exc_ = 395 nm.

g *λ*_exc_ = 400 nm.

h *k*_r_ = *Φ*_f_/*τ*.

i *k*_nr_ = (1 − *Φ*_f_)/*τ*.

j 1% of Et_3_N was added to the solvent.

k 1% of AcOH was added to the solvent.

Regioselective substitution of the outer MeO group by amino groups afforded a global blue-shift of optical properties with (large) differences upon the moieties attached (Fig. 6B). Globally, higher values were obtained for the molar extinction coefficients (*ε*), *Φ*_f_, and *τ*, reaching numbers up to 21 128 M^−1^ cm^−1^, 54% and 13 ns for 5b (NHBn). Even stronger values were expected for azetidine 5e in view of literature precedents indicating a stronger donor character for such auxochromes.^26^ In effect, values increased to 27 253 M^−1^ cm^−1^ for *ε*, 50% for *Φ*_f_ and 12.1 ns for *τ*.

Finally and not surprisingly, phenol 6 proved to be pH-sensitive.^27^ It was briefly studied under acidic and basic conditions, *i.e.* in the presence of AcOH or Et_3_N in acetonitrile (Fig. 7). With AcOH, phenol 6 displayed properties similar to those of 2 while, with Et_3_N, large hypso- and hyperchromic shifts (*λ*_max_ 511 nm and *ε* 25 618 M^−1^ cm^−1^) were observed along with a broadening of absorption and emission bands.

For 6, a remarkably large Stokes shift (3569 cm^−1^), albeit with low *Φ*_f_ and *τ* values (4% and 1 ns), is recorded; all data are consistent with those of a neutral quinone form under basic (deprotonated) conditions.

### Configurational lability (dynamic chirality) and asymmetric induction

Finally, consistent with the X-ray structural analysis that demonstrated a largely helical skeleton for 2 (*vide supra*), further investigations proved the occurrence of chiral geometries for mono-rearranged products in solution and that of compounds 4 and 5 in particular. However, it became rapidly clear that such compounds lacked configurational stability. While room temperature ^1^H-NMR analyses of 4d (OBn) and 5b (NHBn) supported the existence of residual stereoisomerism, since benzylic protons presented anisochronous doublet signals, heating in either CDCl_3_ or CD_3_CN of compound 4d (Fig. S1) resulted in coalescence of these diastereotopic protons above 80 °C (Δ*δ* 0.03 pm at 20 °C). Line-shape analysis of the dynamic exchange was realized for the acetonitrile solution and afforded kinetic constants at different temperatures (Table S1). Using Arrhenius and Eyring kinetic analyses (Table S2), activation energy *E*_a_ (22.8 ± 0.7 kcal mol^−1^), pre-exponential factor A (2.5 × 10^15^ ± 8 × 10^13^), enthalpy of activation Δ*H*^‡^ (22.2 ± 0.7 kcal mol^−1^) and entropy of activation Δ*S*^‡^ (10.0 ± 0.3 cal K^−1^ mol^−1^) were determined. Assuming consistency between solid-state and solution geometries, compound 4d interconverts between enantiomeric *M* (left-) and *P* (right-handed) helical conformers with a barrier of Δ*G*^‡^ 19.2 ± 0.8 kcal mol^−1^ (80.4 kJ mol^−1^) at 298 K. The much lowered kinetic barrier, in comparison with 1 (Δ*G*^‡^_473_ 41.3 kcal mol^−1^),^4*d*^ is the direct consequence of the reduced repulsion between the terminal ends of the helix brought by the loss of one MeO group in the groove (cove) area.

For all compounds 2 and 4–7, enantiomerization barriers remain probably in the same range (19 ± 1 kcal mol^−1^) and, consequently, these moieties are chiral yet configurationally labile, with half-lives in the range of seconds at 20 °C. The *M* and *P* enantiomers interconvert thus freely in solution (dynamic chirality), and the possibility of shifting the 1 : 1 equilibrium toward one preferred configuration was then debatable.^28,29^ In fact, several approaches could be considered to induce diastereoselective interactions in relation to scaffolds 4 and 5. Two such strategies were tried successively and then combined.

**Scheme 5:**
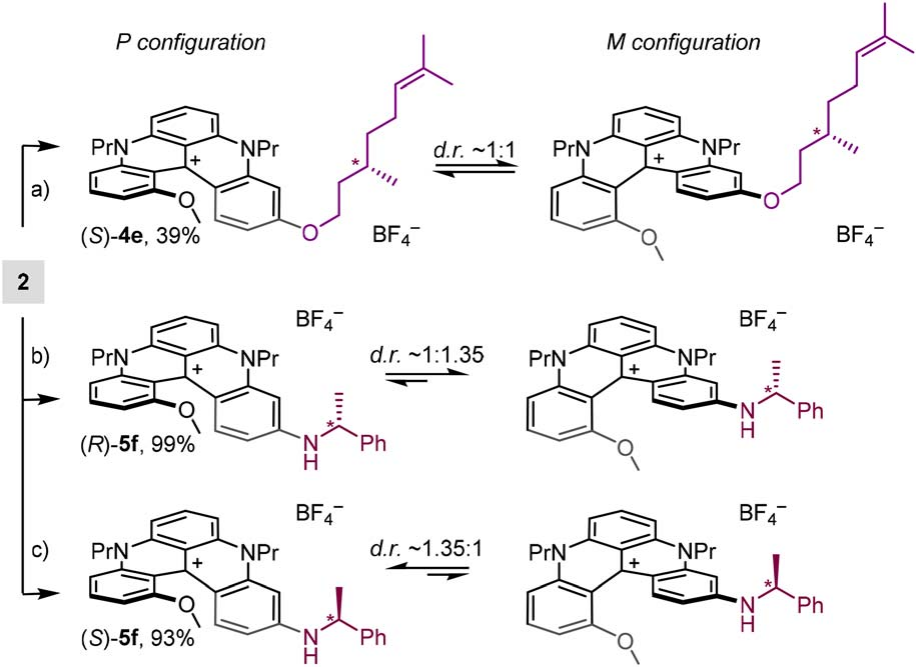
(a) (i) K_2_CO_3_, (−)-citronellol, 80 °C, 4.5 h; (ii) NaBF_4_ (aq.):DCM, 20 °C, air, white light. (b) (*R*)-PhEtNH_2_, 70 °C, 4–7 days. (c) (*R*)-PhEtNH_2_, 70 °C, 4–7 days.

**Fig. 8:**
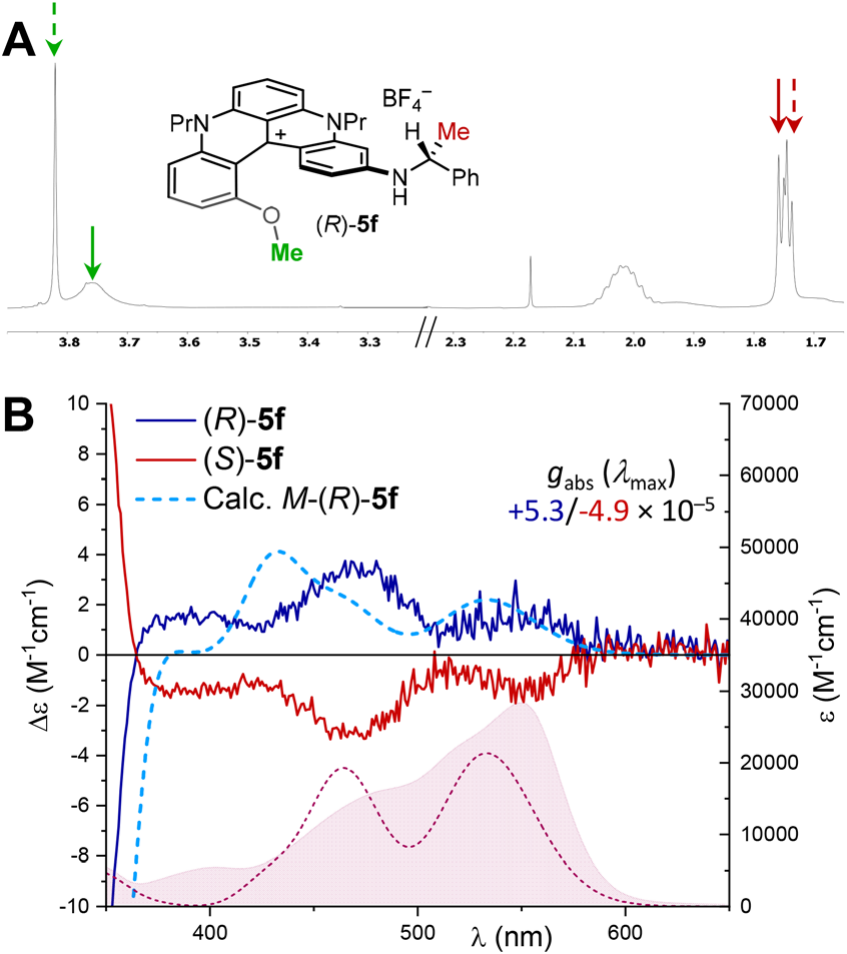
(A) ^1^H-NMR spectra in CDCl_3_ at 298 K, 500 MHz of [(*R*)-5f][BF_4_]; (B) ECD spectra and absorption spectra (underlying filled curve) in air-equilibrated chloroform at 20 °C of [(*S*)-5f][BF_4_] (red) and [(*R*)-5f][BF_4_] (blue), *c* 3–4 × 10^−5^ M. Dotted lines: calculated ECD and absorption spectra of (*M*)–(*R*)-5f at the B3LYP/def2-TZVP/PCM//B3LYP-D3BJ/6-31+G(d)/PCM level (IEF-PCM solvent model for CHCl_3_), plotted as sums of Gaussians with *σ* = 0.15 eV, red shifted by 25 nm, and scaled by a factor of 3.5.

In a first attempt, stereodefinite exocyclic substituents were introduced to impose, ideally, intramolecular discriminating interactions onto the chiral helical geometry. Practically, (*S*)-citronellol, (*S*)– and (*R*)-1-phenylethylamine were reacted with 2 under previously detailed conditions to generate products (*S*)-4e (39%), (*S*)-5f (93%) and (*R*)-5f (99%), in moderate to good yields (Scheme 5). While a minimal amount of discrimination was observed in ^1^H (and ^13^C) NMR spectra of (*S*)-4e (Fig. S45), a moderately better split of the signals was afforded for the interconverting diastereomers of (*S*)– or (*R*)-5f. In practice (Fig. 8A), doublet signals of the methyl group (red color) adjacent to the stereogenic center were separated but the signal separation was not large enough to permit a reliable integration. The NMR split was better for the internal MeO group (green color) yet with a strong broadening for one diastereomeric structure, also limiting the precision of the stereoselectivity measurement (*d. r.*∼1.35 : 1). The asymmetric induction was confirmed by ECD analyses. Whereas 3 Cotton effects were detected above 350 nm for amino (*S*)– and (*R*)-5f (Fig. 8B) and presented the expected mirror image relationship (red and blue traces), no induction could be observed for (*S*)-4e (Fig. S2). For the enantiomers of 5f, *g*_abs_ dissymmetry factor values were measured in the range of ±5 10^−5^ for the lowest energy band reflecting the small level of asymmetric induction observed in ^1^H-NMR spectroscopy.

To determine the absolute sense of asymmetric induction, the ECD spectra of (*R*)-5f were calculated using time-dependent density functional theory (TD-DFT) after DFT geometry optimizations. CAM-B3LYP and B3LYP were screened as functionals for excited-state calculations as they generally provide good accuracy for helicene-type compounds;^30^ in the current case, B3LYP yielded a better agreement. ECD spectra were calculated for (*M*)–(*R*)-5f and (*P*)–(*R*)-5f diastereomers and showed a quasi-mirror image relationship being dominated by the helicity of the diaza[4]helicene core (Fig. S102). (*M*)–(*R*)-5f had 3 positive ECD bands at above 350 nm, in accordance with the experimental spectrum of (*R*)-5f (Fig. 8B). Thus, (*R*)-1-phenylethylamine induces preferential (*M*)-helicity, as shown in Scheme 5. We notice that the calculated *g*_abs_ for the lowest-energy ECD band of 5f is 2.4 10^−4^, which fits well the experimental value of 5 10^−5^ considering a diastereomeric excess of 20% (0.2 × 2.4 10^−4^ = 4.8 10^−5^) and the fact that the two diastereomers have calculated mirror-image ECD spectra. Molecular orbital (MO) analysis revealed that the 3 ECD bands all involve the same LUMO, delocalized over the whole diaza[4]helicene core, but different occupied MOs with different degrees of delocalization: the first transition (HOMO–LUMO) is localized, while the following two have a pronounced charge-transfer (CT) character (Fig. S103).

**Fig. 9:**
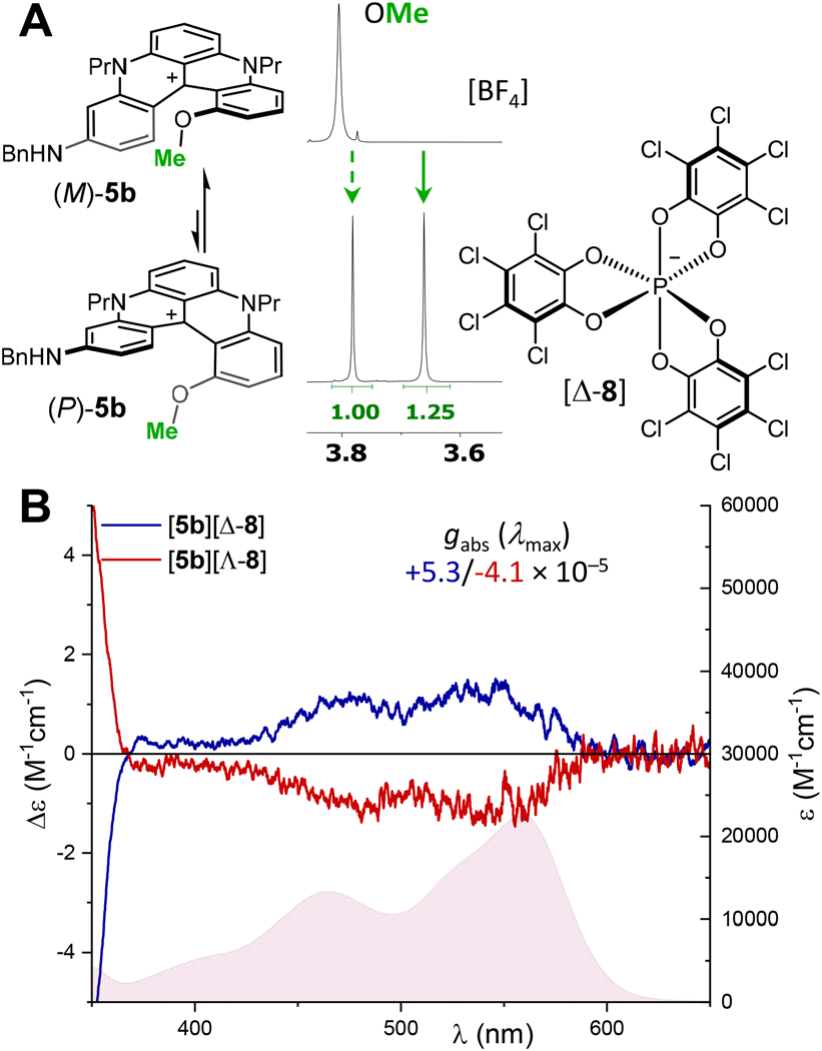
(A) ^1^H-NMR spectra (CDCl_3_, 298 K, 500 MHz) of [5b][BF_4_] and [5b][Δ-8] (Δ-enantiomer of TRISPHAT 8 is shown). (B) ECD spectra and absorption spectra (underlying filled curve) in air-equilibrated chloroform at 20 °C of [5b][Λ-8] (red) and [5b][Δ-8] (blue), *c* 3–4 × 10^−5^ M.

The second asymmetric induction strategy used a diastereoselective ion pairing approach with enantiopure hexacoordinated tris(tetrachlorobenzenediolato)phosphate anion 8, namely TRISPHAT, as a chiral auxiliary. This moiety 8, made in one step by the condensation of PCl_5_ and anhydrous tetrachlorocatechol, is resolved into single Δ and Λ enantiomers (right and left-handed three-bladed propellers) with cinchonidine as a resolving agent.^31^ This anion is an excellent NMR chiral solvating agent, able to distinguish enantiomers among many different types of chiral cationic reagents,^32^ but it is also an effective chiral auxiliary to control the configuration of labile moieties (Pfeiffer effect).^29*a*,*c*,33^ In practice, ion metatheses of BF_4_ to Δ- or Λ-8 anions were realized by mixing equimolar amounts of precursor salts, *e.g.* [5b][BF_4_] and [cinchonidinium][Δ-8] or [Bu_3_NH][Λ-8] followed by rapid chromatography over silica gel (eluent CH_2_Cl_2_). The desired ion pairs [5b][Δ-8] and [5b][Λ-8] were isolated as the only migrating fractions (>90% yield). In ^1^H-NMR spectra, the signals of (*M*)/(*P*)-5b were well distinguished in the presence of anions 8 and the MeO signals in particular (Δ*δ* 0.12 ppm, Fig. 9A). Clear integration of the signals revealed a small induction (*d. r.* 1.25 : 1), confirmed again by ECD measurements (Fig. 9B). In view of the previous configurational assignment by ECD analysis, and since ECD is dominated by the diaza[4]helicene core, the Δ and Λ enantiomers of anion 8 provoke an equilibrium shift toward (*M*)-5b and (*P*)-5b, respectively. Finally, as can be expected, choosing a polar solvent like CD_3_CN reduces the coulombic attraction within the ion pair and leads to a lack of NMR chiral solvating efficiency of the TRISPHAT anion (Fig. S93) and to an absence of diastereoselectivity in ECD spectroscopy (Fig. S3).

**Fig. 10:**
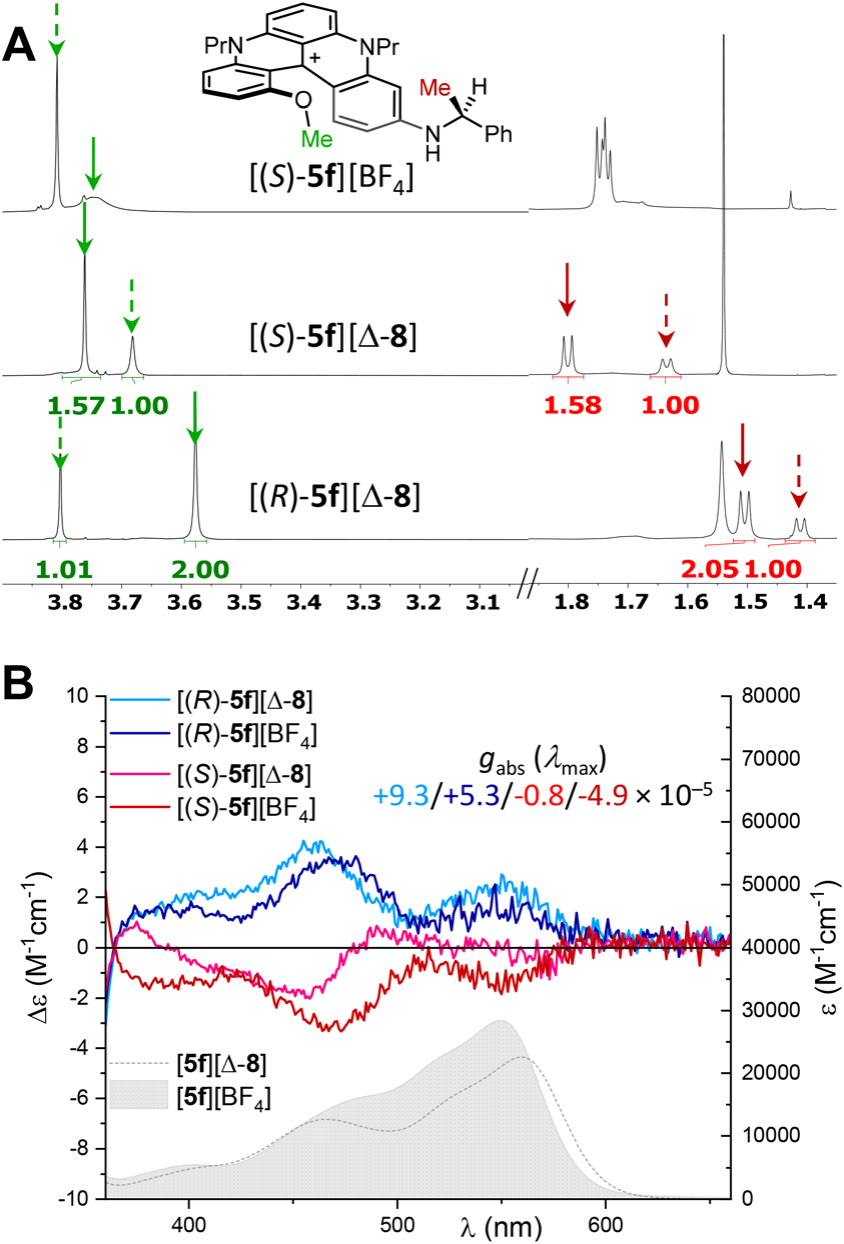
(A) ^1^H-NMR spectra (CDCl_3_, 298 K, 500 MHz) of [(*S*)-5f][BF_4_], [(*S*)-5f][Δ-8] and [(*R*)-5f][Δ-8] with *d. r*. 1.35 : 1, 1.6 : 1 and 2.0 : 1, respectively. (B) ECD spectra and absorption spectra (underlying filled curve, 5f corresponding salt) in air-equilibrated chloroform at 20 °C of [(*S*)-5f][BF_4_] (red), [(*R*)-5f][BF_4_] (blue), [(*S*)-5f][Δ-8] (pink), and [(*R*)-5f][Δ-8] (light blue).

Care was then taken to benefit from both the intramolecular and supramolecular (ionic) asymmetric induction strategies, trying to complement the influence of the stereogenic benzylamine substituent with the stereodefinite counterion. Diastereomeric salts [(*R*)-5f][Δ-8] and [(*S*)-5f][Δ-8] were prepared as above using the corresponding TRISPHAT source and their NMR spectra were compared with that of [5f][BF_4_] salts in CDCl_3_ (500 MHz, ^1^H-NMR, Fig. 10A). Not surprisingly, salts [(*R*)-5f][Δ-8] and [(*S*)-5f][Δ-8] presented well distinguished and dissimilar signals for the interconverting diastereomers in ^1^H-NMR, with a more pronounced nonequivalence of the MeO signals for ion pairs (*R*, Δ) over (*S*, Δ) (Δ*δ* 0.23 *vs.* 0.08 ppm, respectively). Integration of the signals revealed small but definite differences among the diastereomeric ratios (*d. r.* 2.0 : 1 *vs.* 1.6 : 1) for the two pairs.

A difference could also be monitored in ECD spectroscopy. In fact, while ECD spectra of [(*S*)-5f][BF_4_] and [(*R*)-5f][BF_4_] presented mirror-image curves (blue and red), the presence of anionic counterion Δ-TRISPHAT provoked non-reciprocal spectra for [(*S*)-5f][Δ-8] (pink) and [(*R*)-5f][Δ-8] (light blue). In practice, a direct quantitative relationship between the intensity of the Cotton effects and the diastereoselectivity measured in NMR could not be found. Nevertheless, the presence of both stereogenic elements can increase the *g*_abs_ value up to 9 10^−5^ for most favored [(*R*)-5f][Δ-8]; *g*_abs_ curves being displayed in Fig. S4.

## Conclusions

In summary, an acid-mediated skeletal rearrangement of cationic diaza[4]helicenes was developed to provide access to unprecedented regioisomeric 1,11- and 3,11-DMQA scaffolds, not readily available by direct functionalization. This transformation, followed by aerobic photooxidation, converts parent 1,13-substituted system 1 into new cationic 2 and 3 [4]helicenes, thus complementing existing LSF methods. The rearranged cores can be further diversified by selective S_N_Ar processes or by regioselective demethylation/refunctionalization, giving a broad family of unsymmetrical dyes 4–7. Structural changes have a clear impact on electronic and optical properties, with progressive blue shifts, improved fluorescence efficiencies in many cases, and small but definite substituent-dependent modulation across the visible range. X-ray and solution studies further show that peripheral editing of the helical framework reduces steric congestion in the cove region and strongly lowers the configurational barrier. As a result, the new helicenes are not configurationally stable but dynamically chiral, with enantiomerization barriers of around 19 kcal mol^−1^. This feature makes them responsive to asymmetric environments, as shown by modest yet measurable inductions using chiral appendages and/or enantiopure TRISPHAT counterions. Overall, the present work demonstrates that skeletal rearrangement emerges as a powerful complement to late-stage functionalization for controlling the structure, photophysics, and chiral dynamics of cationic [4]helicenes.

## Author contributions

Investigation: AG, RD, CH, CB (crystallography), GP (computation); funding acquisition and supervision: JL; writing – original draft: AG, CB, GP, JL; writing – review & editing: all.

## Conflicts of interest

There are no conflicts to declare

## Supplementary Material

SC-017-D6SC03190K-s001

SC-017-D6SC03190K-s002

## Data Availability

CCDC 2542615, 2542616, 2319499 and 2319500 contain the supplementary crystallographic data for this paper.^34*a*,*b*,*c*,*d*^ The data that support the findings of this study have been uploaded and will be openly available in yareta.unige.ch at https://doi.org/10.26037/yareta:dthr7jssmrcndfkvk6bjc7t4ta upon acceptance of the manuscript. It will be preserved for 10 years. Supplementary information (SI) is available. See DOI: https://doi.org/10.1039/d6sc03190k.
