# Supplementary material for: Skeletal rearrangement of [4]helicenes under acidic conditions: dynamic chirality and improved properties by subsequent peripheral editing
Source: Chem Sci. 2026 May 13;17(21):10422–33. doi: 10.1039/d6sc03190k (PMC13196605; doi:10.1039/d6sc03190k)
Supplement: SC-017-D6SC03190K-s001 [file SC-017-D6SC03190K-s001.pdf]

Supplementary Information (SI) for Chemical Science.
This journal is © The Royal Society of Chemistry 2026

Skeletal rearrangement of [4]helicenes under acid conditions; dynamic chirality and improved properties by subsequent peripheral editing

Arthur Gaucherand,[a] Romain Duwald,[a] Christelle Herse,[a] Céline Besnard,[b] Gennaro Pescitelli[c] and Jérôme Lacour[a]*

[a] Arthur Gaucherand, Romain Duwald, Christelle Herse, Jérôme Lacour – Department of Organic Chemistry, University of Geneva, Quai Ernest Ansermet 30, 1211 Geneva 4 (Switzerland) E-mail: jerome.lacour@unige.ch

[b] Céline Besnard – Laboratory of crystallography, University of Geneva, Quai Ernest Ansermet 30, 1211 Geneva 4 (Switzerland)

[c] Gennaro Pescitelli – Department of Chemistry and Industrial Chemistry, University of Pisa, Via Moruzzi 13, 56124 Pisa (Italy)

Supporting Information

Contents

1. General remarks and analysis conditions S3
2. Synthesis and characterization of new compounds S5
   2.1. Mono-skeletal rearrangement S5
   2.2. Double skeletal rearrangement S7
   2.3. Late-stage functionalization S9
   2.4. Ion exchange metathesis with TRISPHAT S17
3. Dynamic chirality S20
   3.1. VT-NMR and Line-shape analysis S20
   3.2. Electronic Circular Dichroism (ECD) S22
4. Structural Crystallographic Analysis S24
   4.1. Comparative analysis S24
   4.2. Structure CCDC 2542615 .... S25
   4.3. Structure CCDC 2542616 .... S27
5. Electrochemical properties S29
6. Photophysical properties S30
7. NMR & IR spectra; MRMS data S34
8. Computational Section S85
9. Cartesian coordinates S89
10. References S96

1. General remarks and analysis conditions

Dataset

The dataset for this article can be found at the following DOI: 10.26037/yareta:dthr7jssmrcndfkvk6bjc7t4ta. It will be preserved for 10 years.

Reagents and solvents: Unless otherwise stated, reagents were purchased from commercial sources and used without further purification. All reactions involving air-sensitive compounds were carried out under N_2 *via* an inert gas/vacuum double manifold line and standard Schlenk techniques using dry solvents. Reactions involving oxygen-sensitive reagents were performed using degassed solvents. Tetrahydrofuran (THF) was distilled under N_2 atmosphere over sodium and benzophenone.

Chromatography: **Analytical thin-layer chromatography** (TLC) and **retardation factors** (R_f) were performed with Silica gel 60 F_{254} aluminium plates purchased from Merck. **Column chromatography** was performed with Silica gel Supelco® 60 Å, 63-200 μm (70-230 mesh).

Nuclear Magnetic Resonance: NMR spectra were recorded on a Bruker Avance III 500 MHz, Bruker Avance III HD-*NanoBay* 400 MHz and Bruker Avance III HD-*NanoBay* 300 MHz spectrometers at room temperature. **^{1}H NMR** chemical shifts are given in ppm relative to Me_4Si with solvent resonances used as internal standards ($CDCl_3$ δ = 7.26 ppm, CD_2Cl_2 δ = 5.32 ppm). Data are reported as follows: chemical shift (ppm) on the *δ* scale, multiplicity, coupling constant (Hz) and integration. **^{13}C NMR** chemicals shifts are given in ppm relative to Me_4Si with solvent resonances used as internal standards ($CDCl_3$ δ = 77.16 ppm, CD_2Cl_2 δ = 53.84 ppm). **^{19}F NMR** chemical shifts are given in ppm. **Line-shape fitting analysis** was performed using Bruker TopSpin 5 software.

Infrared Spectroscopy: IR spectra were recorded with a Perkin-Elmer 100 FT-IR spectrometer using a diamond ATR Golden Gate sampling and are reported in wavenumbers (cm^{-1}).

Melting points were measured on a standard melting point apparatus in open capillary vials and are uncorrected.

Mass Spectrometry: LRMS spectra were obtained in methanol solutions on an API 150EX (AB/MDS Sciex) spectrometer in positive polarity. **HRMS** spectra were obtained in methanol solutions on a Waters Xevo G2 Tof (TOF) spectrometer in positive polarity by the Department of Mass Spectroscopy at the University of Geneva.

(Chir)Optical properties: all (chir)optical measurements were performed in 1 cm optical path quartz cells, unless otherwise stated. **UV-Vis-NIR absorption spectra** were recorded on a JASCO V-650 spectrophotometer at room temperature. Measurements were performed in air-equilibrated analytical grade acetonitrile at concentrations *ca*. 10^{-5} M. **Electronic Circular Dichroism (ECD)** spectra were recorded on JASCO J-815 or JASCO J-1500 spectrophotometers at room temperature with parameters as follows: scan speed – 200 nm/min, slit width – 1 nm, integration time – 1 sec, multiple accumulations. Measurements were performed in air-equilibrated analytical grade acetonitrile at concentrations *ca.* 1 or 5 $\cdot 10^{-5}$ M. All spectra were baseline corrected by subtraction of the solvent spectrum. **Steady-state fluorescence** spectra were measured using a FluoroMax+ spectrofluorometer from Horiba Scientific. All fluorescence spectra were corrected for the wavelength-dependent sensitivity of the detection. Fluorescence quantum yields φ_f were determined by comparison with a standard of known quantum yield using the following equation:

$$\Phi=\Phi_r \frac{I\, A_r\, n^2}{I_r\, A\, n_r^2}$$

where *A* is the absorbance at the excitation wavelength (λ), *n* the refractive index and *I* the integrated emission intensity; "r" stands for reference. Diluted solutions with absorption lower than 0.1 were employed. Excitations of reference and sample compounds were performed at the same wavelength. **Fluorescence lifetimes** on the nanosecond timescale were measured by a time-correlated single photon counting (TCSPC) setup. Excitation was performed at 395 nm (for **4b**, **5a**), 400 nm (for **2**, **3**), and 470 nm (for the other compounds), using ~60 ps

pulse at 20 MHz produced by a laser diode (PicoQuant, LDH-P-C-400). The fluorescence decay was followed at 640 nm (for **2**, **3**, **6+Et$_3$N**), 690 nm (for **4b**, **5a**), 694 nm (for **4a**, **4e**, **5b**, **5f**, **7**, **6+AcOH**), and 716 nm (for **5c**, **5d**), using band-pass filters. The full width at half-maximum (fwhm) of the instrument response function (IRF) was around 200 ps. The fluorescence time profiles were analysed with the deconvolution of the experimental IRF and an exponential function.

2. Synthesis and characterization of new compounds

[1,13-DMQA][BF4] **1** was synthesized according to reported procedures.[1]

2.1. Mono-skeletal rearrangement

1,13-dimethoxy-5,9-dipropyl-9,13b-dihydro-5H-quinolino[2,3,4-kl]acridine (**1-H**)

In a conical flask, $NaBH_4$ (76 mg, 2 mmol, 4 equiv.) was added to a solution of **1** (250 mg, 0.5 mmol, 1 equiv) in MeOH (10 mL; or absolute EtOH alternatively) and the mixture was manually stirred for 5 min at rt until color disappearance. In the dark, the reaction was quenched with water and the aqueous phase was extracted thrice with Et_2O. The combined organic layers were dried over Na_2SO_4, filtered, and concentrated in vacuo. The crude product was purified by column chromatography (SiO_2, liquid deposit, Et_2O/ Pentane = 10:90) to yield photosensitive white solid **1-H** (199 mg, 0.48 mmol, 96%). Spectroscopic data are in accordance with those reported.[2]

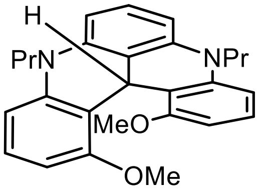

Molecular Weight: 414.5490

R_f = 0.23 (Et_2O/ Pentane = 1:9), **^{1}H NMR (300 MHz, $CDCl_3$)** δ 7.2 (t, *J* = 8.3 Hz, 1H), 7.0 (tdd, *J* = 8.1, 6.1, 0.8 Hz, 2H), 6.7 (d, *J* = 8.1 Hz, 1H), 6.6 – 6.4 (m, 3H), 6.4 (dd, *J* = 8.2, 4.4 Hz, 2H), 4.7 (s, 1H), 3.9 (td, *J* = 7.2, 4.1 Hz, 2H), 3.7 (s, 3H), 3.8 – 3.5 (m, 1H), 3.4 (s, 3H), 2.0 – 1.8 (m, 2H), 1.8 – 1.7 (m, 2H), 1.0 (t, *J* = 7.4 Hz, 3H), 1.0 (t, *J* = 7.4 Hz, 3H).

1,11-dimethoxy-5,9-dipropyl-5,9-dihydro-13bH-quinolino[2,3,4-kl]acridin-13b-ylium tetrafluoroborate salt (**2**)

General equation:

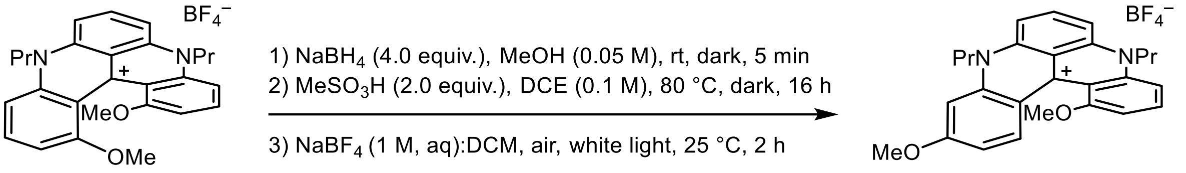

1 **2**

One-pot three-step process. 1) In a conical flask, $NaBH_4$ (63 mg, 1.65 mmol, 4 equiv.) was added to a solution of **1** (206 mg, 0.41 mmol, 1 equiv) in MeOH (8 mL; absolute EtOH, alternatively) and the mixture was manually stirred for 5 min at rt until disappearance of colour. In the dark, the reaction was quenched with water and the aqueous phase was extracted thrice with Et_2O. The combined organic layers were dried over Na_2SO_4, filtered, and concentrated in vacuo. St

2) Still in the dark, the crude white solid (mainly **1-H**) was dissolved in DCE (4 mL) under inert atmosphere (N_2 or Ar). After addition of $MeSO_3H$ (53 µL, 0.82 mmol, 2 equiv.), the mixture was stirred at 80 °C for 16 h. The resulting mixture was partitioned between DCM and $NaBF_4$ (1 M, aq), and the organic layer was separated, dried over Na_2SO_4, filtered, and concentrated in vacuo. The crude product was reduced again with $NaBH_4$ in the dark (similar conditions as for **1-H**) to work only on reduced products, and purified by column chromatography to yield **2-H** as a white solid(multiple times if necessary, SiO_2, dark, liquid deposit, Et_2O/ Pentane = 2:98 to 10:90). Due to the sensitivity of **2-H**, it was usually used directly in the next step.

3) Neutral product **2-H** was stirred in the presence of DCM and $NaBF_4$ (1 M, aq) under white light irradiation for 2 h. After partition, the organic layer was dried over Na_2SO_4, filtered, concentrated in vacuo, and precipitated with a centrifuge in Et_2O (twice) and pentane to yield purple solid **2** (86 mg, 0.89 mmol, 42%).

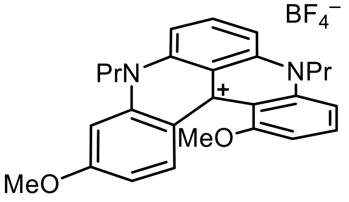

Molecular Weight: 500.3446

R*f* = 0.4 (MeOH/ DCM = 1:9), **^{1}H NMR (500 MHz, $CDCl_3$)** δ 8.2 (t, *J* = 8.5 Hz, 1H), 7.9 (t, *J* = 8.5 Hz, 1H), 7.8 (dd, *J* = 9.1, 3.6 Hz, 1H), 7.5 (d, *J* = 8.5 Hz, 1H), 7.4 (d, *J* = 8.7 Hz, 1H), 7.3 (d, *J* = 9.0 Hz, 1H), 7.2 (s, 1H), 7.1 (d, *J* = 9.0 Hz, 1H), 7.0 (d, *J* = 8.0 Hz, 1H), 4.8 – 4.7 (m, 1H), 4.6 – 4.5 (m, 2H), 4.4 – 4.3 (m, 1H), 4.2 (s, 3H), 3.8 (s, 3H), 2.2 – 2.0 (m, 4H), 1.3 (t, *J* = 9.7 Hz, 3H), 1.2 (t, *J* = 9.5 Hz, 3H), **^{13}C NMR (126 MHz, $CDCl_3$)** δ 166.7, 158.7, 145.1, 143.5, 143.2, 139.3, 139.2, 137.1(8), 137.1(5), 136.7, 134.9, 118.0, 115.5, 114.4, 109.2, 107.8, 105.4, 104.9(1), 104.8(8), 96.8, 56.8, 55.4, 51.2, 51.1, 19.8(1), 19.7(6), 11.3, 11.2, **^{19}F NMR (282 MHz, $CDCl_3$)** δ -153.22, -153.27, **IR (neat, cm^{-1}):** ν = 2966.80, 2879.11, 1604.82, 1581.30, 1550.12, 1488.25, 1471.33, 1437.17, 1381.90, 1343.08, 1294.39, 1249.10, 1171.31, 1135.36, 1054.25, 993.88, 832.74, 803.12, 764.32, **MP** = 263 °C, **HRMS-ESI+ (m/z):** [M]+ calculated for $C_{27}H_{29}N_2O_2^+$ 413.2224, found 413.2238.

1,11-dimethoxy-5,9-dipropyl-9,13b-dihydro-5H-quinolino[2,3,4-kl]acridine (**2-H**)

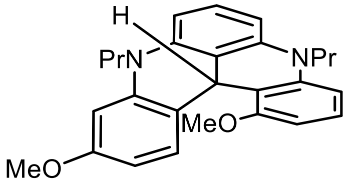

Molecular Weight: 414.5490

An analytical sample was prepared by $NaBH_3CN$ reduction of isolated cation **2.**

R*f* = 0.24 (Et_2O/ Pentane = 1:9), **^{1}H NMR (500 MHz, $CDCl_3$, $NaBH_3CN$ as additive)** δ 7.2 (t, *J* = 8.4 Hz, 1H), 7.0 (t, *J* = 8.1 Hz, 1H), 6.6 (d, *J* = 8.3 Hz, 1H), 6.6 (d, *J* = 2.4 Hz, 1H), 6.5 (m, 3H), 6.5 (dd, *J* = 8.4, 2.4 Hz, 1H), 6.4 (d, *J* = 8.2 Hz, 1H), 4.7 (s, 1H), 3.8 (t, *J* = 7.0 Hz, 2H), 3.8 (s, 3H), 3.7 (s, 3H), 3.7 – 3.5 (m, 2H), 2.0 – 1.9 (m, 2H), 1.8 – 1.7 (m, 2H), 1.0 (t, *J* = 7.4 Hz, 6H).**^{13}C NMR (126 MHz, $CDCl_3$, $NaBH_3CN$ as additive)** δ 158.9, 157.8, 145.5, 145.2, 142.3, 138.7, 128.6, 126.5, 125.3, 124.3, 111.6, 108.2, 105.9, 105.3, 105.1, 104.9, 102.0, 100.7, 55.6, 55.4, 48.1, 47.4, 33.5, 20.9, 19.7, 11.8, 11.2, **IR (neat, cm^{-1}):** ν = 2957.55, 2925.30, 2870.98, 2851.85, 1730.21, 1610.58, 1591.15, 1503.62, 1464.75, 1439.10, 1378.76, 1341.17, 1309.61, 1274.85, 1247.23, 1228.96, 1206.03, 1165.50, 1129.72, 1078.23, 1063.33, 1044.49, 984.76, 889.51, 838.55, 781.90, 753.56, 732.55, 675.56, 653.95, 586.44, **MP** = degradation, **HRMS-ESI+ (m/z):** [M+H]+ calculated for $C_{27}H_{30}N_2O_2^+$ 415.2381, found 415.2389.

2.2. Double skeletal rearrangement

General equation:

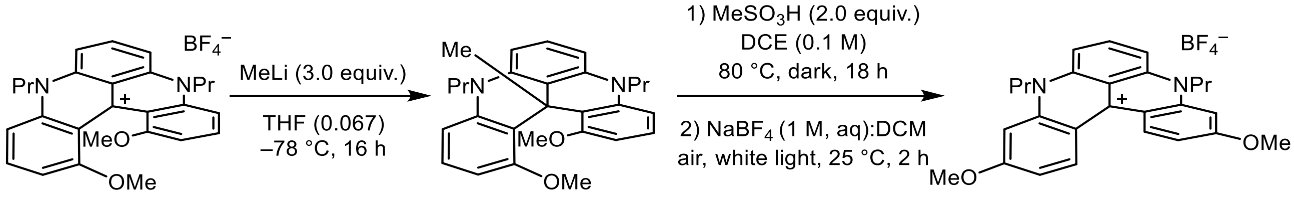

1,13-dimethoxy-13b-methyl-5,9-dipropyl-9,13b-dihydro-5H-quinolino[2,3,4-kl]acridine (**1-Me**)

In a dry 25 mL round-bottom flask, **1** (500 mg, 1.0 mmol, 1 equiv.) was dissolved in freshly distilled THF (15 mL), and MeLi (1.6 M in Et_2O, 1.875 ml, 3.0 mmol, 3 equiv.) was added dropwise at –78 °C. The reaction was stirred for 16 h at –78 °C, in the dark, and the crude medium was then quenched with water. Once at room temperature, still in the dark, the aqueous phase was extracted thrice with Et_2O. The combined organic layers were dried over Na_2SO_4, filtered, and concentrated in vacuo. The red crude product was purified by column chromatography (SiO_2, liquid deposit, Et_2O/ Pentane = 10:90) to yield a photosensitive pale yellow solid (111 mg, 0.26 mmol, 26%). The low yield is explained by the photochemical instability of the adduct. Also, only the ^1H NMR spectrum is affordable; reoxidation of **1** occurring very rapidly.

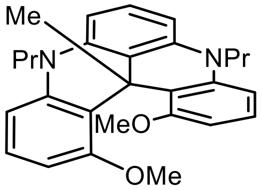

Molecular Weight: 428.5760

R*f* = 0.49 (Et_2O/ Pentane = 1:9), **^{1}H NMR (300 MHz, CDCl$_3$)** δ 7.17 (t, *J* = 8.3 Hz, 1H), 7.07 (t, *J* = 8.1Hz, 1H), 7.06 (t, *J* = 8.1Hz, 1H), 6.78 (dd, *J* = 8.2, 1.1 Hz, 1H), 6.60 (dd, *J* = 8.2, 0.9 Hz, 1H), 6.53 (dd, *J* = 8.6, 1.0 Hz, 1H), 6.50 – 6.42 (m, 3H), 3.96 (ddd, *J* = 9.2, 5.5, 1.6 Hz, 2H), 3.75 (s, 3H), 3.74 – 3.61 (m, 2H), 3.31 (s, 3H), 2.07 – 1.92 (m, 3H), 1.89 – 1.76 (m, 2H), 1.72 (s, 3H), 1.07 (t, *J* = 7.4 Hz, 6H), 1.05 (t, *J* = 7.4 Hz, 6H).

3,11-dimethoxy-5,9-dipropyl-5,9-dihydro-13bH-quinolino[2,3,4-kl]acridin-13b-ylium tetrafluoroborate salt (**3**)

Still in the dark, **1-Me** (43 mg, 0.1 mmol, 1 equiv.) was dissolved in DCE (1 mL) under inert atmosphere (N_2 or Ar). After addition of $MeSO_3H$ (13 µL, 0.2 mmol, 2 equiv.), the mixture was stirred at 80 °C for 18 h.

At 25 °C, the crude reaction mixture is added to DCM and $NaBF_4$ (1 M, aq) and stirred under white light irradiation for 2 h, and the organic layer was separated, dried over Na_2SO_4, filtered, and concentrated in vacuo. The crude product was purified by column chromatography (SiO_2, liquid deposit, MeOH/ DCM = 3:97 to 10:90), and precipitated with a centrifuge in Et_2O (twice) and pentane to yield red solid **3** (23 mg, 0.046 mmol, 46%).

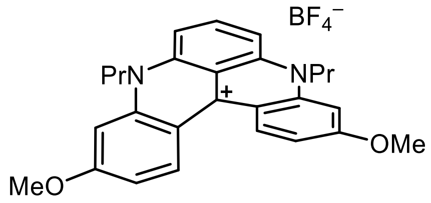

Molecular Weight: 500.3446

R_f = 0.37 (MeOH/ DCM = 1:9), **^{1}H NMR (500 MHz, CDCl$_3$)** δ 8.26 (d, *J* = 9.2 Hz, 2H), 8.13 (t, *J* = 8.5 Hz, 1H), 7.37 (d, *J* = 8.5 Hz, 2H), 7.21 (dd, *J* = 9.2, 2.2 Hz, 2H), 7.15 (d, *J* = 2.3 Hz, 2H), 4.51 (t, *J* = 8.3 Hz, 4H), 4.13 (s, 6H), 2.07 (h, *J* = 7.5 Hz, 4H), 1.24 (t, *J* = 7.4 Hz, 6H), **^{13}C NMR (126 MHz, CDCl$_3$)** δ 166.4, 145.9, 144.1, 139.6, 136.7, 133.6, 116.1, 114.8, 113.4, 105.4, 98.3, 56.7, 50.7, 19.5, 11.3, **^{19}F NMR (282 MHz, CDCl$_3$)** δ -152.46, -152.51, **IR (neat, cm^{-1}):** ν = 2926.68, 2879.72, 2852.36, 1604.79, 1578.43, 1544.59, 1510.26, 1494.66, 1470.58, 1436.85, 1379.37, 1345.74, 1332.38, 1284.10, 1248.34, 1224.55, 1205.89, 1176.33, 1138.83, 1092.09, 1054.53, 996.07, 914.09, 830.76, 794.72, 765.20, 729.50, **MP** = 197 °C, **HRMS-ESI+ (m/z):** [M]+ calculated for $C_{27}H_{29}N_2O_2^+$ 413.2224, found 413.2232.

2.3. Late-stage functionalization

<u>*S_NAr with alcohols **ROH***</u>

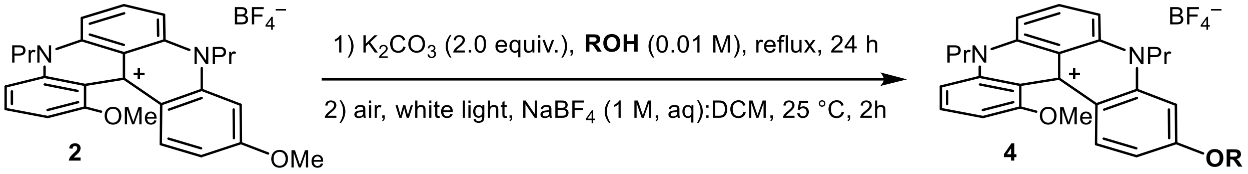

In a round-bottom flask, **2** (1 equiv.) and K_2CO_3 (2 equiv.) were combined and stirred in the presence of alcohol **ROH** (0.01 M) and the reaction was stirred under reflux for 24 hours. The crude was mixed with DCM and $NaBF_4$ (1 M, aq) and stirred under white-light irradiation for 2 h. Once partitioned and separated, the organic layer was dried over Na_2SO_4, filtered, and concentrated in vacuo. The crude products were purified by column chromatography (SiO_2, liquid deposit, MeOH/ DCM = 0:100 to 3:97), then precipitated with a centrifuge in Et_2O (twice) and pentane to yield purple solids.

11-ethoxy-1-methoxy-5,9-dipropyl-5,9-dihydro-13bH-quinolino[2,3,4-kl]acridin-13b-ylium tetrafluoroborate salt (**4a**)

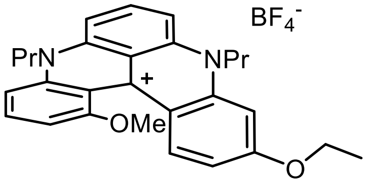

Molecular Weight: 514.3716

Prepared using **2** (10.0 mg, 0.02 mmol), K_2CO_3 (5.4 mg, 0.04 mmol) and EtOH (2 mL) at 78 °C yielding a purple solid (9.0 mg, 0.017 mmol, 87%).

R_f = 0.4 (MeOH/ DCM = 1:9), **^{1}H NMR (500 MHz, $CDCl_3$)** δ 8.16 (t, *J* = 8.5 Hz, 1H), 7.90 (t, *J* = 8.4 Hz, 1H), 7.75 (d, *J* = 9.3 Hz, 1H), 7.46 (d, *J* = 8.6 Hz, 1H), 7.36 (d, *J* = 9.1 Hz, 1H), 7.34 (d, *J* = 9.0 Hz, 1H), 7.13 (d, *J* = 2.2 Hz, 1H), 7.08 (dd, *J* = 9.4, 2.2 Hz, 1H), 6.97 (d, *J* = 8.1 Hz, 1H), 4.69 (ddd, *J* = 16.2, 10.4, 6.1 Hz, 1H), 4.59 – 4.48 (m, 2H), 4.45 – 4.36 (m, 2H), 4.30 (ddd, *J* = 15.8, 10.9, 5.6 Hz, 1H), 3.84 (s, 3H), 2.17 – 1.99 (m, 4H), 1.56 (t, *J* = 7.0 Hz, 3H), 1.26 (t, *J* = 7.3 Hz, 3H), 1.22 (t, *J* = 7.4 Hz, 3H),**^{13}C NMR (126 MHz, $CDCl_3$)** δ 166.1, 158.7, 145.0, 143.5, 143.2, 139.2(4), 139.2(0), 137.2, 136.7, 134.9, 117.9, 115.4, 114.5, 109.1, 107.8, 105.4, 104.9(2), 104.8(8), 97.2, 65.3, 55.4, 51.2, 51.1, 19.8(1), 19.7(5), 14.7, 11.3, 11.2, **^{19}F NMR (282 MHz, $CDCl_3$)** δ-153.43, -153.49, **IR (neat, cm^{-1}):** ν = 2969.70, 2937.92, 2879.71, 1602.74, 1580.84, 1549.28, 1528.14, 1488.86, 1468.51, 1445.85, 1383.40, 1342.48, 1295.98, 1247.16, 1216.46, 1171.04, 1135.68, 1050.98, 1035.48, 915.56, 840.00, 815.10, 802.76, 764.05, 730.82, 646.96, 623.69, 519.89, **MP** = 281 °C, **HRMS-ESI+ (m/z):** [M]+ calculated for $C_{28}H_{31}N_2O_2^+$ 427.2381, found 427.2402.

11-isopropoxy-1-methoxy-5,9-dipropyl-5,9-dihydro-13bH-quinolino[2,3,4-kl]acridin-13b-ylium tetrafluoroborate salt (**4b**)

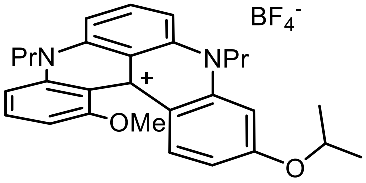

Molecular Weight: 528.3986

Prepared using **2** (5.0 mg, 0.01 mmol), K_2CO_3 (2.7 mg, 0.02 mmol), and ^{i}PrOH (1 mL) at 82 °C, yielding a purple solid (3.3 mg, 0.006 mmol, 62%).

R*f* = 0.34 (MeOH/ DCM = 1:9), **^{1}H NMR (500 MHz, $CDCl_3$)** δ 8.17 (t, *J* = 8.5 Hz, 1H), 7.90 (t, *J* = 8.5 Hz, 1H), 7.80 (d, *J* = 9.3 Hz, 1H), 7.48 (d, *J* = 8.6 Hz, 1H), 7.37 (d, *J* = 8.6 Hz, 1H), 7.35 (d, *J* = 8.7 Hz, 1H), 7.11 (dd, *J* = 9.2, 2.2 Hz, 1H), 7.09 (d, *J* = 2.2 Hz, 1H), 6.99 (d, *J* = 8.1 Hz, 1H), 5.00 (hept, *J* = 6.0 Hz, 1H), 4.65 (ddd, *J* = 16.1, 11.1, 5.7 Hz, 1H), 4.61 – 4.48 (m, 2H), 4.32 (ddd, *J* = 15.9, 11.0, 5.7 Hz, 1H), 3.87 (s, 3H), 2.22 – 2.01 (m, 4H), 1.51 (t, *J* = 5.9 Hz, 6H), 1.28 (t, *J* = 7.4 Hz, 3H), 1.23 (t, *J* = 7.4 Hz, 3H), **^{13}C NMR (126 MHz, $CDCl_3$)** δ 165.3, 158.7, 145.1, 143.5, 139.3(0), 139.2(6), 137.1, 136.8, 135.3, 117.9, 115.4, 114.7, 109.2, 107.8, 105.4, 105.0, 104.9, 98.3, 72.0, 55.4, 51.2, 51.1, 22.2, 22.0, 19.8, 11.3(4), 11.2(7), **^{19}F NMR (282 MHz, $CDCl_3$)** δ-153.72, -153.77, **IR (neat, cm^{-1}):** ν = 3642.03, 2972.30, 2936.64, 2879.62, 2209.75, 2024.81, 1981.44, 1947.92, 1733.75, 1604.02, 1581.89, 1548.06, 1526.72, 1487.25, 1468.60, 1385.20, 1343.04, 1295.06, 1248.71, 1218.68, 1201.78, 1170.94, 1135.94, 1102.75, 1054.79, 968.06, 929.14, 868.55, 835.38, 803.56, 765.08, 732.93, 646.67, 624.40, **MP** = 235 °C, **HRMS-ESI+ (m/z):** [M]+ calculated for $C_{29}H_{33}N_2O_2$+ 441.2537, found 441.2538.

(*S*)-11-((3,7-dimethyloct-6-en-1-yl)oxy)-1-methoxy-5,9-dipropyl-5,9-dihydro-13bH-quinolino[2,3,4-kl]acridin-13b-ylium tetrafluoroborate salt ((*S*)-**4e**)

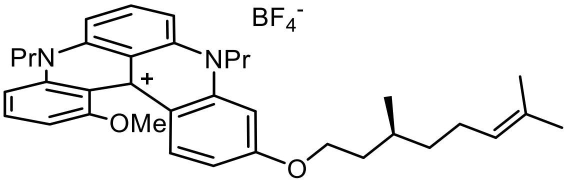

Molecular Weight: 624.5716

Prepared using **2** (5.0 mg, 0.01 mmol), K_2CO_3 (2.7 mg, 0.02 mmol), and (–)-citronellol (0.1 mL, 0.1 M, 55 equiv.) at 80 °C for 4.5 h, yielding a purple solid (2.4 mg, 0.004 mmol, 39%).

R*f* = 0.37 (MeOH/ DCM = 1:9), **^{1}H NMR (500 MHz, $CDCl_3$, two close diastereomers)** δ 8.19 (t, *J* = 8.5 Hz, 1H), 7.91 (t, *J* = 8.5 Hz, 1H), 7.79 (d, *J* = 9.8 Hz, 1H), 7.50 (d, *J* = 8.6 Hz, 1H), 7.38 (d, *J* = 8.5 Hz, 1H), 7.36 (d, *J* = 8.8 Hz, 1H), 7.12 – 7.05 (m, 2H), 6.99 (d, *J* = 8.1 Hz, 1H), 5.17 – 5.09 (m, 1H), 4.68 (ddd, *J* = 16.3, 10.9, 5.8 Hz, 1H), 4.61 – 4.50 (m, 2H), 4.41 – 4.27 (m, 3H), 3.86 (s, 1.5H, *dia1*), 3.85 (s, 1.5H, *dia2*), 2.22 – 1.93 (m, 6H), 1.84 – 1.71 (m, 2H), 1.69 (s, 3H), 1.63 (s, 3H), 1.50 – 1.40 (m, 1H), 1.36 – 1.20 (m, 7H), 1.03 (dd, *J* = 6.5, 3.1 Hz, 3H). **^{13}C NMR (126 MHz, $CDCl_3$, two close diastereomers)** δ 166.2, 158.7, 145.1, 143.5, 143.3, 139.3, 139.2, 137.2, 136.9, 135.1, 131.6, 124.7, 118.0, 115.5, 114.4, 114.3, 109.2, 107.8, 105.5, 105.0, 104.9, 97.2(5), 97.2(3), 67.9, 55.4, 51.2, 51.1, 37.3, 36.0, 29.85 29.6, 25.9(0), 25.8(8), 25.6, 19.8(4), 19.7(6), 19.6, 17.9, 11.4, 11.3. **^{19}F NMR (282 MHz, $CDCl_3$)** δ-153.61, -153.67 **IR (neat, cm^{-1}):** ν = 3558.93, 3112.50, 2961.53, 2926.09, 2878.83, 2854.03, 2495.67, 2156.17, 2032.90, 1971.38, 1956.82, 1738.96, 1603.16, 1580.57, 1549.23, 1528.30, 1509.77, 1488.24, 1468.45, 1445.92, 1380.21, 1342.84, 1294.10, 1247.07, 1217.16, 1201.15, 1171.09, 1135.08, 1051.99, 917.42, 898.03, 872.94, 831.77, 818.49, 803.56, 765.04, 733.77, 656.38, 624.87, 609.98, **MP** = 150 °C, **HRMS-ESI+ (m/z):** [M]+ calculated for $C_{36}H_{45}N_2O_2$+ 537.3476, found 537.3492.

S$_N$Ar with 1^{ry} and 2^{ry} amines

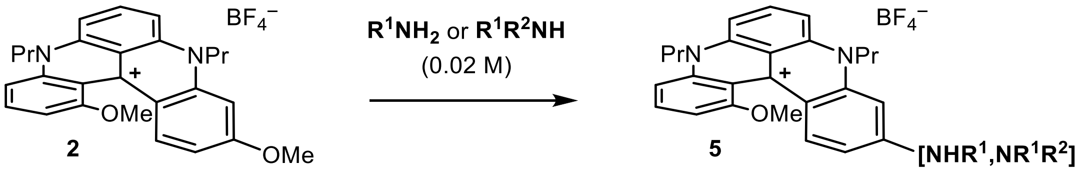

In a round-bottom flask, **2** (1 equiv.) was dissolved in a specific 1^{ry} or 2^{ry} amine (0.02 M) and the reaction was stirred for a given time and temperature. The crude was mixed with DCM and HBF_4 (1 M, aq), and the organic layer was washed with $NaBF_4$ (1 M, aq), dried over Na_2SO_4, filtered, and concentrated in vacuo. The crude

products wer purified by column chromatography (SiO_2, liquid deposit, MeOH/ DCM = 0:100 to 5:95), then precipitated with a centrifuge in Et_2O (twice) and pentane to yield red solids.

1-methoxy-5,9-dipropyl-11-(propylamino)-5,9-dihydro-13bH-quinolino[2,3,4-kl]acridin-13b-ylium tetrafluoroborate salt (**5a**)

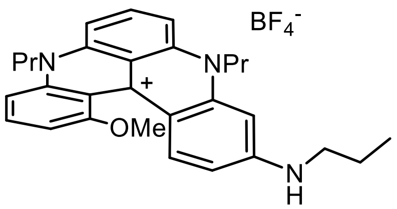

Molecular Weight: 527.4146

Prepared using **2** (4.5 mg, 0.009 mmol) and *n*-propylamine (0.5 mL) at 20 °C for 96 h, yielding a red solid (2.5 mg, 0.005 mmol, 53%).

R*f* = 0.49 (MeOH/ DCM = 1:9), **^{1}H NMR (500 MHz, $CDCl_3$)** δ 7.89 (t, *J* = 8.5 Hz, 1H), 7.72 (t, *J* = 8.4 Hz, 1H), 7.44 (d, *J* = 9.4 Hz, 1H), 7.23 (d, *J* = 8.5 Hz, 1H), 7.13 (d, *J* = 8.6 Hz, 1H), 7.09 (d, *J* = 8.4 Hz, 1H), 7.06 – 6.95 (m, 2H), 6.84 (d, *J* = 8.0 Hz, 1H), 4.57 – 4.44 (m, 1H), 4.41 – 4.27 (m, 2H), 4.13 (ddd, *J* = 15.8, 10.6, 5.9 Hz, 1H), 3.82 (s, 3H), 3.39 – 3.28 (m, 2H), 2.16 – 1.96 (m, 4H), 1.83 (h, *J* = 7.4 Hz, 2H), 1.26 (t, *J* = 7.4 Hz, 3H), 1.20 (t, *J* = 7.4 Hz, 3H), 1.07 (t, *J* = 7.4 Hz, 3H), **^{13}C NMR (126 MHz, $CDCl_3$)** δ 158.8, 156.7, 143.3, 141.9, 139.3, 139.2, 135.4, 134.5, 134.1, 116.5, 114.7, 108.6, 107.0, 104.9, 104.3, 103.9, 55.3, 50.6, 45.4, 22.2, 19.6, 19.2, 11.8, 11.3, **^{19}F NMR (282 MHz, $CDCl_3$)** δ-151.04, -151.10, **IR (neat, cm^{-1}):** ν = 3368.03, 2960.35, 2919.85, 2850.31, 1625.55, 1597.81, 1524.88, 1491.53, 1466.20, 1398.76, 1376.69, 1337.30, 1258.34, 1210.65, 1171.26, 1138.61, 1051.47, 798.57, 759.91, 731.50, 659.90, 622.08, **MP** = 133 °C, **HRMS-ESI+ (m/z):** [M]+ calculated for $C_{29}H_{34}N_3O^+$ 440.2697, found 440.2702.

11-(benzylamino)-1-methoxy-5,9-dipropyl-5,9-dihydro-13bH-quinolino[2,3,4-kl]acridin-13b-ylium tetrafluoroborate salt (**5b**)

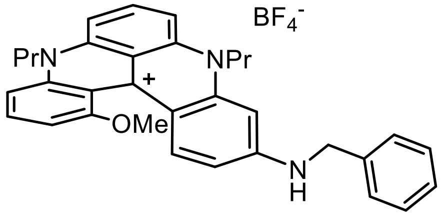

Molecular Weight: 575.4586

Prepared using **2** (6.0 mg, 0.012 mmol) and benzylamine (0.6 mL) at 40 °C for 24 h, yielding a red solid (6.9 mg, 0.009 mmol, 99%).

R*f* = 0.51 (MeOH/ DCM = 1:9), **^{1}H NMR (500 MHz, CD_2Cl_2)** δ 7.96 (t, *J* = 8.5 Hz, 1H), 7.84 – 7.76 (m, 1H), 7.54 (d, *J* = 9.4 Hz, 1H), 7.48 – 7.43 (m, 2H), 7.42 – 7.37 (m, 2H), 7.35 – 7.28 (m, 1H), 7.23 (t, *J* = 8.4 Hz, 2H), 7.19 (d, *J* = 8.5 Hz, 1H), 6.98 (dd, *J* = 9.3, 2.0 Hz, 1H), 6.91 (d, *J* = 8.1 Hz, 1H), 6.66 (t, *J* = 5.9 Hz, 1H), 6.39 (s, 1H), 4.75 – 4.60 (m, 2H), 4.42 (ddd, *J* = 15.3, 10.8, 6.1 Hz, 1H), 4.32 – 4.21 (m, 2H), 4.21 – 4.14 (m, 1H), 3.82 (s, 3H), 2.14 – 1.96 (m, 2H), 1.95 – 1.83 (m, 1H), 1.67 (s, 1H), 1.18 (t, *J* = 7.4 Hz, 3H), 1.10 (t, *J* = 7.4 Hz, 3H), **^{13}C NMR (126 MHz, CD_2Cl_2)** δ 159.2, 155.7, 144.4, 143.8, 143.5, 139.6(3), 139.5(8), 138.1, 136.3, 135.5, 134.5, 129.5(2), 129.5(0), 128.2, 127.6, 117.4, 115.3, 114.9, 109.1, 107.8, 105.2, 104.9, 104.8, 92.2, 55.7, 51.6, 51.2, 48.0, 20.1, 19.2, 11.5, 11.4, **^{19}F NMR (282 MHz, CD_2Cl_2)** δ-153.35, -153.40, **IR (neat, cm^{-1}):** ν = 3381.32, 3197.15, 2968.35, 2932.74, 2880.63, 2268.51, 2217.16, 2200.68, 2179.92, 2166.88, 2152.26, 2125.80, 2070.20, 2047.46, 2032.53, 2021.94, 1988.46, 1938.90, 1735.03, 1629.13, 1599.31, 1559.49, 1525.28, 1494.28, 1457.93, 1396.33, 1378.52, 1350.02,

1336.89, 1316.25, 1299.77, 1259.30, 1211.98, 1172.09, 1160.95, 1141.87, 1059.14, 914.36, 899.38, 860.55, 825.17, 810.15, **MP** = 172 °C, **HRMS-ESI+ (m/z):** [M]+ calculated for $C_{33}H_{34}N_3O+$ 488.2697, found 488.2680.

1-methoxy-11-morpholino-5,9-dipropyl-5,9-dihydro-13bH-quinolino[2,3,4-kl]acridin-13b-ylium tetrafluoroborate salt (**5c**)

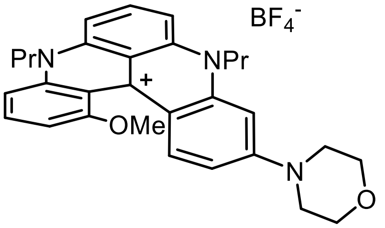

Molecular Weight: 555.4246

Prepared using **2** (5 mg, 0.01 mmol) and morpholine (0.5 mL) at 80 °C for 5 h, yielding a red solid (4.2 mg, 0.0075 mmol, 75%).

R*f* = 0.33 (MeOH/ DCM = 1:9), **^{1}H NMR (400 MHz, CDCl$_3$)** δ 8.00 (t, *J* = 8.5 Hz, 1H), 7.80 (t, *J* = 8.4 Hz, 1H), 7.65 (d, *J* = 9.5 Hz, 1H), 7.32 (d, *J* = 8.6 Hz, 1H), 7.25 – 7.15 (m, 3H), 6.92 (d, *J* = 8.1 Hz, 1H), 6.81 (d, *J* = 2.3 Hz, 1H), 4.70 – 4.58 (m, 1H), 4.52 – 4.38 (m, 2H), 4.19 (ddd, *J* = 18.4, 9.4, 4.5 Hz, 1H), 3.95 (t, *J* = 4.9 Hz, 4H), 3.86 (s, 3H), 3.68 (t, *J* = 5.0 Hz, 4H), 2.16 – 1.98 (m, 4H), 1.25 (t, *J* = 7.4 Hz, 3H), 1.21 (t, *J* = 7.4 Hz, 3H), **^{13}C NMR (126 MHz, CDCl$_3$)** δ 158.8, 156.1, 143.7, 143.3, 143.2, 139.4, 139.2, 136.3, 135.5, 134.6, 117.2, 114.7, 112.6, 108.8, 107.3, 105.2, 104.7, 104.3, 93.8, 66.6, 55.5, 50.9, 50.7, 47.0, 19.7, 19.5, 11.5, 11.3, **^{19}F NMR (282 MHz, CDCl$_3$)** δ-153.43, -153.49, **IR (neat, cm^{-1}):** ν = 2964.89, 2878.19, 1621.30, 1597.85, 1584.42, 1538.58, 1505.33, 1489.23, 1467.90, 1446.92, 1398.11, 1340.24, 1263.21, 1244.99, 1172.95, 1141.18, 1051.55, 1001.86, 916.90, 808.65, 760.39, 626.42, 520.19, **MP** = 170 °C, **HRMS-ESI+ (m/z):** [M]+ calculated for $C_{30}H_{34}N_3O_2+$ 468.2646, found 468.2667.

1-methoxy-5,9-dipropyl-11-(pyrrolidin-1-yl)-5,9-dihydro-13bH-quinolino[2,3,4-kl]acridin-13b-ylium tetrafluoroborate salt (**5d**)

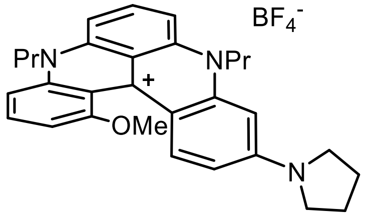

Molecular Weight: 539.4256

Prepared using **2** (10 mg, 0.02 mmol) and pyrrolidine (1 mL) at 80 °C for 19 h, yielding a red solid (7 mg, 0.0013 mmol, 65%).

R*f* = 0.31 (MeOH/ DCM = 1:9), **^{1}H NMR (500 MHz, CDCl$_3$)** δ 7.95 (t, *J* = 8.5 Hz, 1H), 7.77 (t, *J* = 8.4 Hz, 1H), 7.56 (d, *J* = 9.6 Hz, 1H), 7.28 (d, *J* = 8.6 Hz, 1H), 7.18 (d, *J* = 8.8 Hz, 1H), 7.15 (d, *J* = 8.4 Hz, 1H), 6.92 – 6.86 (m, 2H), 6.36 (d, *J* = 2.5 Hz, 1H), 4.55 – 4.45 (m, 1H), 4.45 – 4.34 (m, 2H), 4.16 (ddd, *J* = 15.8, 10.7, 5.9 Hz, 1H), 3.84 (s, 3H), 3.64 (broad s, 4H), 2.17 (broad s, 4H), 2.13 – 1.93 (m, 4H), 1.24 (t, *J* = 7.4 Hz, 3H), 1.19 (t, *J* = 7.4 Hz, 3H), **^{13}C NMR (126 MHz, CDCl$_3$)** δ 158.7, 153.3, 143.6, 143.3, 142.6, 139.3, 139.1, 135.9, 135.1, 134.7, 116.6, 114.1, 112.9, 108.6, 107.3, 105.0, 104.6, 104.3, 91.7, 55.4, 50.7, 48.6, 25.5, 19.7, 19.1, 11.5, 11.2, **^{19}F NMR (282 MHz, CDCl$_3$)** δ-153.90, -153.95, **IR (neat, cm^{-1}):** ν = 2967.06, 2877.07, 2261.77, 2002.91, 1623.50, 1594.91, 1533.90, 1505.39, 1483.39, 1458.12, 1403.24, 1375.30, 1338.62, 1303.68, 1266.98, 1242.62, 1213.07, 1164.56, 1140.51, 1051.62, 915.73, 861.34, 805.35, 759.02, 727.60, 647.04, 628.27, 519.93, **MP** = 146 °C, **HRMS-ESI+ (m/z):** [M]+ calculated for $C_{30}H_{34}N_3O_2+$ 452.2697, found 452.2689.

11-(azetidin-1-yl)-1-methoxy-5,9-dipropyl-5,9-dihydro-13bH-quinolino[2,3,4-kl]acridin-13b-ylium tetrafluoroborate salt (**5e**)

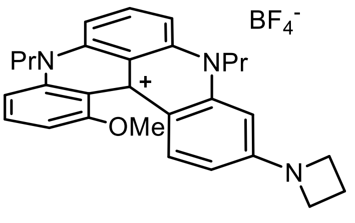

Molecular Weight: 525.3986

Prepared using **2** (5 mg, 0.01 mmol) and azetidine (67 μL, 57 mg, 1.0 mmol, 100 equiv.) at 20 °C for 72 h, yielding a red solid (5.2 mg, 0.005 mmol, 99%).

R_f = 0.31 (MeOH/ DCM = 1:9), **^{1}H NMR (500 MHz, $CDCl_3$)** δ 7.95 (t, *J* = 8.5 Hz, 1H), 7.77 (dd, *J* = 8.7, 8.1 Hz, 1H), 7.55 (d, *J* = 9.3 Hz, 1H), 7.29 (d, *J* = 8.6 Hz, 1H), 7.18 (d, *J* = 8.7 Hz, 1H), 7.16 (d, *J* = 8.4 Hz, 1H), 6.89 (d, *J* = 7.9 Hz, 1H), 6.63 (dd, *J* = 9.2, 2.0 Hz, 1H), 6.17 (d, *J* = 2.0 Hz, 1H), 4.56 – 4.46 (m, 1H), 4.45 – 4.37 (m, 2H), 4.34 (t, *J* = 7.5 Hz, 4H), 4.17 (ddd, *J* = 15.8, 9.9, 5.8 Hz, 1H), 3.84 (s, 3H), 2.59 (p, *J* = 7.5 Hz, 2H), 2.11 – 2.00 (m, 4H), 1.24 (t, *J* = 7.5 Hz, 3H), 1.20 (t, *J* = 7.4 Hz, 3H), **^{13}C NMR (126 MHz, $CDCl_3$)** δ 158.7, 155.3, 143.7, 143.3, 142.8, 139.3, 139.1, 135.8, 135.1, 134.7, 116.7, 114.3, 110.4, 108.7, 107.2, 105.1, 104.5, 104.3, 89.5, 55.4, 51.6, 50.7, 50.6, 19.7, 19.2, 16.2, 11.4, 11.3, **^{19}F NMR (282 MHz, $CDCl_3$)** δ-153.64, -153.69, **IR (neat, cm^{-1}):** ν = 2936.13, 2877.48, 1623.27, 1596.39, 1584.91, 1533.45, 1507.67, 1494.85, 1477.28, 1462.89, 1406.59, 1373.92, 1338.10, 1306.15, 1276.80, 1260.79, 1239.69, 1206.54, 1161.43, 1141.47, 1089.92, 1078.87, 1049.77, 1035.29, 966.04, 899.30, 824.37, 810.73, 795.29, 761.15, 730.92, **MP** = 231 °C, **HRMS-ESI+ (m/z):** [M]+ calculated for $C_{29}H_{32}N_3O^+$ 438.2540, found 438.2536.

(*R*)-1-methoxy-11-((1-phenylethyl)amino)-5,9-dipropyl-5,9-dihydro-13bH-quinolino[2,3,4-kl]acridin-13b-ylium tetrafluoroborate salt ((*R*)-**5f**)

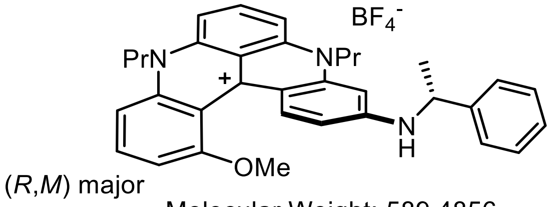

Molecular Weight: 589.4856

Prepared using **2** (4.9 mg, 0.01 mmol) and (*R*)-ethylphenylamine (0.5 mL) at 70 °C for 4 days, yielding a red solid (5.8 mg, 0.01 mmol, 99%).

R_f = 0.43 (MeOH/ DCM = 1:9), **^{1}H NMR (500 MHz, CD_2Cl_2, two diastereomers, some signals are two broad to be picked)** δ 7.86 (dt, *J* = 10.0, 8.5 Hz, 1H), 7.72 (td, *J* = 8.4, 3.1 Hz, 1H), 7.50 – 7.14 (m, 8H), 7.11 (dd, *J* = 8.7, 4.0 Hz, 2H), 7.07 (d, *J* = 8.4 Hz, 1H), 6.82 (dd, *J* = 8.2, 3.8 Hz, 1H), 4.73 – 4.62 (m, 1H), 4.40 – 4.30 (m, 1H), 4.28 – 4.02 (m, 2H), 3.82 (s, 1.3H), 3.76 (broad s, 1.7H), 2.10 – 1.96 (m, 2H), 1.96 – 1.64 (m, 1H), 1.75 (dd, *J* = 6.8, 4.2 Hz, 3H), 1.18 (td, *J* = 7.4, 1.5 Hz, 4H), 1.04 (broad s, 2H), **^{13}C NMR (126 MHz, CD_2Cl_2, two diastereomers)** δ 158.8, 155.5, 144.3, 144.2, 143.8, 143.2(4), 143.2(0), 142.2, 142.1, 139.2(1), 139.1(6), 139.1, 139.0, 135.6, 135.5, 134.6, 134.5, 133.6, 133.4, 129.1, 127.4, 127.3, 126.2, 125.9, 116.6, 116.5, 114.6(4), 114.5(7), 108.6, 108.4, 107.0, 104.7(1), 104.6(7), 104.4, 104.3, 103.9(4), 103.8(6), 55.4, 55.3, 54.6, 54.5, 51.1, 50.6(3), 50.5(8), 24.5, 24.4, 19.6, 19.0, 18.4, 11.4, 11.3, **^{19}F NMR (282 MHz, CD_2Cl_2)** δ-151.65, -151.71, **IR (neat, cm^{-1}):** ν = 3366.34, 2968.27, 2933.55, 2877.78, 1625.19, 1598.47, 1586.06, 1559.43, 1524.83, 1489.59, 1394.28, 1376.81, 1338.33, 1257.79, 1210.46, 1172.12, 1134.32, 1056.42, 914.13, 828.32, 810.90, 798.42, 760.28, 729.51, 704.02, 647.44, 626.50, **MP** = 152 °C, **HRMS-ESI+ (m/z):** [M]+ calculated for $C_{34}H_{36}N_3O^+$ 502.2853, found 502.2846.

(*S*)-1-methoxy-11-((1-phenylethyl)amino)-5,9-dipropyl-5,9-dihydro-13bH-quinolino[2,3,4-kl]acridin-13b-ylium tetrafluoroborate salt ((*S*)-**5f**)

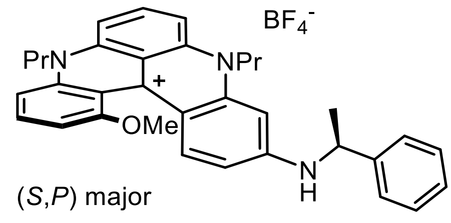

Molecular Weight: 589.4856

Prepared using **2** (4.8 mg, 0.01 mmol) and (*S*)-ethylphenylamine (0.5 mL) at 70 °C for 7 days, yielding a red solid (5.3 mg, 0.009 mmol, 93%).

All classical spectroscopic data are in accordance with those of (*R*)-**5f** above.

Regioselective mono-demethylation

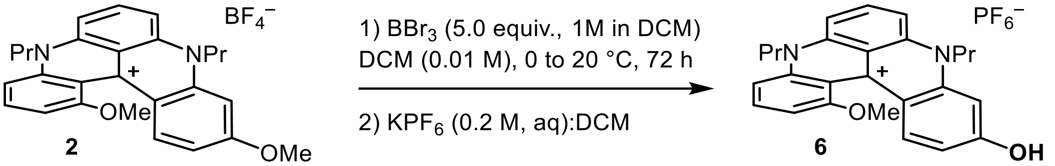

Molecular Weight: 544.4782

In a round-bottom flask, **2** (1 equiv., 15 mg, 0.03 mmol) was dissolved in DCM (3 mL, 0.01 M), and BBr_3 (1 M in DCM, 150 µl, 0.15 mmol, 5 equiv.) was slowly added at 0 °C, and the reaction was stirred for 72 h at 20 °C. The resulting mixture was partitioned between DCM and HPF_6 (0.1 M, aq), and the organic layer was washed with KPF_6 (0.2 M, aq), dried over Na_2SO_4, filtered, and concentrated in vacuo. The crude product was purified by column chromatography (SiO_2, liquid deposit, MeOH/ DCM = 0:100 to 5:95), then precipitated with a centrifuge in Et_2O (twice) and pentane to yield a red solid (7.8 mg, 0.014 mmol, 48%).

11-hydroxy-1-methoxy-5,9-dipropyl-5,9-dihydro-13bH-quinolino[2,3,4-kl]acridin-13b-ylium hexafluorophosphorate salt (**6**)

R_f = 0.29 (MeOH/ DCM = 1:9), **^{1}H NMR (400 MHz, CD_2Cl_2)** δ 8.07 (t, *J* = 8.5 Hz, 1H), 7.86 (t, *J* = 8.5 Hz, 1H), 7.73 (d, *J* = 9.2 Hz, 1H), 7.37 (d, *J* = 8.6 Hz, 1H), 7.30 (d, *J* = 4.7 Hz, 1H), 7.28 (d, *J* = 4.3 Hz, 1H), 7.25 (s, 1H), 7.12 (d, *J* = 9.2 Hz, 1H), 6.95 (d, *J* = 8.1 Hz, 1H), 4.63 – 4.45 (m, 2H), 4.44 – 4.34 (m, 1H), 4.26 (ddd, *J* = 15.8, 10.6, 5.9 Hz, 1H), 3.83 (s, 3H), 2.16 – 2.00 (m, 4H), 1.22 (t, *J* = 7.5 Hz, 3H), 1.21 (t, *J* = 7.4 Hz, 3H), **^{13}C NMR (126 MHz, $CDCl_3$, quinone form starts to appear in solution)** δ 158.9, 143.9, 143.3, 139.3(1), 139.2(8), 136.3, 135.7, 134.6, 128.9, 117.5, 117.2(1), 117.1(7), 115.1, 109.0, 10738, 105.1, 104.5, 104.1, 99.4, 55.3, 51.0, 29.9, 19.7, 19.5, 11.3, 11.0, **^{19}F NMR (282 MHz, $CDCl_3$)** δ-70.97, -70.50, **IR (neat, cm^{-1}):** ν = 3471.20, 2924.22, 2851.61, 1735.44, 1601.82, 1581.93, 1525.33, 1484.35, 1467.94, 1392.63, 1339.47, 1250.89, 1207.15, 1171.09, 1135.09, 1095.85, 1075.46, 1025.03, 836.47, 803.15, 762.01, 733.82, 657.00, 618.16, **MP** = 235 °C, **HRMS-ESI+ (m/z):** [M]+ calculated for $C_{26}H_{27}N_2O_2$+ 399.2067, found 399.2083.

O-alkylation

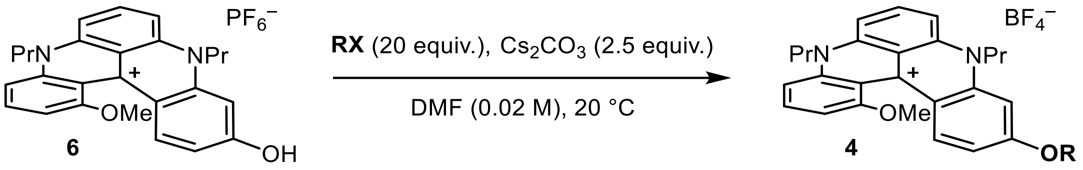

In a round-bottom flask, **6** (1 equiv.) and Cs_2CO_3 (2.5 equiv.) were combined in DMF (0.02 M) under N_2 atmosphere; halogenoalkane RX (20 equiv.) was added, and the reaction was stirred at 20 °C. The resulting mixture was partitioned between DCM and LiCl (10% aq), and the organic layer was washed with $CuSO_4$ (sat, aq), $NaBF_4$ (1 M, aq), dried over Na_2SO_4, filtered, and concentrated in vacuo. The crude product was purified by column chromatography (SiO_2, liquid deposit, MeOH/ DCM = 0:100 to 2:98), then precipitated with a centrifuge in Et_2O (twice) and pentane to yield a purple solid.

11-isopropoxy-1-methoxy-5,9-dipropyl-5,9-dihydro-13bH-quinolino[2,3,4-kl]acridin-13b-ylium tetrafluoroborate salt (**4b**)

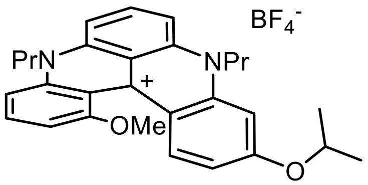

Molecular Weight: 528.3986

Prepared using **6** (3.4 mg, 0.006 mmol), Cs_2CO_3 (4.9 mg, 0.015 mmol), and ^{i}PrI (12 µL, 20.4 mg, 0.12 mmol) for 40 h, yielding a purple solid (1.5 mg, 0.003 mmol, 46%).

Spectroscopic data are in accordance with those of **4b** reported above.

11-(benzyloxy)-1-methoxy-5,9-dipropyl-5,9-dihydro-13bH-quinolino[2,3,4-kl]acridin-13b-ylium tetrafluoroborate salt (**4d**)

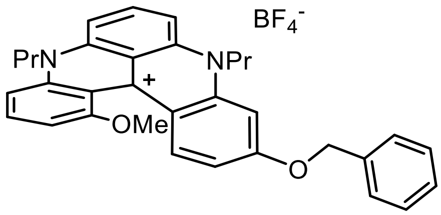

Molecular Weight: 576.4426

Prepared using **6** (4.3 mg, 0.008 mmol), Cs_2CO_3 (6.5 mg, 0.02 mmol), and BnBr (19 µL, 27.4 mg, 0.16 mmol.) for 2.5 h, yielding a purple solid (4.6 mg, 0.008 mmol, 99%).

R_f = 0.51 (MeOH/ DCM = 1:9), **^{1}H NMR (500 MHz, $CDCl_3$)** δ 8.15 (t, *J* = 8.5 Hz, 1H), 7.93 – 7.86 (m, 1H), 7.75 (d, *J* = 9.8 Hz, 1H), 7.57 – 7.51 (m, 2H), 7.47 – 7.30 (m, 6H), 7.17 (dt, *J* = 7.4, 2.2 Hz, 2H), 6.97 (d, *J* = 8.0 Hz, 1H), 5.46 (d, *J* = 2.0 Hz, 2H), 4.64 (ddd, *J* = 15.3, 11.4, 5.5 Hz, 1H), 4.54 (ddd, *J* = 15.2, 11.0, 5.9 Hz, 1H), 4.45 (ddd, *J* = 15.5, 11.3, 4.9 Hz, 1H), 4.30 (ddd, *J* = 15.9, 11.0, 5.7 Hz, 1H), 3.83 (s, 3H), 2.15 – 1.94 (m, 3H), 1.91 – 1.78 (m, 1H), 1.21 (t, *J* = 7.3 Hz, 3H), 1.20 (t, *J* = 7.2 Hz, 3H), **^{13}C NMR (126 MHz, $CDCl_3$)** δ 165.5, 158.7, 143.3, 143.2, 139.2(2), 139.1(9), 137.2, 136.8, 135.8, 134.9, 129.0, 128.6, 127.7, 118.0, 115.6, 114.9, 109.2, 107.8, 105.4, 104.9(3), 104.8(9), 98.1, 71.2, 55.4, 51.2, 19.8, 19.5, 11.3, 11.2, **^{19}F NMR (282 MHz, $CDCl_3$)** δ-153.12, -153.17, **IR (neat, cm^{-1}):** ν = 2964.79, 2933.50, 2879.13, 2160.49, 1603.27, 1580.21, 1550.07, 1528.13, 1487.30, 1468.29, 1444.04,

1380.79, 1342.53, 1291.94, 1246.42, 1212.23, 1170.95, 1135.43, 1052.60, 914.13, 830.20, 818.09, 802.85, 763.44, 730.39, **MP** = 168 °C, **HRMS-ESI+ (m/z):** [M]+ calculated for $C_{33}H_{33}N_2O_2$+ 489.2537, found 489.2541.

Acetylation

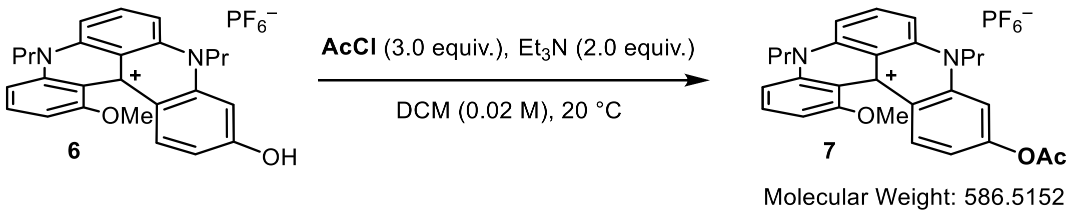

In a round-bottom flask, **6** (1 equiv., 10.4 mg, 0.019 mmol) was dissolved in DCM (0.02 M) under N_2 atmosphere, Et_3N (5.3 µL, 3.8 mg, 0.038 mmol, 2 equiv.) and AcCl (4 µL, 4.4 mg, 0.056 mmol, 3 equiv.) were added and the reaction was stirred at 20 °C for 15 min. The resulting mixture was partitioned between DCM and HBF_4 (1 M, aq), and the organic layer was washed with $NaBF_4$ (1 M, aq), dried over Na_2SO_4, filtered, and concentrated in vacuo. The crude product was purified by column chromatography (SiO_2, liquid deposit, MeOH/ DCM = 0:100 to 5:95), then precipitated with a centrifuge in Et_2O (twice) and pentane to yield a purple solid (8.7 mg, 0.015 mmol, 78%).

11-acetoxy-1-methoxy-5,9-dipropyl-5,9-dihydro-13bH-quinolino[2,3,4-kl]acridin-13b-ylium hexafluorophosphorate salt (**7**)

R*f* = 0.57 (MeOH/ DCM = 1:9), **^{1}H NMR (500 MHz, CD_2Cl_2)** 8.26 (t, *J* = 8.5 Hz, 1H), 7.99 (dd, *J* = 8.8, 8.2 Hz, 1H), 7.92 (d, *J* = 9.1 Hz, 1H), 7.62 (d, *J* = 2.1 Hz, 1H), 7.50 (d, *J* = 8.6 Hz, 1H), 7.48 (d, *J* = 8.5 Hz, 1H), 7.45 (d, *J* = 8.9 Hz, 1H), 7.28 (dd, *J* = 9.1, 2.0 Hz, 1H), 7.05 (d, *J* = 8.0 Hz, 1H), 4.69 – 4.54 (m, 2H), 4.52 – 4.36 (m, 2H), 3.86 (s, 3H), 2.43 (s, 3H), 2.26 – 2.04 (m, 4H), 1.27 (t, *J* = 7.5 Hz, 3H), 1.24 (t, *J* = 7.4 Hz, 3H), **^{13}C NMR (126 MHz, CD_2Cl_2)** δ 169.0, 158.9, 157.2, 146.2, 143.6, 142.3, 139.7, 139.6, 138.1, 137.7, 135.0, 119.3, 118.2, 110.2, 108.3, 108.0, 105.4(8), 105.4(5), 105.3(9), 55.7, 52.0, 51.8, 21.6, 20.2, 20.0, 11.3, 11.2, **^{19}F NMR (282 MHz, CD_2Cl_2)** δ-72.22, -74.73, **IR (neat, cm^{-1}):** ν = 3111.94, 2965.49, 2938.58, 2881.03, 1770.50, 1609.30, 1577.71, 1554.19, 1523.35, 1509.74, 1487.11, 1467.26, 1432.38, 1370.40, 1346.08, 1262.80, 1246.46, 1196.57, 1172.34, 1135.90, 1097.63, 1076.86, 1045.71, 1016.08, 956.03, 938.05, 909.31, 834.93, 806.60, 789.69, 764.04, 733.97, 709.36, 677.48, 656.60, 618.57, **MP** = 264 °C, **HRMS-ESI+ (m/z):** [M]+ calculated for $C_{28}H_{29}N_2O_3$+ 441.2173, found 441.2186.

2.4. Ion exchange metathesis with TRISPHAT

In a small vial, [BF_4] salt (1 equiv.) and [cinchonidinium][Δ-TRISPHAT] or [Bu_3NH][Λ-TRISPHAT] (1.2 equiv.) were combined in DCM (0.01 M) and the mixture was manually shaken for a few seconds. The resulting liquid was directly purified by chromatography (SiO_2 in a Pasteur pipette, DCM) then precipitated with a centrifuge in pentane to yield a purple solid (> 90%).

11-(benzylamino)-1-methoxy-5,9-dipropyl-5,9-dihydro-13bH-quinolino[2,3,4-kl]acridin-13b-ylium Δ-TRISPHAT salt ([**5b**][Δ-**8**])

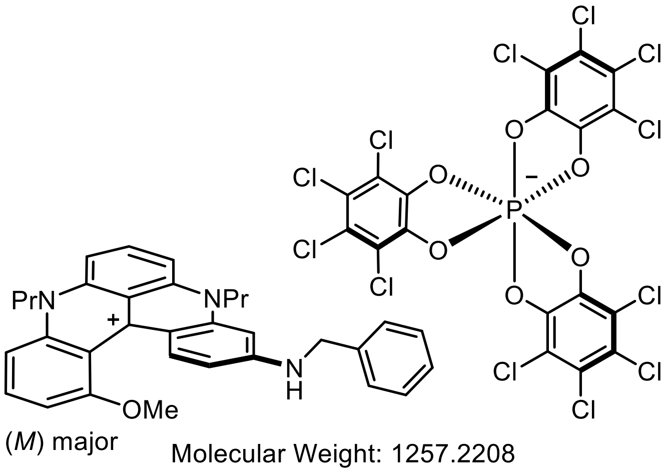

R*f* = 0.53 (100% DCM), **¹H NMR (500 MHz, $CDCl_3$, two diastereomers)** δ 7.88 (td, *J* = 8.5, 6.0 Hz, 1H), 7.74 (ddd, *J* = 8.7, 8.1, 2.0 Hz, 1H), 7.46 – 7.27 (m, 6H), 7.20 – 7.14 (m, 2H), 7.12 (dd, *J* = 8.4, 2.6 Hz, 1H), 6.95 (d, *J* = 9.2 Hz, 0.55H), 6.89 (dd, *J* = 9.3, 1.9 Hz, 0.45H), 6.84 (d, *J* = 8.1 Hz, 0.45H), 6.80 (d, *J* = 8.0 Hz, 0.55H), 6.61 – 6.24 (m, 2H), 4.67 – 4.50 (m, 2H), 4.47 – 4.33 (m, 1H), 4.29 – 4.04 (m, 3H), 3.78 (s, 1.3H), 3.66 (s, 1.7H), 2.12 – 1.97 (m, 2H), 1.97 – 1.86 (m, 1H), 1.81 – 1.59 (m, 1H), 1.19 (t, *J* = 7.4 Hz, 3H), 1.04 (t, *J* = 7.4 Hz, 3H), **¹³C NMR (126 MHz, $CDCl_3$, two diastereomers)** δ 158.7, 158.6, 155.5, 155.2, 143.8(5), 143.7(7), 143.3, 143.2, 142.9, 142.8, 142.1, 142.0, 139.2, 139.1(5), 139.1(2), 137.0, 136.9, 135.8(4), 135.8(0), 135.0, 134.9, 134.0, 133.9, 129.0(7), 129.0(4), 127.8(9), 127.8(5), 127.3, 127.2, 122.6, 116.9, 116.8, 114.7, 114.6, 114.2, 114.0, 108.7(1), 108.6(5), 107.2, 107.1, 104.8, 104.7, 104.4, 104.2(1), 104.1(5), 55.2, 55.0, 51.1, 50.8(3), 50.7(7), 48.0(0), 47.9(5), 19.7, 19.0, 18.9, 11.3(4), 11.2(9), **³¹P NMR (121 MHz, $CDCl_3$)** δ-81.06, **¹H NMR (500 MHz, CD_3CN)** δ 7.96 (t, *J* = 8.5 Hz, 1H), 7.81 (dd, *J* = 8.9, 8.1 Hz, 1H), 7.54 (dd, *J* = 9.2, 1.2 Hz, 1H), 7.48 – 7.42 (m, 2H), 7.42 – 7.38 (m, 2H), 7.36 (dd, *J* = 8.7, 2.1 Hz, 2H), 7.33 – 7.29 (m, 2H), 6.98 (dd, *J* = 8.3, 0.9 Hz, 1H), 6.90 (dt, *J* = 9.3, 2.0 Hz, 1H), 6.76 (broad s, 1H), 6.41 (broad s, 1H), 4.74 – 4.59 (m, 2H), 4.44 (ddd, *J* = 16.2, 11.0, 5.8 Hz, 1H), 4.37 – 4.14 (m, 3H), 3.79 (s, 3H), 2.03 – 1.97 (m, 1H), 1.91 – 1.78 (m, 1H), 1.68 – 1.49 (m, 2H), 1.13 (t, *J* = 7.4 Hz, 3H), 1.07 (t, *J* = 7.4 Hz, 3H), **IR (neat, cm^{-1}):** ν = 3402.24, 2961.15, 2926.40, 1626.03, 1600.58, 1586.53, 1523.10, 1492.27, 1449.00, 1390.48, 1338.46, 1301.46, 1259.00, 1235.94, 1210.16, 1171.25, 1156.65, 1140.48, 1080.74, 1007.72, 991.16, 823.94, 756.67, 718.90, **MP** = 169 °C.

11-(benzylamino)-1-methoxy-5,9-dipropyl-5,9-dihydro-13bH-quinolino[2,3,4-kl]acridin-13b-ylium Λ-TRISPHAT salt ([**5b**][Λ-**8**])

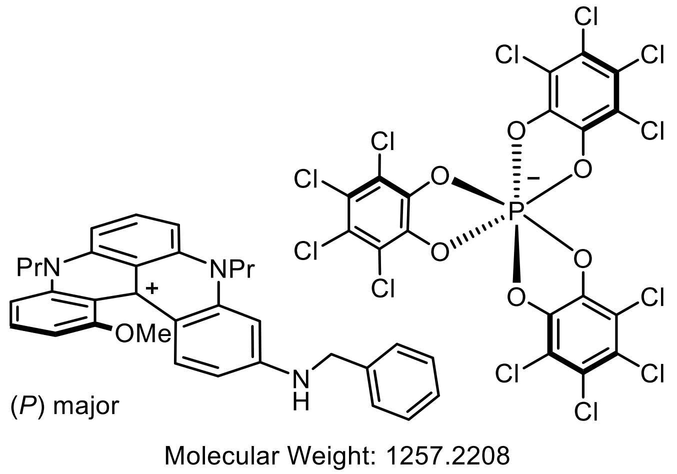

All classical spectroscopic data are in accordance with those of [Δ-**8**][**5b**].

(*S*)-1-methoxy-11-((1-phenylethyl)amino)-5,9-dipropyl-5,9-dihydro-13bH-quinolino[2,3,4-kl]acridin-13b-ylium Δ-TRISPHAT salt ([(*S*)-**5f**][Δ-**8**])

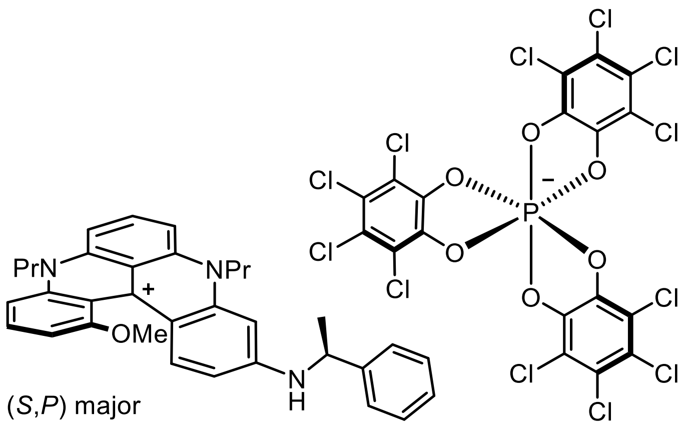

R_f = 0.49 (100% DCM), **^{1}H NMR (500 MHz, $CDCl_3$, two diastereomers)** δ 7.86 (t, *J* = 8.5 Hz, 1H), 7.72 (td, *J* = 8.4, 6.4 Hz, 1H), 7.48 – 7.27 (m, 6H), 7.21 – 7.06 (m, 3H), 6.87 – 6.76 (m, 1H), 6.66 – 6.60 (m, 1H), 6.25 – 5.90 (m, 1H), 4.79 – 4.69 (m, 0.6H), 4.66 (t, *J* = 6.0 Hz, 0.4H), 4.48 – 4.33 (m, 1H), 4.23 – 3.97 (m, 2.4H), 3.83 – 3.71 (m, 0.6H), 3.76 (s, 1.8H), 3.68 (s, 1.2H), 2.08 – 1.97 (m, 3H), 1.80 (d, *J* = 6.7 Hz, 1.8H), 1.76 – 1.67 (m, 1H), 1.64 (d, *J* = 6.7 Hz, 1.2H), 1.19 (t, *J* = 7.4 Hz, 1.8H), 1.17 (t, *J* = 7.4 Hz, 1.2H), 1.11 (t, *J* = 7.3 Hz, 1.8H), 0.90 (t, *J* = 7.3 Hz, 1.2H), **^{13}C NMR (126 MHz, $CDCl_3$, two diastereomers)** δ 158.6, 158.5, 154.3, 154.2, 143.9, 143.6, 143.3, 143.1, 142.6, 142.1(2), 142.0(6), 139.2, 139.1(4), 139.0(9), 139.0, 136.0, 135.7, 135.1, 134.8, 133.9, 132.8, 129.4, 129.3, 127.7, 126.0, 125.6, 122.5, 116.9(3), 116.8(7), 115.0, 114.4, 114.1, 114.0, 108.7, 108.5, 107.3, 107.0, 104.8, 104.4(3), 104.3(8), 104.3, 104.2, 104.0, 92.8, 92.2, 55.2, 55.0, 54.9, 54.5, 51.2, 50.9, 50.7, 24.9, 24.6, 19.8, 19.7, 18.8, 18.6, 11.4, 11.3, 11.2, **^{31}P NMR (121 MHz, $CDCl_3$)** δ -80.98, **IR (neat, cm^{-1}):** ν = 3386.48, 2966.05, 2928.25, 2161.61, 2139.11, 1974.56, 1625.87, 1600.85, 1586.34, 1554.34, 1522.99, 1490.64, 1448.75, 1390.54, 1338.73, 1301.38, 1256.08, 1235.92, 1210.01, 1171.64, 1157.79, 1140.32, 1080.00, 1050.85, 1007.47, 991.31, 908.12, 823.95, 756.89, 729.12, 719.72, 700.71, 672.68, 651.32, 620.89, **MP** = 150 °C.

(*R*)-1-methoxy-11-((1-phenylethyl)amino)-5,9-dipropyl-5,9-dihydro-13bH-quinolino[2,3,4-kl]acridin-13b-ylium Δ-TRISPHAT salt ([(*R*)-**5f**][Δ-**8**])

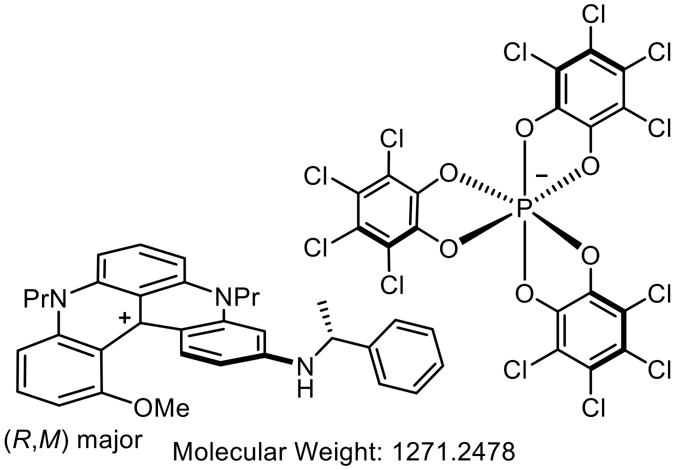

^{1}H NMR (500 MHz, $CDCl_3$, two diastereomers) δ 7.85 (dt, *J* = 14.4, 8.5 Hz, 1H), 7.73 (td, *J* = 8.4, 5.1 Hz, 1H), 7.43 – 7.23 (m, 6H), 7.20 – 6.99 (m, 4H), 6.86 – 6.75 (m, 1H), 6.32 – 5.63 (m, 1H), 4.60 – 4.45 (m, 1H), 4.45 – 4.31 (m, 1H), 4.15 (ddd, *J* = 15.9, 10.7, 6.0 Hz, 1H), 4.11 – 3.91 (m, 2H), 3.80 (s, 1H), 3.58 (s, 2H), 2.12 – 1.91 (m, 3H), 1.77 – 1.56 (m, 1H), 1.50 (d, *J* = 6.8 Hz, 2H), 1.41 (d, *J* = 6.7 Hz, 1H), 1.18 (t, *J* = 7.4 Hz, 1H), 1.17 (t, *J* = 7.4 Hz, 2H), 1.12 (t, *J* = 7.4 Hz, 1H), 0.93 (t, *J* = 7.3 Hz, 2H).

3. Dynamic chirality

3.1. VT-NMR and Line-shape analysis

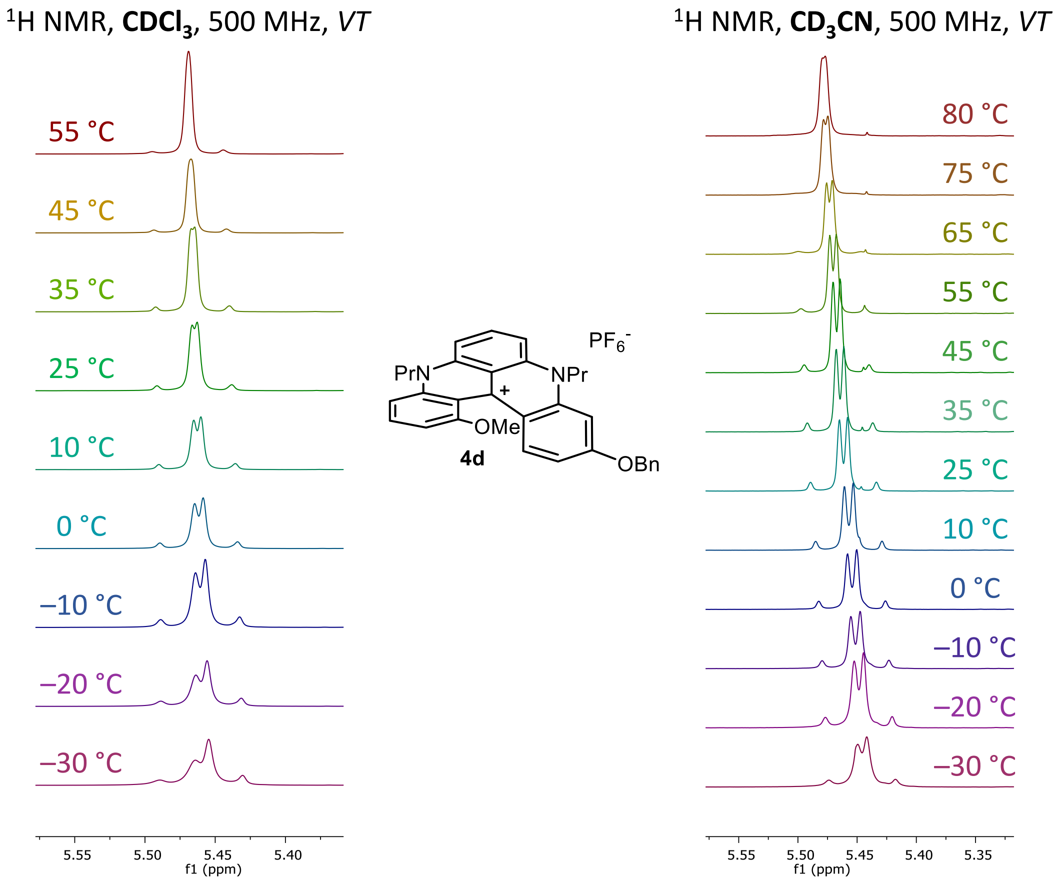

Figure S1. VT-^{1}H NMR of **4d** in $CDCl_3$ (left) and CD_3CN (right)

In CD_3CN

T [°C]	T [K]	1000/T	k	ln(k)
-30	243	4.1152	1.628E-05	-11.026
-20	253	3.9526	4.898E-05	-9.924
-10	263	3.8023	3.188E-04	-8.051
0	273	3.6630	8.358E-04	-7.087
10	283	3.5336	8.891E-03	-4.723
25	298	3.3557	2.811E-02	-3.572
35	308	3.2468	1.541E-01	-1.870
45	318	3.1447	3.521E-01	-1.044
55	328	3.0488	9.873E-01	-0.013
65	338	2.9586	5.033E+00	1.616
75	348	2.8736	2.406E+01	3.180
80	353	2.8329	3.622E+01	3.590

Table S1. Rate exchange (k) in CD_3CN at different temperatures determined by line-shape fitting analysis

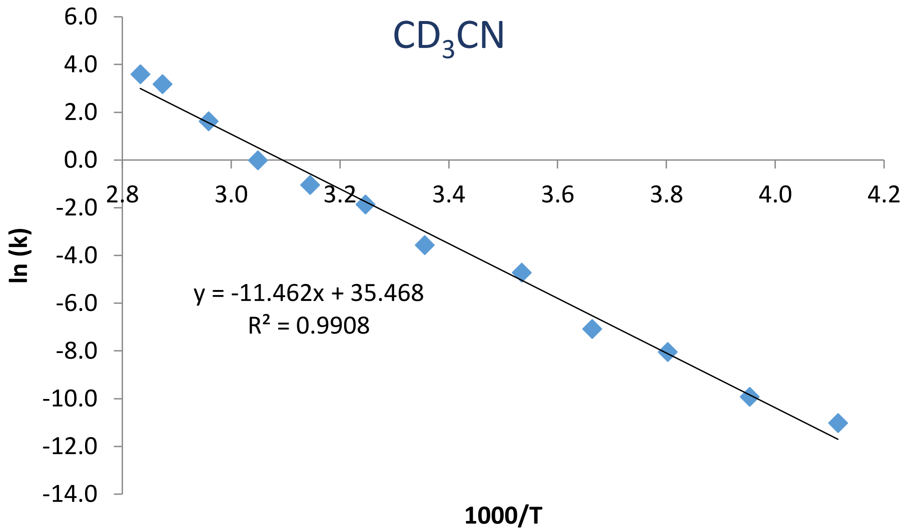

Ea	95.3	±	2.9	kJ/mol		22.8	±	0.7	kcal/mol
A	2.5E+15	±	8.5E+13						

at 25 °C					$t_{1/2}$		14	s
ΔH# = Ea - RT	92.8	±	-2.8	kJ/mol	22.2	±	-0.7	kcal/mol
ΔS# = R [ln(h*A/k*T)-1]	41.7	±	1.4	$J.K^{-1}.mol^{-1}$	10.0	±	0.3	$cal.K^{-1}.mol^{-1}$
ΔG#	80.4	±	-3.2	kJ/mol	19.2	±	-0.8	kcal/mol

Table S2. Values of activation energies for the interconversion between *M* and *P* configurations for **4d** in CD_3CN

3.2. Electronic Circular Dichroism (ECD)

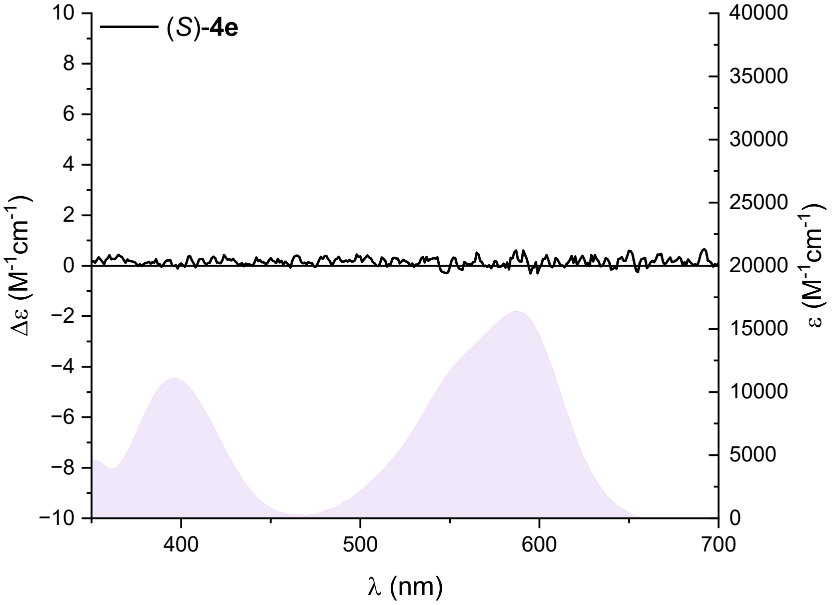

Figure S2. ECD spectrum absorption spectrum (underlying filled curve) of (*S*)-**4e** in air equilibrated chloroform at 20 °C with concentration $4 \cdot 10^{-5}$ M.

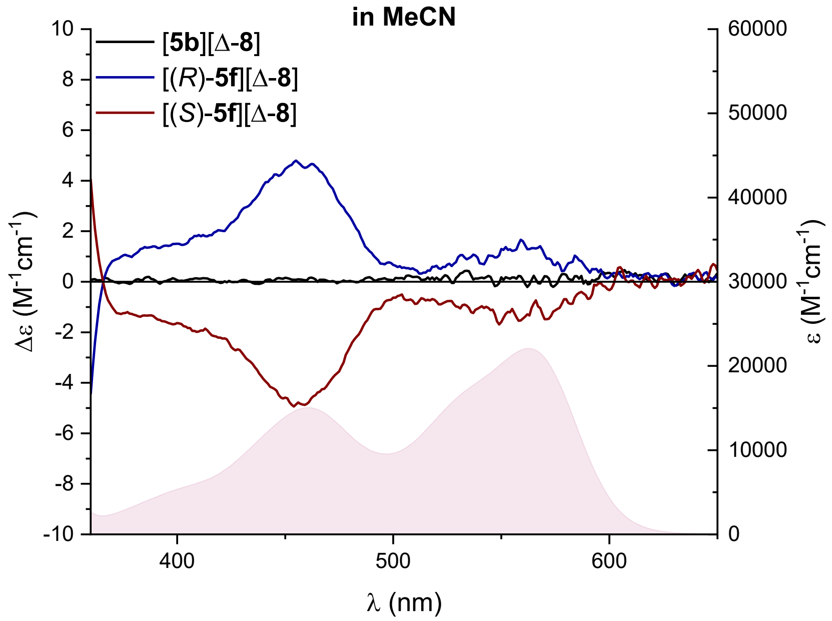

Figure S3. ECD spectra and absorption spectrum (underlying filled curve) in air-equilibrated **MeCN** at 20 °C of [(*R*)-**5f**][Δ-**8**] (blue), [**5b**][Δ-**8**] (black),and [(*S*)-**5f**][Δ-**8**] (red), *c* $3–4 \cdot 10^{-5}$ M.

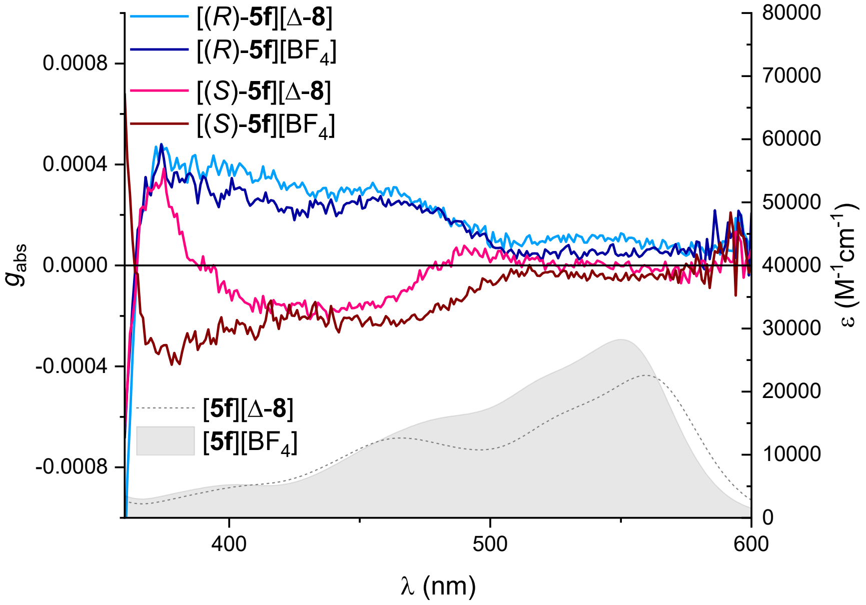

Figure S4. g_{abs} values and absorption spectra (underlying filled curve, **5f** corresponding salt) in air-equilibrated chloroform at 20 °C of [(*R*)-**5f**][Δ-**8**] (light blue), [(*R*)-**5f**][BF_4] (blue), [(*S*)-**5f**][Δ-**8**] (pink), and [(*S*)-**5f**][BF_4] (red), *c* 3–4·10^{-5} M.

4. Structural Crystallographic Analysis

4.1. Comparative analysis

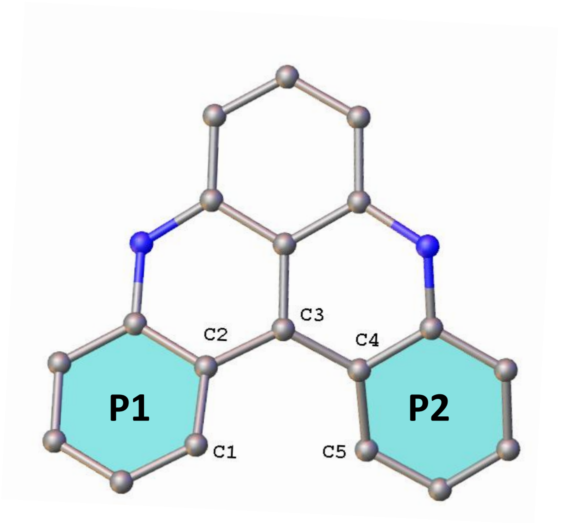

	1	1	**1**	**2**	3	3	3	3	**3**
**CCDC**	**2319499**	**2319500**	**Average** [a]	**2542615**	**2542616** mol 1	**2542616** mol 2 (B)	**2542616** mol3 (C)	**2542616** mol4 (D)	**Average** [b]
angle between planes **P1** and **P2** (º)	39.94	41.08	**40.51**	**38.6**	30.68	25.76	30.65	29.93	**29.255**
C1-C2-C3-C4 (º)	25.14	27.44	**26.29**	**17.34**	17.24	19.36	17.47	21.26	18.8325
C2-C3-C4-C5 (º)	28.26	27.44	**27.85**	**30.22**	19.94	15.51	23.26	17.16	18.9615
Average (º)	26.7	27.44	**27.07**	**23.78**	18.59	17.435	20.365	19.21	**18.9**
C1-C5 (Å)	3.203	3.192	**3.1975**	**3.103**	2.97	2.9602	2.987	2.967	**2.97105**
C1-C2-C4-C5 (º)	44.4	45.8	45.1	39.55	30.9	29.19	34.035	32.11	31.55876
RMS deviation from plane containing all 21 atoms (Å)	0.383	0.393	0.388	0.357	0.245	0.242	0.295	0.281	0.26575

Table S3. Structural features of **1**, **2**, and **3.** [a] Average over CCDC 2319499 and 2319500; [b] Average over the 4 independent molecules of CCDC 2542616

4.2. Structure CCDC 2542615

Four fluorine atoms of the hexafluorophosphate anion are disordered over two positions each. No restraints were used.

CCDC Number	2542615	
Empirical formula	C27 H29 F6 N2 O2 P	
Formula weight	558.49	
Temperature	180.00(10) K	
Wavelength	1.54184 Å	
Crystal system	Monoclinic	
Space group	*I* 2/*a*	
Unit cell dimensions	a = 17.6335(2) Å	α= 90°
	b = 14.1547(2) Å	β= 94.7470(10)°
	c = 20.4989(2) Å	γ = 90°
Volume	5098.91(11) $Å^3$	
Z	8	
Density (calculated)	1.455 Mg/m^3	
Absorption coefficient	1.611 mm^{-1}	
F(000)	2320	
Crystal size	0.873 x 0.58 x 0.097 mm^3	
Theta range for data collection	3.799 to 67.717°.	
Index ranges	$-21 \leq h \leq 21$, $-16 \leq k \leq 16$, $-24 \leq l \leq 24$	
Reflections collected	42476	
Independent reflections	4590 [R(int) = 0.0314]	
Completeness to theta = 67.684°	99.7 %	
Absorption correction	Gaussian	
Max. and min. transmission	1.000 and 0.107	
Refinement method	Full-matrix least-squares on F^2	
Data / restraints / parameters	4590 / 0 / 384	
Goodness-of-fit on F^2	1.053	
Final R indices [I>2sigma(I)]	R1 = 0.0395, wR2 = 0.1107	
R indices (all data)	R1 = 0.0441, wR2 = 0.1160	
Largest diff. peak and hole	0.357 and -0.265 e.$Å^{-3}$	

Table S4. Crystal data and structure refinement for 1,11-DMQA_**2**

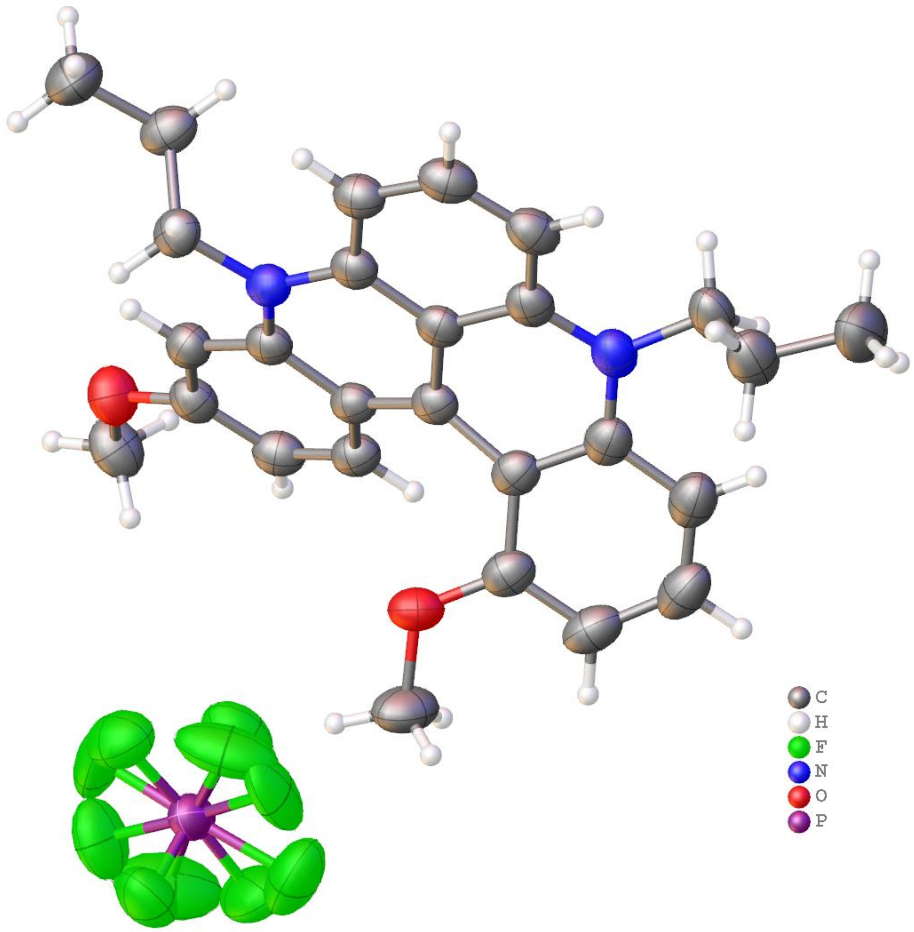

Figure S5. View of the Asymmetric unit of DMQA_**2** with displacement parameters at 50 percent probability.

4.3. Structure CCDC 2542616

There are four macrocycles and one water per asymmetric unit. The space group is non-centrosymmetric but contains symmetry element of the second kind. The crystal is therefore a racemic mixture.

CCDC Number	2542616	
Empirical formula	C27 H29.50 B F4 N2 O2.25	
Formula weight	504.83	
Temperature	120.00(10) K	
Wavelength	1.54184 Å	
Crystal system	Monoclinic	
Space group	*P c*	
Unit cell dimensions	a = 23.5293(5) Å	α= 90°
	b = 13.6598(3) Å	β= 99.633(2)°
	c = 15.2276(4) Å	γ = 90°
Volume	4825.26(19) $Å^3$	
Z	8	
Density (calculated)	1.390 Mg/m^3	
Absorption coefficient	0.914 mm^{-1}	
F(000)	2116	
Crystal size	0.27 x 0.023 x 0.018 mm^3	
Theta range for data collection	3.235 to 76.163°.	
Index ranges	$-29 \leq h \leq 26$, $-16 \leq k \leq 15$, $-19 \leq l \leq 19$	
Reflections collected	86343	
Independent reflections	16821 [R(int) = 0.0541]	
Completeness to theta = 67.684°	100.0 %	
Absorption correction	Gaussian	
Max. and min. transmission	1.000 and 0.498	
Refinement method	Full-matrix least-squares on F^2	
Data / restraints / parameters	16821 / 4 / 1328	
Goodness-of-fit on F^2	1.020	
Final R indices [I>2sigma(I)]	R1 = 0.0523, wR2 = 0.1161	
R indices (all data)	R1 = 0.0859, wR2 = 0.1327	
Absolute structure parameter	-0.05(7)	
Largest diff. peak and hole	0.393 and -0.359 e.$Å^{-3}$	

Table S5. Crystal data and structure refinement for 3-11-DMQA_**3**

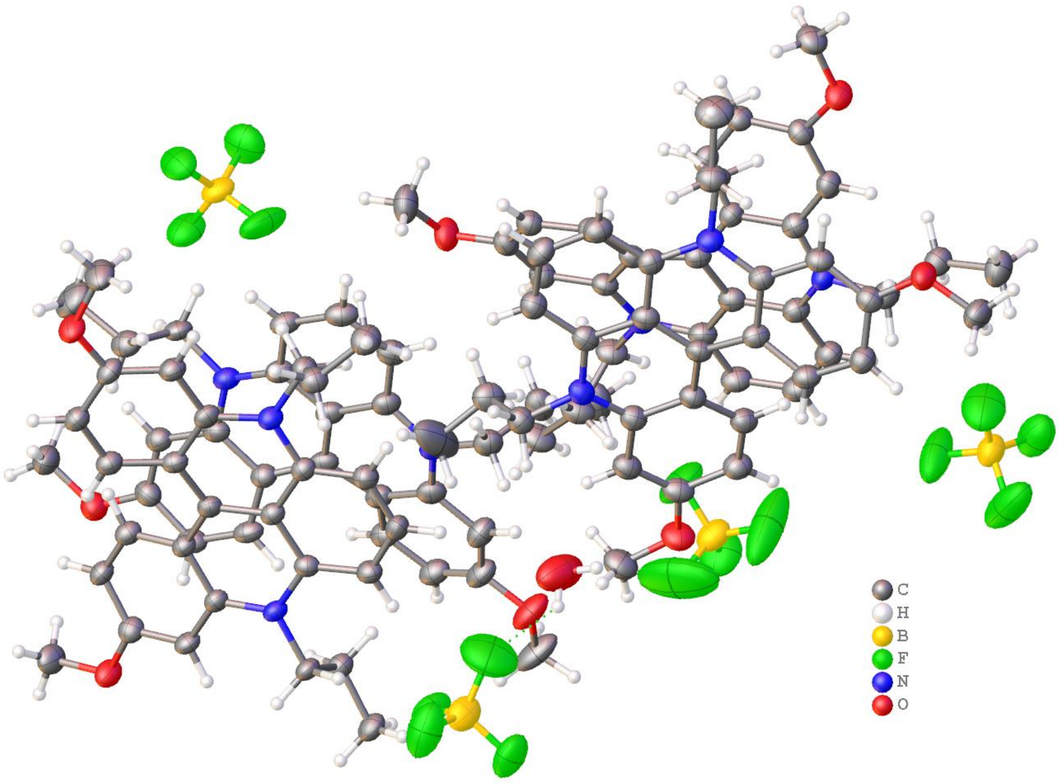

Figure S6. View of the Asymmetric unit of DMQA_**3** with displacement parameters at 50 percent probability.

5. Electrochemical properties

	Reduction process				Oxidation process		
Molecule	$E_{1/2}^{red,1}$	ΔE_p	i_b/i_f	$E_{1/2}^{red,1}$	$E_{1/2}^{ox}$	ΔE_p	i_b/i_f
**1**[a]	−1.23	75	0.96	−2.11	+0.88	90	0.83
**2**[b]	−1.28	68	0.67	−2.05	+0.87	99	0.59
**3**[b]	−1.35	90	0.51	−2.14	+0.87	126	0.50

Table S6. Electrochemical data of DMQA regioisomers in MeCN, $E_{1/2}$ (V, vs Fc^+/Fc) and peak-to-peak separations (ΔE_p in mV), and ratio between current intensity back- (i_b) and forward (i_f) for the redox processes exhibited at the Pt electrode by compounds **1**, **2**, and **3** (5×10^{-4} M) in dry acetonitrile with $[^nBu_4N][PF_6]$ 10^{-1} M as supporting electrolyte. [a] Measured with $\nu = 0.1$ V/s. [b] Measured with $\nu = 0.02$ V/s.

Molecule	HOMO (eV)	LUMO (eV)
**1**	−5.98	−3.87
**2**	−5.97	−3.82
**3**	−5.97	−3.75

Table S7. Absolute energy values of HOMO and LUMO orbitals in vacuum for DMQA regioisomers **1**, **2**, and **3** obtained from electrochemical data. HOMO ~ E^{ox}_{vacuum} (eV) and LUMO ~ E^{red}_{vacuum} (eV), where $E^{red/ox}_{vacuum} \sim -(E_{vs\ Fc} + 5.1\ V)$ as $E_{vacuum}(Fc^+/Fc) = -5.1$ V.[3]

6. Photophysical properties

*DMQA regioisomers (**1**, **2**, and **3**)*

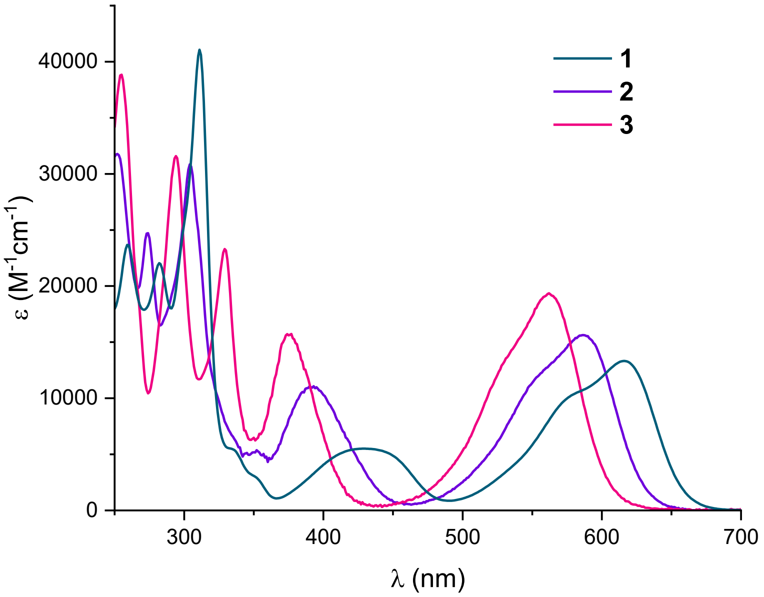

Figure S7. Absorption spectra of DMQA regioisomers **1**, **2**, and **3** in air-equilibrated MeCN at 20 °C with concentrations $1 \cdot 10^{-5}$ to $3 \cdot 10^{-5}$ M.

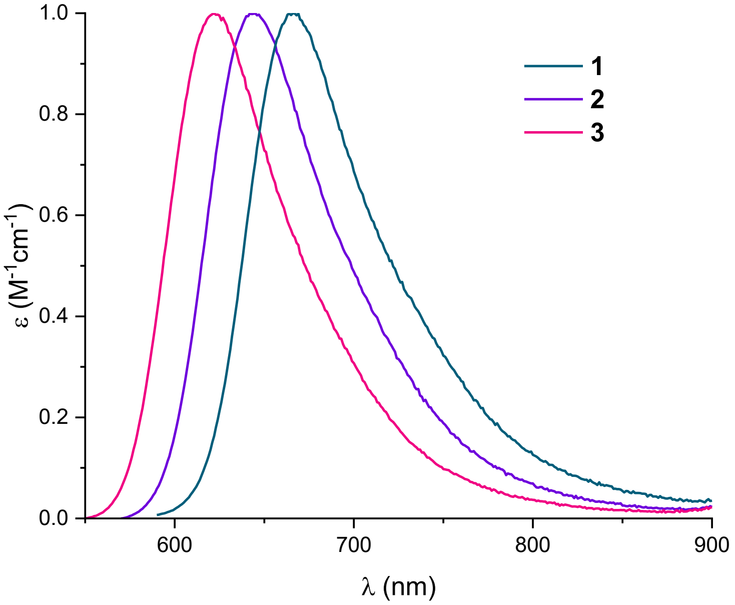

Figure S8. Emission spectra of DMQAs **1**, **2**, and **3** in air-equilibrated MeCN at 20 °C with concentrations $<1 \cdot 10^{-5}$ M.

*Ethers (**4**)*

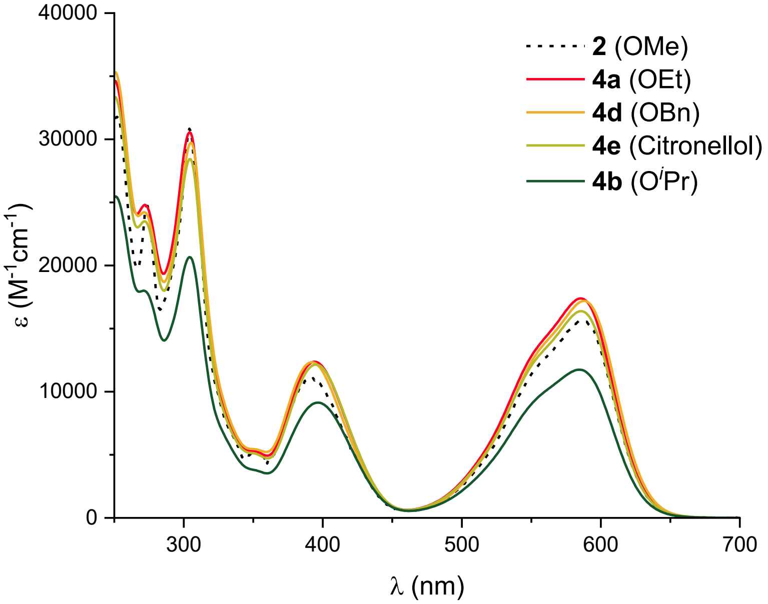

Figure S9. Absorption spectra of ethers **4** in air-equilibrated MeCN at 20 °C with concentrations $1 \cdot 10^{-5}$ to $3 \cdot 10^{-5}$ M.

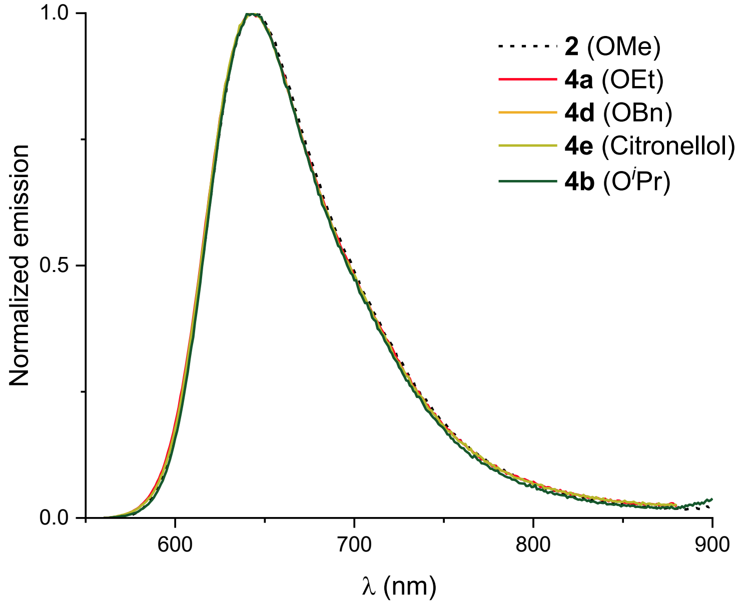

Figure S10. Emission spectra of ethers **4** in air-equilibrated MeCN at 20 °C with concentrations $<1 \cdot 10^{-5}$ M.

*Amines (**5**)*

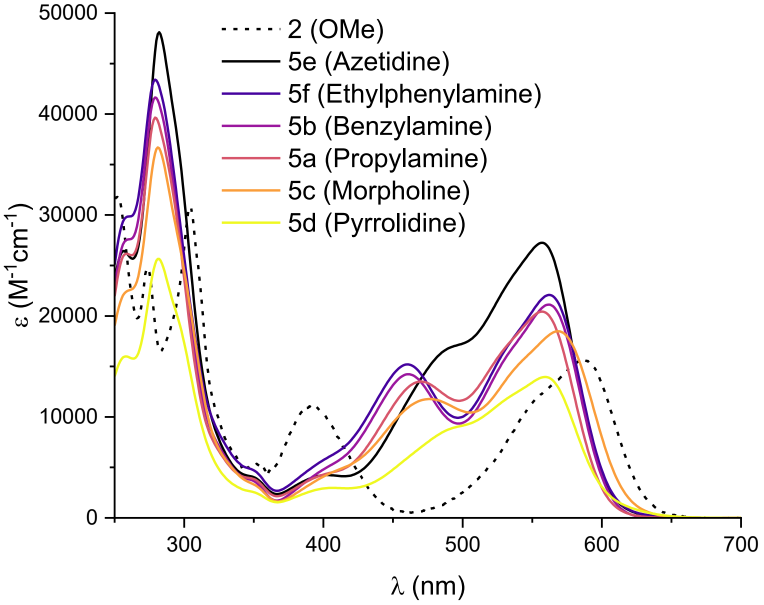

Figure S11. Absorption spectra of amines **5** in air-equilibrated MeCN at 20 °C with concentrations $1 \cdot 10^{-5}$ to $3 \cdot 10^{-5}$ M.

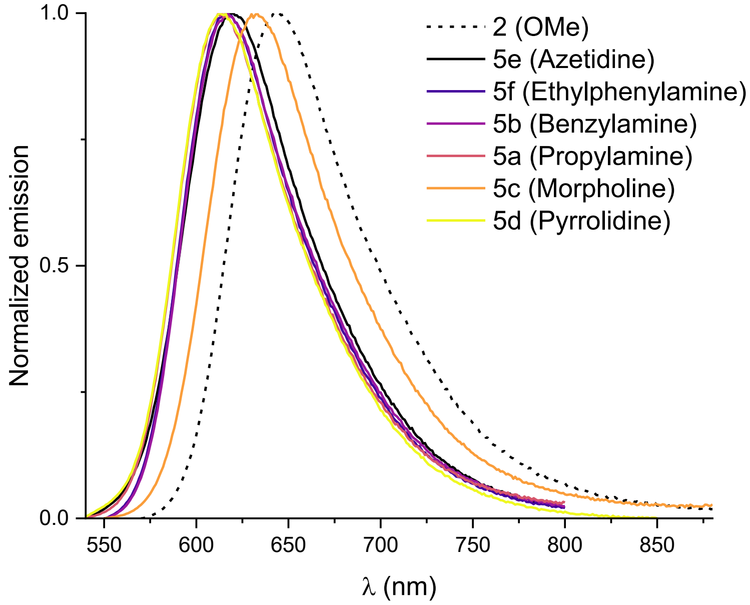

Figure S12. Emission spectra of amines **5** in air-equilibrated MeCN at 20 °C with concentrations <$1 \cdot 10^{-5}$ M.

*Phenol (**6**) and Ester (**7**)*

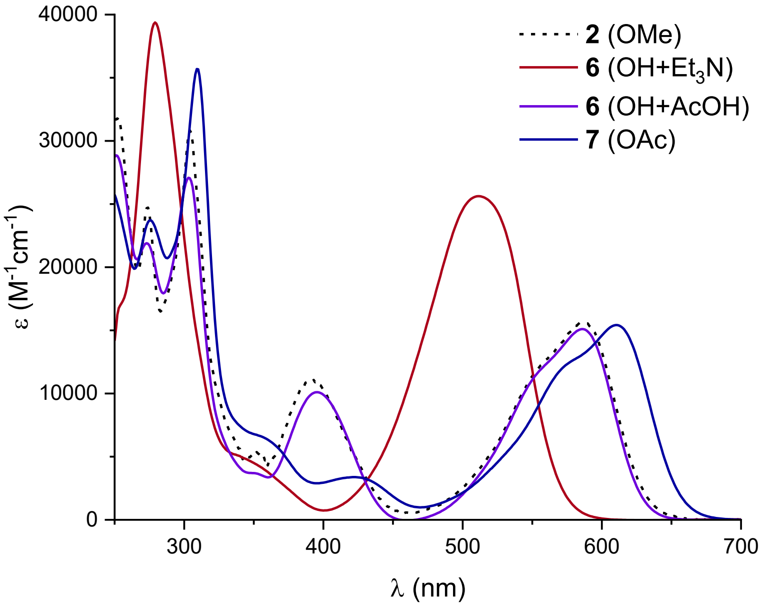

Figure S13. Absorption spectra of phenol **6** (with either Et_3N or AcOH as dopant) and ester **7** in air-equilibrated MeCN at 20 °C with concentrations $1 \cdot 10^{-5}$ to $3 \cdot 10^{-5}$ M.

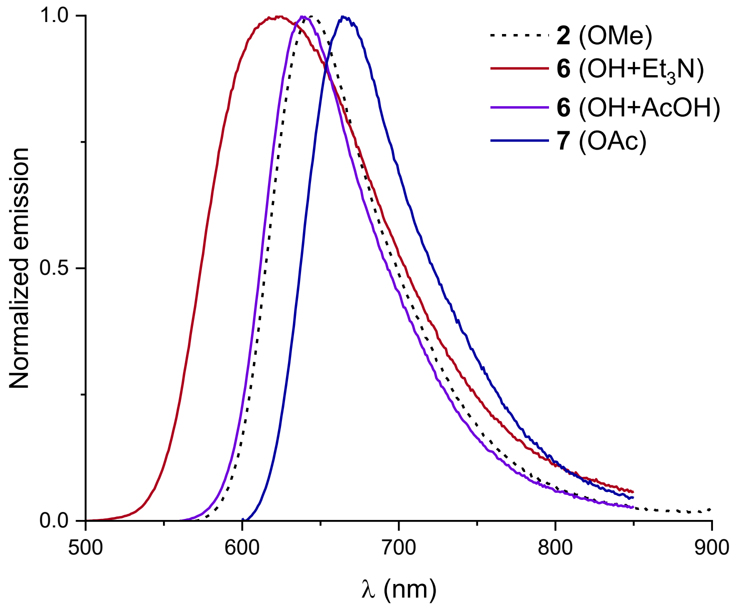

Figure S14. Emission spectra of phenol **6** (with either Et_3N or AcOH as dopant) and ester **7** in air-equilibrated MeCN at 20 °C with concentrations $<1 \cdot 10^{-5}$ M.

7. NMR & IR spectra; MRMS data

Compound 2

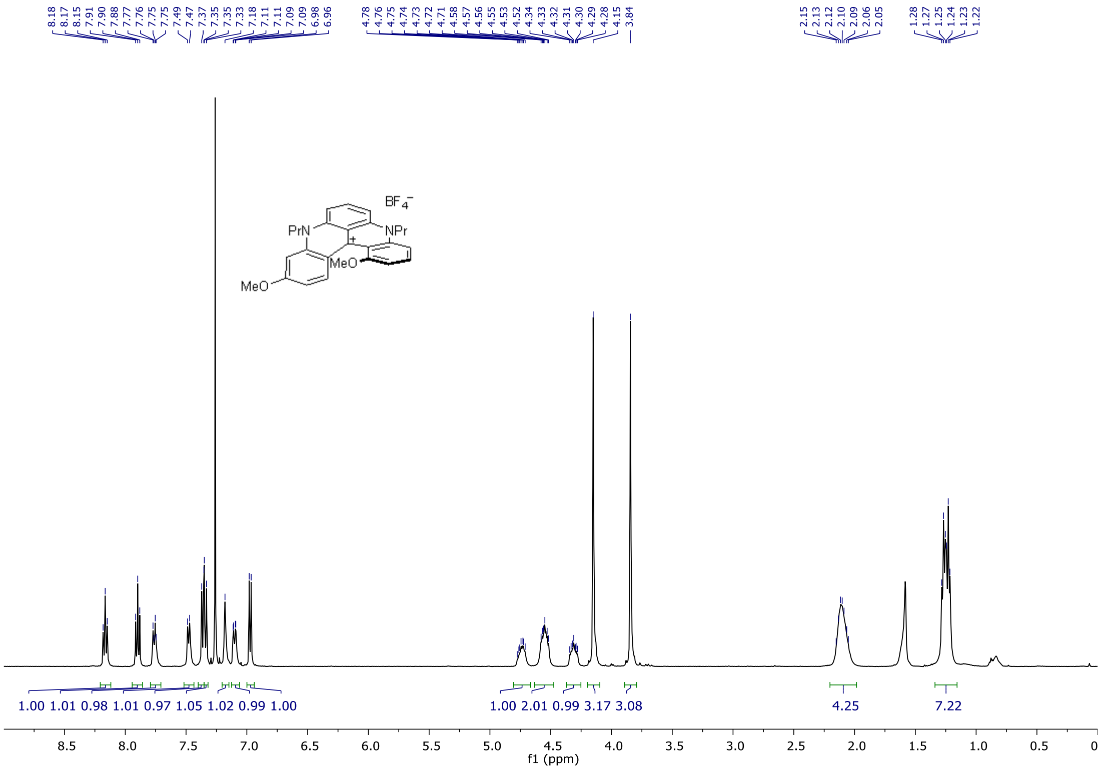

Figure S15. ^{1}H NMR (500 MHz, $CDCl_3$) spectrum of **2**

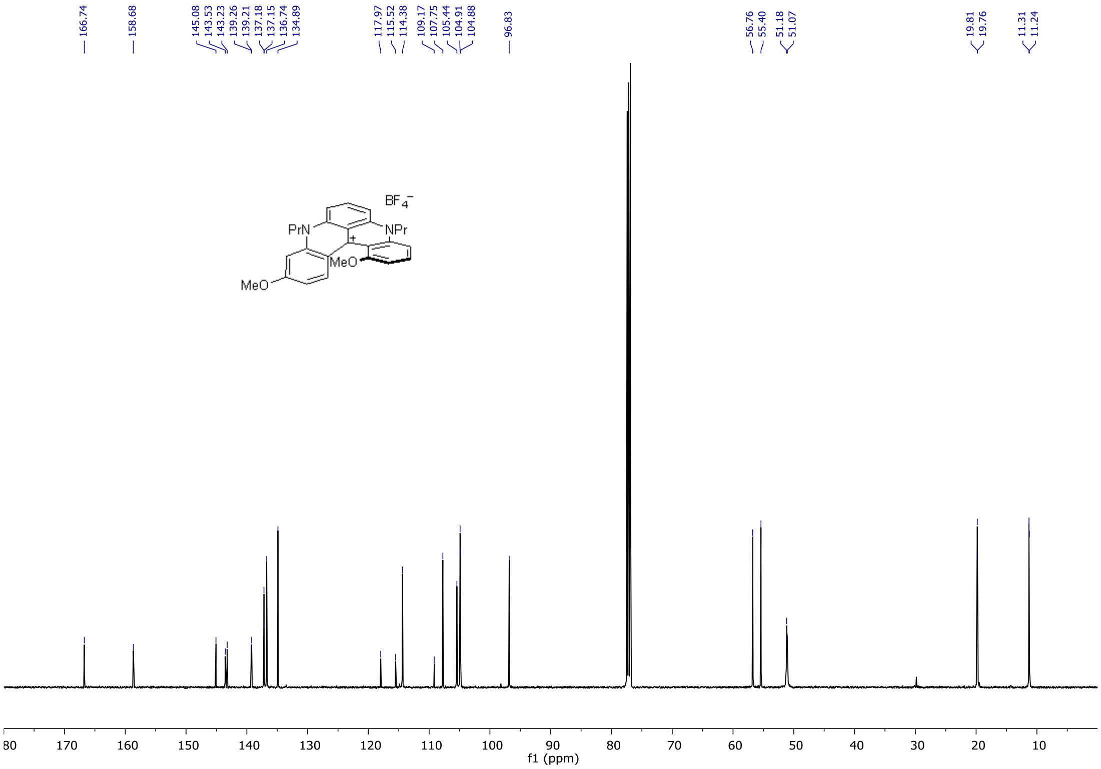

Figure S16. ^{13}C NMR (126 MHz, $CDCl_3$) spectrum of **2**

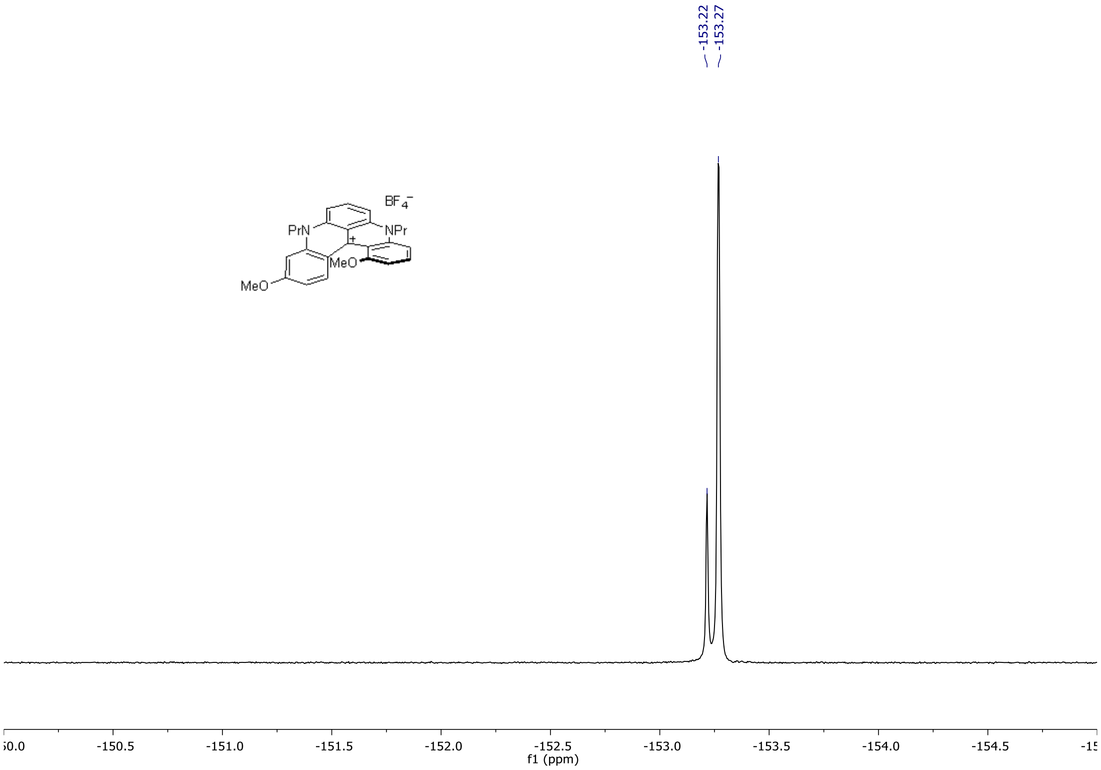

Figure S17. ^{19}F NMR (282 MHz, $CDCl_3$) spectrum of **2**

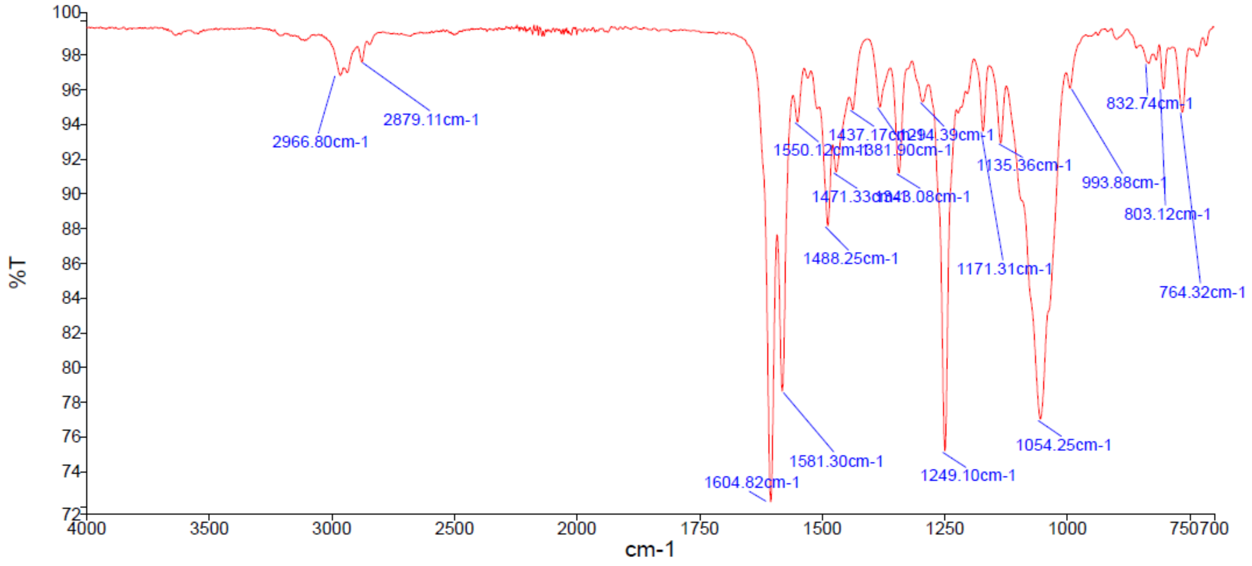

Figure S18. IR (neat) spectrum of **2**

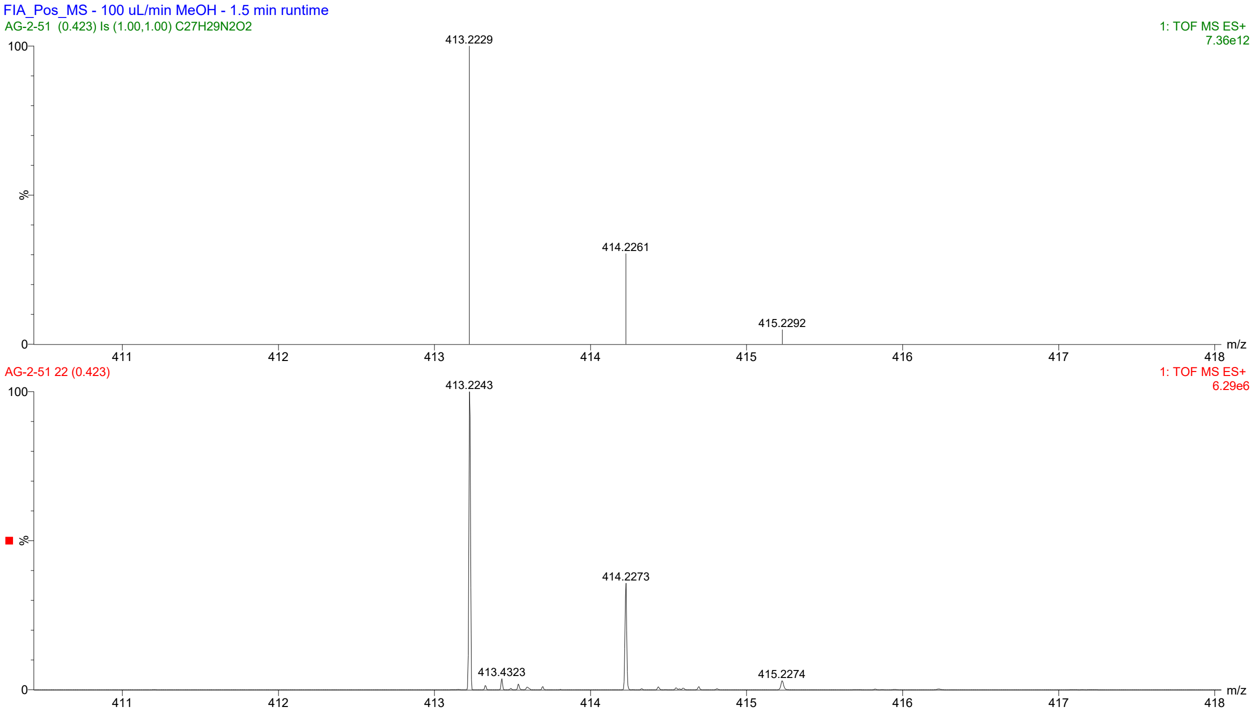

Figure S19. HRMS analysis (ESI, CH_3OH) report of **2**

Compound 2-H

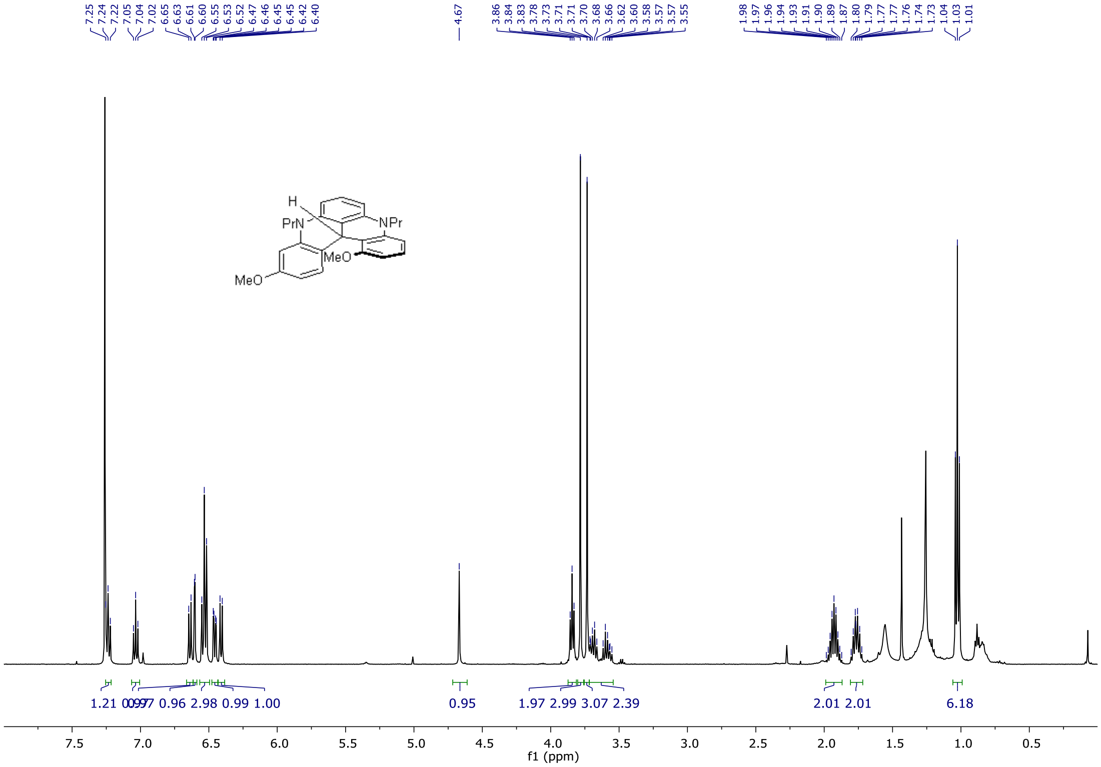

Figure S20. ^{1}H NMR (500 MHz, $CDCl_3$, $NaBH_3CN$ as additive) spectrum of **2-H**

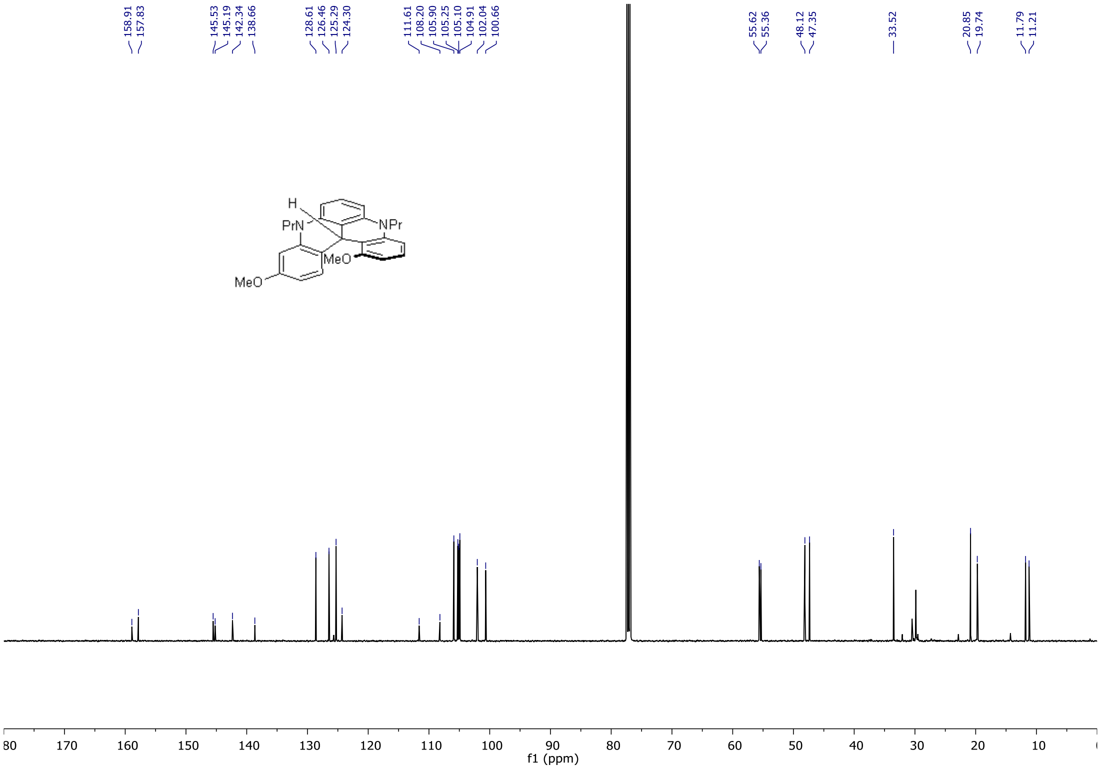

Figure S21. ^{13}C NMR (126 MHz, $CDCl_3$, $NaBH_3CN$ as additive) spectrum of **2-H**

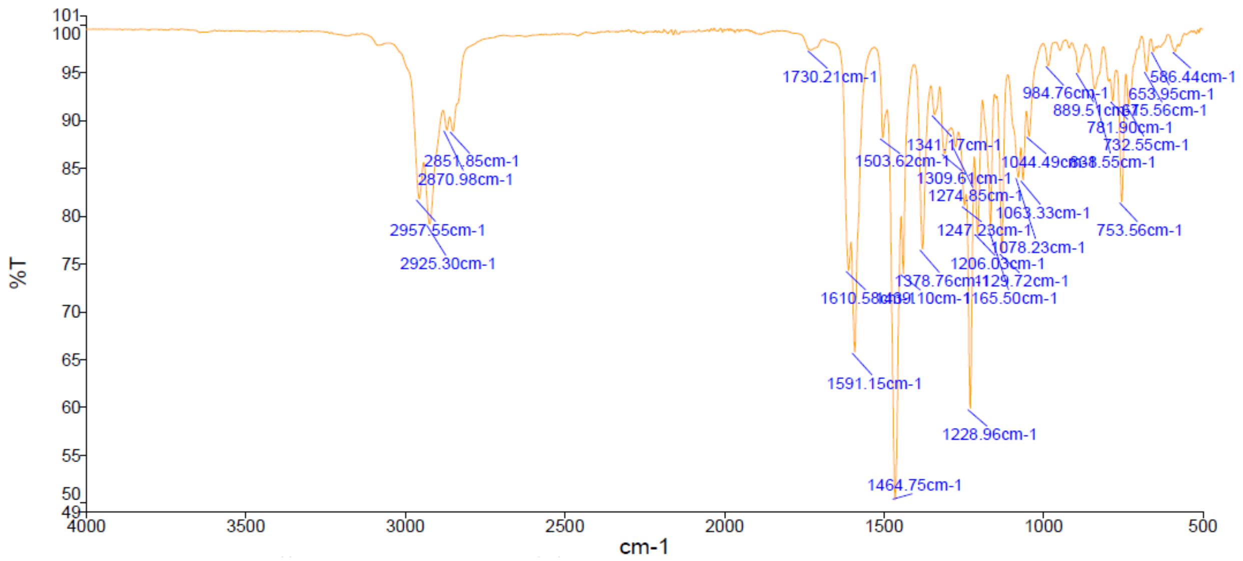

Figure S22. IR (neat) spectrum of **2-H**

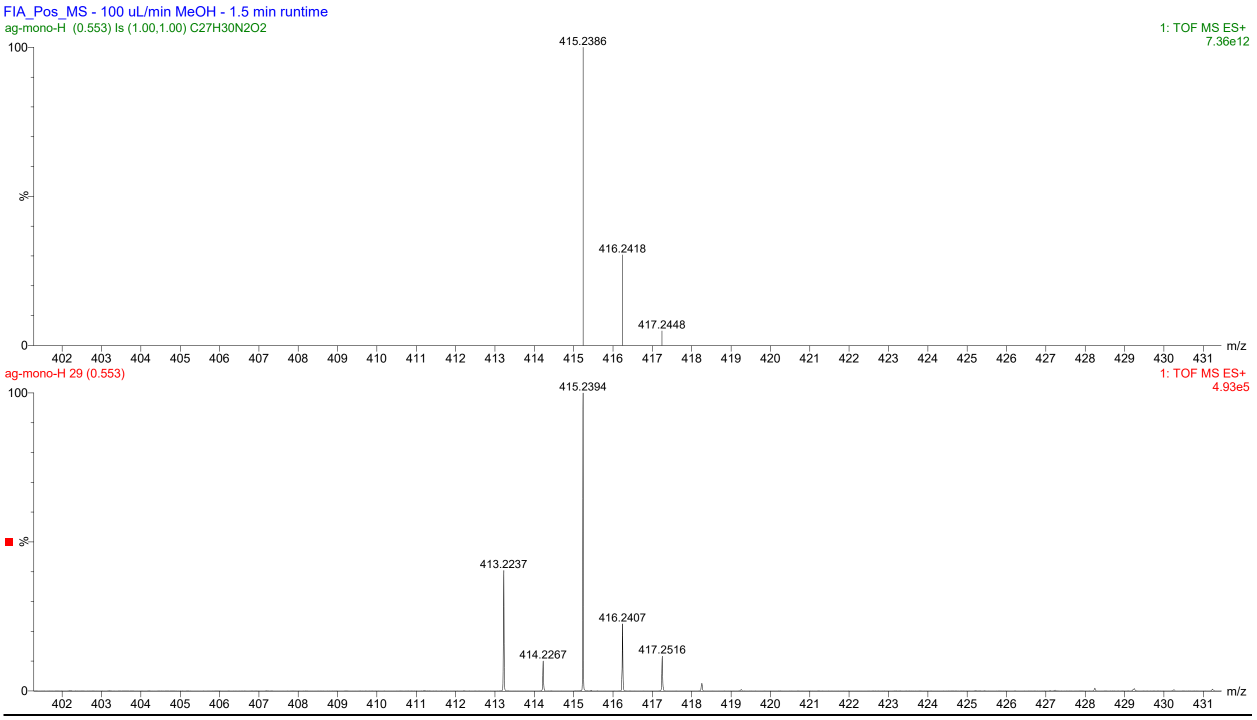

Figure S23. HRMS analysis (ESI, CH_3OH) report of **2-H**

Compound 1-Me

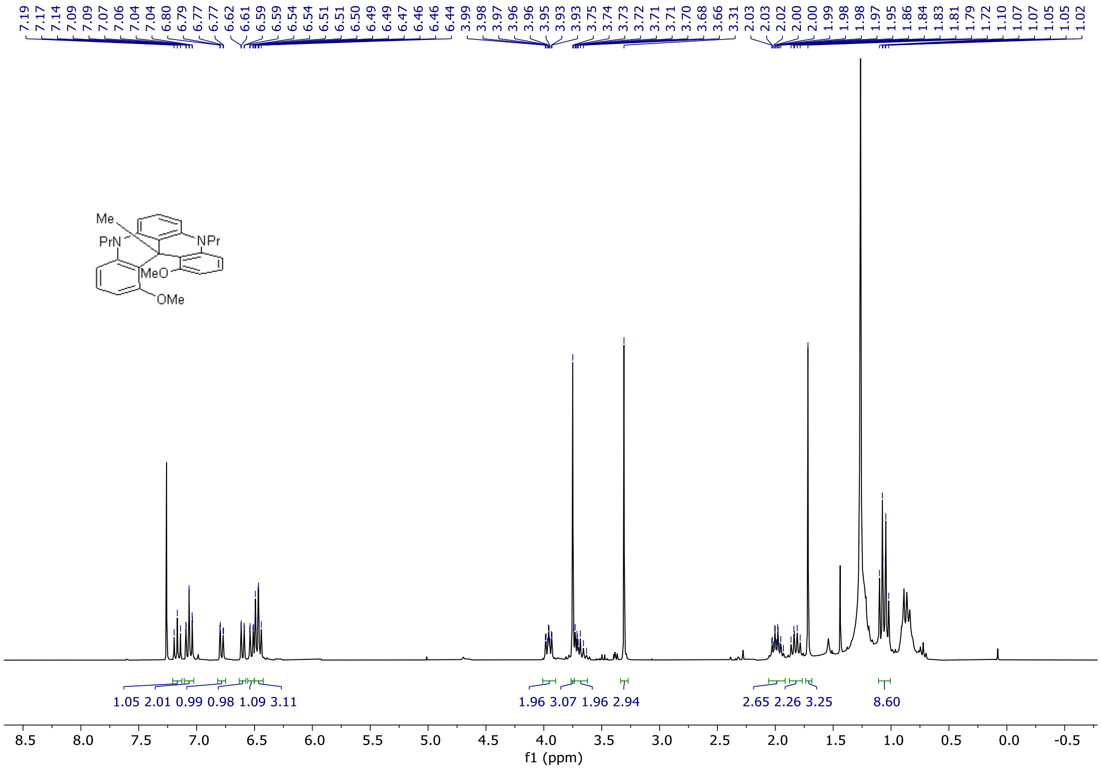

Figure S24. ^{1}H NMR (300 MHz, $CDCl_3$) spectrum of **1-Me**

Compound 3

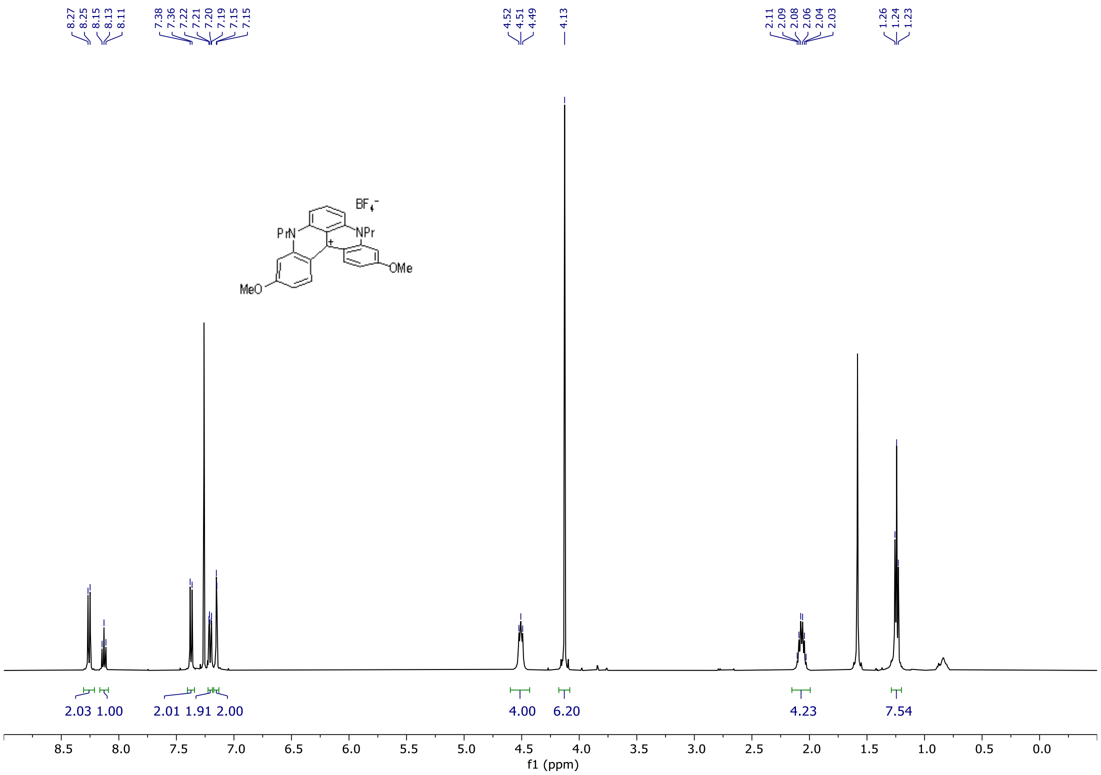

Figure S25. ^{1}H NMR (500 MHz, $CDCl_3$) spectrum of **3**

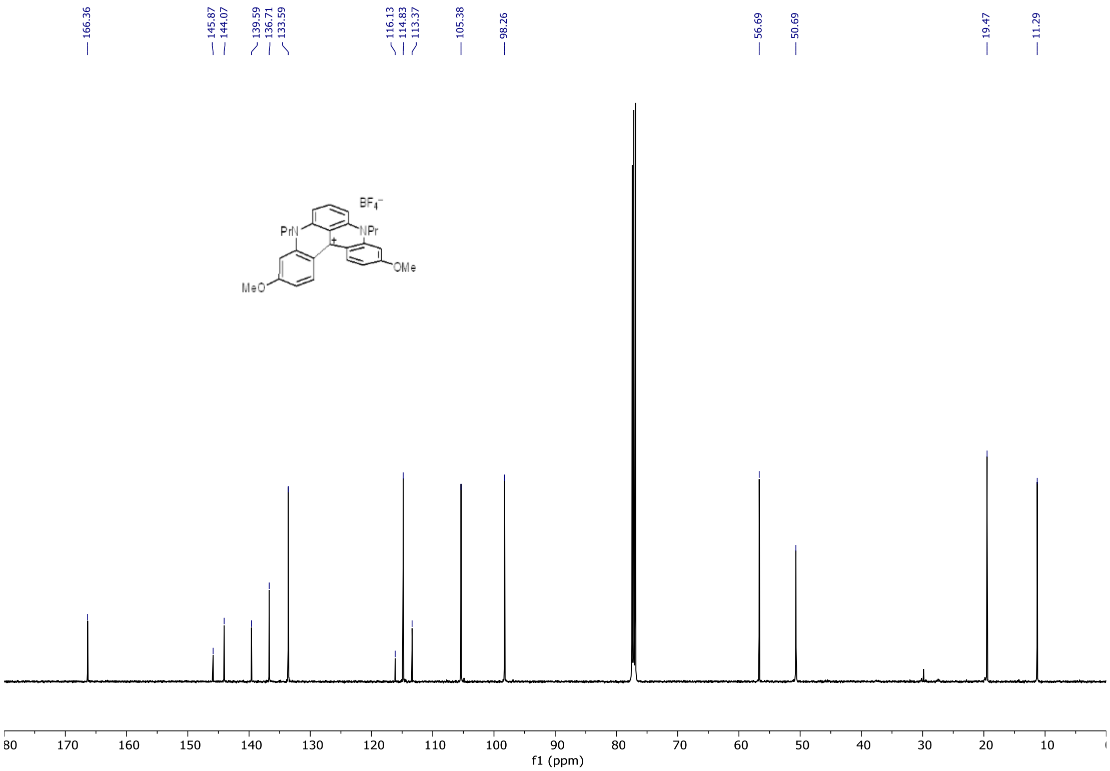

Figure S26. ^{13}C NMR (126 MHz, $CDCl_3$) spectrum of **3**

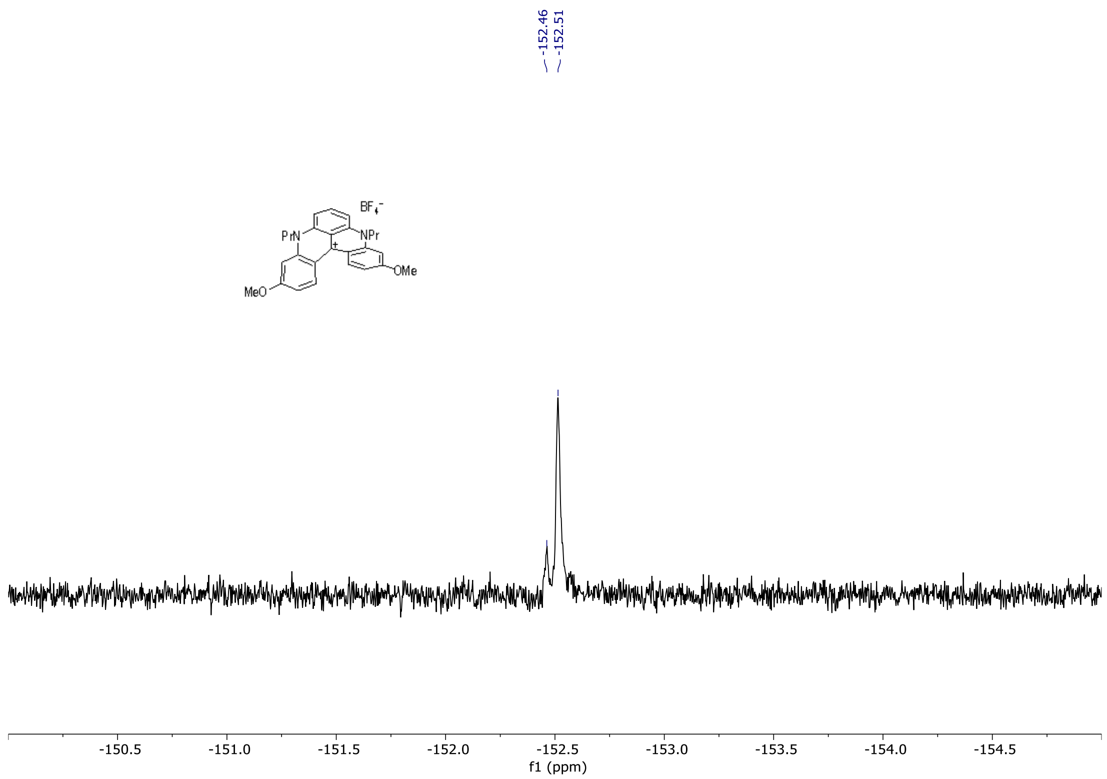

Figure S27. ^{19}F NMR (282 MHz, $CDCl_3$) spectrum of **3**

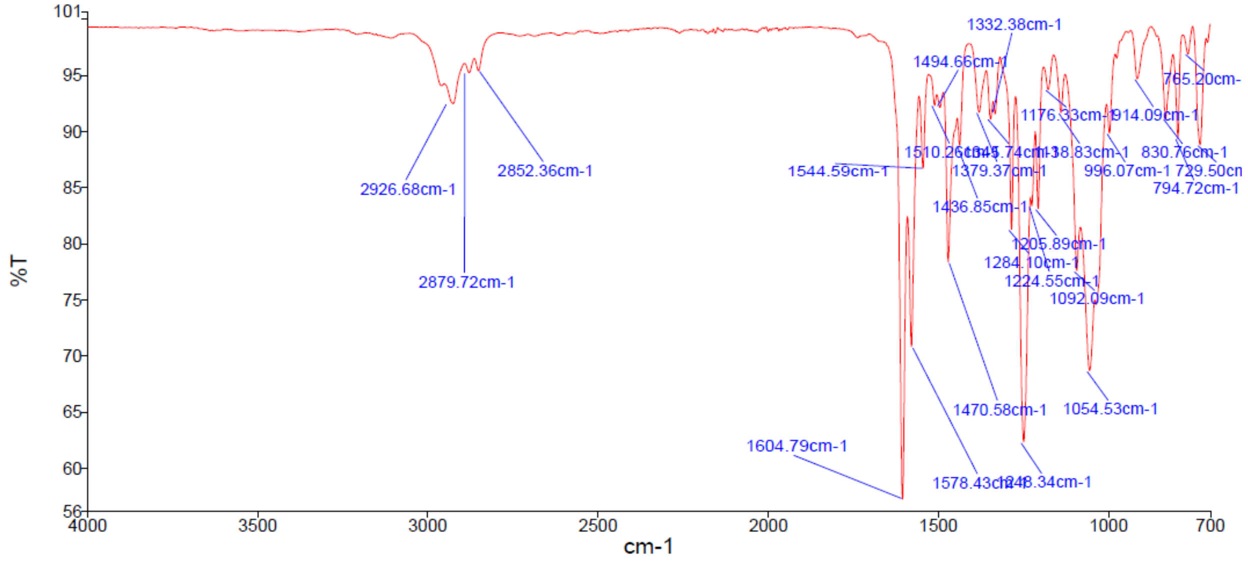

Figure S28. IR (neat) spectrum of **3**

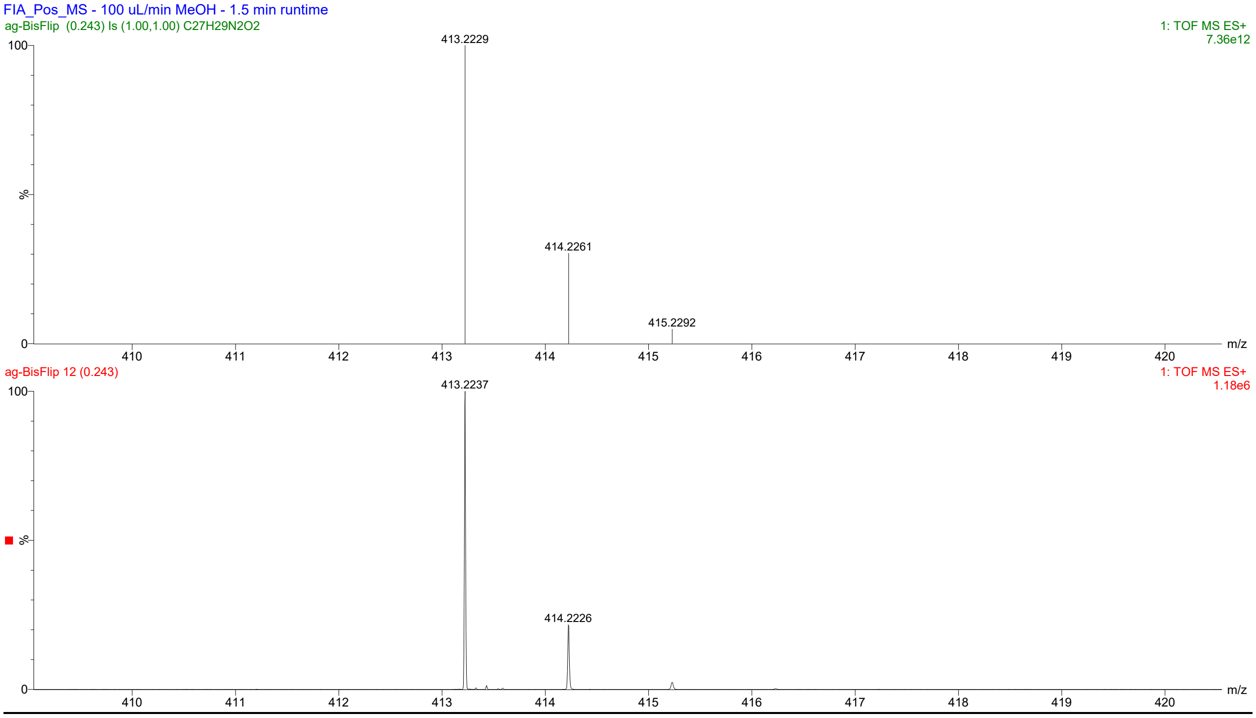

Figure S29. HRMS analysis (ESI, CH_3OH) report of **3**

Compound 4a

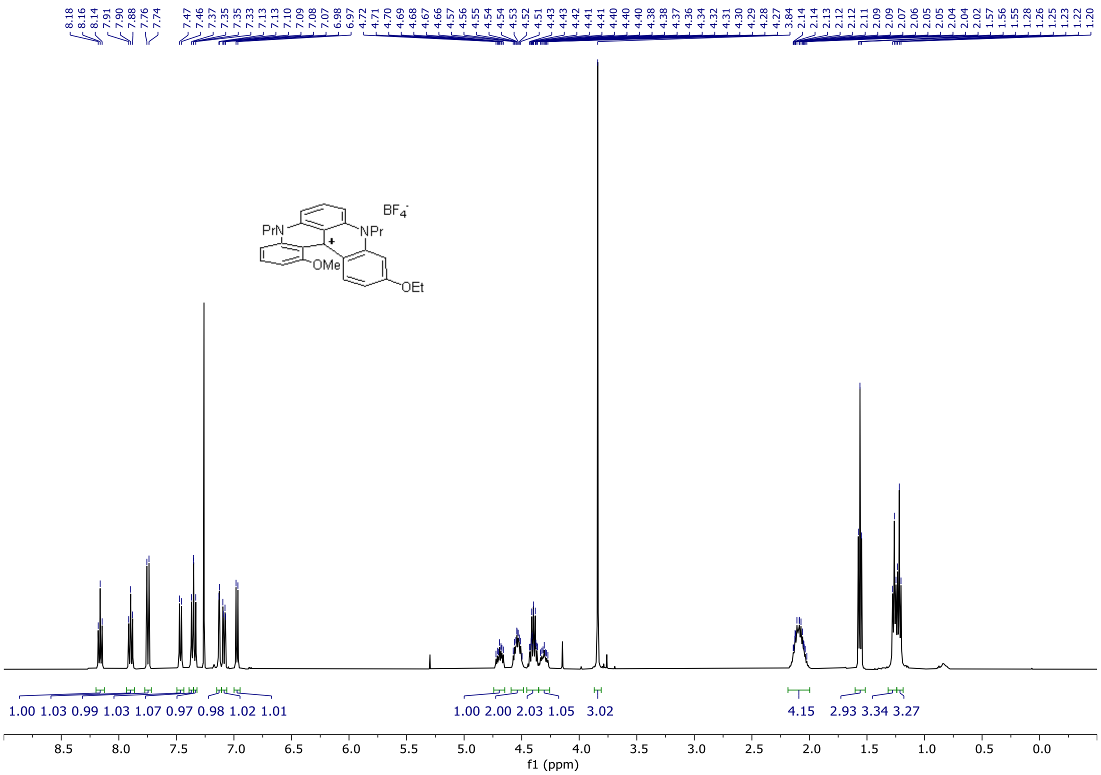

Figure S30. ^{1}H NMR (500 MHz, $CDCl_3$) spectrum of **4a**

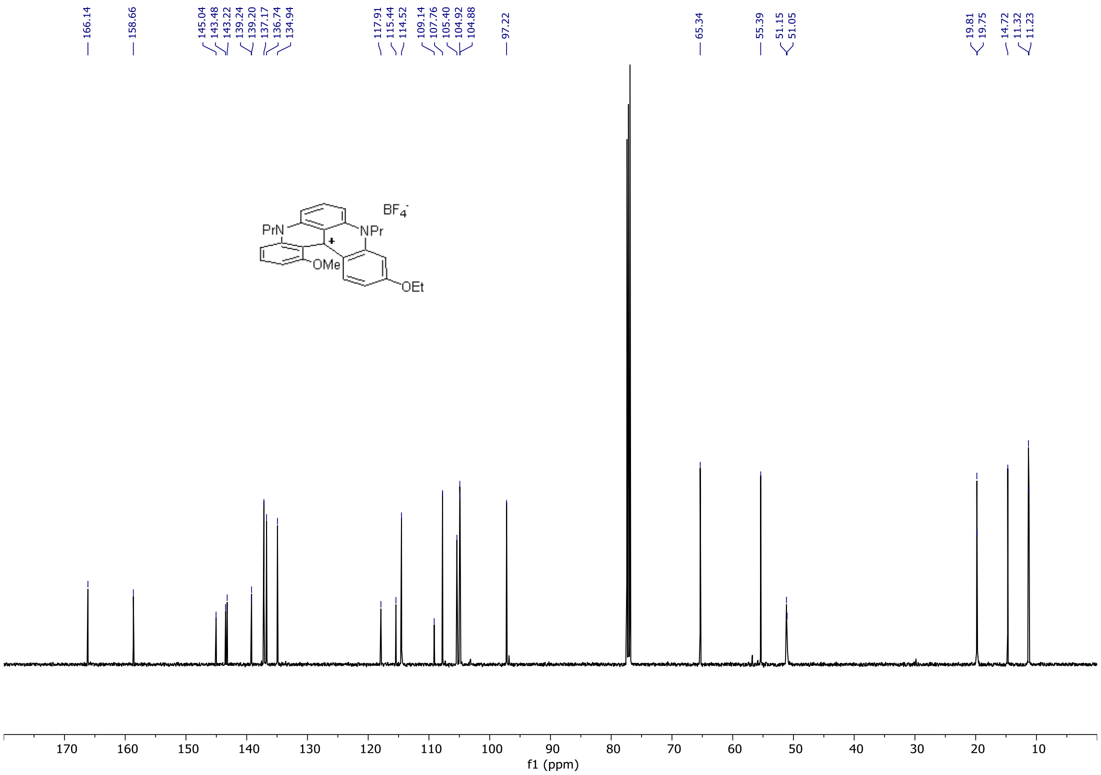

Figure S31. ^{13}C NMR (126 MHz, $CDCl_3$) spectrum of **4a**

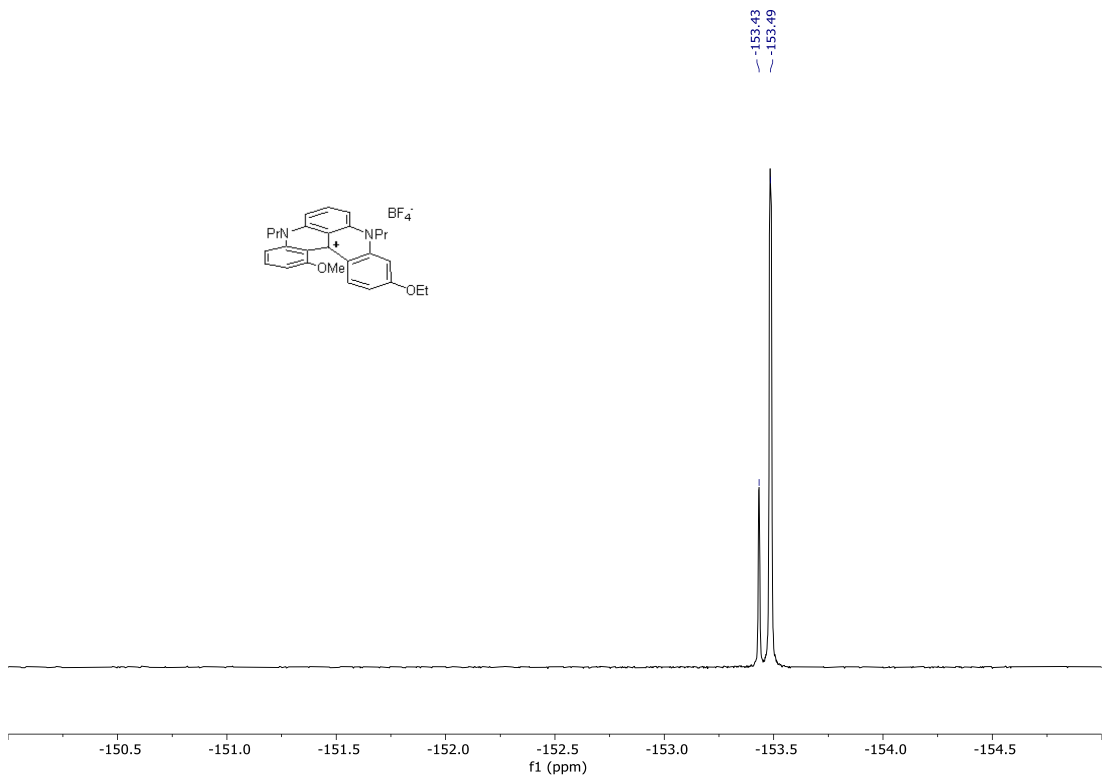

Figure S32. ^{19}F NMR (282 MHz, $CDCl_3$) spectrum of **4a**

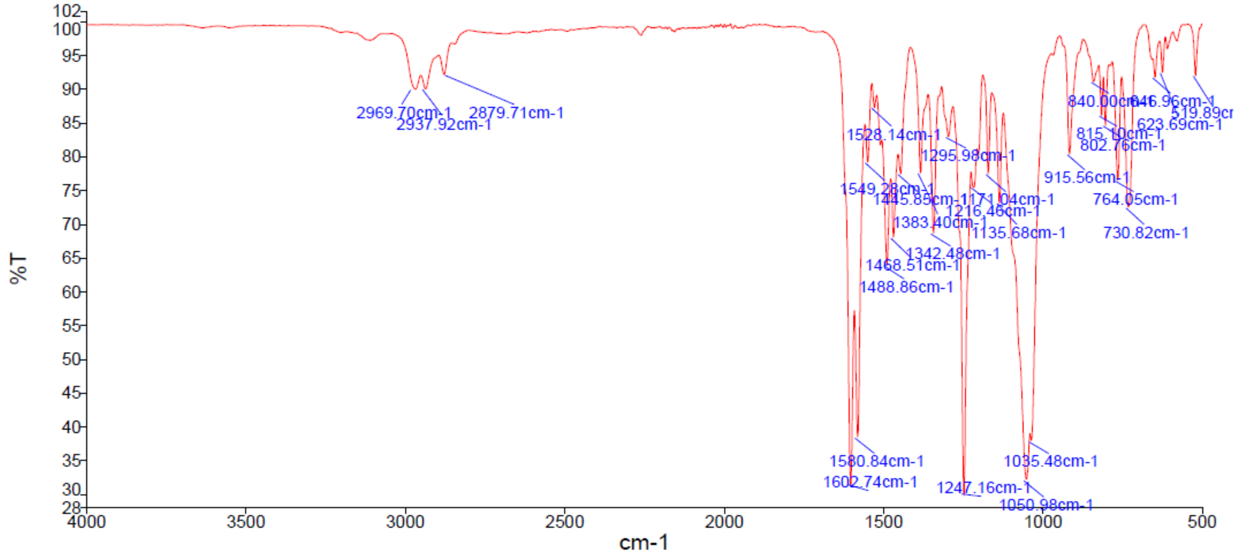

Figure S33. IR (neat) spectrum of **4a**

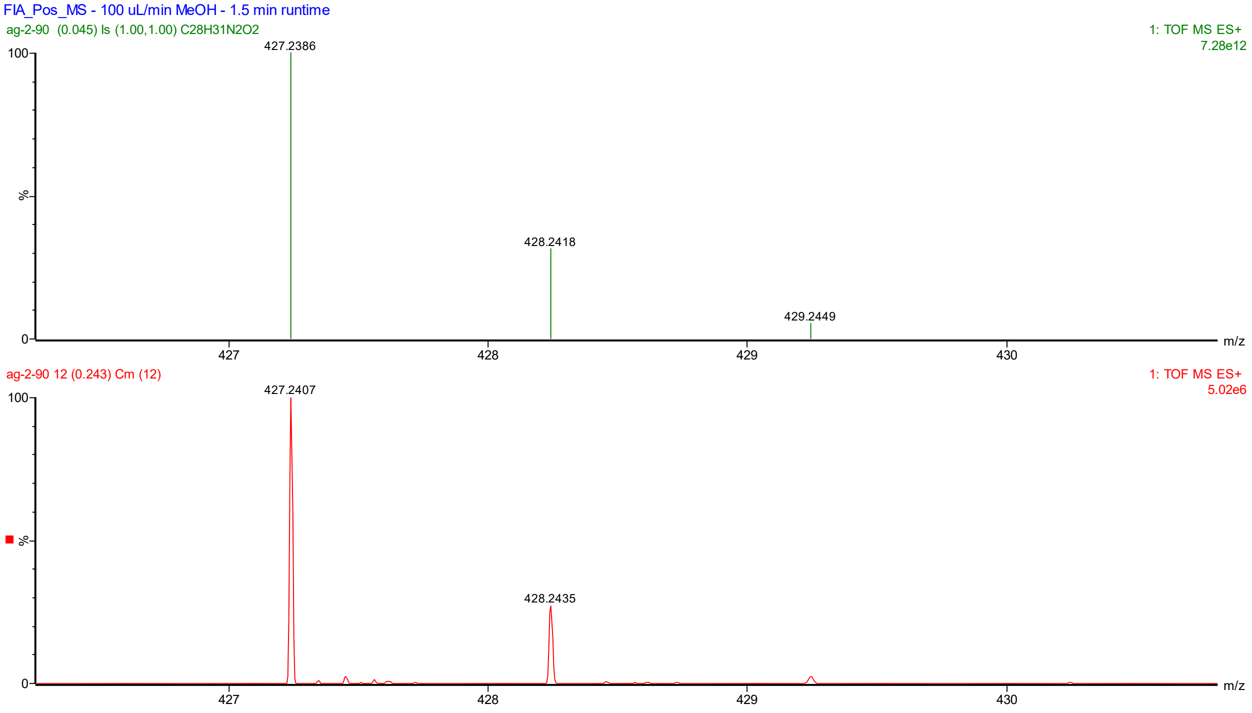

Figure S34. HRMS analysis (ESI, CH_3OH) report of **4a**

Compound 4b

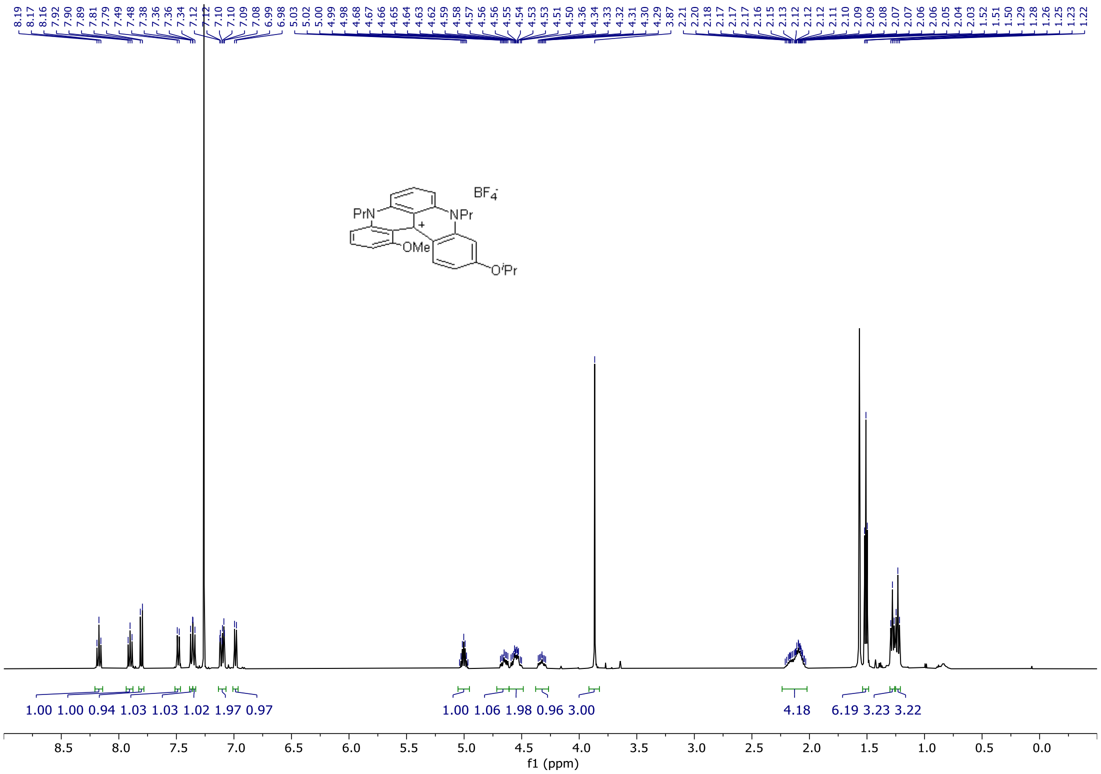

Figure S35. ^{1}H NMR (500 MHz, $CDCl_3$) spectrum of **4b**

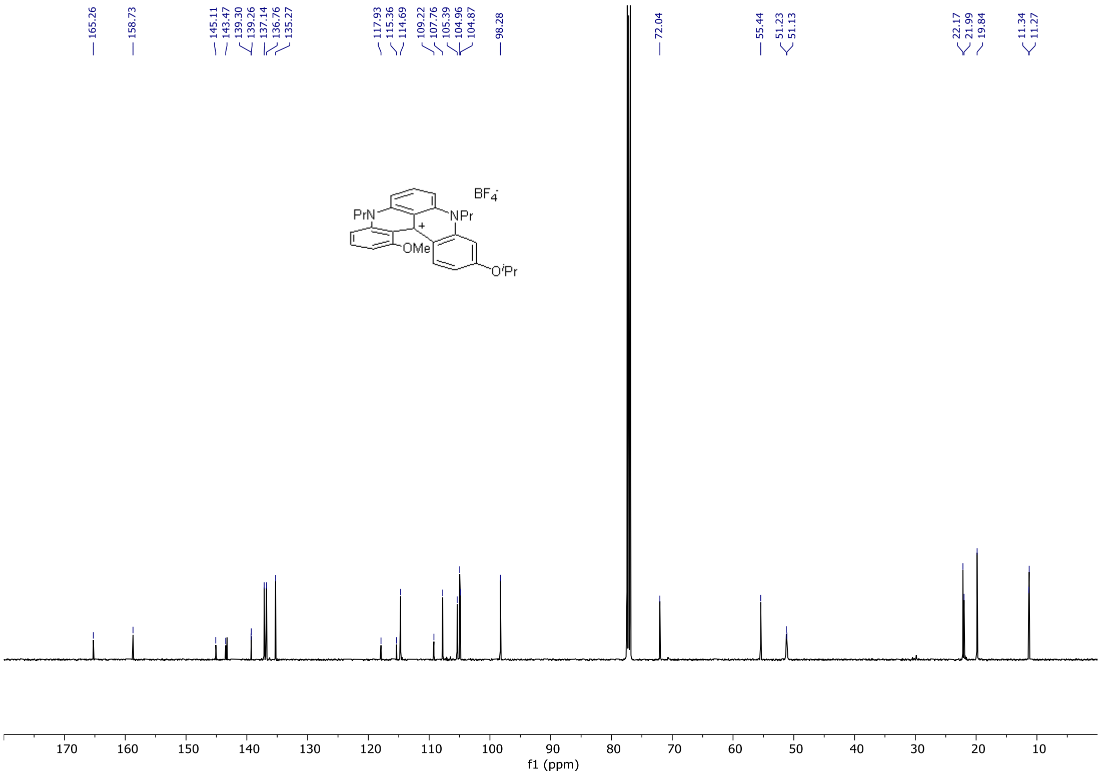

Figure S36. ^{13}C NMR (126 MHz, $CDCl_3$) spectrum of **4b**

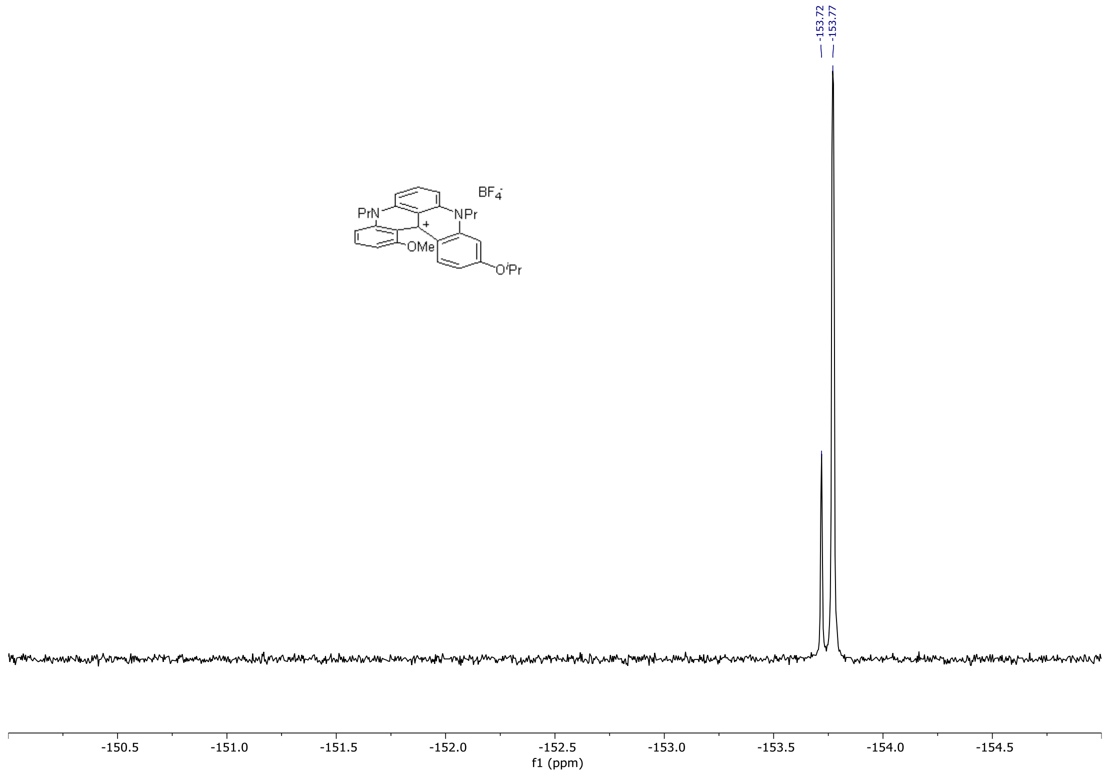

Figure S37. ^{19}F NMR (282 MHz, $CDCl_3$) spectrum of **4b**

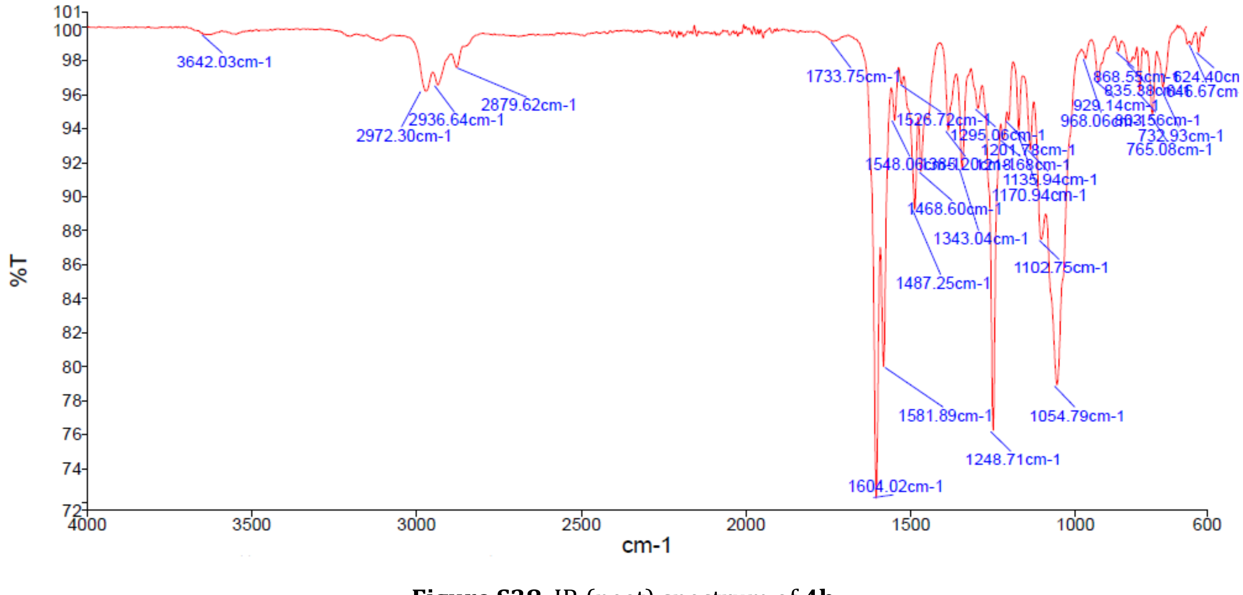

Figure S38. IR (neat) spectrum of **4b**

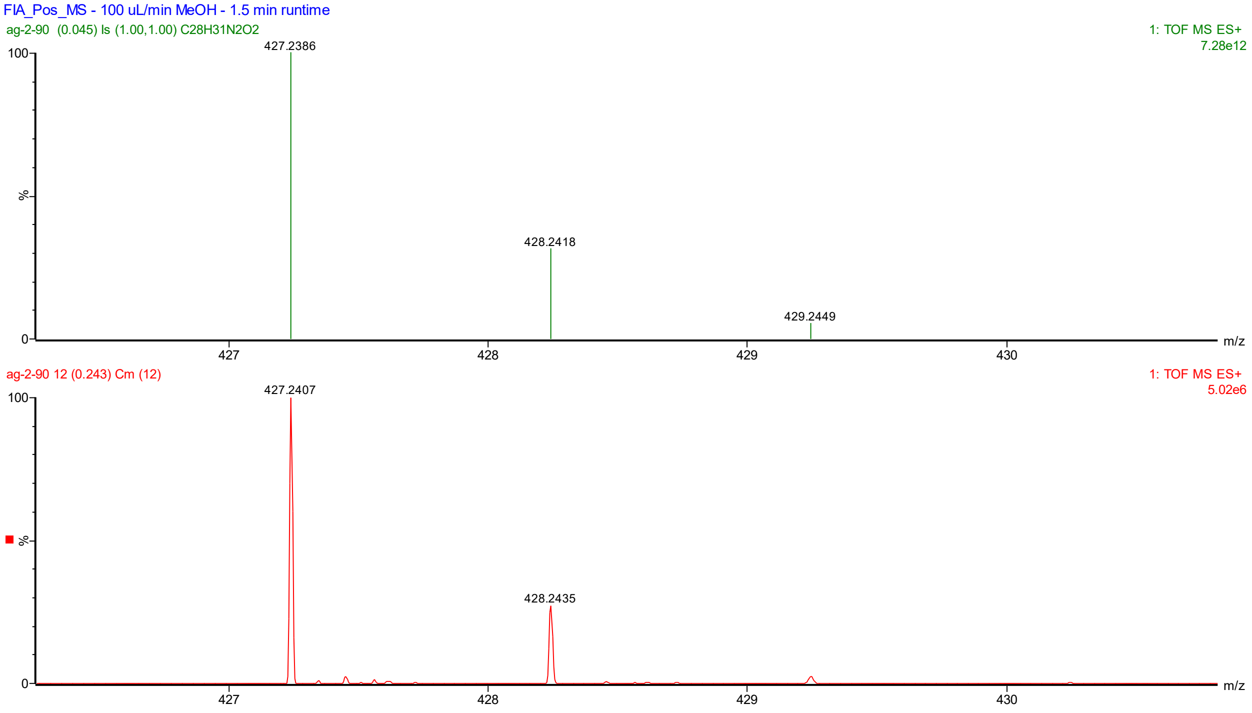

Figure S39. HRMS analysis (ESI, CH_3OH) report of **4b**

Compound 4d

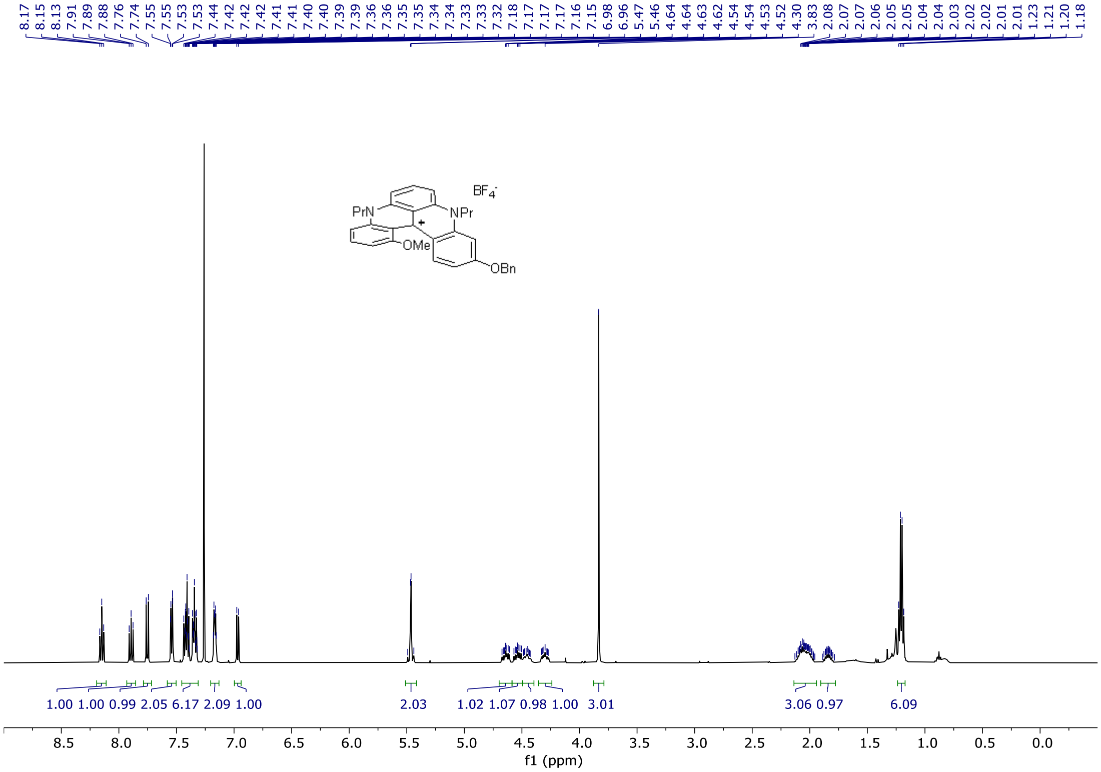

Figure S40. ^{1}H NMR (500 MHz, $CDCl_3$) spectrum of **4d**

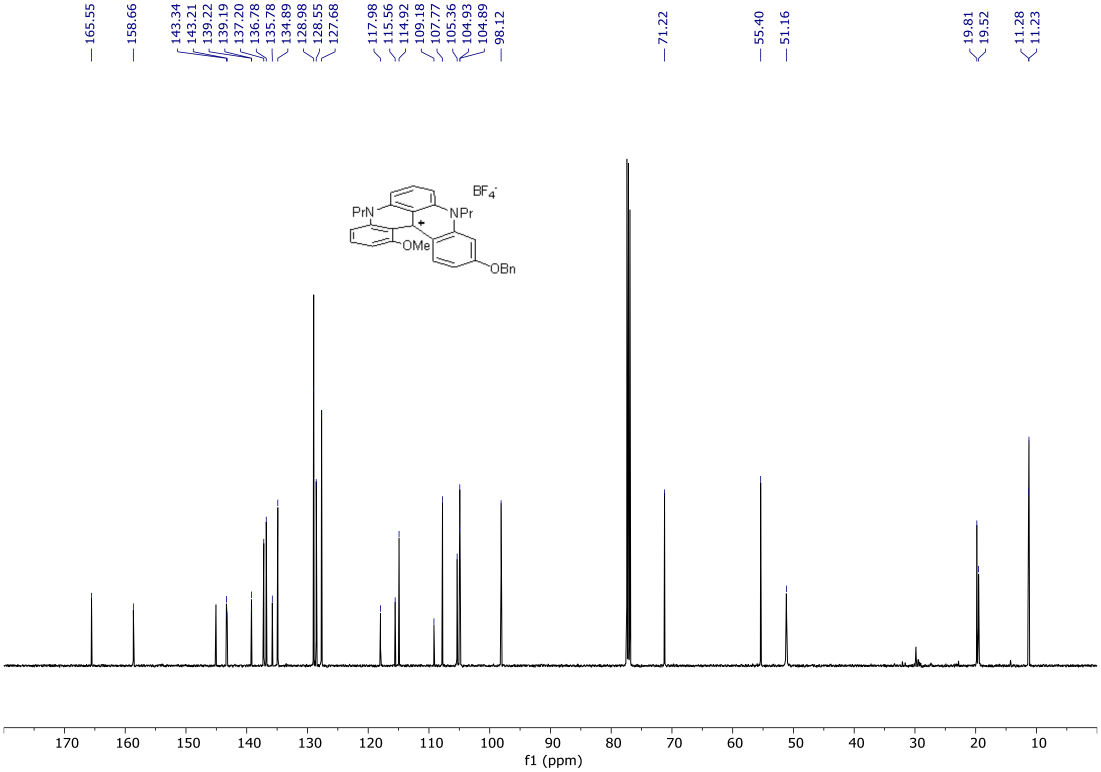

Figure S41. ^{13}C NMR (126 MHz, $CDCl_3$) spectrum of **4d**

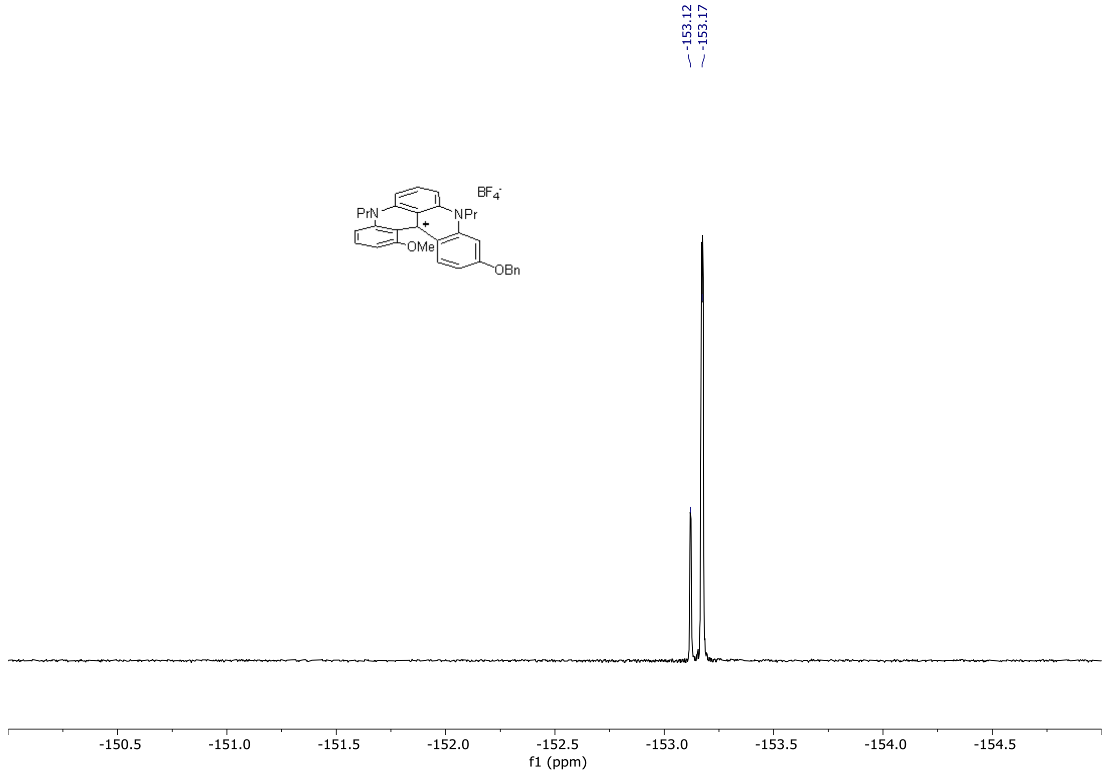

Figure S42. ^{19}F NMR (282 MHz, $CDCl_3$) spectrum of **4d**

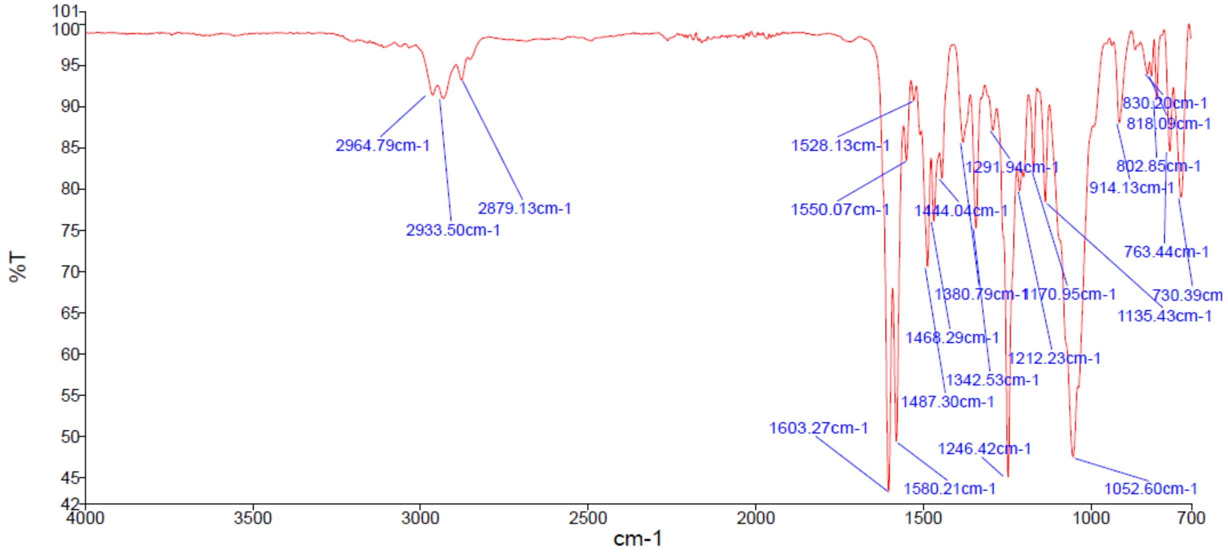

Figure S43. IR (neat) spectrum of **4d**

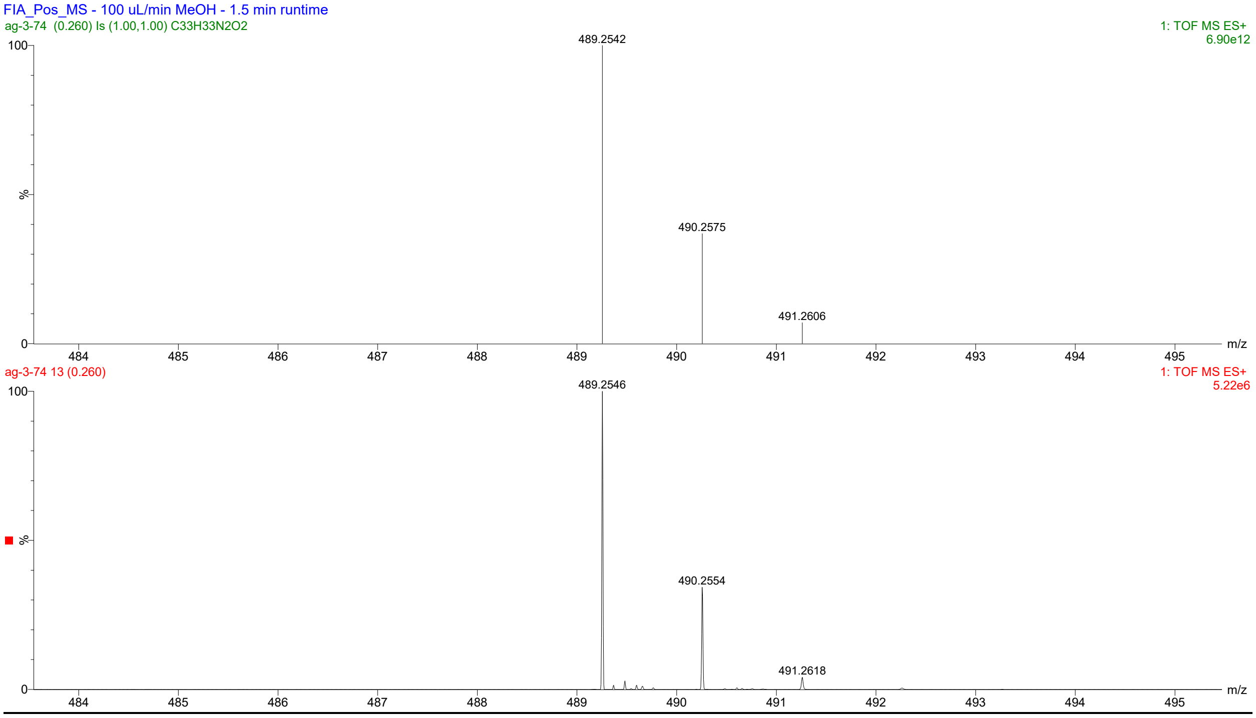

Figure S44. HRMS analysis (ESI, CH_3OH) report of **4d**

Compound (*S*)-4e

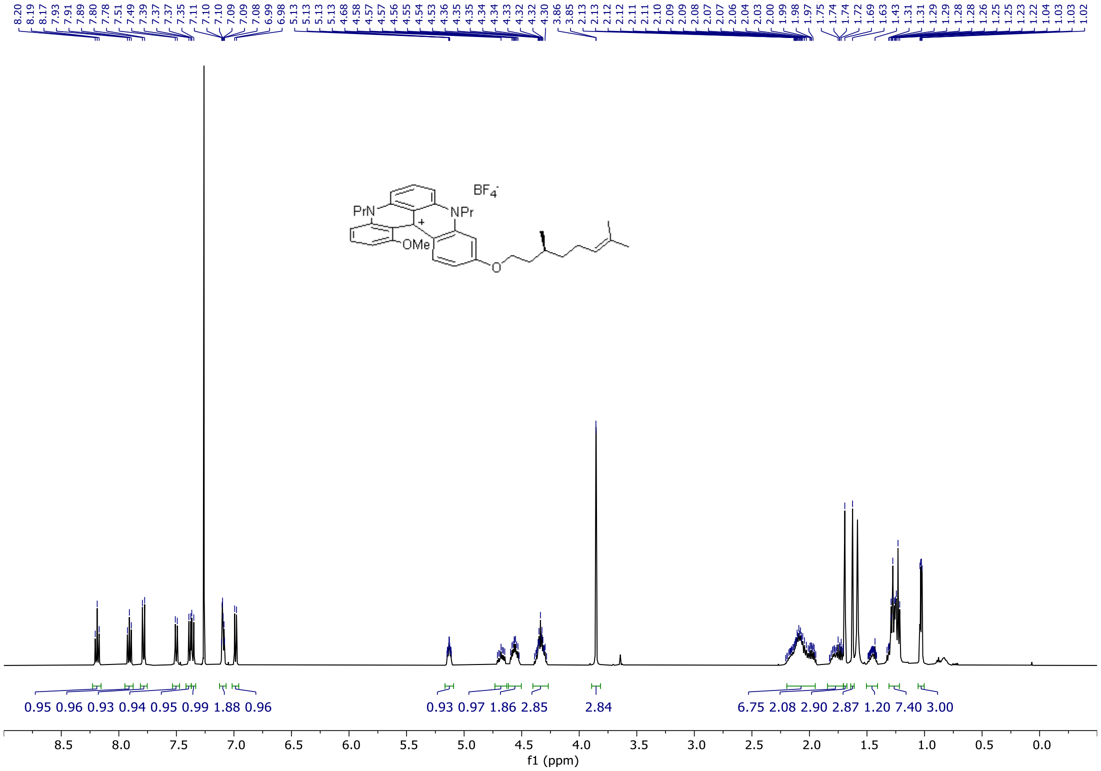

Figure S45. ^{1}H NMR (500 MHz, $CDCl_3$) spectrum of (*S*)-**4e**

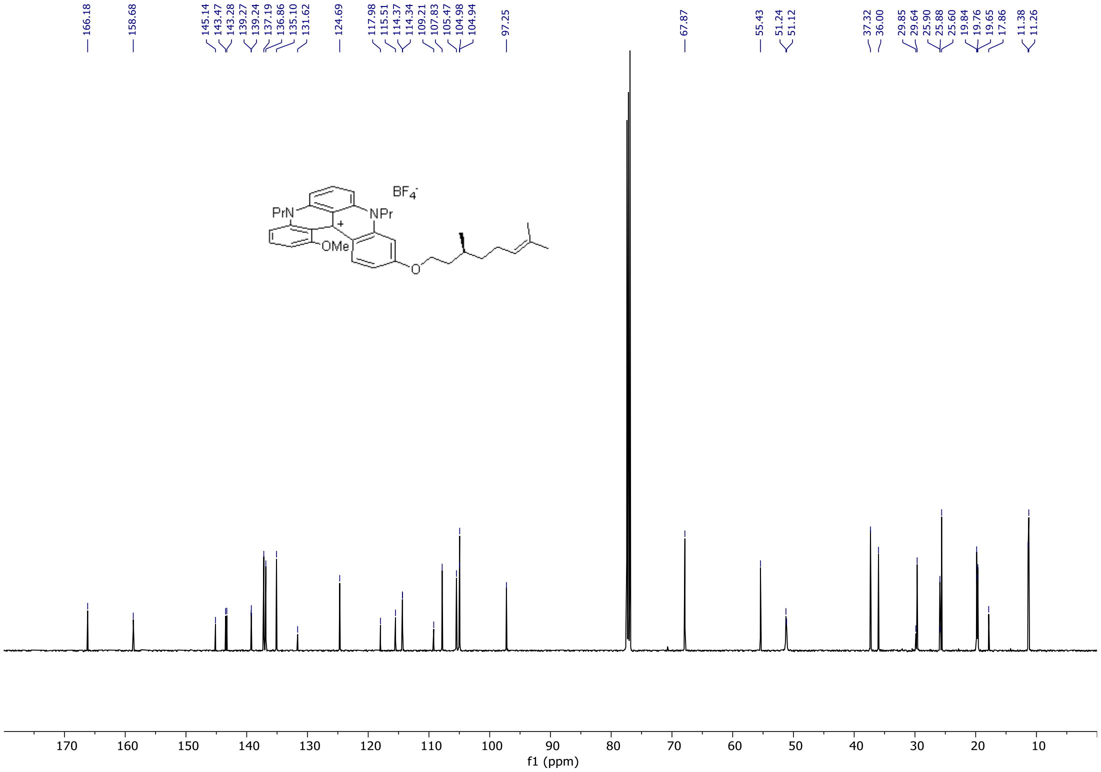

Figure S46. ^{13}C NMR (126 MHz, $CDCl_3$) spectrum of (*S*)-**4e**

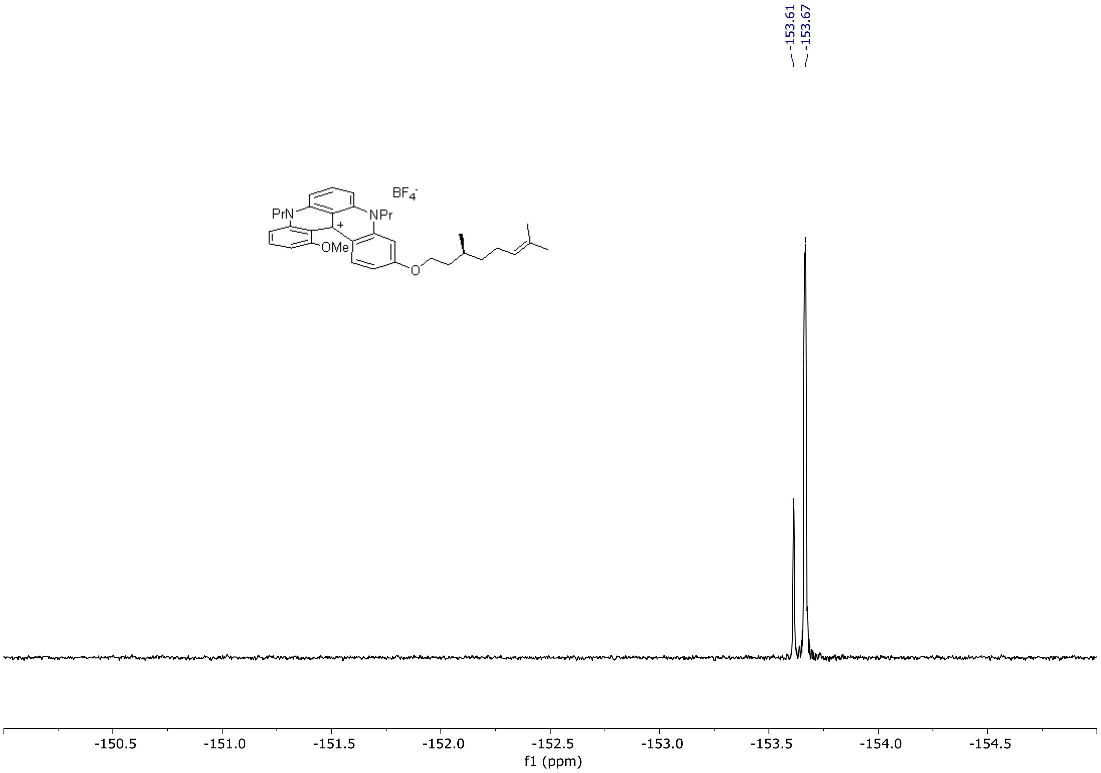

Figure S47. ^{19}F NMR (282 MHz, $CDCl_3$) spectrum of (*S*)-**4e**

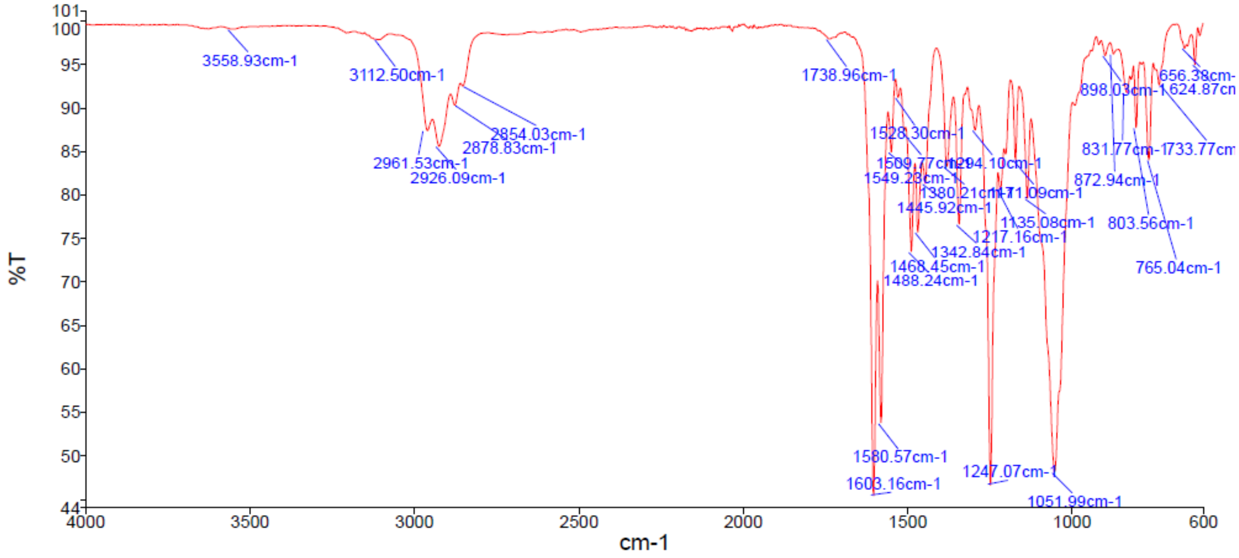

Figure S48. IR (neat) spectrum of (*S*)-**4e**

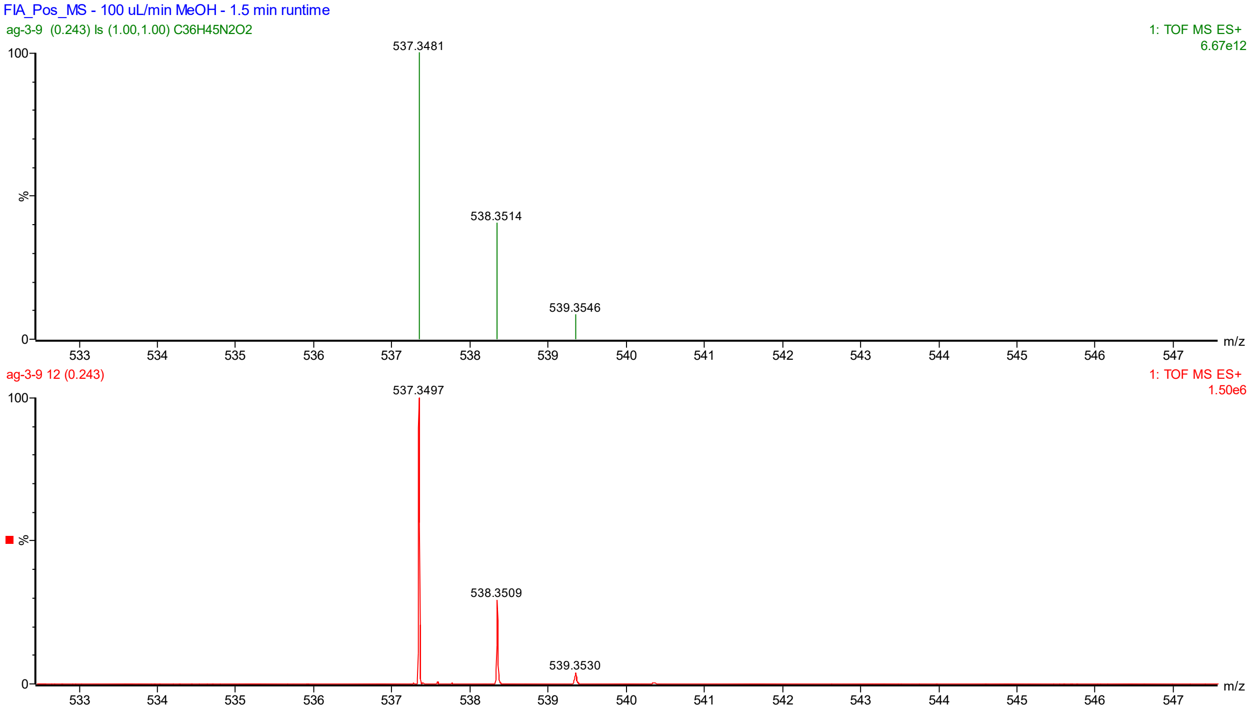

Figure S49. HRMS analysis (ESI, CH_3OH) report of (*S*)-**4e**

Compound 5a

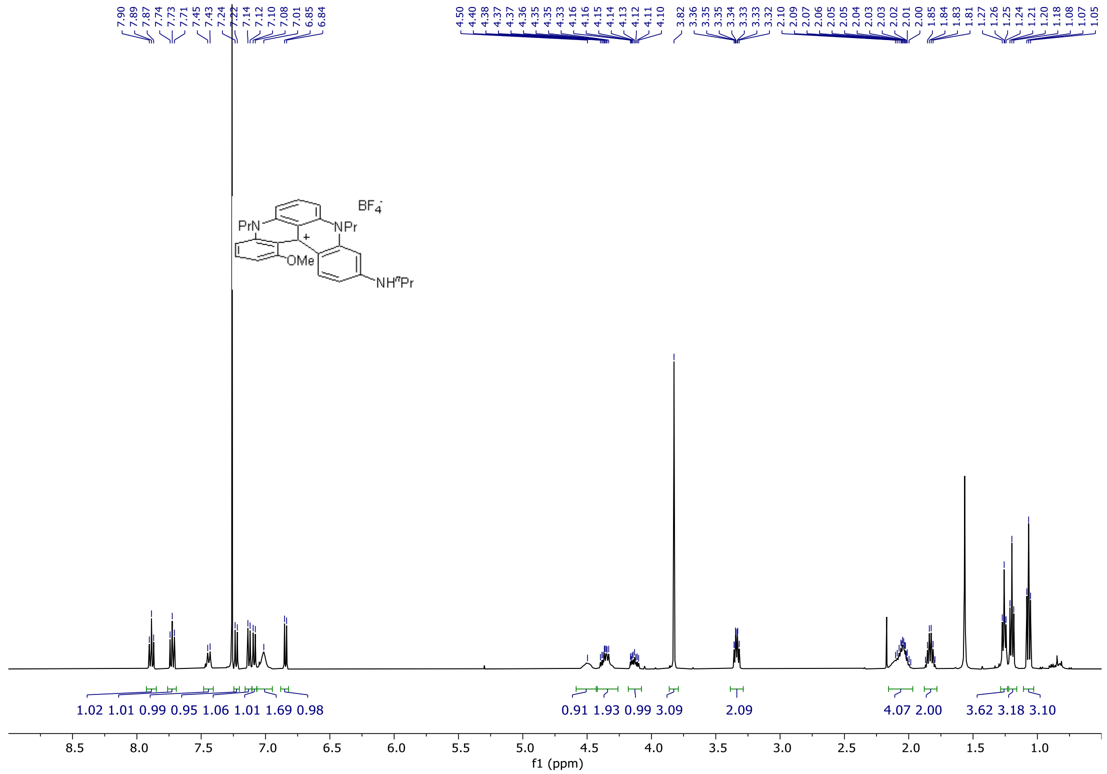

Figure S50. ^{1}H NMR (500 MHz, $CDCl_3$) spectrum of **5a**

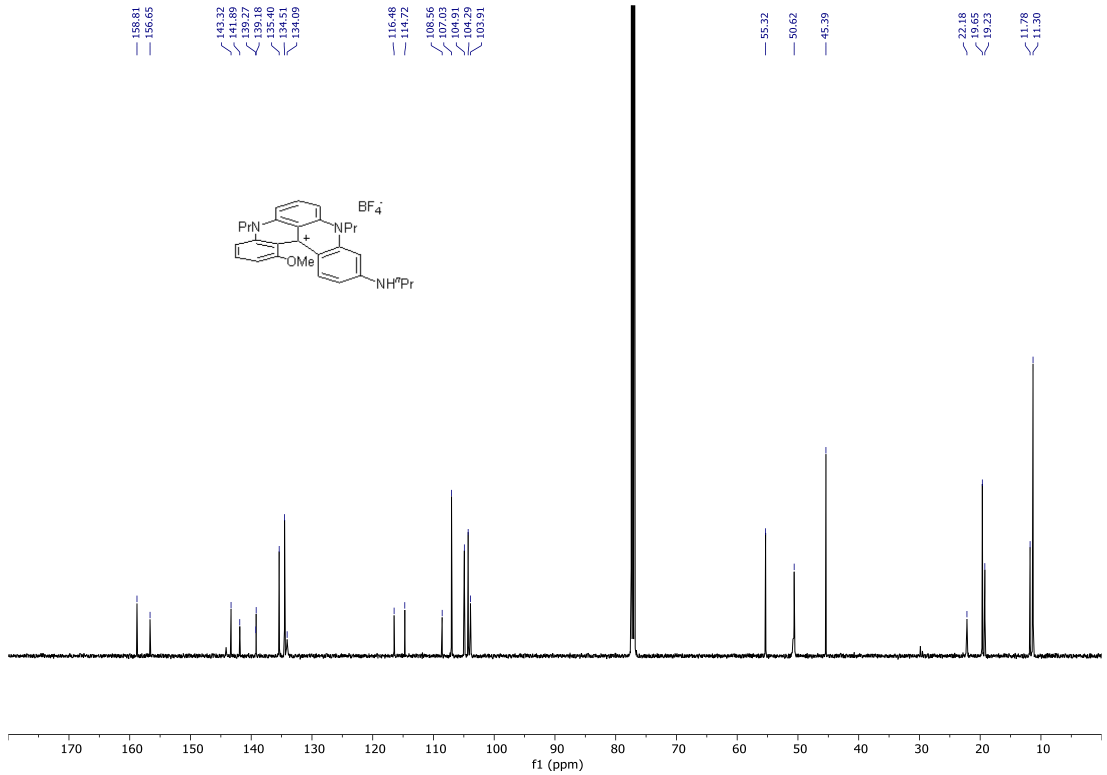

Figure S51. ^{13}C NMR (126 MHz, $CDCl_3$) spectrum of **5a**

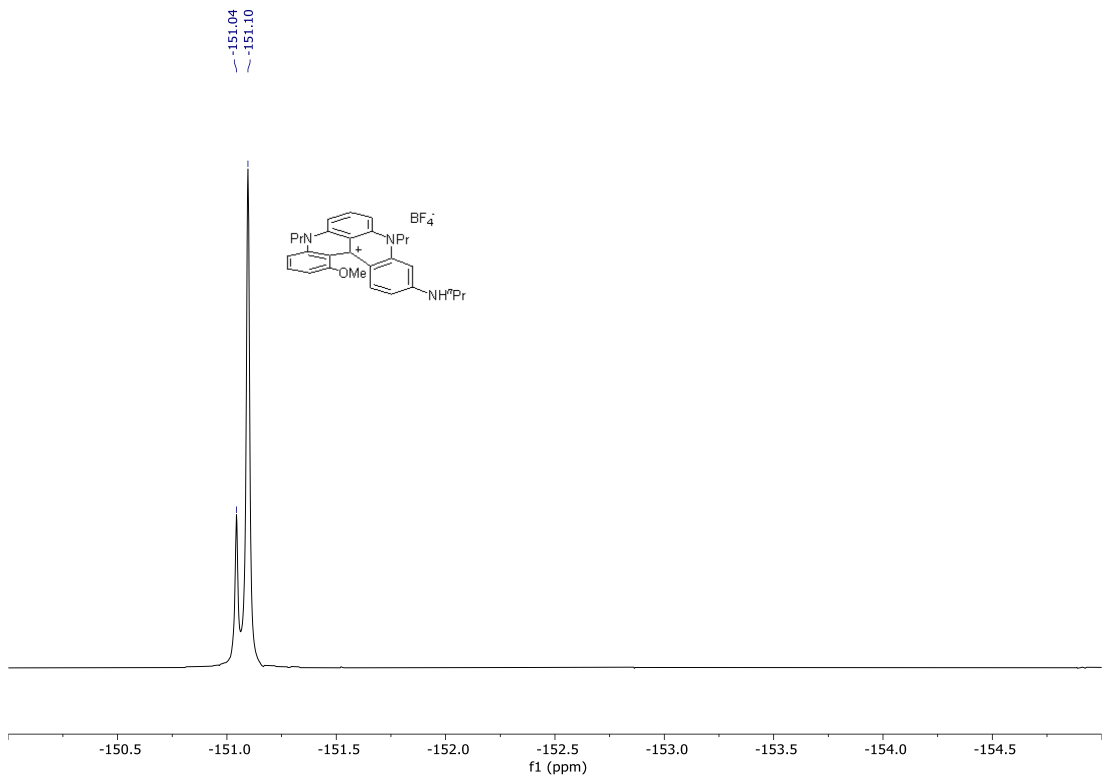

Figure S52. ^{19}F NMR (282 MHz, $CDCl_3$) spectrum of **5a**

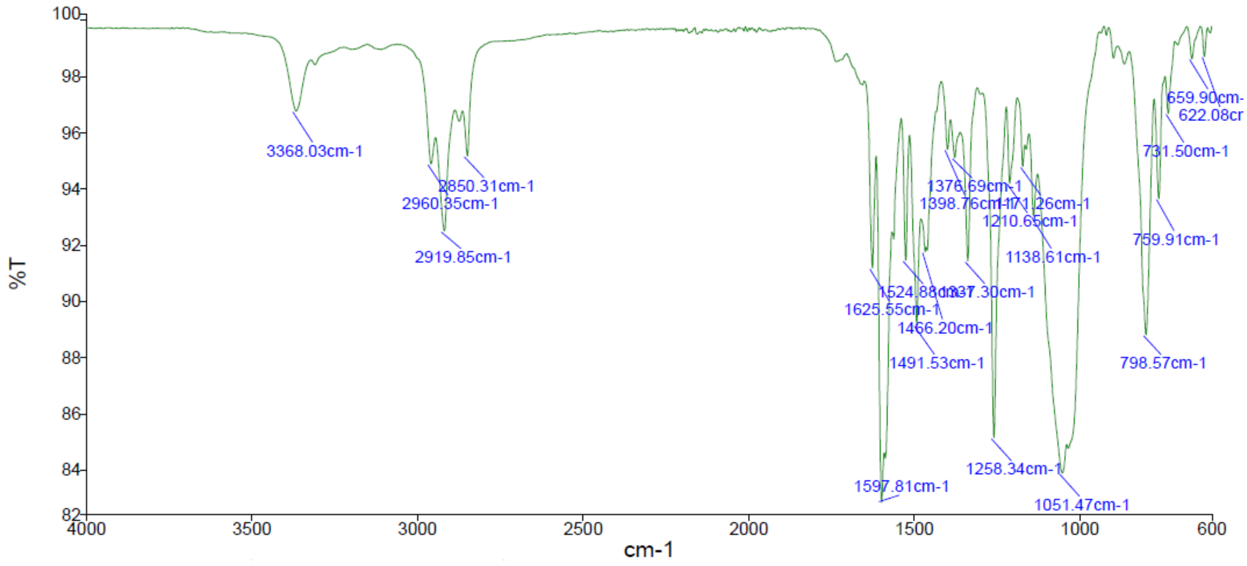

Figure S53. IR (neat) spectrum of **5a**

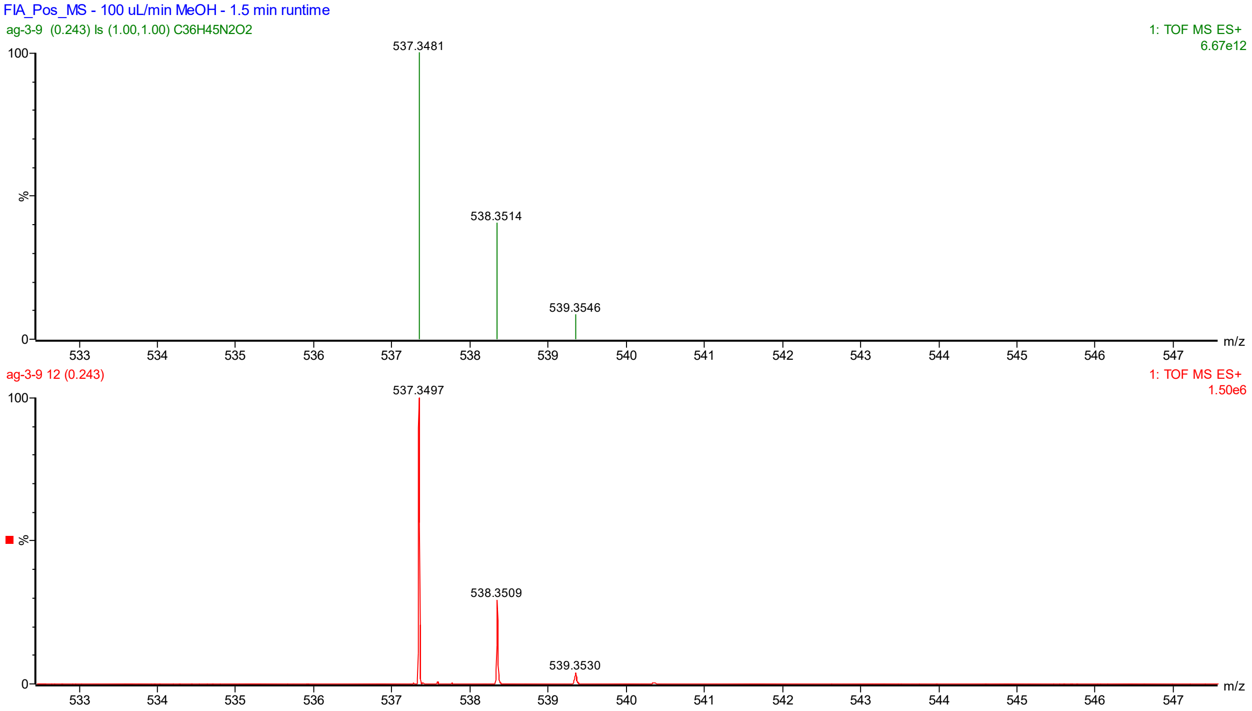

Figure S54. HRMS analysis (ESI, CH_3OH) report of **5a**

Compound 5b

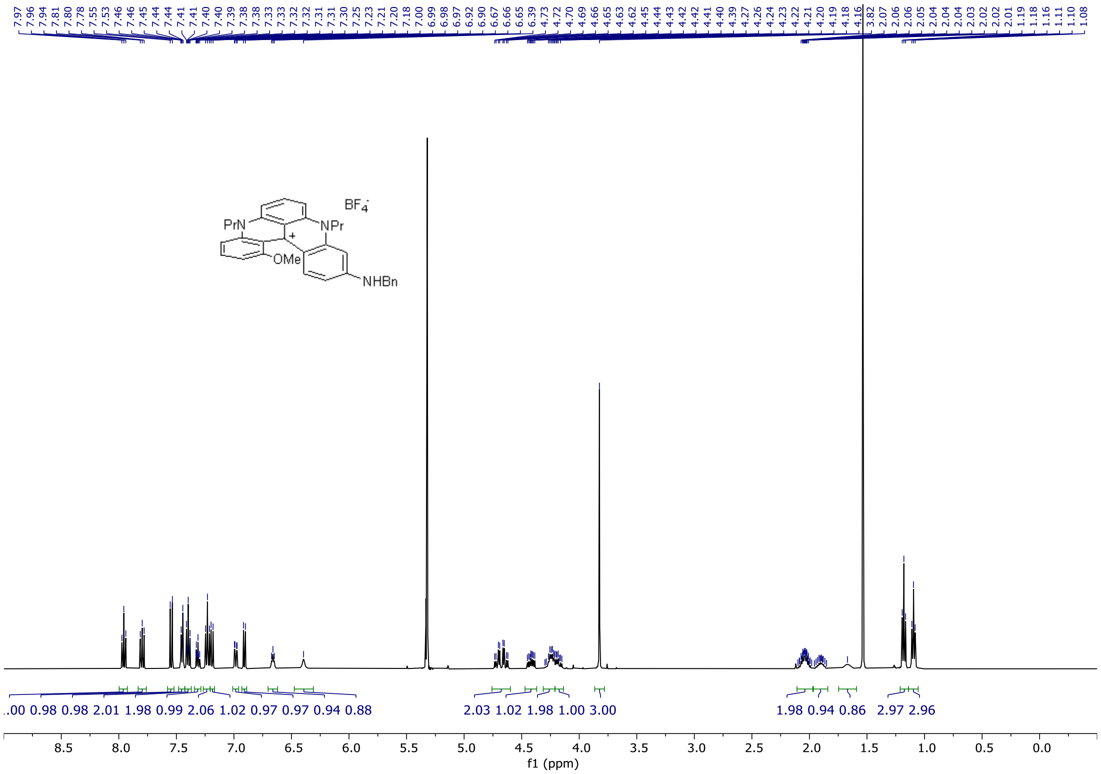

Figure S55. ^1H NMR (500 MHz, CD_2Cl_2) spectrum of **5b**

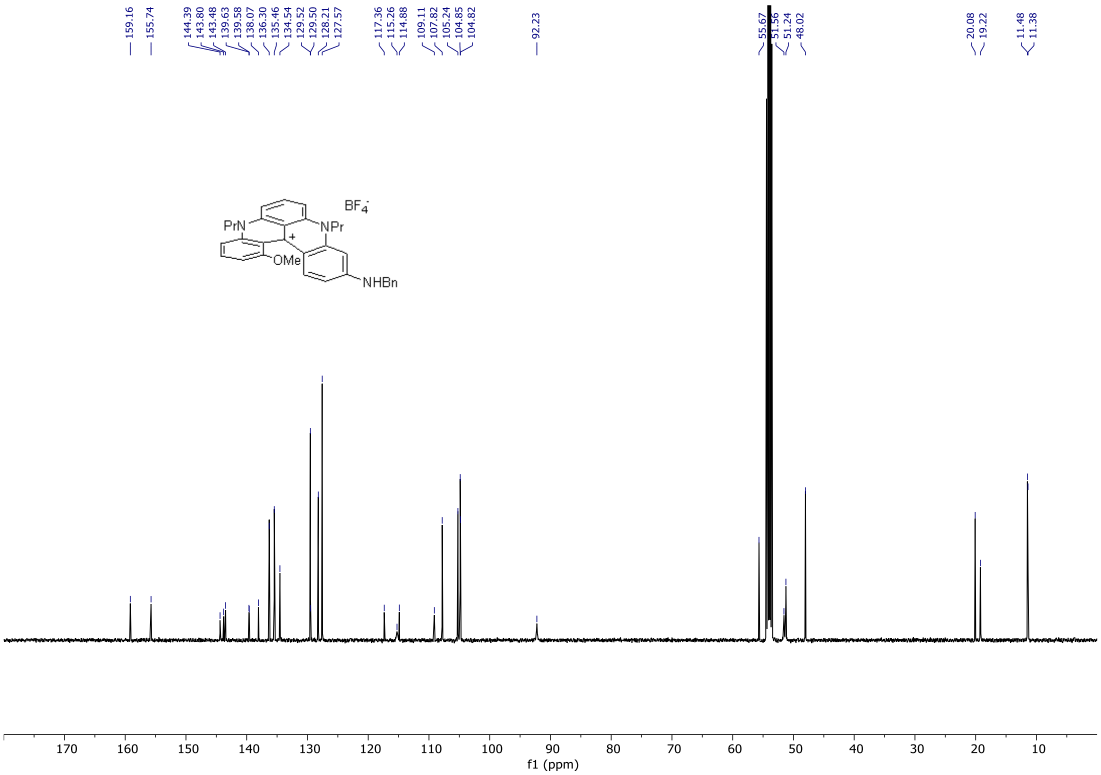

Figure S56. ^{13}C NMR (126 MHz, CD_2Cl_2) spectrum of **5b**

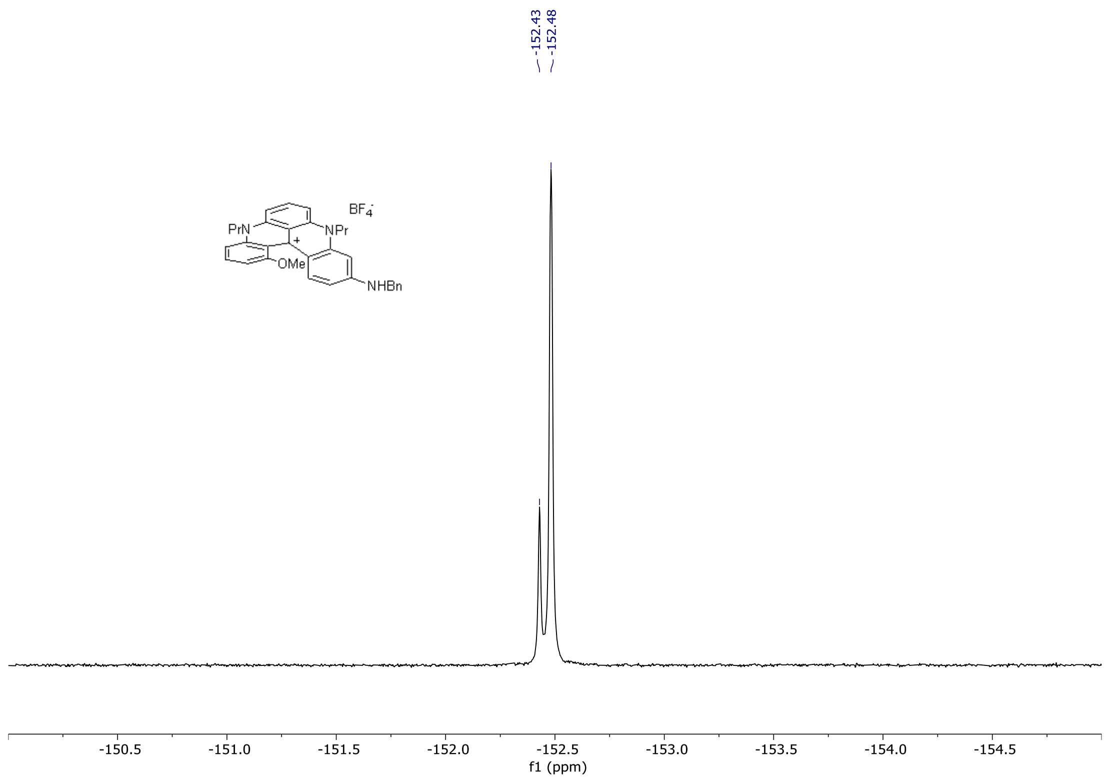

Figure S57. ^{19}F NMR (282 MHz, CD_2Cl_2) spectrum of **5b**

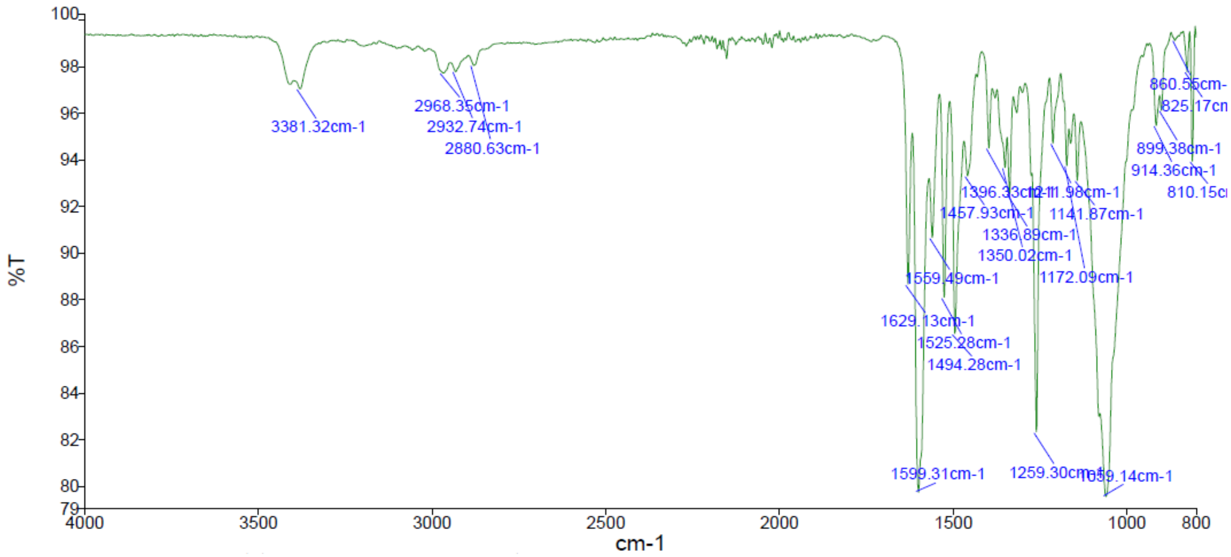

Figure S58. IR (neat) spectrum of **5b**

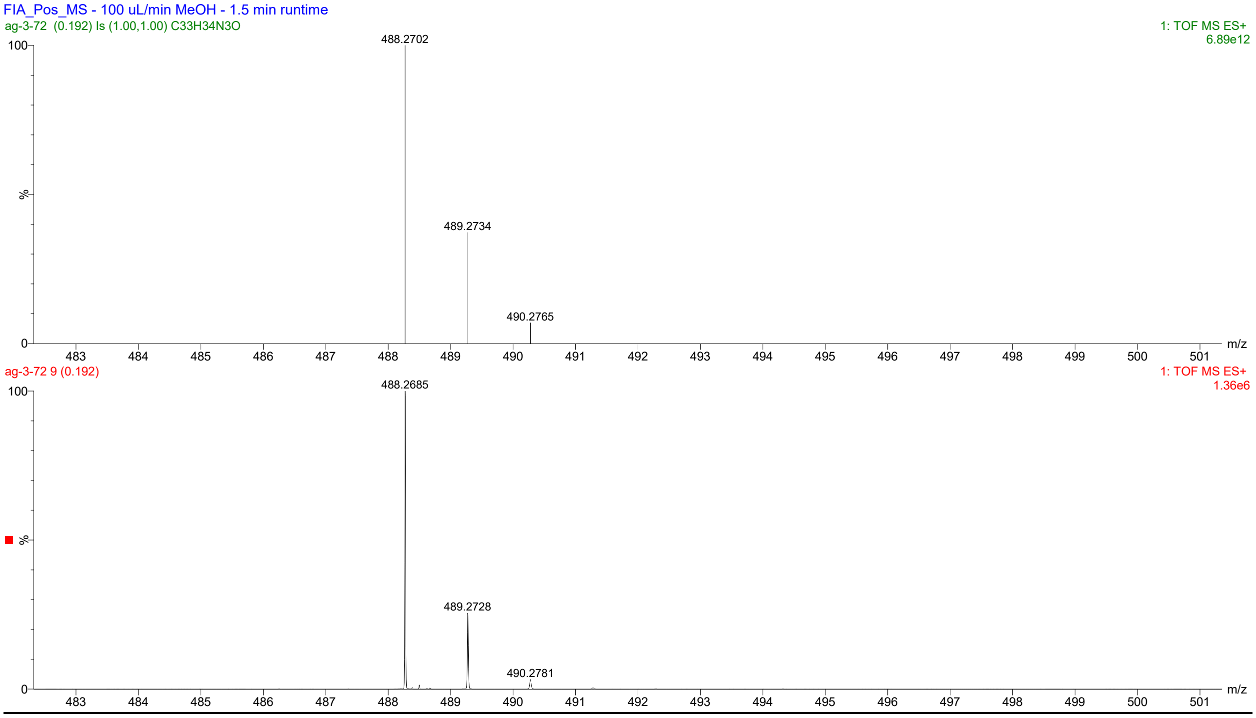

Figure S59. HRMS analysis (ESI, CH_3OH) report of **5b**

Compound 5c

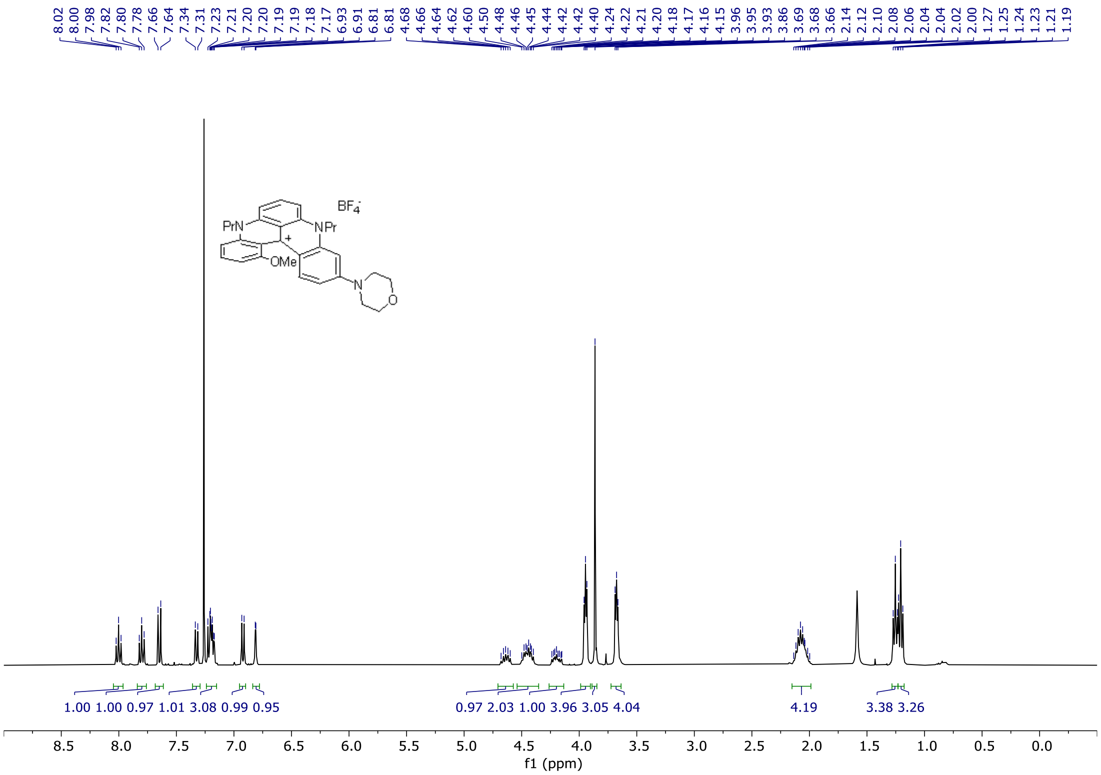

Figure S60. ^{1}H NMR (400 MHz, $CDCl_3$) spectrum of **5c**

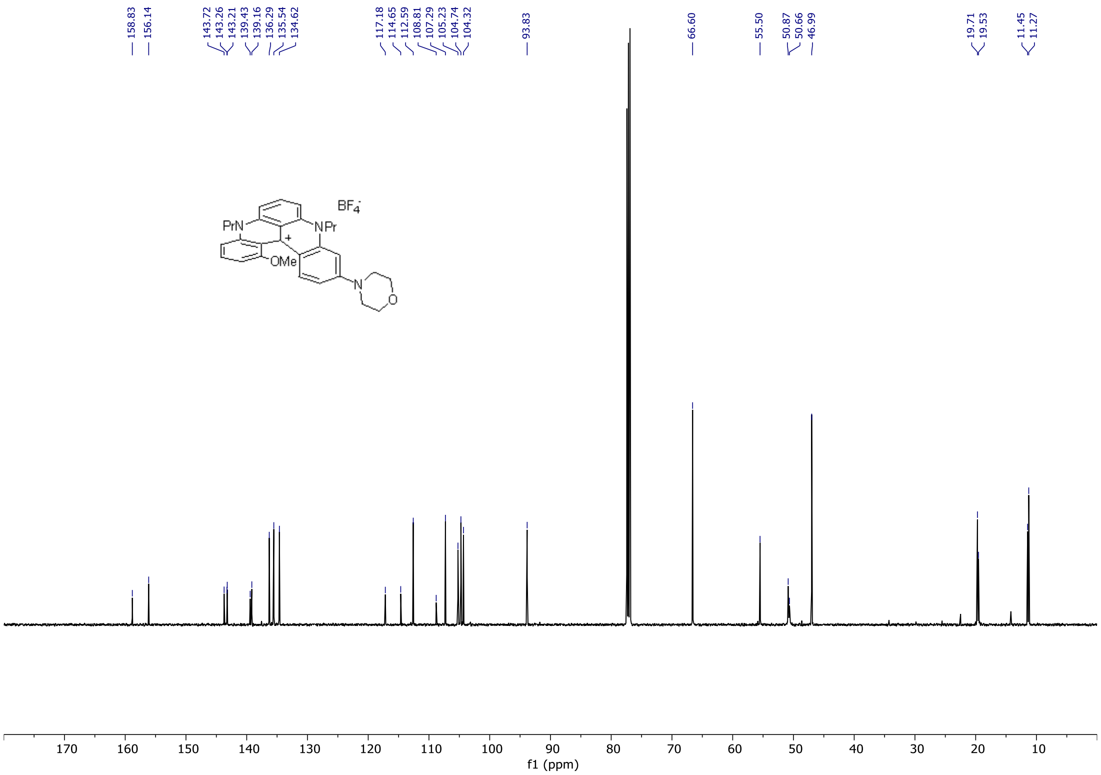

Figure S61. ^{13}C NMR (126 MHz, $CDCl_3$) spectrum of **5c**

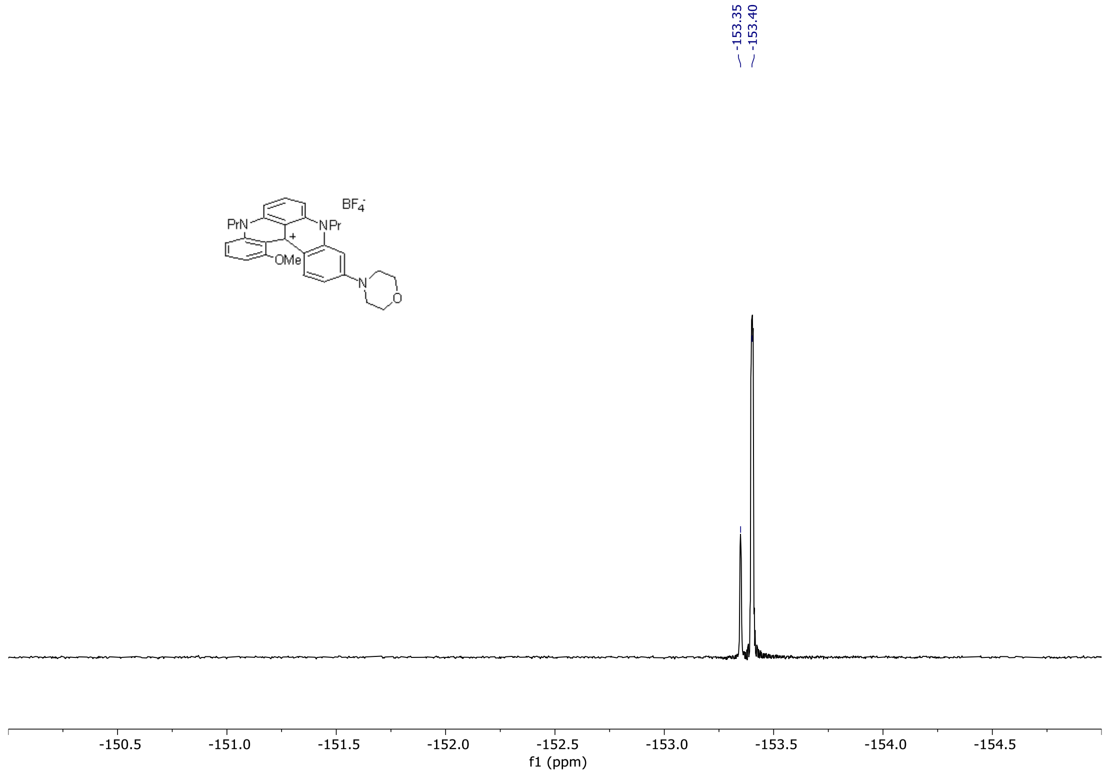

Figure S62. ^{19}F NMR (282 MHz, $CDCl_3$) spectrum of **5c**

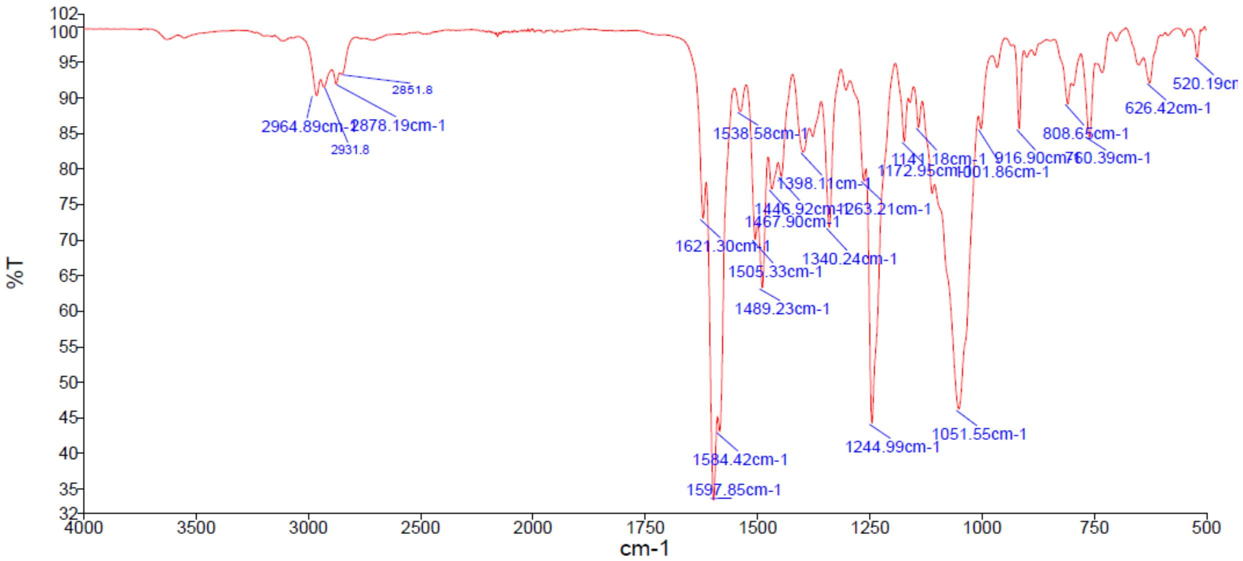

Figure S63. IR (neat) spectrum of **5c**

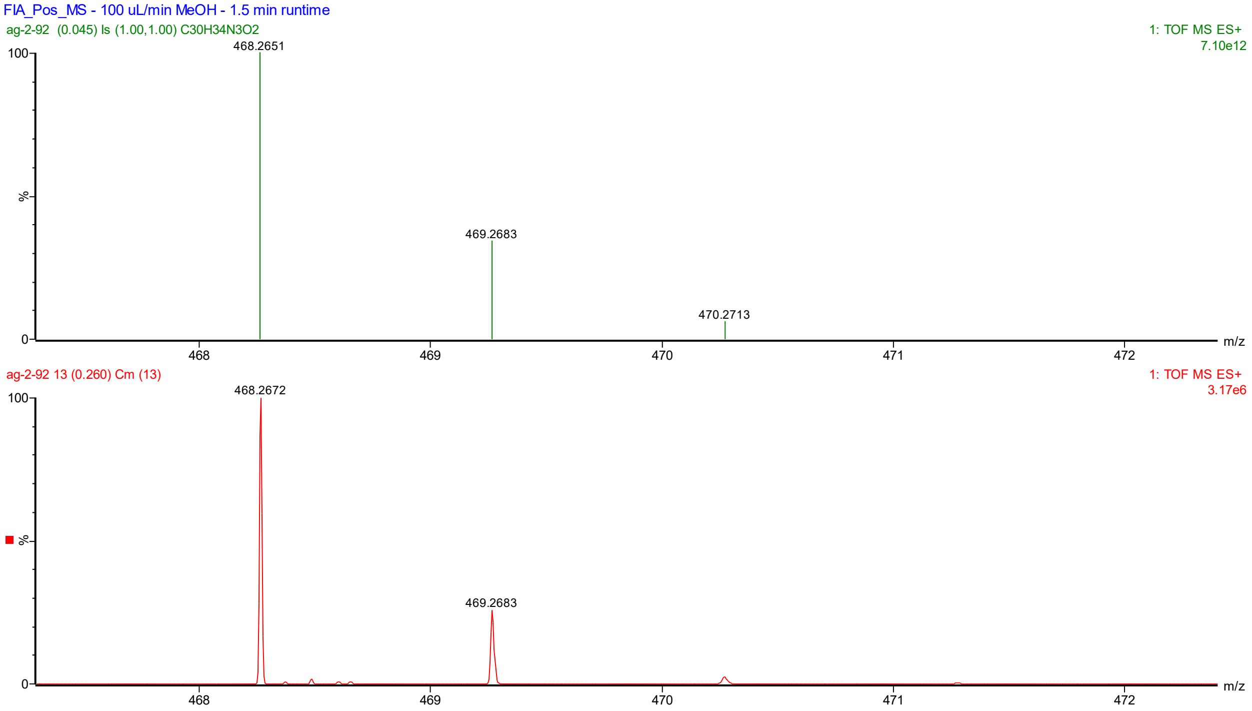

Figure S64. HRMS analysis (ESI, CH_3OH) report of **5c**

Compound 5d

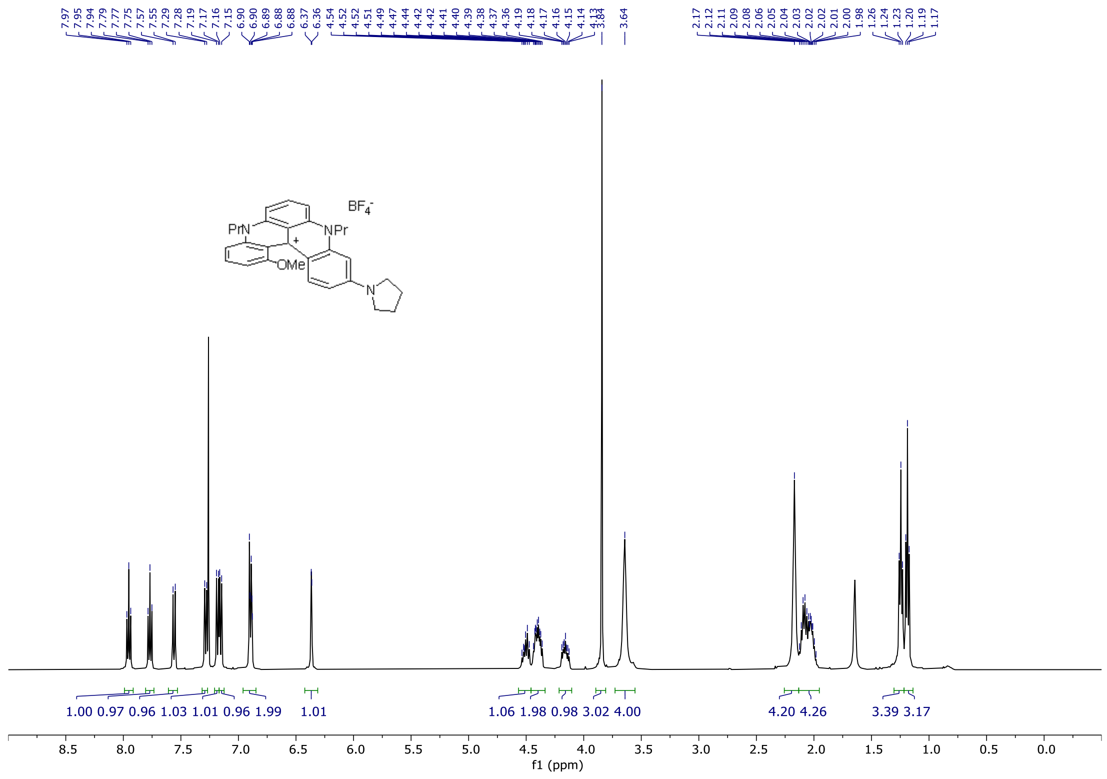

Figure S65. ^{1}H NMR (500 MHz, $CDCl_3$) spectrum of **5d**

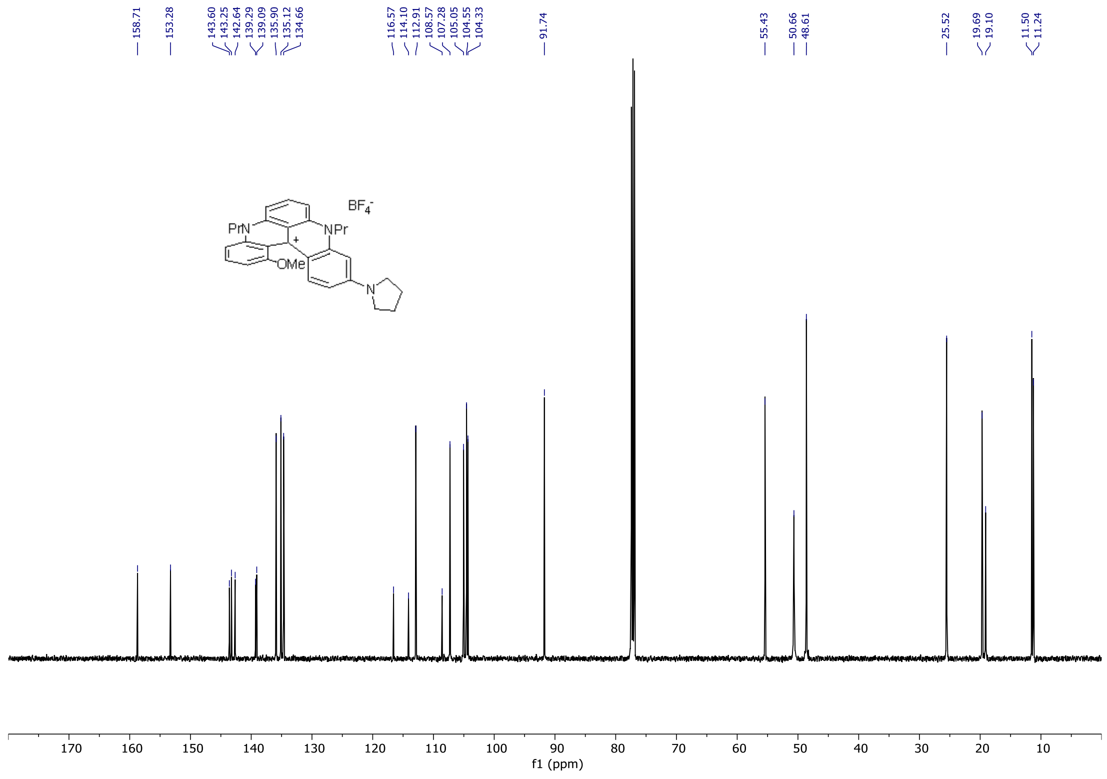

Figure S66. ^{13}C NMR (126 MHz, $CDCl_3$) spectrum of **5d**

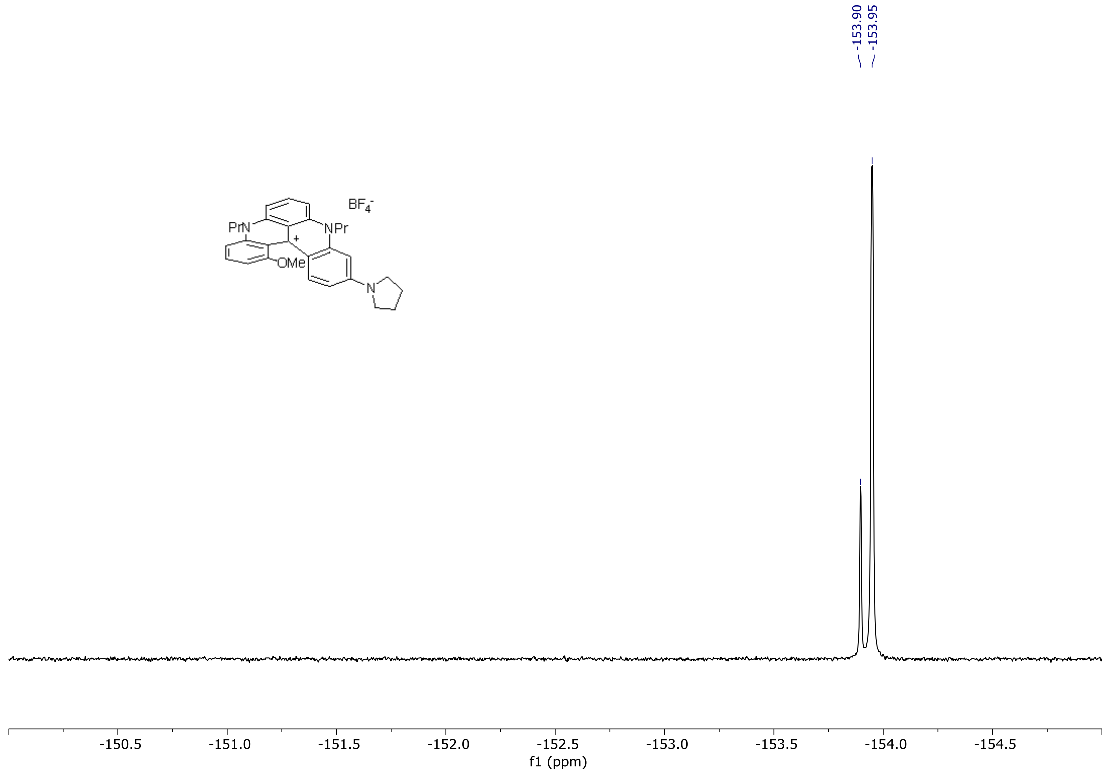

Figure S67. ^{19}F NMR (282 MHz, $CDCl_3$) spectrum of **5d**

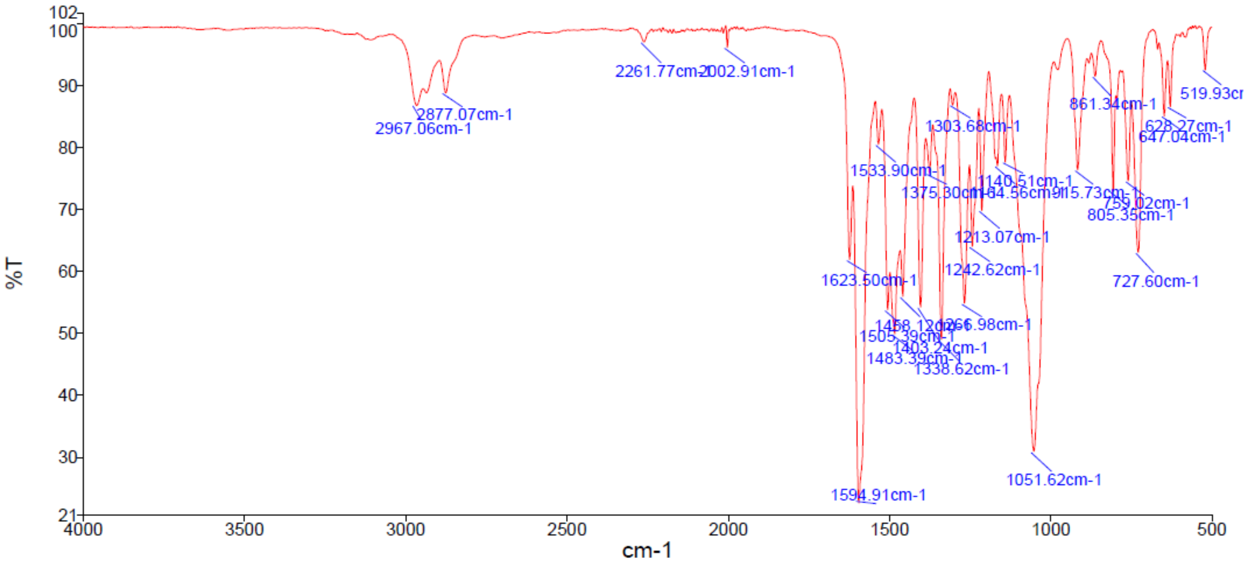

Figure S68. IR (neat) spectrum of **5d**

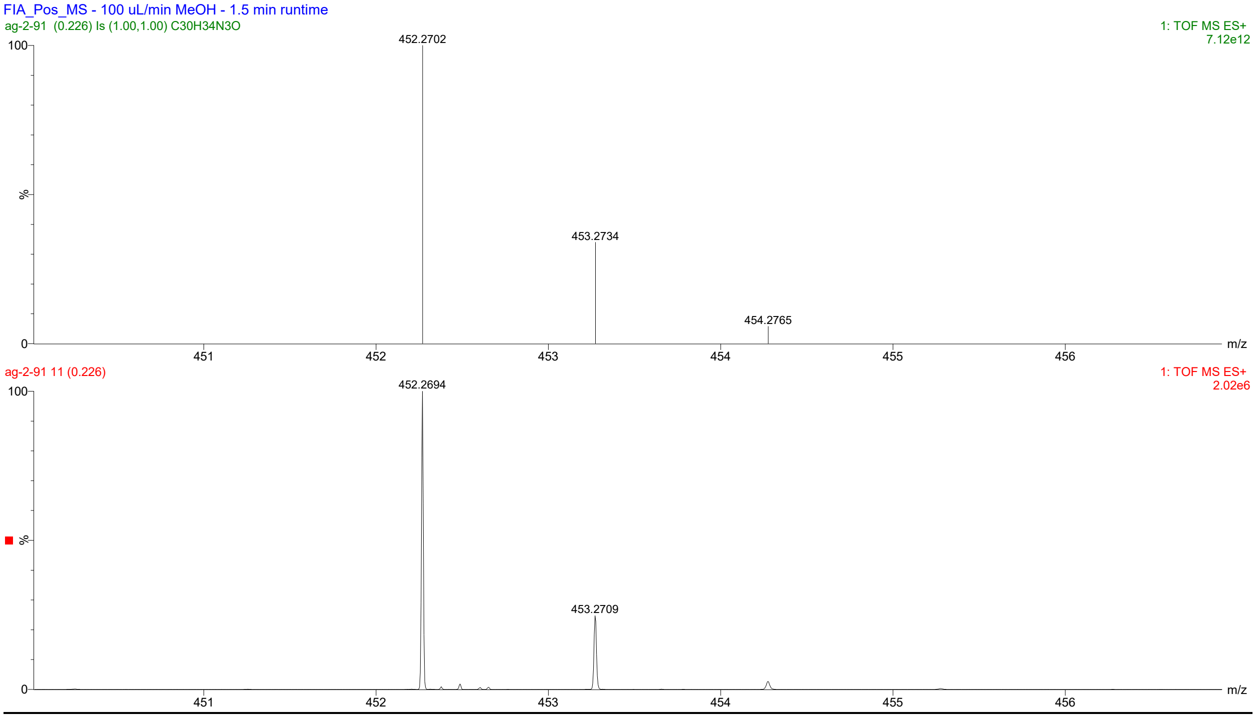

Figure S69. HRMS analysis (ESI, CH_3OH) report of **5d**

Compound 5e

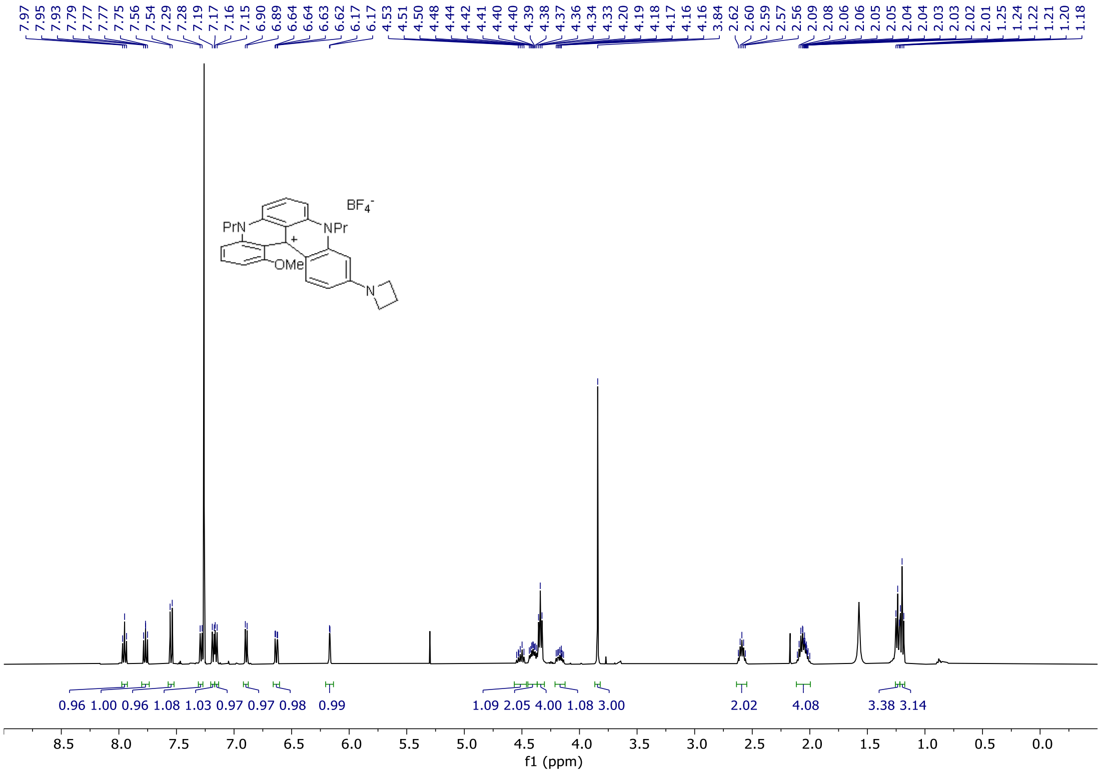

Figure S70. ^{1}H NMR (500 MHz, $CDCl_3$) spectrum of **5e**

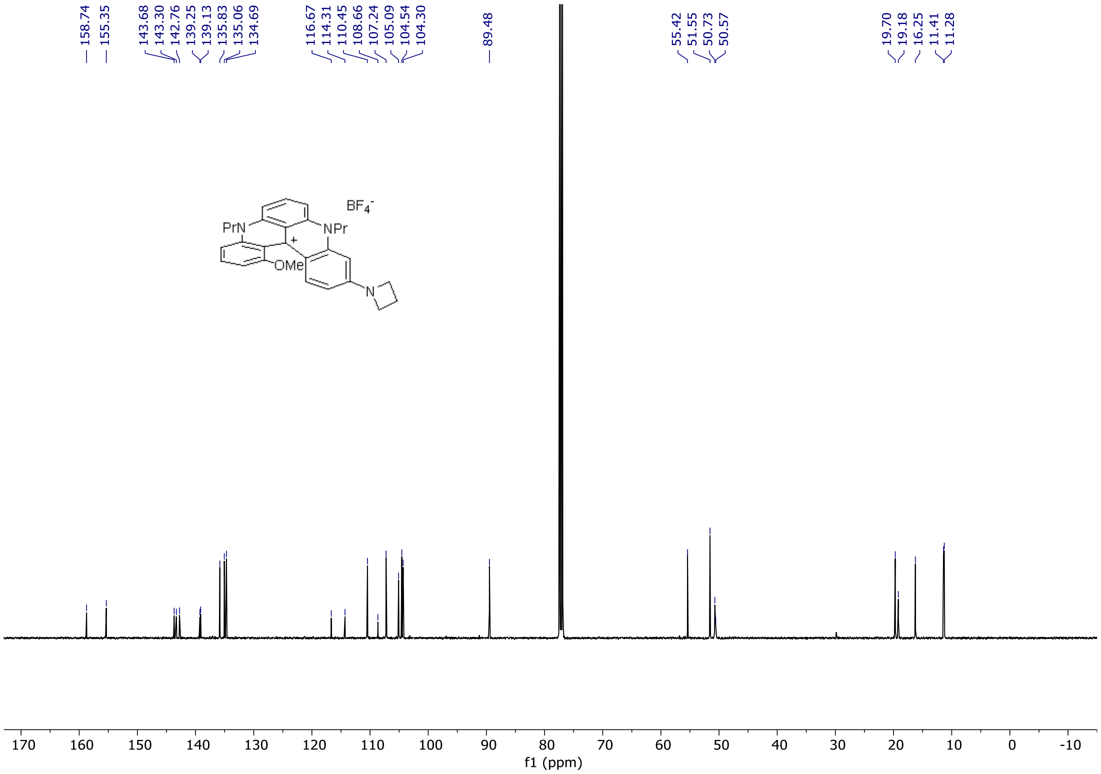

Figure S71. ^{13}C NMR (126 MHz, $CDCl_3$) spectrum of **5e**

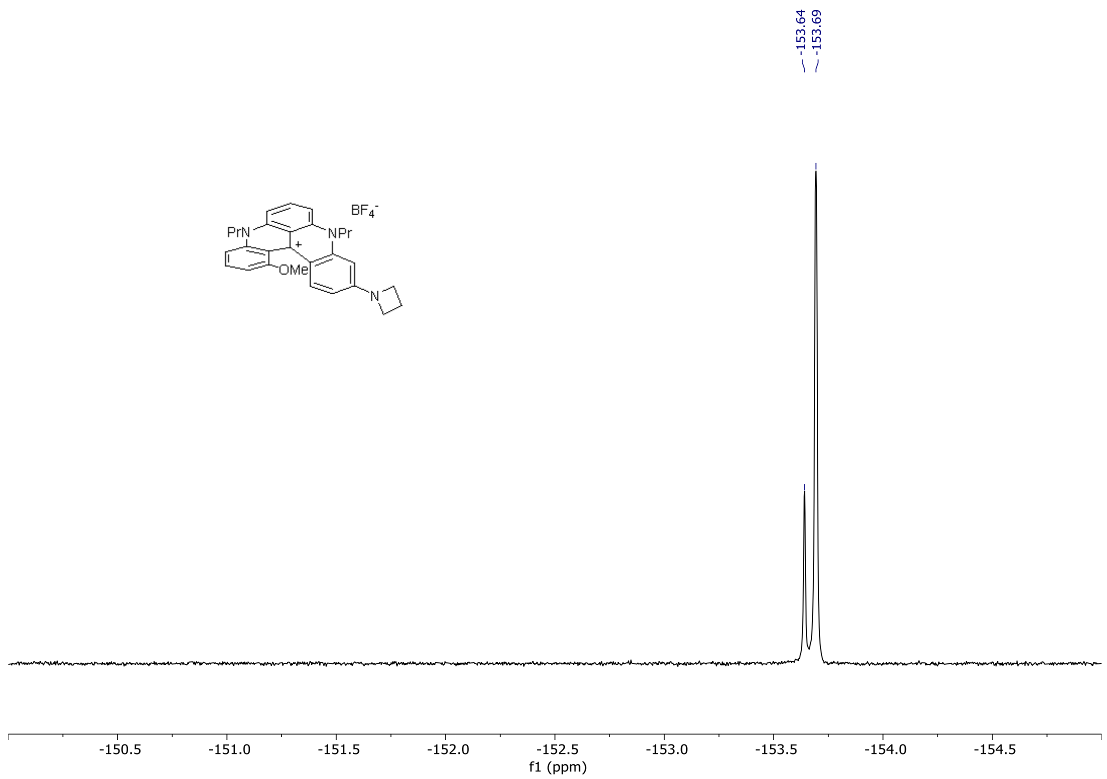

Figure S72. ^{19}F NMR (282 MHz, $CDCl_3$) spectrum of **5e**

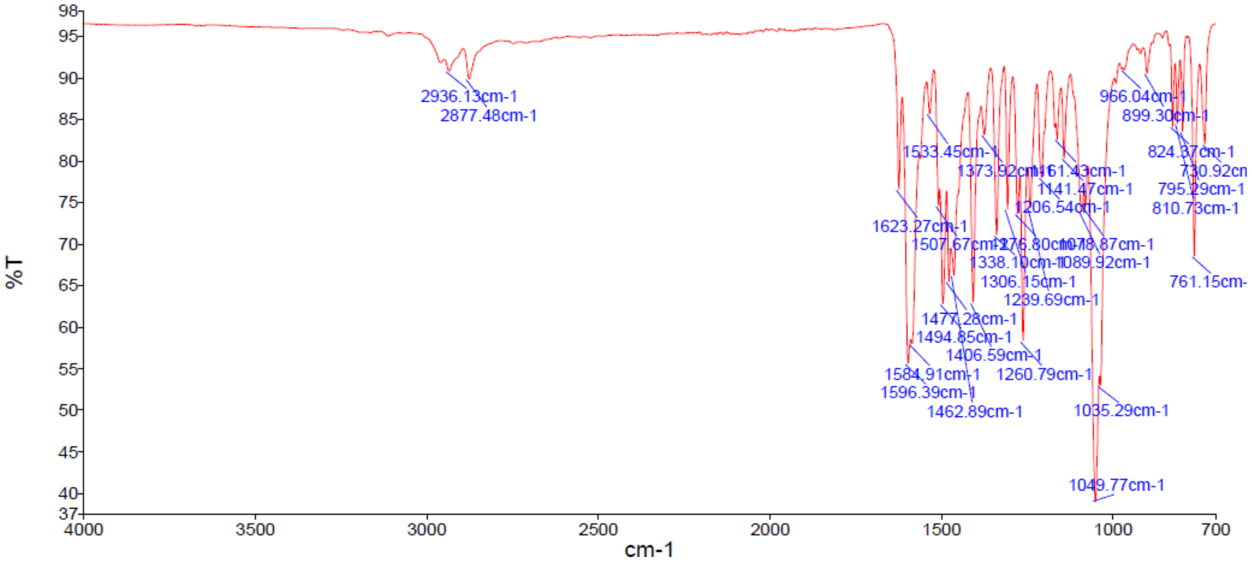

Figure S73. IR (neat) spectrum of **5e**

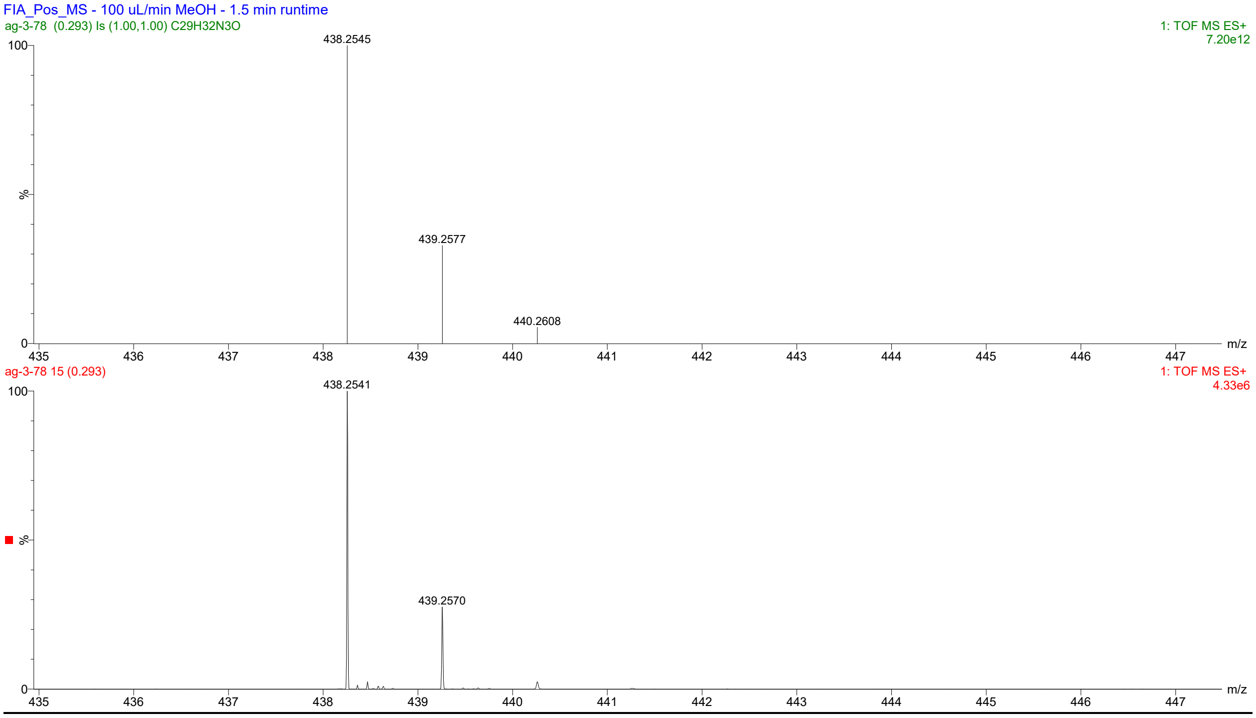

Figure S74. HRMS analysis (ESI, CH_3OH) report of **5e**

Compound (*R*)-5f

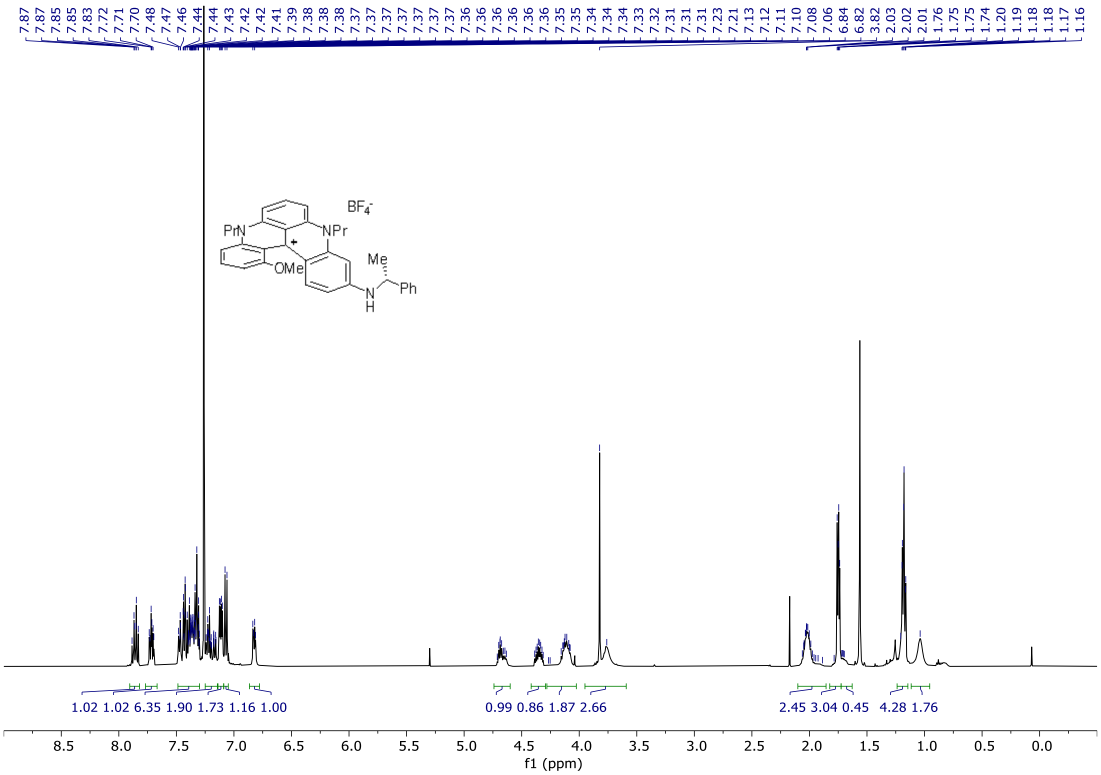

Figure S75. ^{1}H NMR (500 MHz, $CDCl_3$) spectrum of (*R*)-**5f**

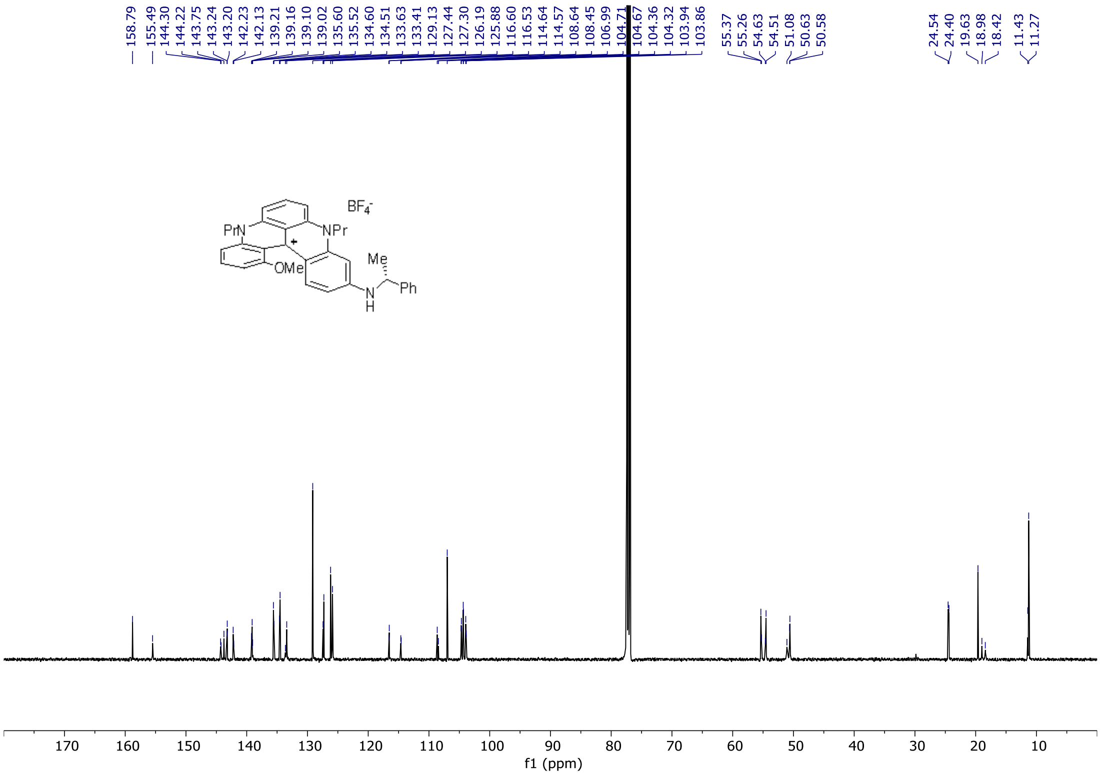

Figure S76. ^{13}C NMR (126 MHz, $CDCl_3$) spectrum of (*R*)-**5f**

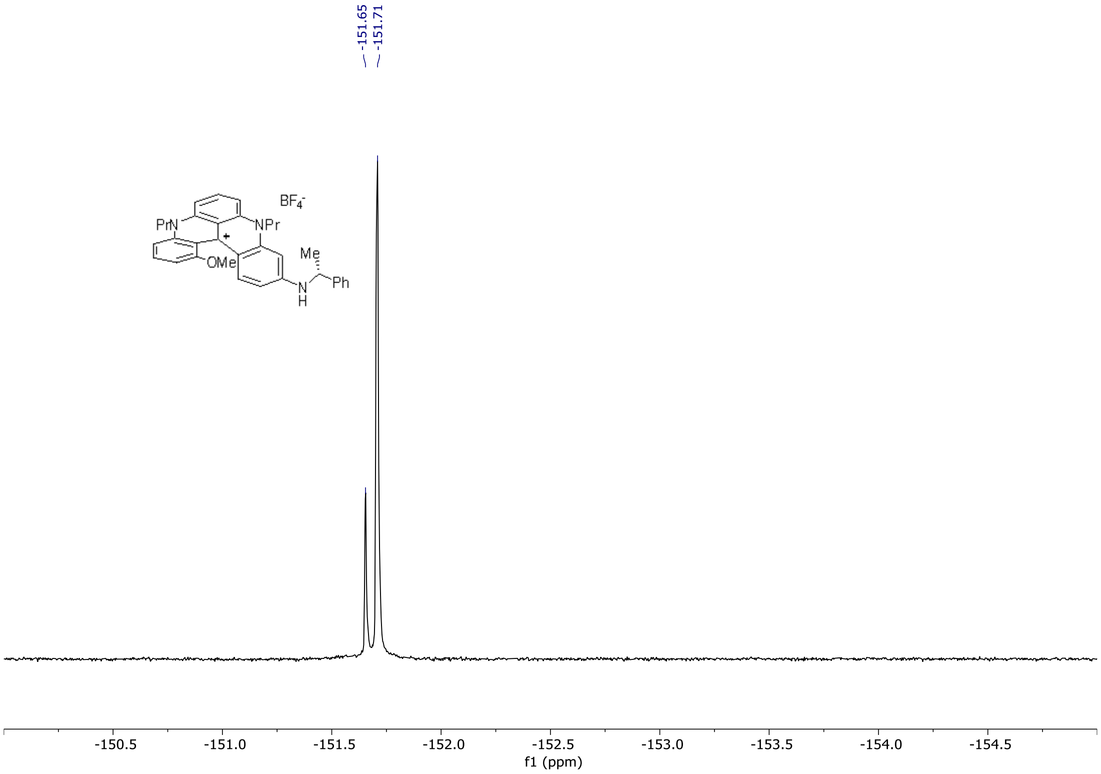

Figure S77. ^{19}F NMR (282 MHz, $CDCl_3$) spectrum of (*R*)-**5f**

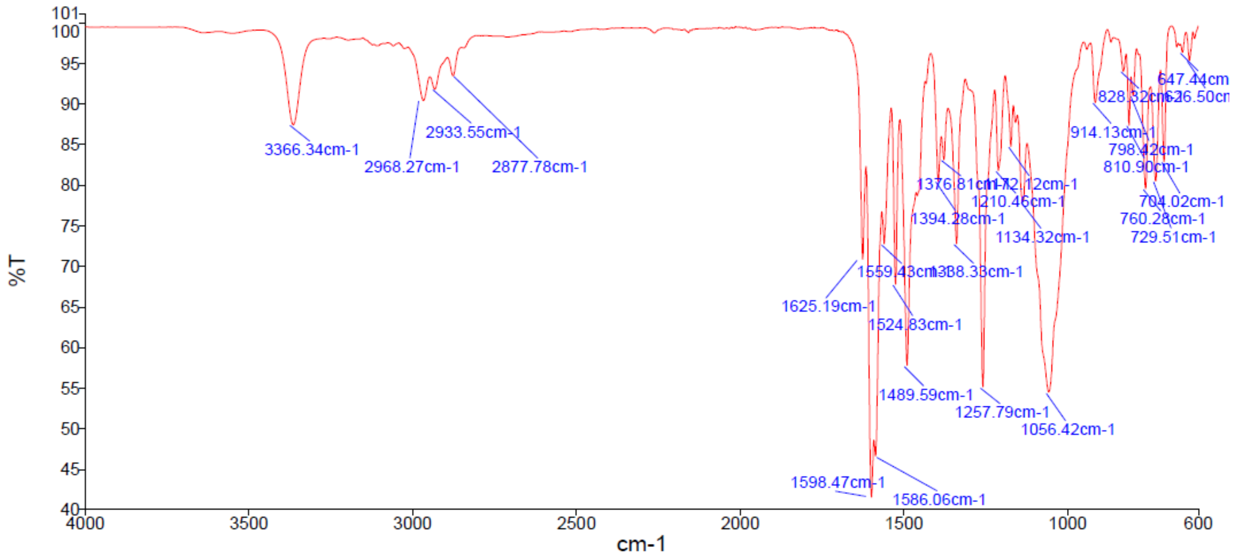

Figure S78. IR (neat) spectrum of (*S*)-**5f**

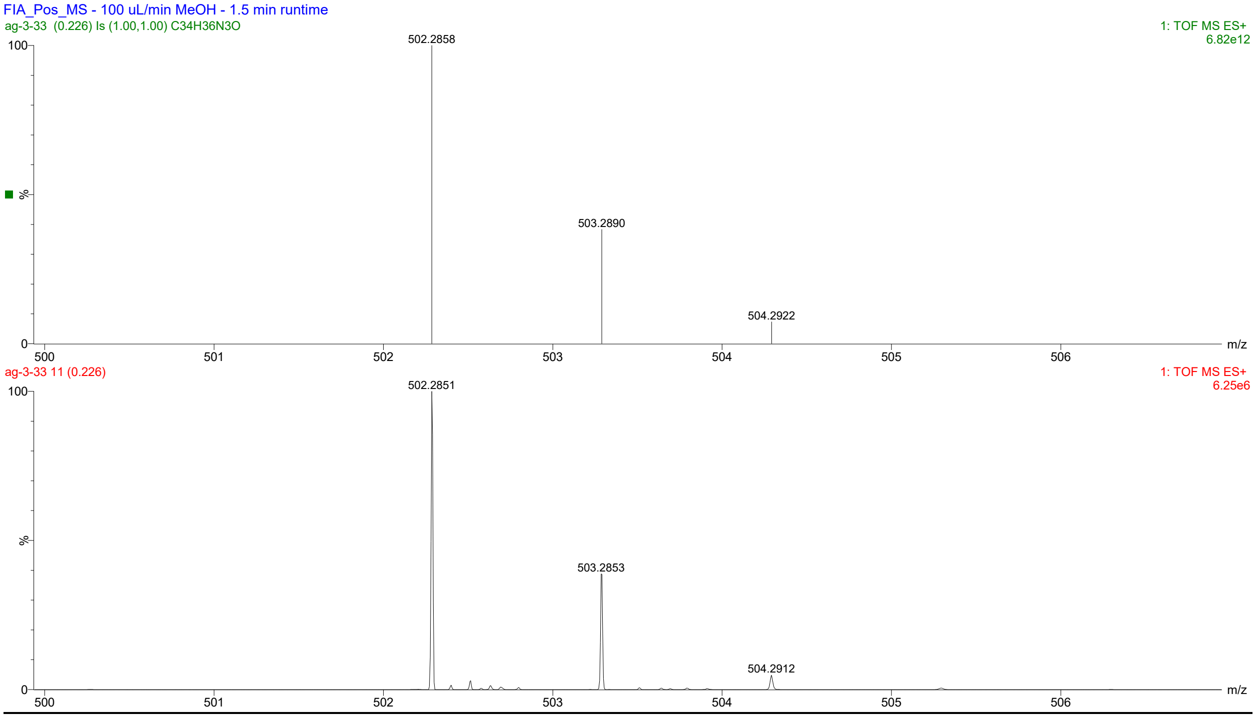

Figure S79. HRMS analysis (ESI, CH_3OH) report of (*S*)-**5f**

Compound 6

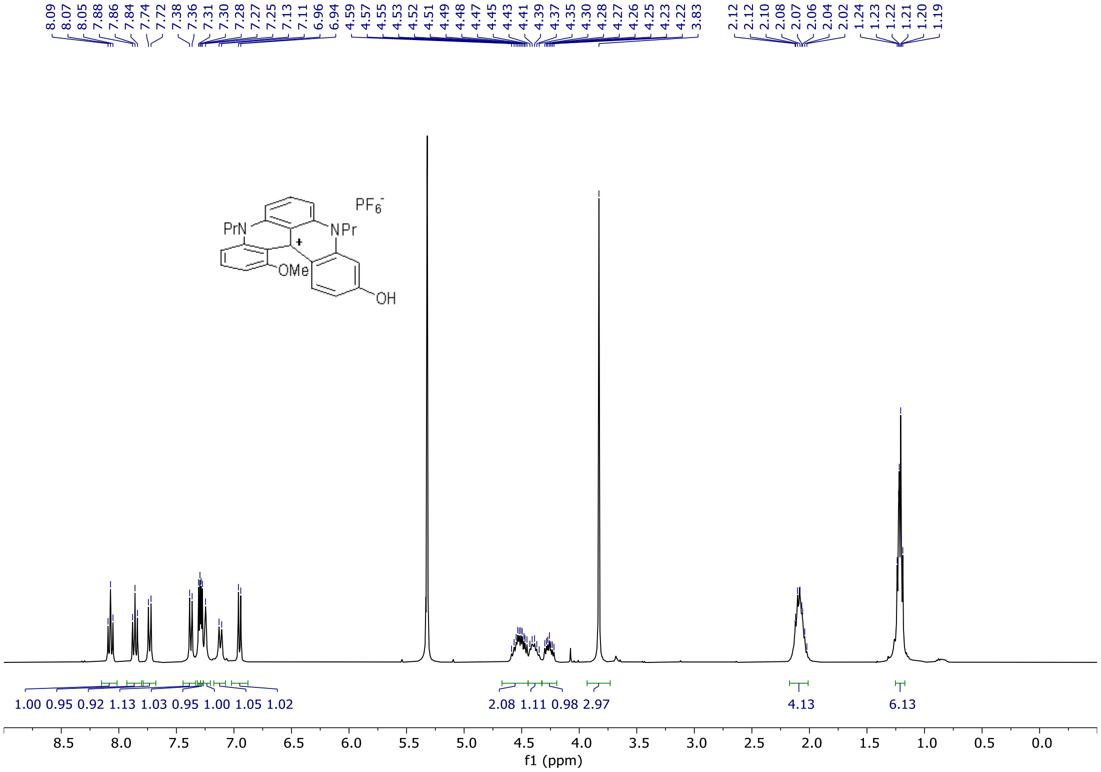

Figure S80. ^{1}H NMR (500 MHz, CD_2Cl_2) spectrum of **6**

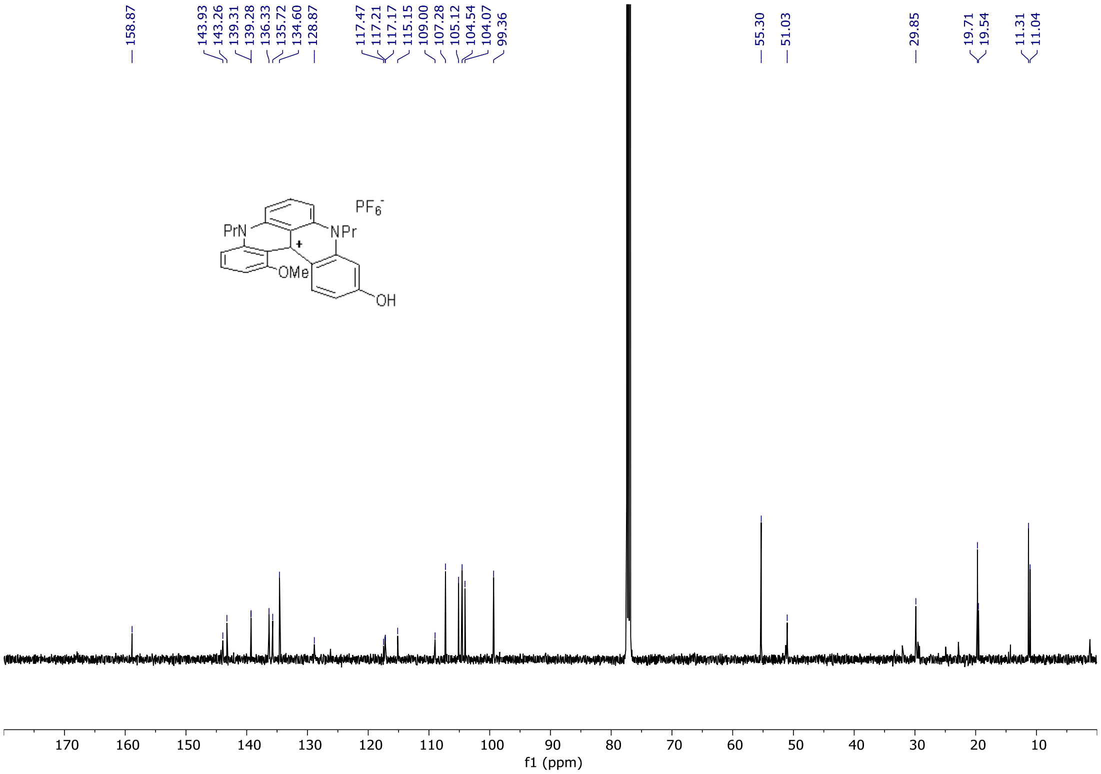

Figure S81. ^{13}C NMR (126 MHz, $CDCl_3$) spectrum of **6**

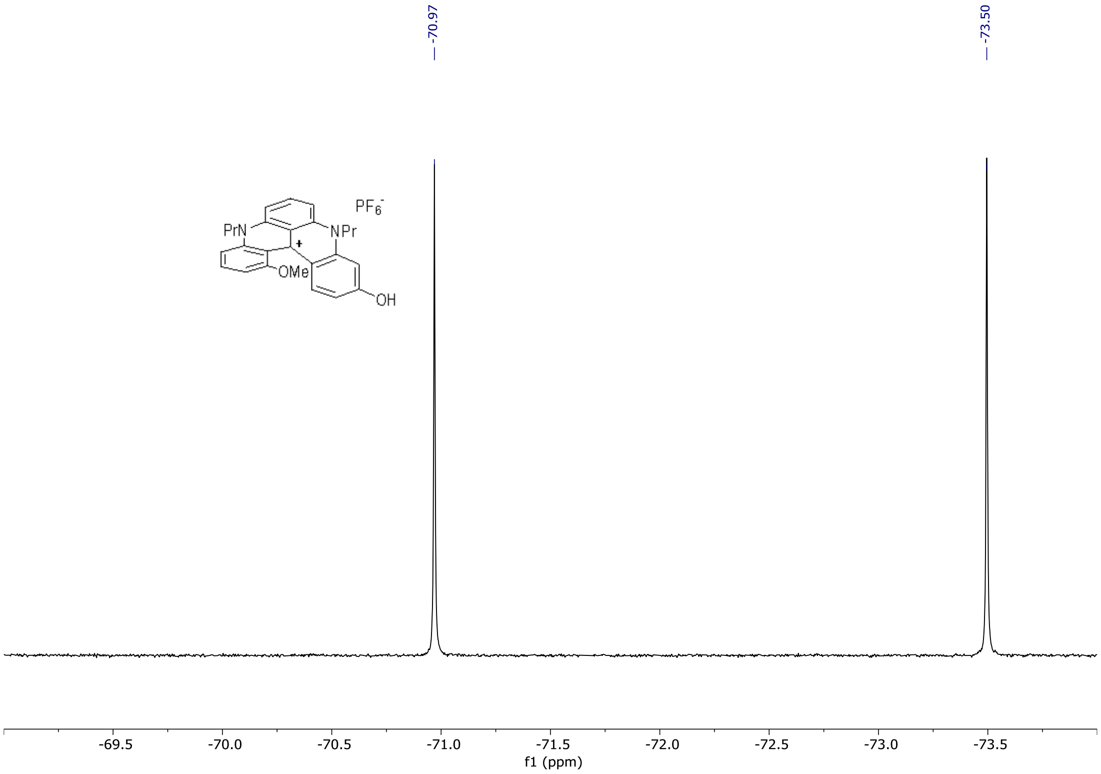

Figure S82. ^{19}F NMR (282 MHz, $CDCl_3$) spectrum of **6**

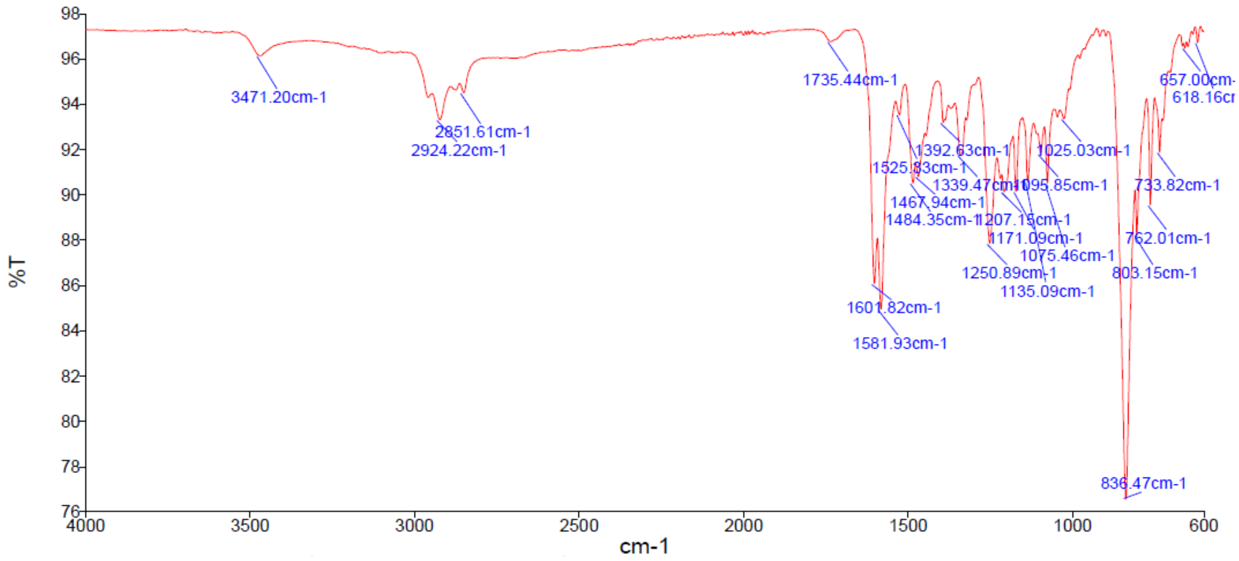

Figure S83. IR (neat) spectrum of **6**

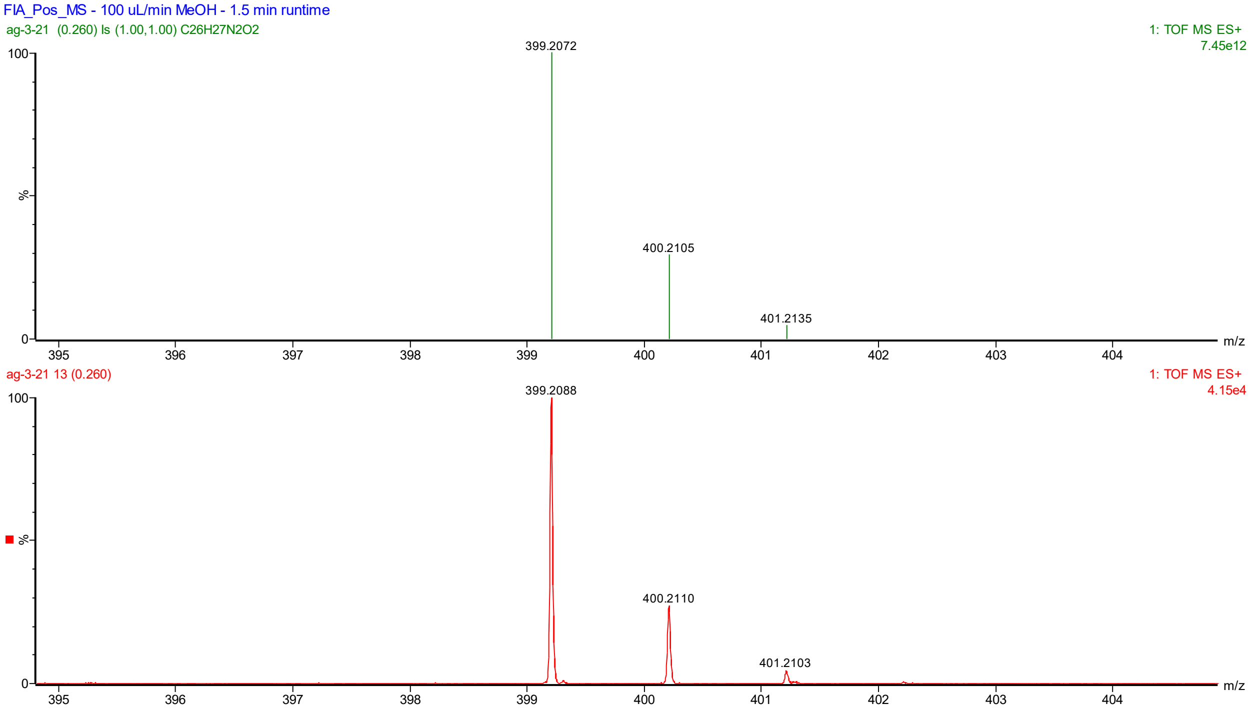

Figure S84. HRMS analysis (ESI, CH_3OH) report of **6**

Compound 7

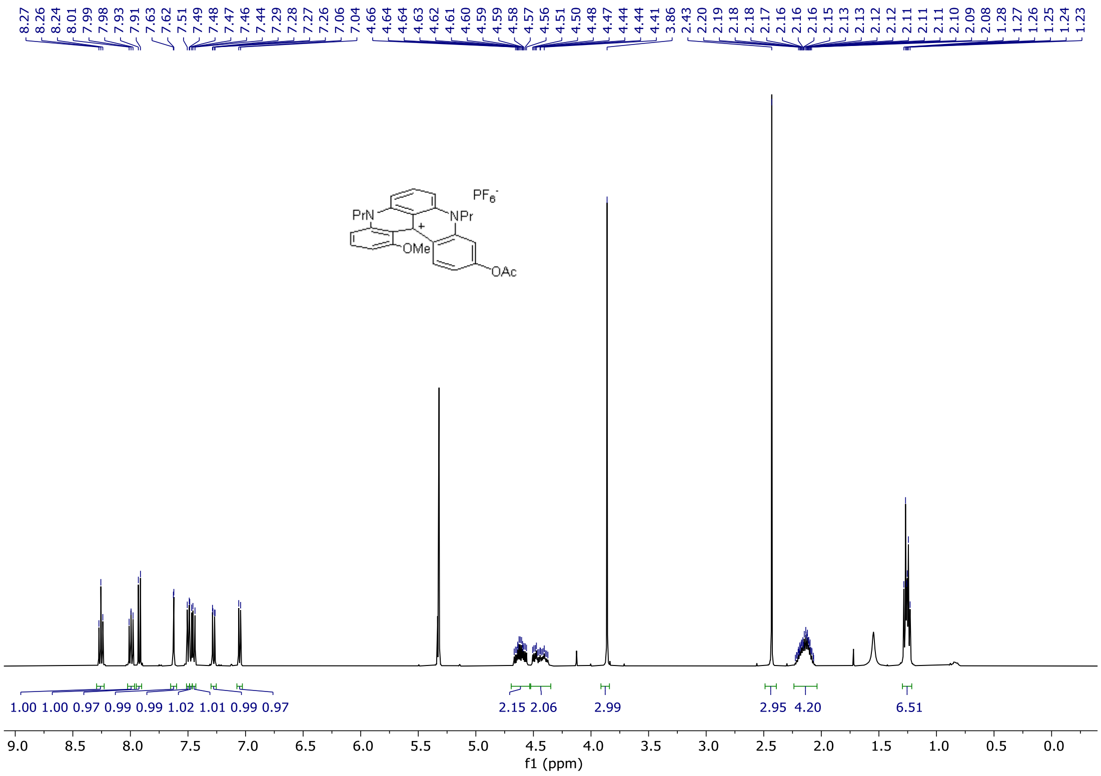

Figure S85. ^1H NMR (500 MHz, $CDCl_3$) spectrum of **7**

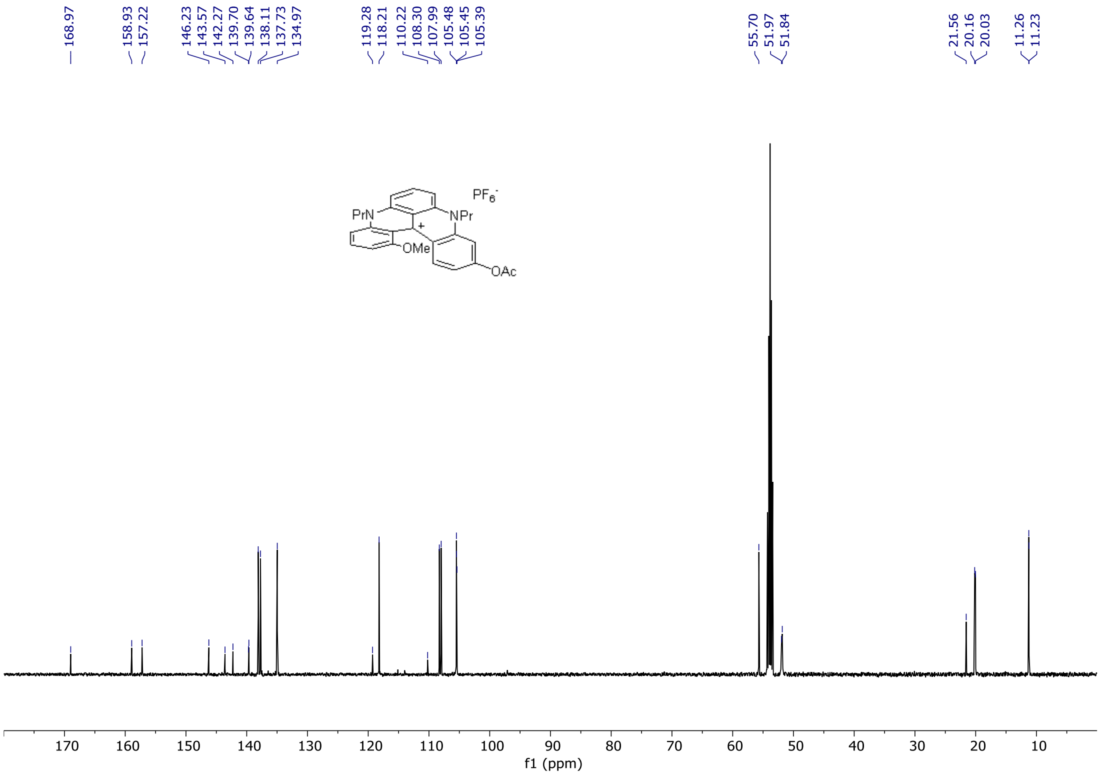

Figure S86. ^{13}C NMR (126 MHz, $CDCl_3$) spectrum of **7**

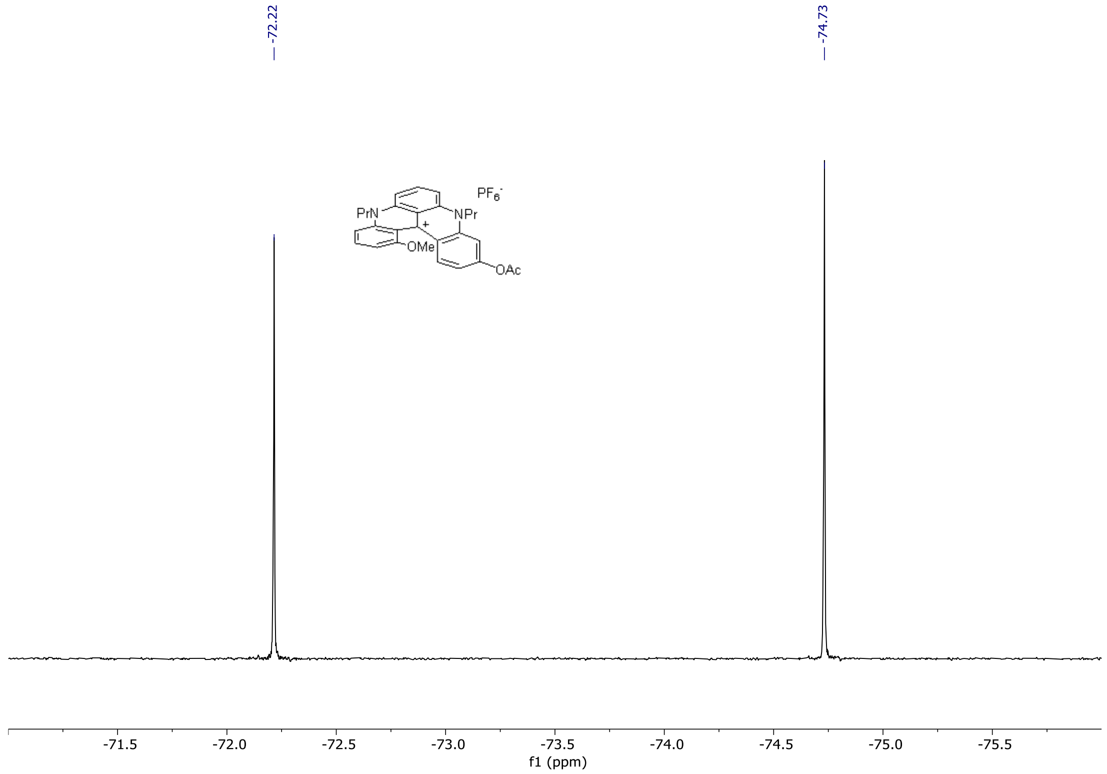

Figure S87. ^{19}F NMR (282 MHz, $CDCl_3$) spectrum of **7**

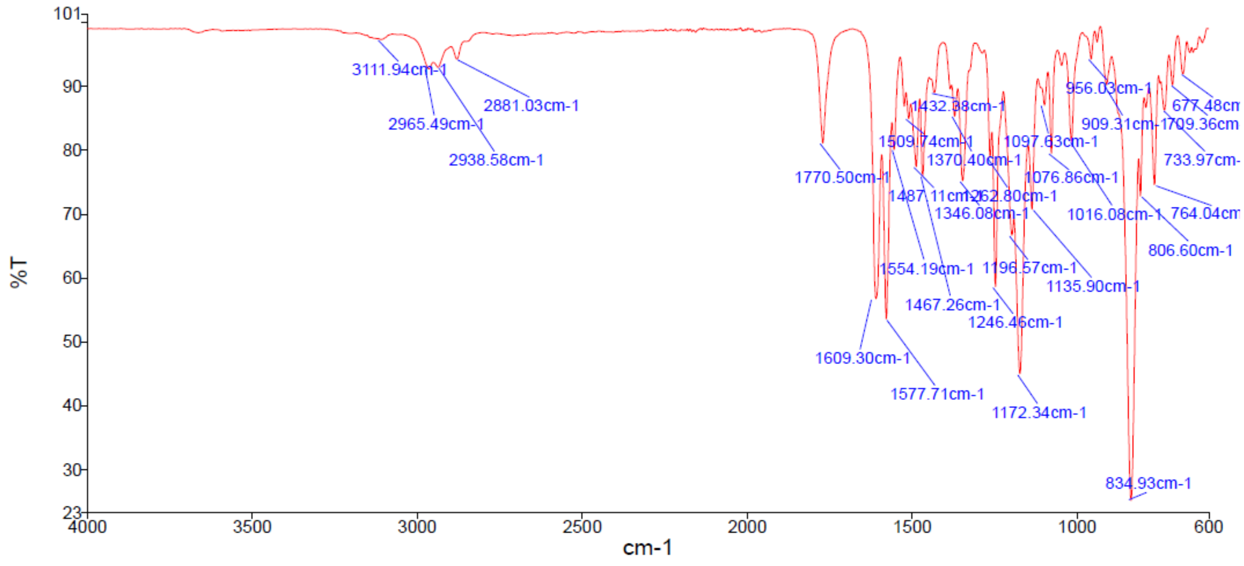

Figure S88. IR (neat) spectrum of **7**

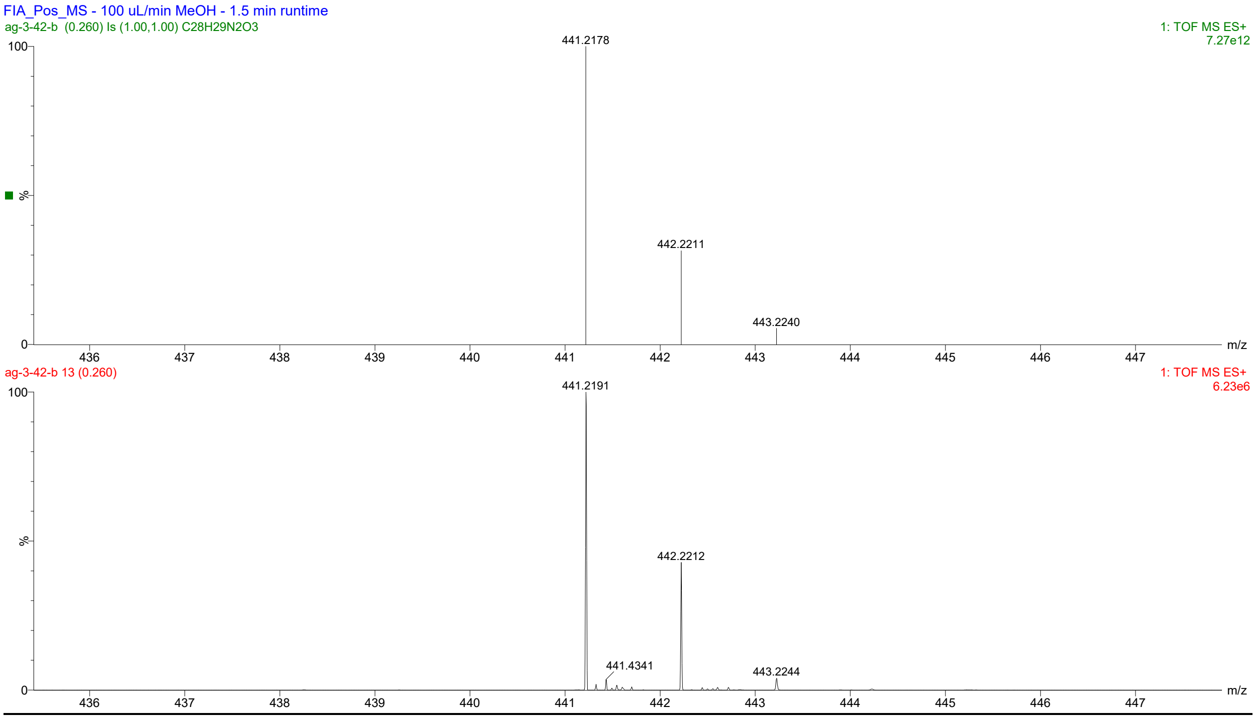

Figure S89. HRMS analysis (ESI, CH_3OH) report of **7**

Compound [**5b**][Δ-**8**]

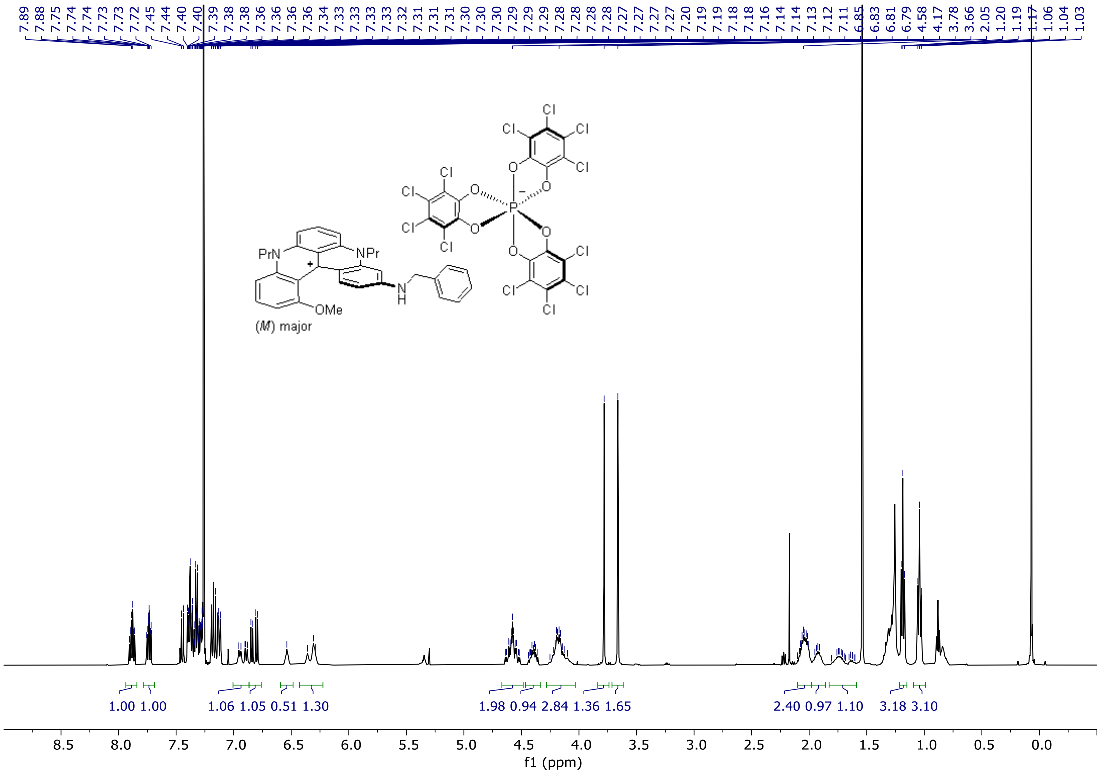

Figure S90. ^{1}H NMR (500 MHz, $CDCl_3$) spectrum of [**5b**][Δ-**8**]

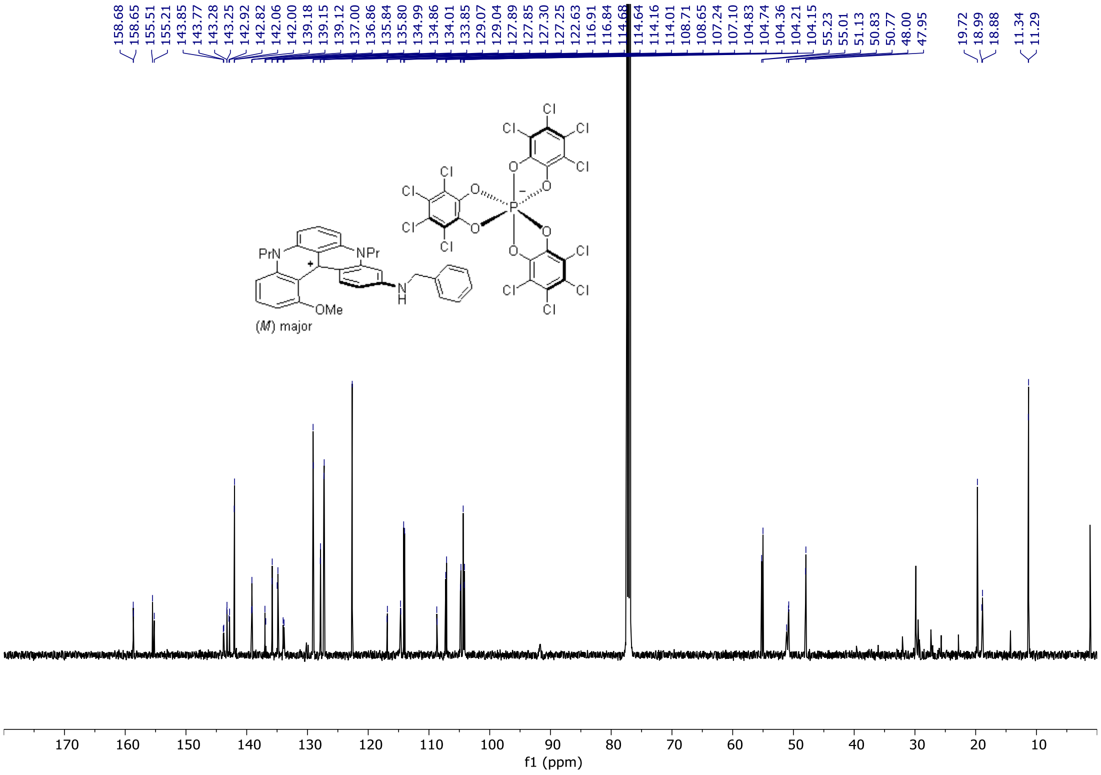

Figure S91. ^{13}C NMR (126 MHz, $CDCl_3$) spectrum of [**5b**][Δ-**8**]

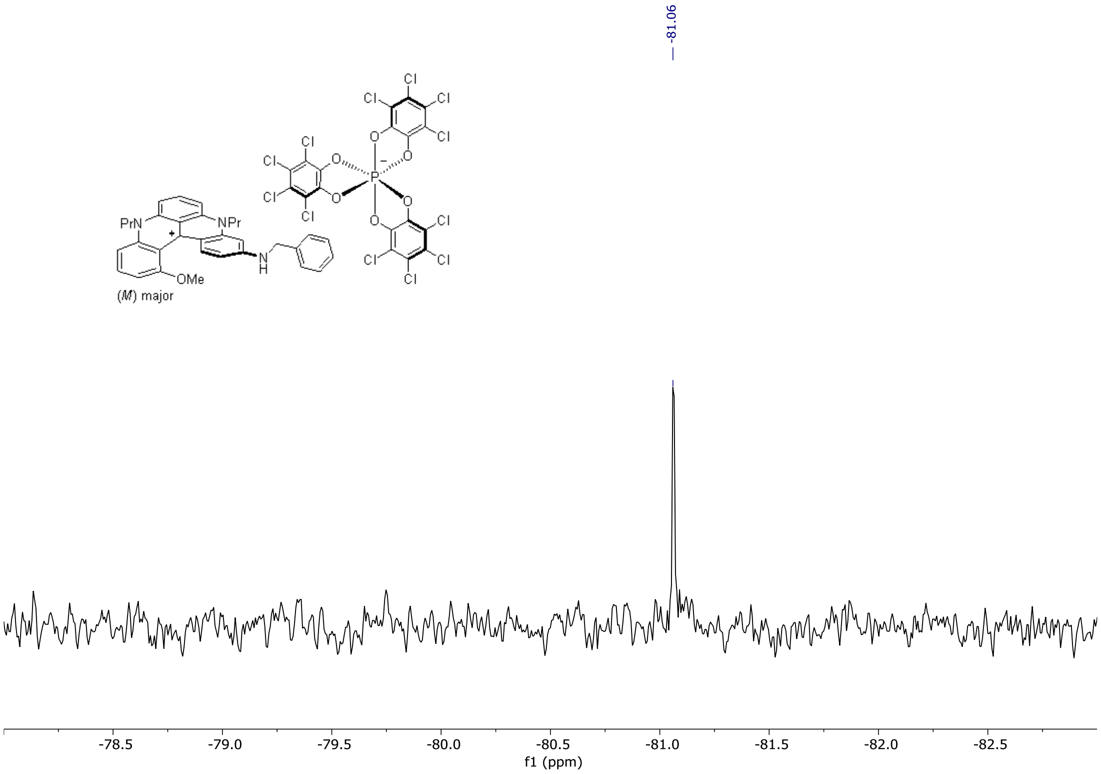

Figure S92. ^{31}P NMR (121 MHz, $CDCl_3$) spectrum of [**5b**][Δ-**8**]

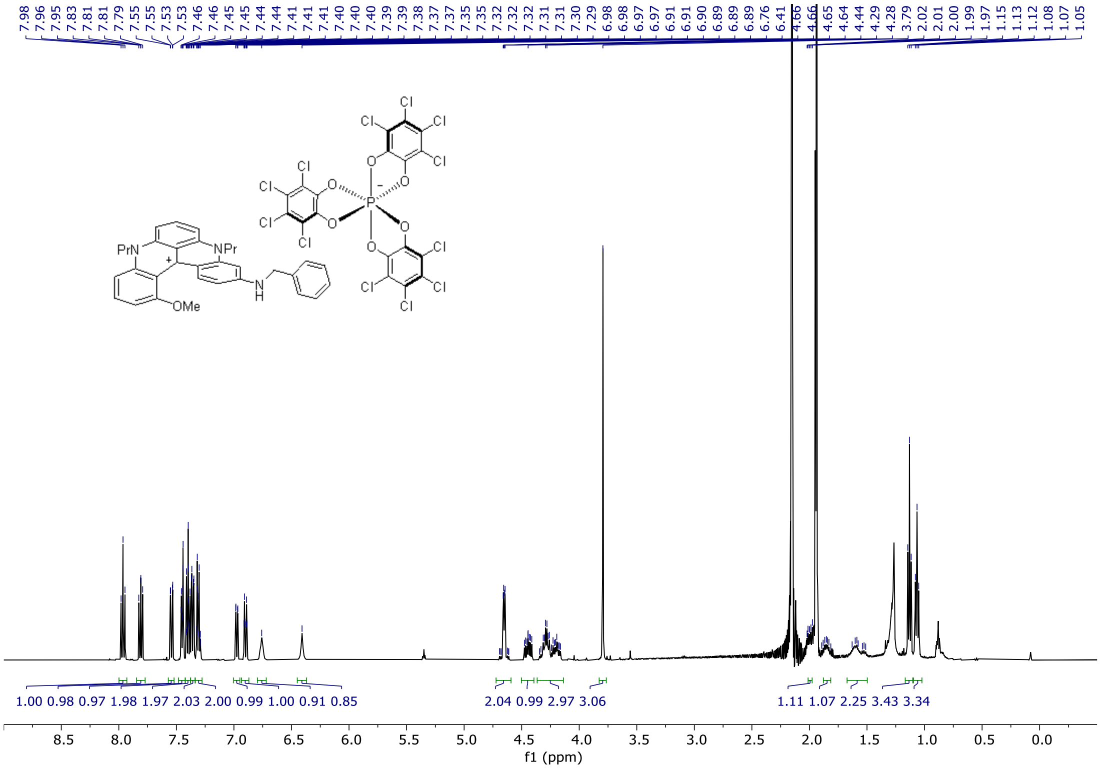

Figure S93. ^{1}H NMR (500 MHz, CD_3CN) spectrum of [**5b**][Δ-**8**]

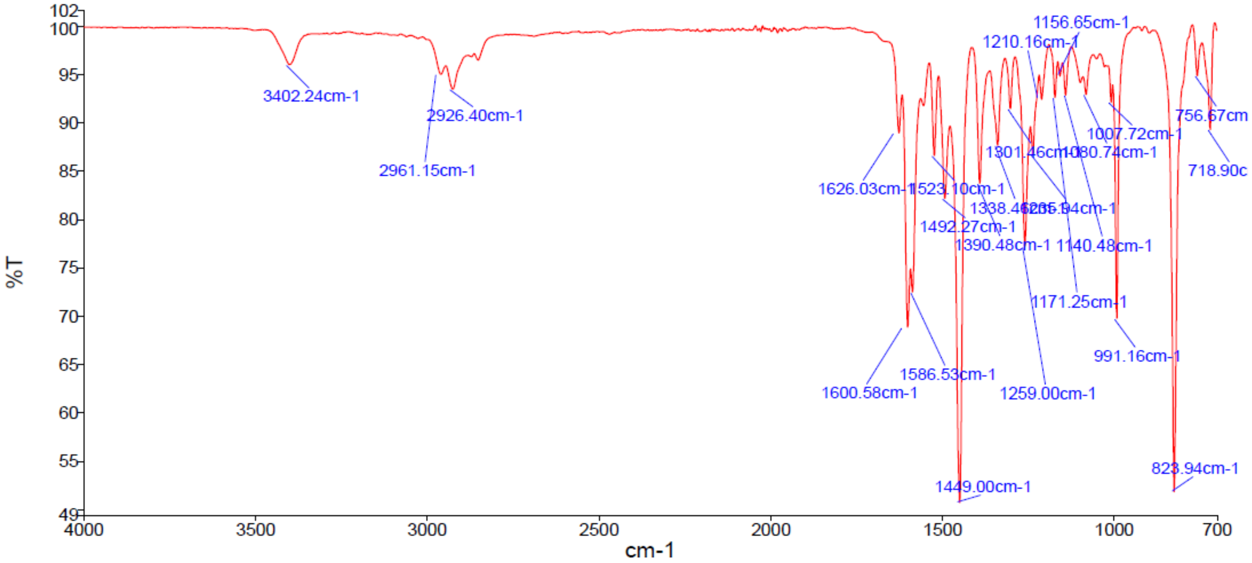

Figure S94. IR (neat) spectrum of [**5b**][Δ-**8**]

Compound [(*S*)-**5f**][Δ-**8**]

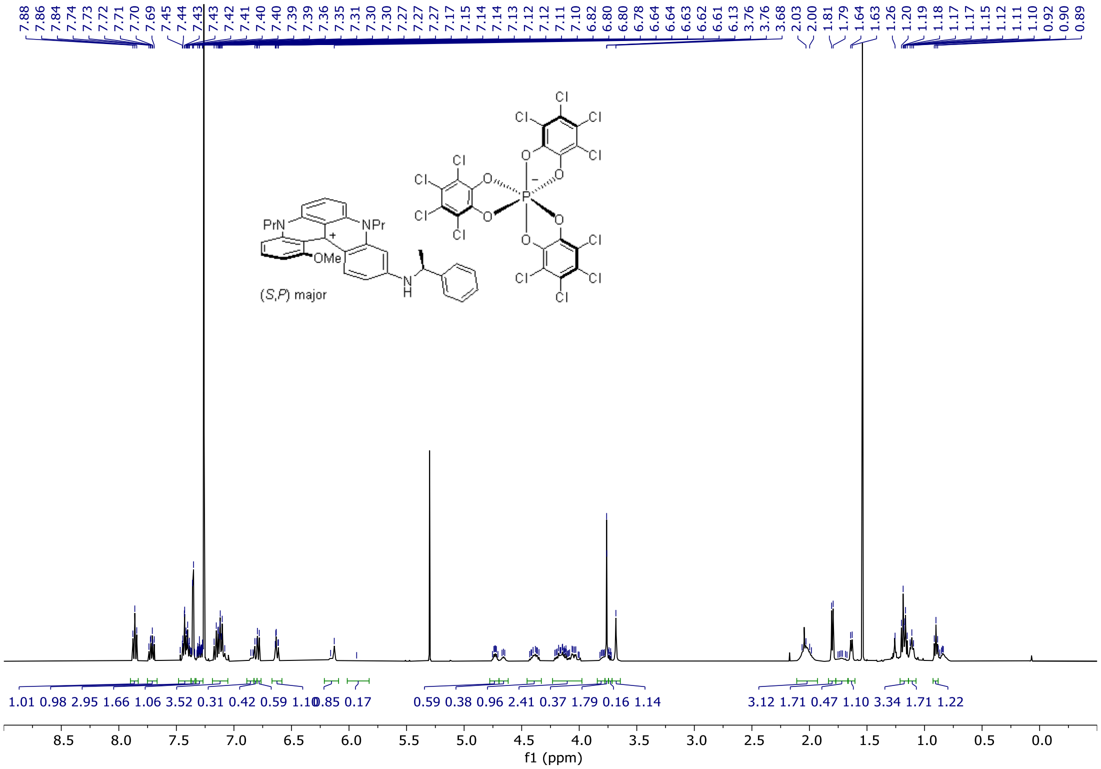

Figure S95. ^{1}H NMR (500 MHz, $CDCl_3$) spectrum of [(*S*)-**5f**][Δ-**8**]

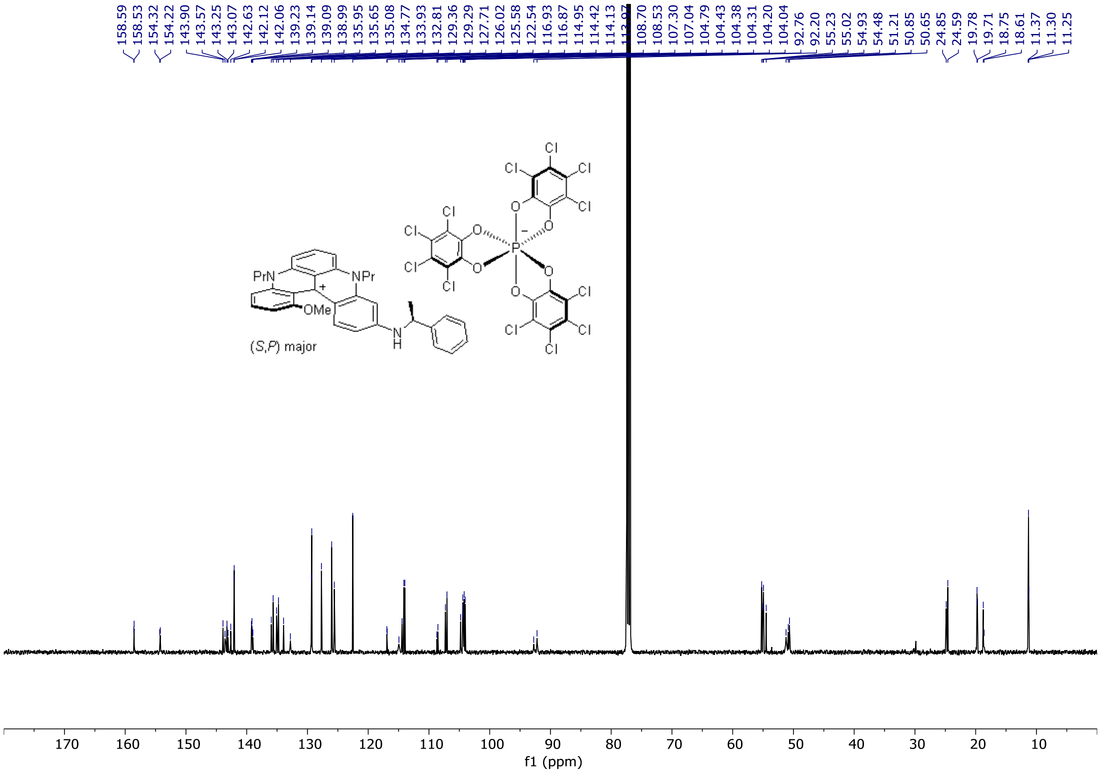

Figure S96. ^{13}C NMR (126 MHz, $CDCl_3$) spectrum of [(*S*)-**5f**][Δ-**8**]

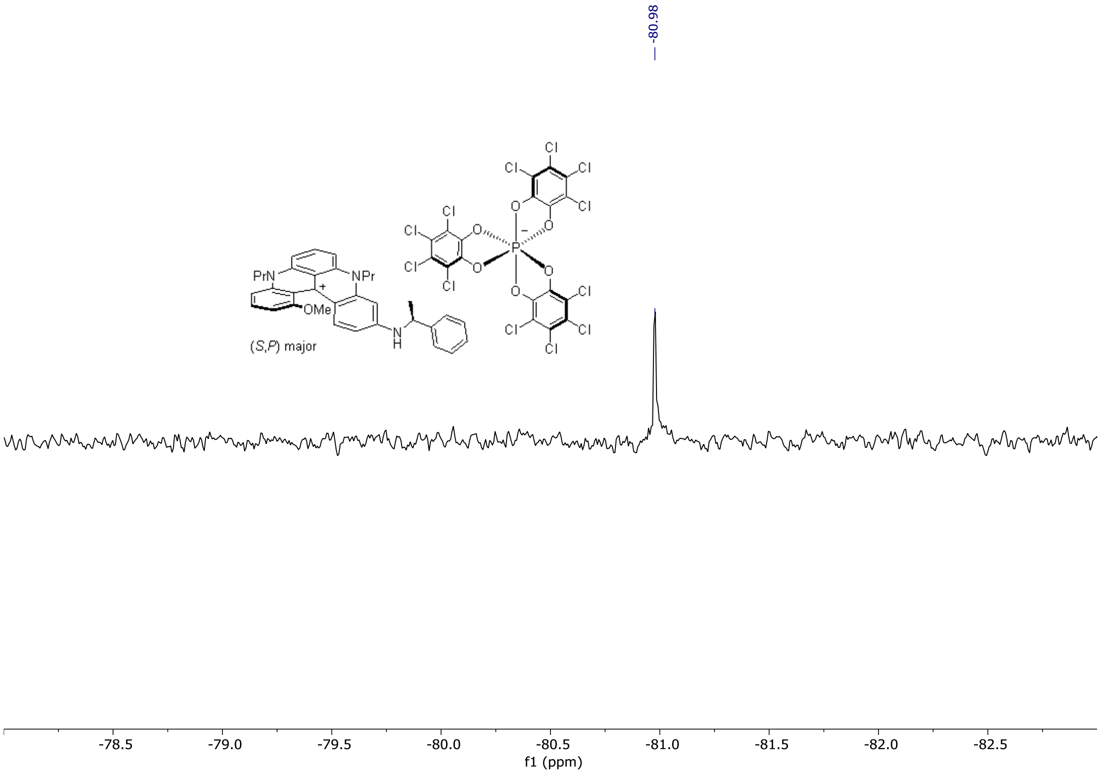

Figure S97. ^{31}P NMR (121 MHz, $CDCl_3$) spectrum of [(*S*)-**5f**][Δ-**8**]

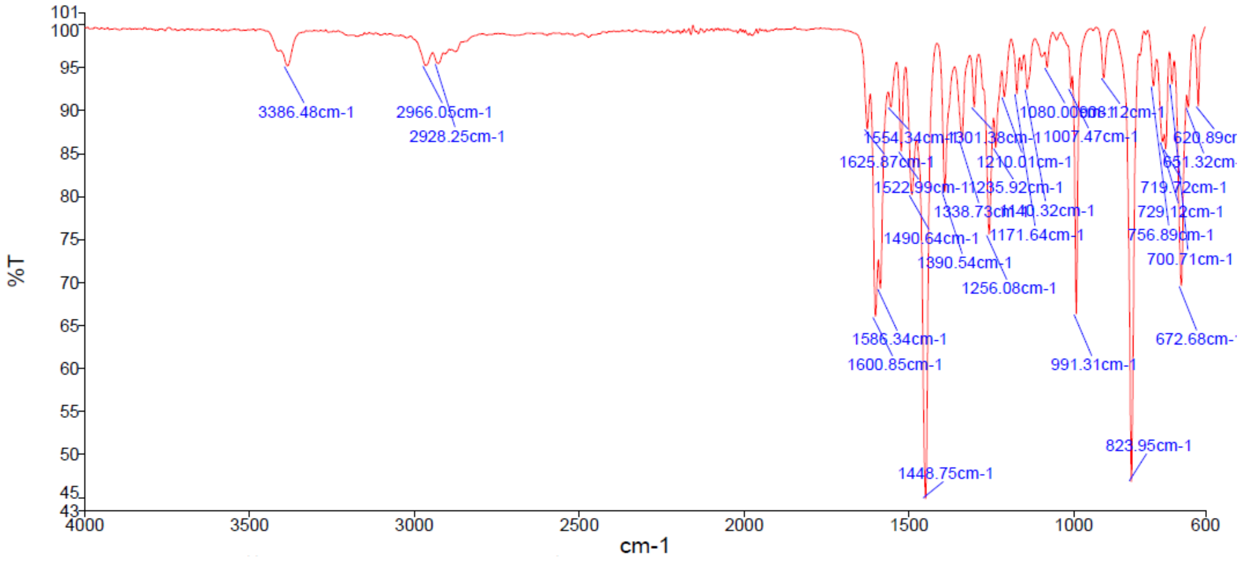

Figure S98. IR (neat) spectrum of [(*S*)-**5f**][Δ-**8**]

Compound [(*R*)-**5f**][Δ-**8**]

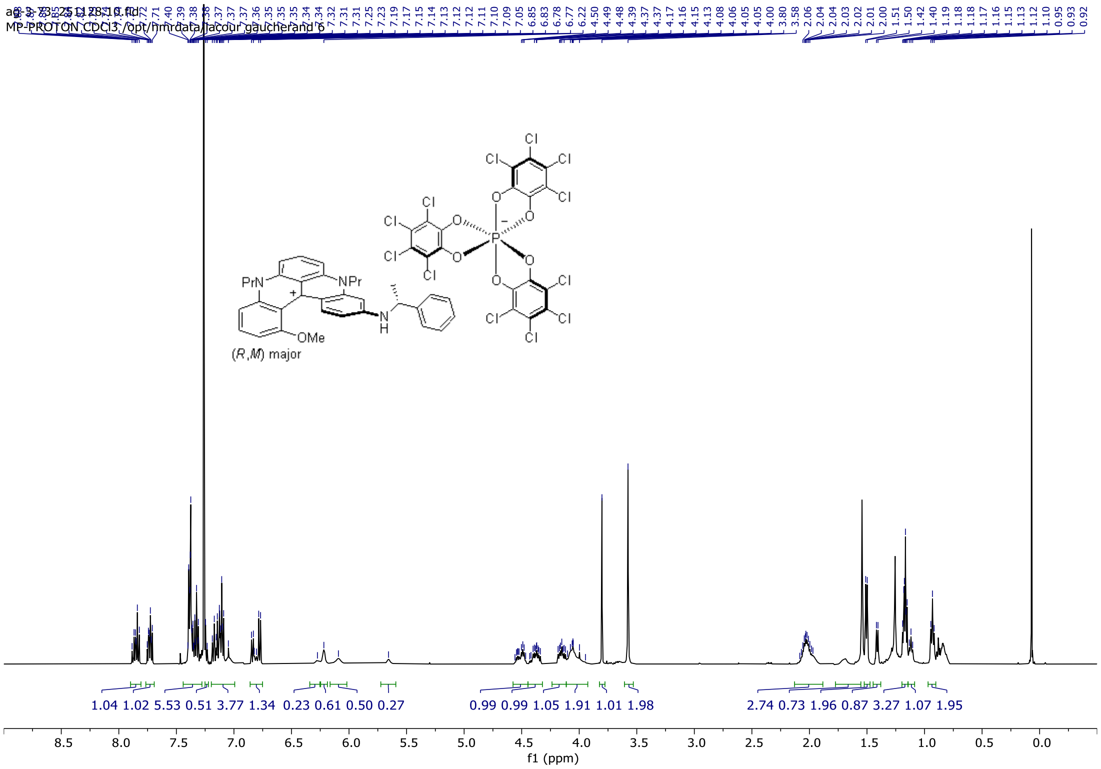

Figure S99. ^{1}H NMR (500 MHz, $CDCl_3$) spectrum of [(*R*)-**5f**][Δ-**8**]

8. Computational Section

Conformational searches and DFT calculations were run with Spartan'24 (Wavefunction Inc., Irvine, CA) with default parameters and convergence criteria. Conformational searches were run with the Monte Carlo algorithm and Merck Molecular Force Field (MMFF). All minima thus found were optimized at B3LYP-D3/6-31G(d) level in vacuo and frequency calculations were run at the same level. All conformers of **1-H**, **2-H** and **3-H** with internal energies within 2 kcal/mol from each respective lowest-energy conformer were then re-evaluated (single-point calculation) at B97M-V/6-311+G(2df,2p) level,[4] including SMD solvent model for $CHCl_2$. The coordinates for all relevant conformers are provided in the data repository.

For the input structures of *M*-(*R*)-**5f** and *P*-(*R*)-**5f** for ECD calculations, geometry optimizations and frequency calculations were run with Gaussian16,[5] with default grids and convergence criteria, at B3LYP-D3BJ/6-31+G(d) level, including IEF-PCM solvent model for $CHCl_3$. The coordinates are listed at the end of the present file. Excited-state calculations were run on all minima with relative population > 0.5% at 300K at B3LYP/def2-TZVP/PCM level, including 24 roots. Other functionals (CAM-B3LYP and M06-2X) were also tested on the lowest-energy conformers, yielding consistent results. Final spectra were Boltzmann-averaged at 300K using internal energies and plotted using SpecDis v.1.71 (T. Bruhn, A. Schaumlöffel, Y. Hemberger, G. Pescitelli, Berlin, Germany, 2017, http://specdis-software.jimdofree.com)[6] using the parameters listed in the figure captions. Compounds **1-3** were evaluated with the same approach used for **5f** except that the IEF-PCM solvent model for CH_3CN was adopted in the calculations. Only the lowest-energy conformer of each compound was considered. Natural Transition Orbitals (NTO), orbital composition analysis and Δr metrics[7] were plotted and calculated with Multiwfn v. 3.8.[8]

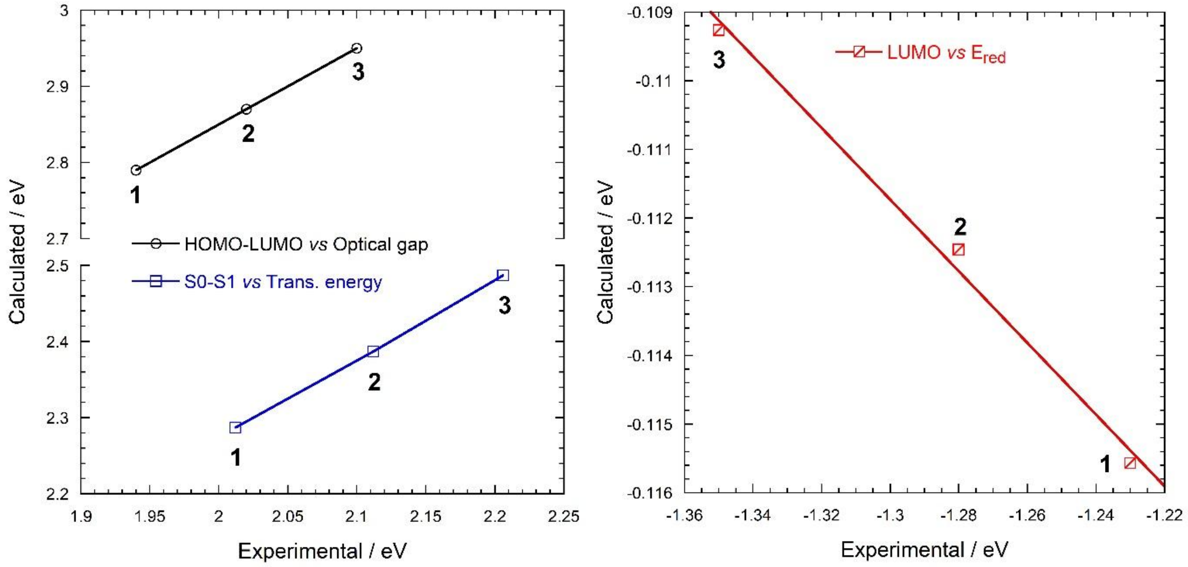

Figure S100. Correlations between experimental and calculated data for **1-3**: left, calculated HOMO-LUMO gap *vs* optical gap, and calculated S0-S1 *vs* experimental transition energy; right, calculated LUMO energy *vs* E_{red} potential. All data in eV; experimental quantities measured in acetonitrile; calculations run at B3LYP/def2-TZVP/PCM//B3LYP-D3BJ/6-31+G(d)/PCM level (IEF-PCM solvent model for CH_3CN).

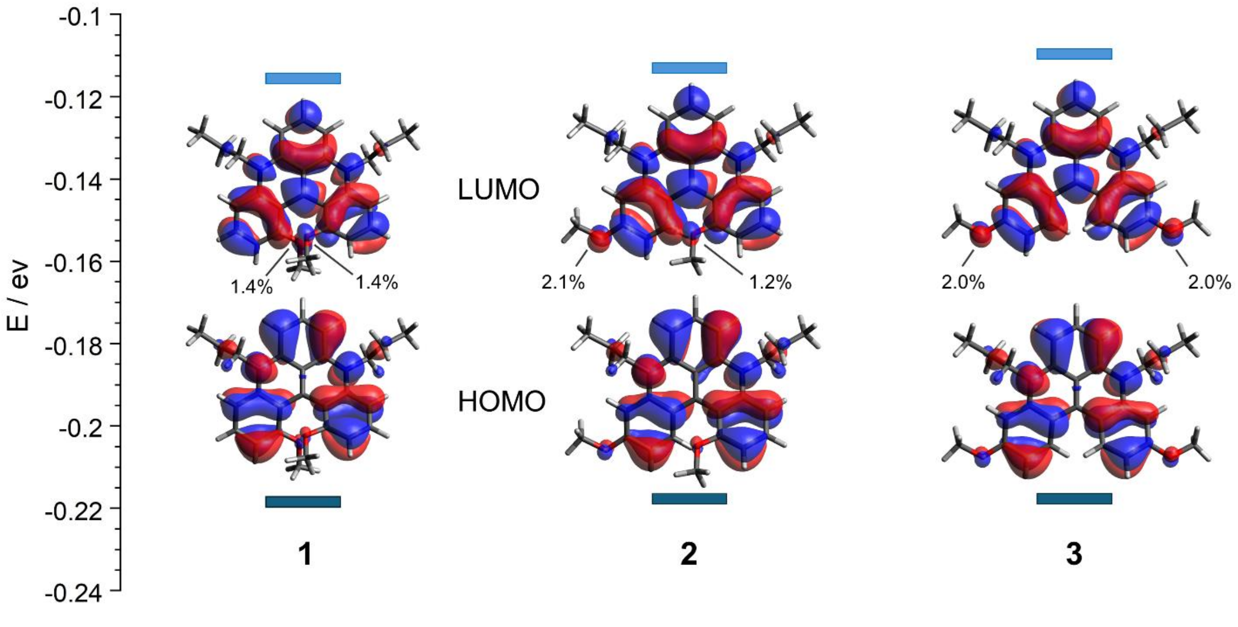

Figure S101. Kohn-Sham frontier orbitals calculated at B3LYP/def2-TZVP/PCM//B3LYP-D3BJ/6-31+G(d)/PCM level (IEF-PCM solvent model for CH_3CN) for the lowest-energy minima of compounds **1-3**. The numbers indicate the percent contribution of oxygen atoms to the LUMO.

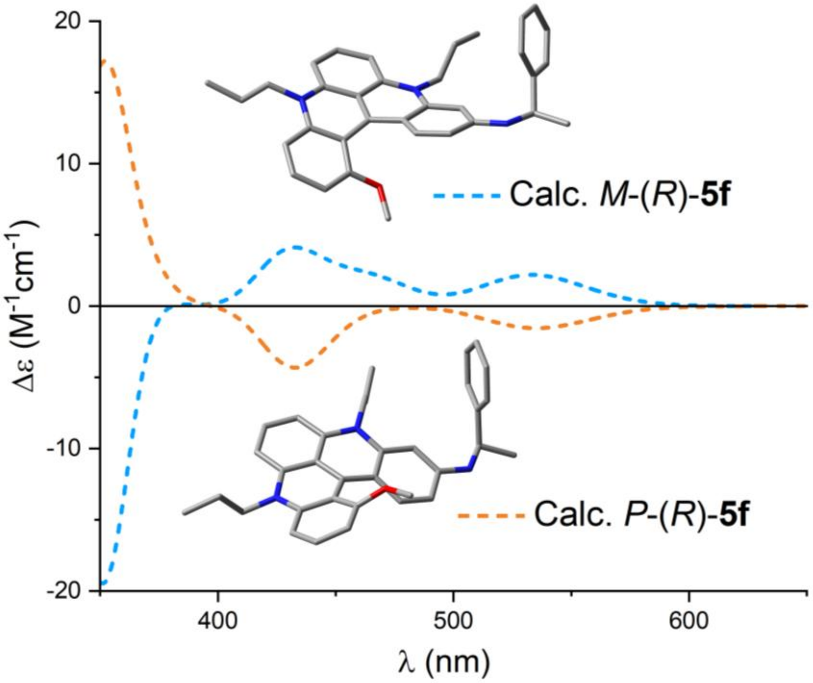

Figure S102. ECD spectra calculated at B3LYP/def2-TZVP/PCM//B3LYP-D3BJ/6-31+G(d)/PCM level (IEF-PCM solvent model for $CHCl_3$) for *M*-(*R*)-**5f** and *P*-(*R*)-**5f** diastereomers. In both cases, the calculated spectra were estimated as Boltzmann average of 3 low-energy minima. The lowest-energy minima, each with population > 95% at 300K, are shown as inset. Spectra plotted as sums of Gaussians with σ = 0.15 eV, red shifted by 25 nm, scaled by a factor 3.5.

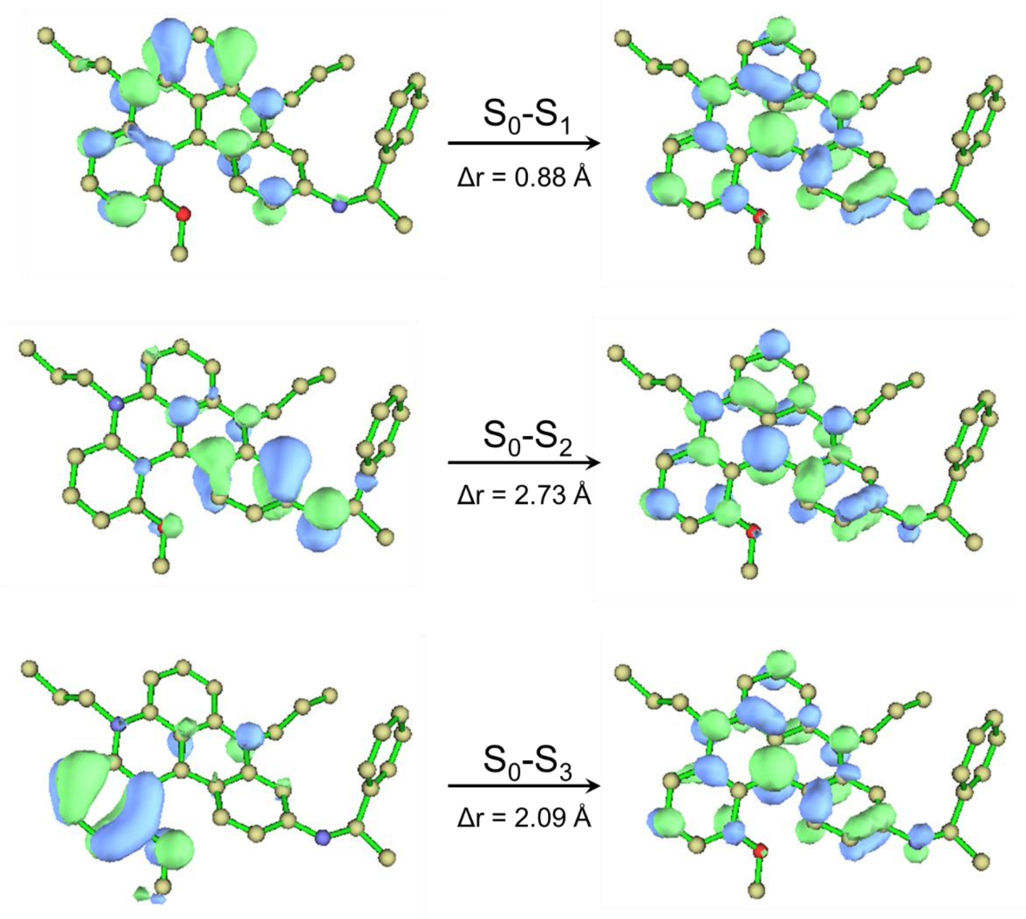

Figure S103. Natural Transition Orbitals (NTO) involved in the first 3 transitions computed for *M*-(*R*)-**5f** at B3LYP/def2-TZVP/PCM level. In all cases, the weight for the displayed contribution was > 99%. Isovalue 0.04. CT metric Δr values are also shown on each arrow.

Table S8. Relative energies (in kcal/mol) calculated for compounds **1-H**, **2-H** and **3-H** with two different methods as Boltzmann averages of all conformers within 2 kcal/mol from the lowest energy structure.

Compound	No. of conformers	ΔE (kcal/mol)[a]	ΔG (kcal/mol)[b]
**1-H**	29	+4.10	+3.05
**2-H**	34	0	+0.43
**3-H**	48	+0.14	0

[a] Internal energies calculated at B97M-V/6-311+G(2df,2p)/SMD//B3LYP-D3/6-31G(d) level with SMD solvent model for CH_2Cl_2.

[b] Gibbs energies calculated at B3LYP-D3/6-31G(d) level in vacuo.

9. Cartesian coordinates

Table S9. Atom coordinates for the lowest-energy minimum of **1** optimized at B3LYP-D3BJ/6-31+G(d)/PCM level (IEF-PCM solvent model for CH_3CN) level. SCF energy -1306.342642 h, no imaginary frequency.

Symbol	X	Y	Z
O	-1.3507160	-0.2096560	-2.7418690
N	-0.3372090	2.3950040	1.1325200
N	0.3372090	-2.3950040	1.1325200
C	0.0000000	0.0000000	3.9252210
H	0.0000000	0.0000000	5.0109160
C	-0.1622770	1.2107070	3.2568540
H	-0.2676380	2.1181730	3.8334020
C	-0.1512180	1.2302080	1.8564020
C	0.0000000	2.4589970	-0.2087760
C	0.0834870	3.7002120	-0.8780360
H	-0.1512530	4.6264220	-0.3748880
C	0.5349810	3.7404550	-2.1839870
C	0.9610190	2.5852590	-2.8584280
H	1.3887840	2.6705270	-3.8486220
C	0.8584880	1.3558620	-2.2258200
C	0.2690730	1.2524650	-0.9232460
C	0.0000000	0.0000000	-0.2740300
C	0.0000000	0.0000000	1.1379240
C	-0.2690730	-1.2524650	-0.9232460
C	-0.8584880	-1.3558620	-2.2258200
C	-1.9177090	-0.2250840	-4.0546860
H	-2.1938080	0.8085250	-4.2617400
H	-2.8074320	-0.8629040	-4.0862290
H	-1.1845490	-0.5689100	-4.7916410
C	-0.9610190	-2.5852590	-2.8584280
H	-1.3887840	-2.6705270	-3.8486220
C	-0.5349810	-3.7404550	-2.1839870
H	-0.6121190	-4.7005920	-2.6852460
C	-0.0834870	-3.7002120	-0.8780360
H	0.1512530	-4.6264220	-0.3748880
C	0.0000000	-2.4589970	-0.2087760
C	0.1512180	-1.2302080	1.8564020
C	0.1622770	-1.2107070	3.2568540
H	0.2676380	-2.1181730	3.8334020
C	-0.8003940	3.6073980	1.8352410
H	-1.4771860	3.2827110	2.6278150
H	-1.4098190	4.1819240	1.1355860
C	0.3373920	4.4630990	2.4036190
H	0.9467190	3.8579120	3.0843080
H	1.0012130	4.7751570	1.5896500
C	-0.2114860	5.6894630	3.1368320
H	-0.8034630	6.3211440	2.4634010
H	0.6049910	6.2988030	3.5382210
H	-0.8548950	5.3943820	3.9746740
C	0.8003940	-3.6073980	1.8352410

H	1.4771860	-3.2827110	2.6278150
H	1.4098190	-4.1819240	1.1355860
C	-0.3373920	-4.4630990	2.4036190
H	-0.9467190	-3.8579120	3.0843080
H	-1.0012130	-4.7751570	1.5896500
C	0.2114860	-5.6894630	3.1368320
H	0.8034630	-6.3211440	2.4634010
H	-0.6049910	-6.2988030	3.5382210
H	0.8548950	-5.3943820	3.9746740
H	0.6121190	4.7005920	-2.6852460
O	1.3507160	0.2096560	-2.7418690
C	1.9177090	0.2250840	-4.0546860
H	1.1845490	0.5689100	-4.7916410
H	2.1938080	-0.8085250	-4.2617400
H	2.8074320	0.8629040	-4.0862290

Table S10. Atom coordinates for the lowest-energy minimum of **2** optimized at B3LYP-D3BJ/6-31+G(d)/PCM level (IEF-PCM solvent model for CH_3CN) level. SCF energy -1306.348482 h, no imaginary frequency.

Symbol	X	Y	Z
N	-2.8959230	-0.6045020	-0.4570960
N	1.7985600	-1.3826230	0.4415580
C	-1.0614000	-3.7186260	0.3109340
H	-1.2568900	-4.7813470	0.4162800
C	-2.1261840	-2.8679560	0.0223560
H	-3.1177810	-3.2844850	-0.0789270
C	-1.8925200	-1.4938600	-0.1064220
C	-2.7409460	0.7589040	-0.2689890
C	-3.8405820	1.6371630	-0.3844520
H	-4.8188770	1.2782000	-0.6679790
C	-3.6823310	2.9756370	-0.0736910
C	-2.4696230	3.4838130	0.4141180
H	-2.4116350	4.5137720	0.7405870
C	-1.3744560	2.6370930	0.5182880
C	-1.4518590	1.2817450	0.0564700
C	-0.3170370	0.3996410	-0.0345550
C	-0.5639030	-0.9876850	0.0638030
C	1.0316880	0.8086020	-0.2692940
C	1.3633690	2.0619260	-0.8522300
C	2.6627890	2.4446060	-1.0516260
H	2.9111690	3.3918360	-1.5176910
C	3.7113060	1.5776950	-0.6517550
C	3.4335760	0.3186990	-0.1342900
H	4.2449550	-0.3514600	0.0968320
C	2.0934820	-0.0995530	0.0173670
C	0.5168910	-1.8942420	0.3150030
C	0.2451990	-3.2622730	0.4558740
H	1.0310930	-3.9792060	0.6437250
C	-4.1818320	-1.1379120	-0.9461180
H	-3.9636600	-2.0543780	-1.4972070
H	-4.5744590	-0.4327440	-1.6806360
C	-5.2042840	-1.3996810	0.1652900

H	-4.7809310	-2.0990700	0.8952110
H	-5.4011400	-0.4681930	0.7077820
C	-6.5072690	-1.9618250	-0.4085360
H	-6.9605840	-1.2615480	-1.1206860
H	-7.2341200	-2.1478630	0.3890600
H	-6.3322990	-2.9092380	-0.9328260
C	2.8765050	-2.2589050	0.9401800
H	2.4365030	-2.9277350	1.6817570
H	3.5832480	-1.6318070	1.4865010
C	3.5843620	-3.0531600	-0.1635080
H	2.8552350	-3.6823970	-0.6860660
H	3.9871990	-2.3614380	-0.9118740
C	4.7084690	-3.9155240	0.4155550
H	5.4649820	-3.2963530	0.9129330
H	5.2069730	-4.4849810	-0.3757670
H	4.3204680	-4.6300450	1.1516150
H	-4.5370590	3.6401960	-0.1552270
O	-0.2142390	2.9948360	1.1132390
C	-0.0189460	4.3592060	1.4985270
H	-0.1193270	5.0238840	0.6340600
H	0.9985400	4.4057010	1.8857370
H	-0.7286210	4.6482910	2.2806090
H	0.5636490	2.7126610	-1.1808230
O	4.9589330	2.0521580	-0.8525070
C	6.0800350	1.2337710	-0.4924700
H	6.0911080	0.3121180	-1.0836340
H	6.0574530	0.9967610	0.5763670
H	6.9618090	1.8309850	-0.7217680

Table S11. Atom coordinates for the lowest-energy minimum of **3** optimized at B3LYP-D3BJ/6-31+G(d)/PCM level (IEF-PCM solvent model for CH_3CN) level. SCF energy -1306.356974 h, no imaginary frequency.

Symbol	X	Y	Z
N	-0.2850610	2.4101590	1.0038880
N	0.2850610	-2.4101590	1.0038880
C	0.0000000	0.0000000	3.7891370
H	0.0000000	0.0000000	4.8748130
C	-0.1339160	1.2129040	3.1199160
H	-0.2147470	2.1218750	3.6975310
C	-0.1296410	1.2317000	1.7191020
C	0.0000000	2.4648640	-0.3487360
C	0.1025120	3.7071690	-1.0094260
H	-0.0570540	4.6257870	-0.4699520
C	0.4773230	3.7580460	-2.3469390
C	0.7923250	2.5717370	-3.0513730
H	1.1600860	2.6423340	-4.0691000
C	0.6503110	1.3602050	-2.4236930
C	0.1982840	1.2543030	-1.0794880
C	0.0000000	0.0000000	-0.4152480
C	0.0000000	0.0000000	0.9995780
C	-0.1982840	-1.2543030	-1.0794880
C	-0.6503110	-1.3602050	-2.4236930

C	-0.7923250	-2.5717370	-3.0513730
H	-1.1600860	-2.6423340	-4.0691000
C	-0.4773230	-3.7580460	-2.3469390
C	-0.1025120	-3.7071690	-1.0094260
H	0.0570540	-4.6257870	-0.4699520
C	0.0000000	-2.4648640	-0.3487360
C	0.1296410	-1.2317000	1.7191020
C	0.1339160	-1.2129040	3.1199160
H	0.2147470	-2.1218750	3.6975310
C	-0.6703350	3.6390880	1.7244450
H	-1.3391780	3.3463950	2.5354070
H	-1.2739420	4.2446770	1.0459920
C	0.5231850	4.4375350	2.2606140
H	1.1104380	3.8067670	2.9374700
H	1.1886820	4.7031790	1.4314440
C	0.0572630	5.7012380	2.9878510
H	-0.5072790	6.3589300	2.3157850
H	0.9134870	6.2664160	3.3705150
H	-0.5896180	5.4527870	3.8379980
C	0.6703350	-3.6390880	1.7244450
H	1.3391780	-3.3463950	2.5354070
H	1.2739420	-4.2446770	1.0459920
C	-0.5231850	-4.4375350	2.2606140
H	-1.1104380	-3.8067670	2.9374700
H	-1.1886820	-4.7031790	1.4314440
C	-0.0572630	-5.7012380	2.9878510
H	0.5072790	-6.3589300	2.3157850
H	-0.9134870	-6.2664160	3.3705150
H	0.5896180	-5.4527870	3.8379980
H	-0.9401710	-0.4624230	-2.9533810
O	-0.6144180	-4.9034200	-3.0483150
C	-0.3342640	-6.1526320	-2.4019380
H	-1.0224910	-6.3191680	-1.5665480
H	0.7019170	-6.1807510	-2.0489890
H	-0.4873310	-6.9152520	-3.1646810
H	0.9401710	0.4624230	-2.9533810
O	0.6144180	4.9034200	-3.0483150
C	0.3342640	6.1526320	-2.4019380
H	0.4873310	6.9152520	-3.1646810
H	1.0224910	6.3191680	-1.5665480
H	-0.7019170	6.1807510	-2.0489890

Table S12. Atom coordinates for the lowest-energy minimum of *M*-(*R*)-**5f** optimized at B3LYP-D3BJ/6-31+G(d)/PCM level (IEF-PCM solvent model for $CHCl_3$) level, accounting for 97.7% population at 300 K (based on internal energies). SCF energy -1556.899239 h, no imaginary frequency.

Symbol	X	Y	Z
N	-3.6926040	2.3768900	0.2262820
O	1.7842500	3.0923770	-1.0800050
N	-0.5895270	-1.1522960	-0.8978920
N	4.0100340	-0.7752310	0.5693460
C	2.0632570	-3.7044300	-0.5691690

H	2.1941370	-4.7737360	-0.7050240
C	0.8183700	-3.1434700	-0.8353230
H	0.0082700	-3.7890080	-1.1420210
C	0.6313230	-1.7646520	-0.6582480
C	-0.8450400	0.1280900	-0.4362910
C	-2.1524640	0.6356050	-0.4186610
H	-2.9758290	0.0320290	-0.7605450
C	-2.4274120	1.8855210	0.1509340
C	-4.8842440	1.7312760	-0.3141730
H	-4.7067810	1.5289440	-1.3803260
C	-1.3558960	2.6435220	0.7171720
H	-1.5740180	3.5802110	1.2219920
C	-0.0734650	2.1822670	0.6345560
H	0.7249350	2.7549710	1.0882940
C	0.2439910	0.9362910	0.0219170
C	1.5710070	0.4423410	-0.0829440
C	1.7352970	-0.9565260	-0.2398770
C	2.7721470	1.2398320	0.0057380
C	2.8456640	2.6148430	-0.3903220
C	1.7481130	4.4755090	-1.4381370
H	0.7864680	4.6198750	-1.9302130
H	2.5602860	4.7210590	-2.1307220
H	1.8089490	5.1106520	-0.5473370
C	3.9837150	3.3682280	-0.1302690
H	4.0369880	4.4126890	-0.4078270
C	5.0951760	2.7468060	0.4547480
H	5.9830370	3.3374870	0.6591840
C	5.1176610	1.3877430	0.7124780
H	6.0300120	0.9399600	1.0779270
C	3.9751720	0.6050970	0.4381420
C	2.9933100	-1.5681020	0.0558190
C	3.1440370	-2.9491570	-0.1197220
H	4.0854580	-3.4431110	0.0711080
C	-1.6637660	-1.9259290	-1.5481640
H	-1.1899900	-2.5983480	-2.2657370
H	-2.2599780	-1.2287320	-2.1401200
C	-2.5476180	-2.7100360	-0.5705090
H	-1.9154850	-3.3441450	0.0618940
H	-3.0664850	-2.0182440	0.1013440
C	-3.5651600	-3.5679310	-1.3251790
H	-4.2227220	-2.9469670	-1.9445210
H	-4.1978440	-4.1226860	-0.6256590
H	-3.0652520	-4.2919530	-1.9804010
C	5.1944090	-1.4281040	1.1562200
H	4.8556500	-2.3507390	1.6321590
H	5.5591150	-0.7912120	1.9643930
C	6.3073360	-1.7169300	0.1427190
H	5.9093390	-2.3338280	-0.6711160
H	6.6366960	-0.7779450	-0.3163050
C	7.4904260	-2.4230450	0.8094400
H	7.9149060	-1.8114840	1.6149650
H	8.2857820	-2.6200580	0.0833080

H	7.1857920	-3.3835940	1.2427420
H	-3.8153720	3.2765560	0.6716300
C	-6.0602630	2.7092660	-0.2014950
H	-6.9602460	2.2588240	-0.6276350
H	-5.8465040	3.6382270	-0.7423980
H	-6.2653890	2.9450610	0.8492980
C	-5.2053040	0.4047440	0.3677750
C	-5.8799350	-2.0338750	1.5888690
C	-4.8802030	0.1705190	1.7079200
C	-5.8679690	-0.5939190	-0.3559540
C	-6.2095290	-1.8047150	0.2504070
C	-5.2130190	-1.0428930	2.3153040
H	-4.3512020	0.9334940	2.2718810
H	-6.1139650	-0.4254870	-1.4025920
H	-6.7197120	-2.5717250	-0.3251840
H	-4.9481640	-1.2154030	3.3549750
H	-6.1349480	-2.9789110	2.0601680

Table S13. Atom coordinates for the lowest-energy minimum of *P*-(*R*)-**5f** optimized at B3LYP-D3BJ/6-31+G(d)/PCM level (IEF-PCM solvent model for $CHCl_3$) level, accounting for 94.8% population at 300 K (based on internal energies). SCF energy -1556.898032 h, no imaginary frequency.

Symbol	X	Y	Z
N	-3.7348330	-1.8261120	1.4390560
O	1.2763770	-2.9391840	-1.1022670
N	-0.5458640	1.4627950	-0.0969470
N	4.1791510	0.5283680	0.3641840
C	2.3836920	3.7119050	-0.1944880
H	2.6027910	4.7702430	-0.2990030
C	1.0566560	3.2960740	-0.2337010
H	0.2771570	4.0368800	-0.3392480
C	0.7572830	1.9333080	-0.0899390
C	-0.8394850	0.1832280	0.3445160
C	-2.1600850	-0.1799710	0.6417830
H	-2.9590790	0.5310170	0.5216790
C	-2.4608990	-1.4559030	1.1329920
C	-4.8803050	-0.9180330	1.4706820
H	-4.6273950	-0.0635820	2.1167330
C	-1.3964310	-2.3846010	1.3495610
H	-1.6161650	-3.3523680	1.7916570
C	-0.1128150	-2.0436270	1.0296680
H	0.6859300	-2.7428640	1.2399580
C	0.2180320	-0.7716590	0.4791920
C	1.5431240	-0.3950060	0.1324710
C	1.8255610	0.9912970	0.0445080
C	2.6451830	-1.3023660	-0.0876960
C	2.4927460	-2.6330400	-0.5951740
C	1.0247800	-4.2704590	-1.5576160
H	-0.0186950	-4.2744440	-1.8719600
H	1.6695600	-4.5213890	-2.4067330
H	1.1719360	-4.9933160	-0.7474820
C	3.5696880	-3.5106260	-0.6181400

H	3.4556650	-4.5244860	-0.9783770
C	4.8316450	-3.0539210	-0.2141030
H	5.6717720	-3.7416870	-0.2301010
C	5.0507150	-1.7329120	0.1339880
H	6.0582970	-1.4075850	0.3470130
C	3.9699560	-0.8248060	0.1450130
C	3.1761570	1.4543860	0.1140740
C	3.4412560	2.8236080	-0.0097200
H	4.4509940	3.2073130	0.0146570
C	-1.6384630	2.3683650	-0.5003100
H	-1.2451690	3.0219950	-1.2815430
H	-2.4099600	1.7593740	-0.9761420
C	-2.2273540	3.1920330	0.6508610
H	-1.4271460	3.7495780	1.1507670
H	-2.6513590	2.5187990	1.4043390
C	-3.3032450	4.1534580	0.1395060
H	-4.1143540	3.6082400	-0.3564060
H	-3.7372450	4.7270490	0.9651350
H	-2.8858090	4.8666750	-0.5819320
C	5.5115740	1.0122920	0.7687870
H	5.3601800	1.9163490	1.3623680
H	5.9389480	0.2741980	1.4505680
C	6.4599630	1.2859780	-0.4042250
H	6.0078900	2.0244570	-1.0760810
H	6.5864190	0.3714300	-0.9945040
C	7.8183860	1.7862940	0.0930180
H	8.3028690	1.0431390	0.7380730
H	8.4901010	1.9893720	-0.7473580
H	7.7126680	2.7128540	0.6705060
H	-3.8501900	-2.7205890	1.8976230
C	-6.0720580	-1.6546870	2.0931160
H	-6.9329760	-0.9836330	2.1508550
H	-6.3537680	-2.5173470	1.4790720
H	-5.8330060	-1.9984610	3.1063320
C	-5.2301060	-0.3741390	0.0907700
C	-5.9309650	0.6547350	-2.4272380
C	-5.7026140	0.9371980	-0.0342540
C	-5.1097270	-1.1667380	-1.0557260
C	-5.4574500	-0.6558770	-2.3082870
C	-6.0542400	1.4509500	-1.2852120
H	-5.7867020	1.5642270	0.8509580
H	-4.7258100	-2.1792370	-0.9668500
H	-5.3544130	-1.2791960	-3.1924380
H	-6.4175970	2.4716760	-1.3678840
H	-6.1974690	1.0530140	-3.4022370

10. References

1 C. Herse, D. Bas, F. C. Krebs, T. Bürgi, J. Weber, T. Wesolowski, B. W. Laursen and J. Lacour, *Angew. Chem. Int. Ed.*, 2003, **42**, 3162-3166.

2 D. Conreaux, N. Mehanna, C. Herse and J. Lacour, *J. Org. Chem.*, 2011, **76**, 2716-2722.

3 C. M. Cardona, W. Li, A. E. Kaifer, D. Stockdale and G. C. Bazan, *Adv. Mater.*, 2011, **23**, 2367-2371.

4 N. Mardirossian and M. Head-Gordon, *J. Chem. Phys.*, 2015, **142**.

5 M. J. Frisch, G. W. Trucks, H. B. Schlegel, G. E. Scuseria, M. A. Robb, J. R. Cheeseman, G. Scalmani, V. Barone, G. A. Petersson, H. Nakatsuji, X. Li, M. Caricato, A. V. Marenich, J. Bloino, B. G. Janesko, R. Gomperts, B. Mennucci, H. P. Hratchian, J. V. Ortiz, A. F. Izmaylov, J. L. Sonnenberg, Williams, F. Ding, F. Lipparini, F. Egidi, J. Goings, B. Peng, A. Petrone, T. Henderson, D. Ranasinghe, V. G. Zakrzewski, J. Gao, N. Rega, G. Zheng, W. Liang, M. Hada, M. Ehara, K. Toyota, R. Fukuda, J. Hasegawa, M. Ishida, T. Nakajima, Y. Honda, O. Kitao, H. Nakai, T. Vreven, K. Throssell, J. A. Montgomery Jr., J. E. Peralta, F. Ogliaro, M. J. Bearpark, J. J. Heyd, E. N. Brothers, K. N. Kudin, V. N. Staroverov, T. A. Keith, R. Kobayashi, J. Normand, K. Raghavachari, A. P. Rendell, J. C. Burant, S. S. Iyengar, J. Tomasi, M. Cossi, J. M. Millam, M. Klene, C. Adamo, R. Cammi, J. W. Ochterski, R. L. Martin, K. Morokuma, O. Farkas, J. B. Foresman and D. J. Fox, Wallingford, CT, 2016.

6 T. Bruhn, A. Schaumlöffel, Y. Hemberger and G. Bringmann, *Chirality*, 2013, **25**, 243-249.

7 C. A. Guido, P. Cortona, B. Mennucci and C. Adamo, *J. Chem. Theory Comput.*, 2013, **9**, 3118-3126.

8 (a) T. Lu, *J. Chem. Phys.*, 2024, **161**, 082503; (b) T. Lu and F. Chen, *J. Comput. Chem.*, 2012, **33**, 580-592.
